# Reduced Symmetry Metal–Organic Cage‐to‐Framework Materials

**DOI:** 10.1002/anie.8127392

**Published:** 2026-05-14

**Authors:** Cameron J. T. Cox, Aaron H. Bernardino, Louise Male, Rebecca L. Greenaway, Georgia R. F. Orton, James E. M. Lewis

**Affiliations:** ^1^ School of Chemistry University of Birmingham, Molecular Sciences Building, Edgbaston Birmingham UK; ^2^ Department of Chemistry Imperial College London Molecular Sciences Research Hub, White City Campus, Wood Lane London UK

## Abstract

Metal–organic frameworks (MOFs) are typically assembled from inflexible, 2D aromatic linker units to provide structural predictability, rigidity and prevent architectural collapse. Limiting the pool of structural units from which these materials are derived, however, inevitably restricts the diversity of architectures that can be realised. In this work, we have explored organic cages with 1,2,3‐triazole struts as 3D linkers for Ag(I)‐based MOFs. These linkers are unusual in two key facets. First, the cage structure is semi‐rigid, providing both shape persistence and (limited) conformational freedom. Second, in contrast to the reticular design concepts of traditional MOFs, minor structural modifications at locations remote from the coordinating units were found to induce profound changes to the resultant MOF architectures, which included 2D honeycomb structures, 2D corrugated sheets and an interpenetrated 3D network. This is the first report of the incorporation of reduced symmetry cage linkers into metal–organic cage‐to‐framework structures, providing a blueprint for the introduction of low‐symmetry and chiral intrinsic porosity into framework materials.

## Graphical abstract

A series of Ag(I) MOFs was synthesised from organic cages of varying structure and symmetry as linkers. Minor variations in the cage linkers were shown to significantly influence the structures of the resulting MOFs. This work represents the first incorporation of reduced symmetry (including chiral), intrinsically porous building blocks into MOFs, and demonstrates the potential for porous linkers to tune structures and properties.

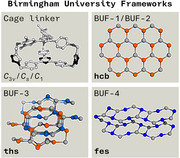

## 1 Introduction

Metal–organic frameworks (MOFs) have become one of the most intensely studied classes of porous materials over the last three decades. Since early seminal work [1, 2, 3, 4, 5], MOFs have been investigated for their use in gas storage/separation [6, 7, 8], sensing [9], catalysis [10], drug delivery [11, 12] and water harvesting [13]. Indeed, the impact of MOFs on such diverse areas was recently recognised through the award of the 2025 Nobel Prize in Chemistry for their development [14].

Most commonly, MOFs are assembled from single metal nodes and single, high‐symmetry linkers that are generally 2D, aromatic and rigid to promote stability and porosity arising from the void space between building blocks. Exclusive use of such components, however, has two significant drawbacks. First, it is challenging to precision engineer pore spaces, particularly anisotropic voids, even with the site‐specific incorporation of multiple linkers [15] or the use of reduced symmetry [16] and chiral [17] linkers. Second, to promote porosity, there is, by necessity, limited contact between linkers. While this allows isoreticular assemblies to be purposefully designed and generated from geometrically similar linkers [18], and functional moieties to be predictably incorporated within MOF pores [19, 20], it negates the potential to use interactions between linkers to gain fine control over network topologies.

Cage‐to‐framework materials [21, 22, 23, 24, 25, 26, 27, 28, 29], in which discrete molecular cages [30, 31] (including coordination cages) [32, 33, 34, 35, 36, 37], the structures of which can be engineered with precision at the molecular level, are incorporated into networks, could potentially provide a solution to both these challenges. Rosseinsky and Cooper reported the first cage‐based MOF in 2010 [38]. In comparison to prototypical MOFs, porosity in such cage‐to‐framework materials can be defined by intralinker void space (*intrinsic* porosity of the cages) instead/in addition to interlinker pores (*extrinsic* porosity). Indeed, recent work by Natarajan has shown that organic cages can retain their ability to bind polycyclic aromatic hydrocarbons when incorporated into MOFs [39]. Despite the potential of this approach, of the small number of previously reported MOFs using cages [40, 41, 42, 43, 44, 45, 46, 47, 48] none have explored the potential to use reduced symmetry cages, with anisotropic pores, as linkers. Furthermore, an as‐yet underexplored property of cage‐based linkers is their *thickness*. The intrinsic porosity and shape persistence of cages means that extrinsic porosity may not be significantly important in cage‐to‐framework materials. As such, interactions between cage linkers could be exploited to control framework topology. The impact of structural modifications and symmetry of cage linkers on their resultant frameworks has, however, not been studied, despite the importance of understanding this relationship to the future design of these materials.

In this work, we report the solid‐state structures of four new, cage‐based MOFs that include the first examples of systems assembled from reduced symmetry cage [49, 50, 51, 52, 53, 54] linkers. In contrast to design strategies based on reticular chemistry, minor structural modifications at locations remote from the coordination units were shown to lead to dramatic changes to the framework structure due to the close packing of the cages and interactions between their surfaces. Linker disorder observed within frameworks assembled from reduced symmetry cages showed that precise ordering is not a prerequisite for preparing crystalline MOFs derived from low symmetry linkers. Furthermore, disorder in the linker orientation results in materials exhibiting a statistical distribution of node environments, something typically associated with multivariate frameworks. This work therefore demonstrates the potential for the bottom‐up, precision engineering of bespoke metal–organic frameworks through careful structural tuning of cages as building blocks.

## 2 Results and Discussion

**FIGURE 1:**
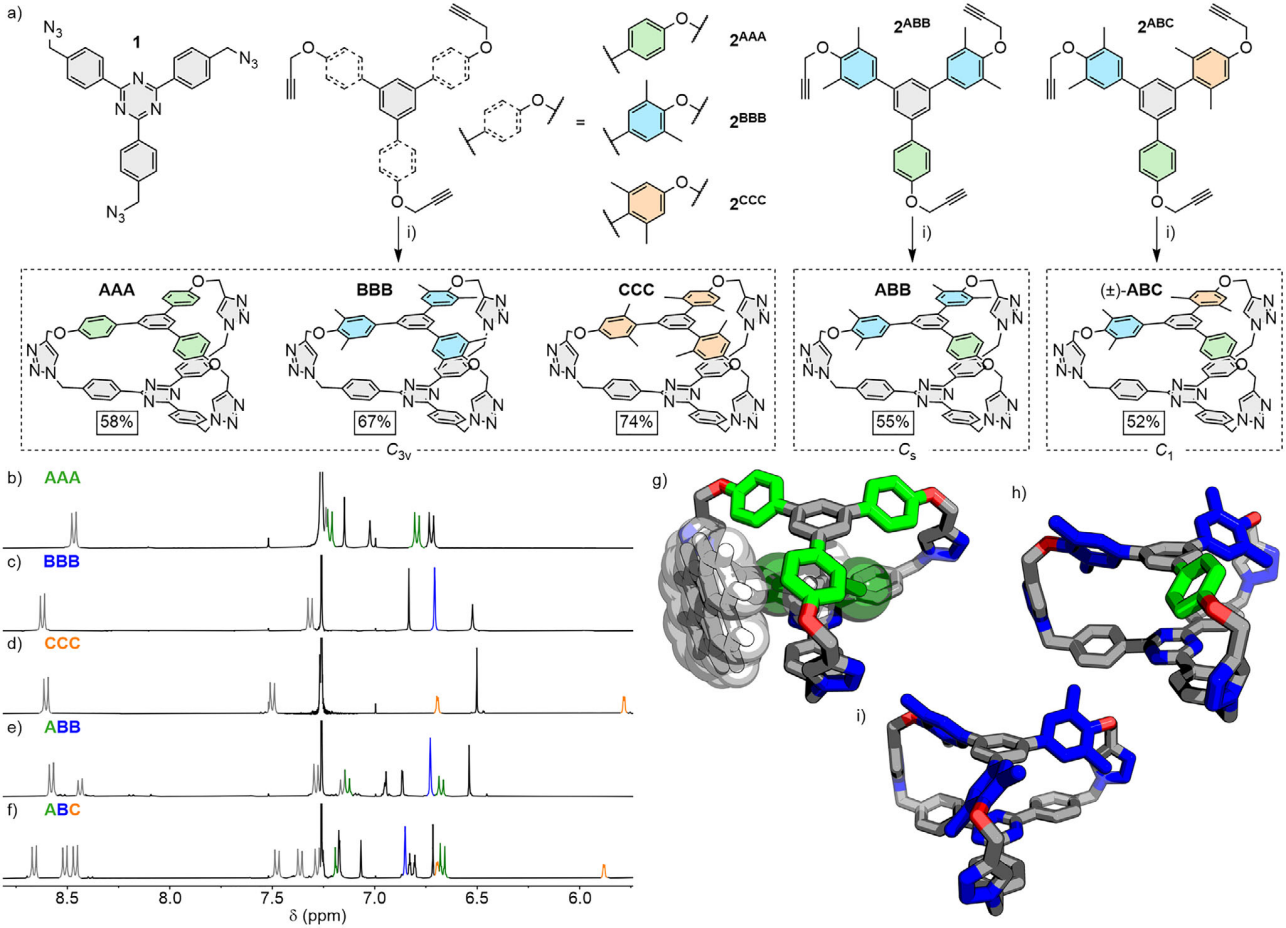
(a) Synthesis of organic cages **AAA**, **BBB**, **CCC**, **ABB** and **ABC**. Reagents and conditions: (i) **1**, CuI, DBU, THF/toluene, 75–110°C. ^1^H NMR spectra (400 MHz, CDCl_3_, 298 K) of (b) **AAA**, (c) **BBB**, (d) **CCC**, (e) **ABB** and (f) **ABC**, with peaks highlighted, belong to arms **A** (green), **B** (blue), **C** (orange) and the phenyl arms of the triazine face (grey). SCXRD structures of (g) **AAA**⊃C_2_H_4_Cl_2_·pyrene, (h) **ABB** and (i) **BBB**.

Organic cages were synthesised from the [1 + 1] CuAAC ‘click’ reaction [55, 56, 57, 58, 59, 60, 61, 62, 63, 64] between tris‐azide building block **1** and a tris‐alkyne (**2**) of tuneable structure and symmetry (Figure 1a) that were readily constructed incorporating one, two or three different *arms*: phenyl (**A**), 3,5‐xylyl (**B**) and 2,6‐xylyl (**C**). Five tris‐alkynes were synthesised with varying symmetry: *D*_3h_ (**2^AAA^**, **2^BBB^** and **2^CCC^**), *C*_2v_ (**2^ABB^**) and *C*_s_ (**2^ABC^**). Reaction of **1** and **2** under pseudo‐high dilution CuAAC conditions produced the cages in good isolated yields of 52%–74%. The identities of all five cages were confirmed by NMR analysis (Figure 1b–f) and high‐resolution electrospray ionisation mass spectrometry (HR‐ESI‐MS). The solid‐state structures of cages **AAA**, **BBB** and **ABB** (Figure 1g–i) were examined by single‐crystal x‐ray diffraction (SCXRD).

For the *C*_1_ (**ABC**) and *C*_s_ (**ABB**) cages, the observation of three and two sets of ^1^H NMR signals for the triaryltriazine face, respectively, reinforced the fact of their reduced symmetry (Figure 1e–f). Interestingly, desymmetrisation of the arms of **CCC** was seen (Figure 1d) due to hindered rotation of the 2,6‐xylyl units, resulting in distinct signals for the *internal* and *external* aromatic (δ = 5.78 and 6.69 ppm, respectively; orange peaks in Figure 1d) and methyl protons (δ = 1.03 and 1.65 ppm, respectively).

Unsurprisingly, given the reduced planarity of the 1,3,5‐triarylphenyl face derived from **2** compared to the triazine face [65], no interactions between the cages and selected aromatic guests (anthracene, pyrene, triphenylene) examined in several solvents (chloroform, THF, acetone, methanol, and combinations of these) were observed. Indeed, a co‐crystal of **AAA** with pyrene located outside the cage was obtained (Figure 1g), with no discernible π–π interactions between the two!

With cages of varying symmetry in hand, the use of these as building blocks for Ag(I)‐based MOFs [66, 67, 68, 69] through coordination of the 1,2,3‐triazole units [70, 71, 72, 73, 74, 75, 76, 77, 78, 79, 80, 81, 82] was examined. While cages **AAA**, **BBB** and **CCC** possess three identical triazole units, **ABB** and **ABC** could act as reduced symmetry tritopic linkers with two or three chemically distinct coordination sites, respectively [83, 84, 85, 86, 87, 88, 89].

Layering a MeOH solution of AgBF_4_ on top of a solution of each cage in either 1:2 DMF/CH_2_Cl_2_ (**BBB** and **ABC**) or 1:2 MeOH/CH_2_Cl_2_ (**CCC** and **ABB**) allowed single crystals of sufficient quality for SCXRD analysis to be obtained for frameworks incorporating cages **BBB**, **CCC**, **ABB** and **ABC** (**BUF‐1**, **BUF‐2**, **BUF‐3** and **BUF‐4**, respectively), all of which had the general formula [Ag**XXX**(BF_4_)]*_n_* (where **XXX** is the cage linker). Attempts to grow x‐ray quality crystals of an Ag(I) framework with **AAA** were unsuccessful. Powder x‐ray diffraction (PXRD) measurements (Figure S121) and optical microscopy (Figure S136) of the solid precipitate obtained from attempts to crystallise this framework indeed suggested the material was amorphous.

Topological analysis of the resultant BUF (*Birmingham University Framework*) structures to determine their underlying net was performed using the ToposPro software [90]. Solvent‐accessible void volumes and surface areas for the cationic frameworks (i.e., excluding anions) were calculated using PLATON [91, 92] (Table 1). Bulk material of the coordination frameworks from each of the five cages was shown by thermogravimetric analysis (TGA) to be stable up to at least 250°C (Figures S126–S130). All of the BUF single crystals examined diffracted weakly. For **BUF‐1**, **‐3** and **‐4**, this necessitated the use of synchrotron radiation at Diamond Light Source to obtain data of suitable quality. For all frameworks, residual electron density associated with disordered solvent molecules was removed using a solvent mask. In the case of **BUF‐1**, BF_4_^−^ counterions were also severely disordered and included in the solvent mask.

**TABLE 1:** Calculated solvent accessible void volumes and surface areas for cationic BUF networks.^a^

Framework	Void volume (%)^b^	Surface area (m^2^ cm^−3^)^c^
**BUF‐1**	40	994
**BUF‐2**	29	576
**BUF‐3**	32	680
**BUF‐4**	43	964

^a^ Volumes and areas calculated from structures with anions and disorder removed.

^b^ Percentage of unit cell volume, calculated using 1.2 Å probe.

^c^ Calculated using 1.84 Å probe.

**FIGURE 2:**
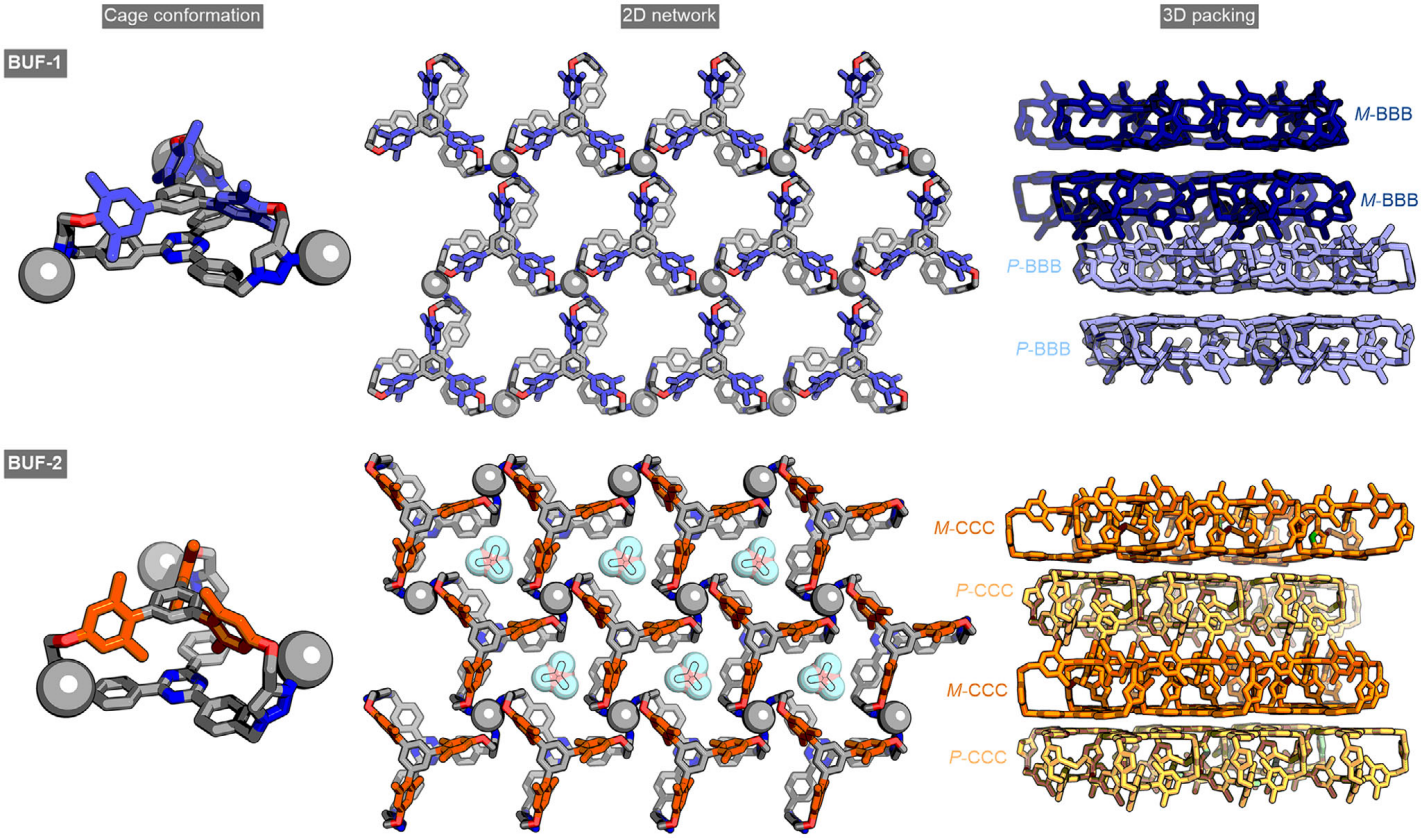
SCXRD structures of **BUF‐1** and **BUF‐2** showing the conformations of the cage linkers (both as *M* conformers), the structures of the 2D coordination networks, and the packing of these networks within the crystals.

**BUF‐1** and **‐2** (from cages **BBB** and **CCC**, respectively) crystallised in space groups $P\bar{1}$ and $R\bar{3}$, respectively, and are structurally the most similar. Each consists of 2D honeycomb‐like sheets of coplanar cages across the plane defined by the crystallographic *a* and *b* axes (**hcb** net; Figure 2). Aside from their aesthetics, such 2D architectures with both in‐plane pores and internal porosity are potentially attractive as high surface area materials [93, 94].

The Ag(I) ions adopt distorted trigonal planar geometries, sitting out of the plane of the coordinating nitrogen atoms by 0.25–0.33 (**BUF‐1**) and 0.92 Å (**BUF‐2**). In the case of **BUF‐2**, BF_4_^−^ counteranions were found within the spaces between the cages. There were significant differences in the packing modes between the two frameworks: smaller distances between Ag(I) ions for **BUF‐2** compared to **BUF‐1** (∼14 and ∼17 Å, respectively), and a greater distance between cage layers in **BUF‐2**, partly from an enhanced effective thickness of **CCC** arising from the increased angle between the arms and phenyl core (Θ_arm_; see below), were observed (Figures S106 and S108). Overall, there is a greater density of cages in **BUF‐2** compared to **BUF‐1** (0.58 and 0.52 cages per nm^3^, respectively) and a lower solvent accessible void space (29% of the unit cell volume for **BUF‐2** compared to 40% for **BUF‐1**).

Within both **BUF‐1** and **BUF‐2**, the cages adopt chiral conformations (assigned, somewhat arbitrarily, as *P* and *M* enantiomers) with the triazole units all oriented in the same direction. While each individual 2D network is composed of homochiral conformers, both frameworks crystallised as racemic mixtures. In **BUF‐2**, the layers pack in an alternating *PMPM*‐fashion, while **BUF‐1** has a *PPMM* ordering (Figure 2). Although there are many similarities between the structures of **BUF‐1** and **‐2**, it is striking the differences that could be effected through simply changing the regiochemistry of the xylyl arms.

**FIGURE 3:**
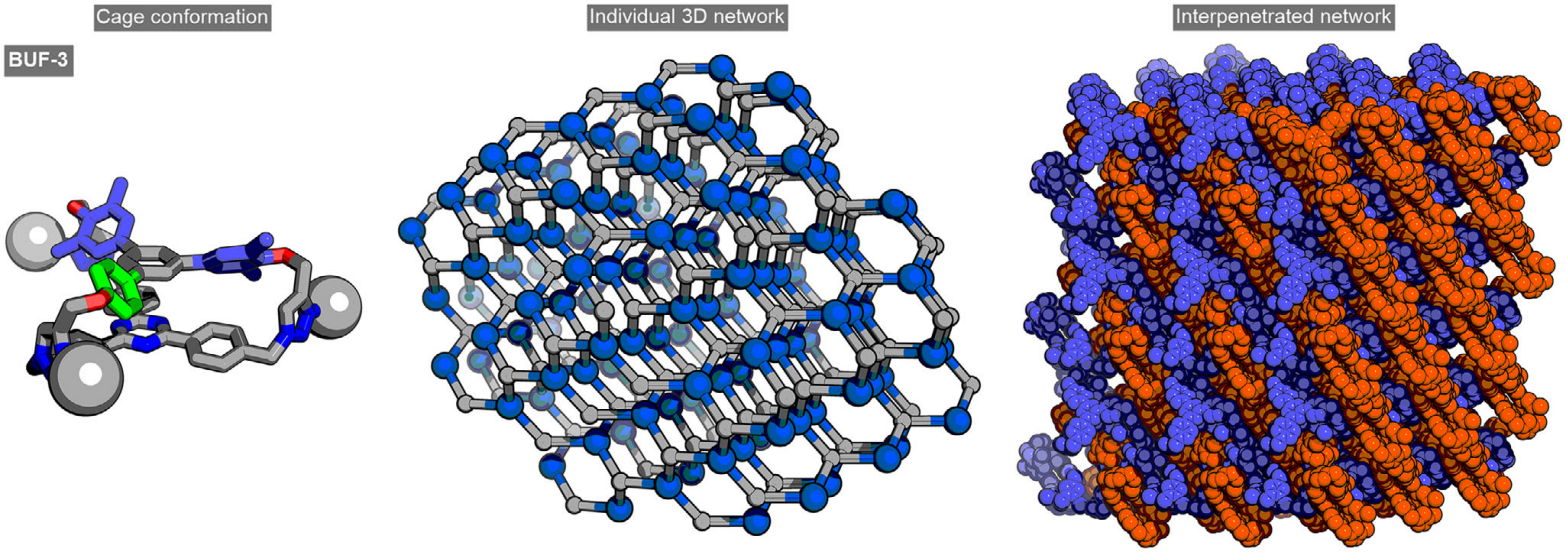
SCXRD structure of **BUF‐3** showing the conformation of the cage linker (disorder omitted), cartoon of the **ths** net of an individual 3D coordination network (blue = linkers, grey = Ag), and structure of the 2‐fold interpenetrated network (individual networks coloured blue and orange; BF_4_^−^ counterions and disorder omitted for clarity).

**FIGURE 4:**
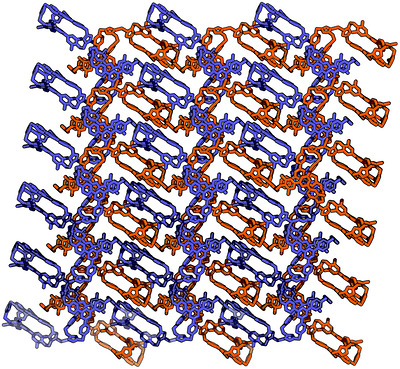
SCXRD structure of **BUF‐3** viewed down the 1D channels created through the pore‐to‐pore arrangement of the cage linkers (individual networks coloured blue and orange; BF_4_^−^ counterions and disorder omitted for clarity).

**BUF‐3** (from **ABB**), which crystallised in the *C2*/*c* space group, formed as a 2‐fold interpenetrated [95, 96] 3D coordination network (**ths** net; Figure 3), retaining the pseudo‐trigonal planar Ag(I) coordination geometry observed for the other systems. The formation of a catenated structure from the relatively bulky cage linker was unexpected, given that introducing steric hindrance is a known design strategy to inhibit network interpenetration [97, 98, 99, 100, 101]. Despite the network interpenetration, the cage linkers align in an unobstructed pore‐to‐pore fashion, resulting in 1D channels throughout the structure defined by the intrinsic pores of the cages (Figure 4).

The **ABB** linker was rotationally disordered across two orientations (Figure S110). With two distinct triazole donors (from arms **A** and **B**), the disorder means there are three possible coordination modes for the Ag(I) ions, with coordination to the triazoles from three **B** arms, two **B** arms and one **A** arm, or one **B** arm and two **A** arms, that statistically would be present in a 2:1:1 ratio.

In contrast to **BUF‐1**, the **ABB** cages in **BUF‐3** form stacked pairs (Figure S111) with close contacts between the phenyl cores and interdigitation of the arms. This is presumably enabled by the single **A** arm that allows interdigitation with minimal steric interactions between the bulky xylyl units, which is not possible with **BBB**. A significantly reduced solvent accessible void volume (32%) and surface area (680 m^2^ cm^−3^) was observed for **BUF‐3** compared to **BUF‐1** (40% and 994 m^2^ cm^−3^, respectively).

The differences in the structures of the coordination frameworks formed by cages **ABB** and **BBB** are dramatic, given that these cages only differ in the replacement of a single xylyl group with a phenyl unit. In the traditional reticular chemistry design of MOFs, inter‐linker interactions are generally minimised, which has allowed multiple different linkers to be incorporated into MOFs without affecting the core structural scaffold [102]. The close‐packing nature of the linkers in these cage‐to‐framework materials, however, allows significant changes to the framework structure to arise from minor linker modifications by exploiting inter‐component supramolecular interactions [103]. This demonstrates the potential for precision molecular engineering to fine‐tune cage linkers in directing the formation of customisable frameworks of varying structures.

**FIGURE 5:**
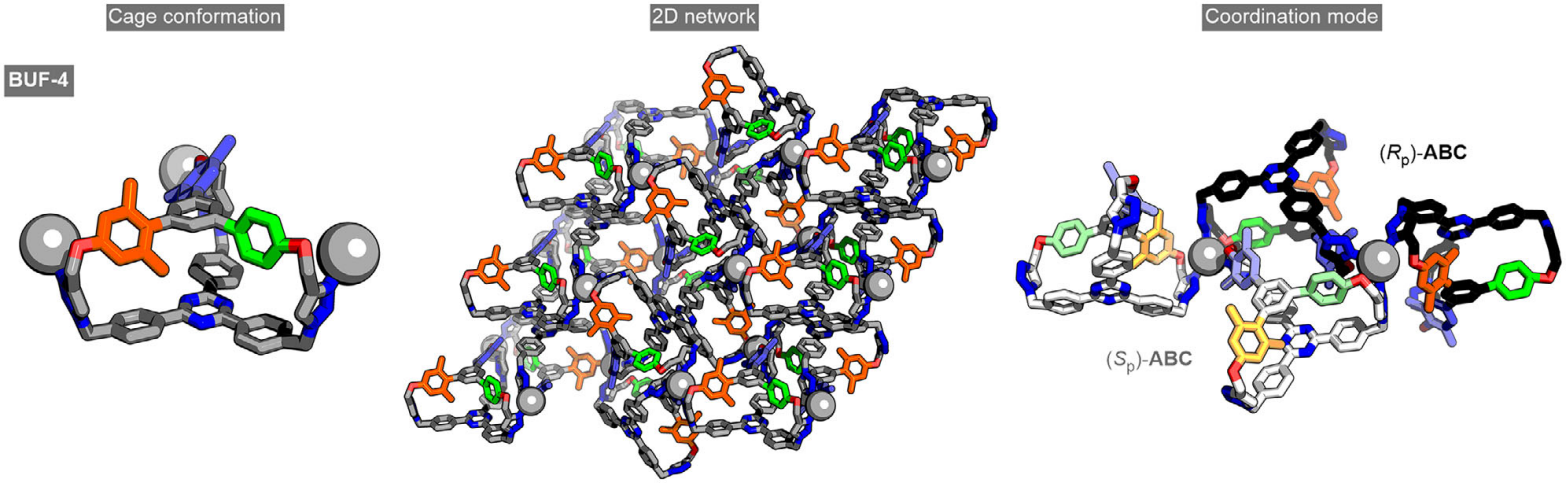
SCXRD structure of **BUF‐4** showing the conformation of the cage linkers, the structures of the 2D coordination networks, and the coordination environment around the Ag(I) ions (only major component of disorder shown).

**BUF‐4** (from **ABC**) crystallised in the monoclinic space group *C*2/*c* and was shown to be a 2D framework. In contrast to **BUF‐1** and **BUF‐2**, the cages within the 2D sheets of the network are not all coplanar, but instead form a corrugated arrangement (**fes** net; Figure 5). Despite the high structural similarity between **ABB** and **ABC**, the cages in **BUF‐4** are prevented from forming interdigitated pairs, like those found in **BUF‐3**, by the single xylyl **C** arm, almost orthogonal to the phenyl core, that inhibits such close contacts. In a further departure from structural motifs common to the other three frameworks, the cage adopts a conformation with the triazole donors not all orientated in the same direction. Attempts to separate the enantiomers of **ABC** by chiral HPLC, with the aim of generating enantiomeric frameworks, were unsuccessful. Efforts to prepare enantiopure cage linkers are ongoing in our lab.

The 2D MOF sheets are composed of Ag_2_ **ABC**_2_ metallocycles, with two Ag(I) ions coordinated between two adjacent cages (Figure 5). The third coordination site of each Ag(I) ion is occupied by the triazole from a third cage that is virtually orthogonal to the other two (∼80° between the planes of the triazine units). As with **BUF‐3** derived from **ABB**, within the crystal structure of **BUF‐4**, the cages were found to be disordered. In this instance, disorder between the **A** and **C** arms represents partial occupancy between the *R* and *S* enantiomers (Figure S113). As such, **BUF‐4** can be considered akin to a multivariate MOF in which linker sites are interchangeable between the enantiomeric cages.

**FIGURE 6:**
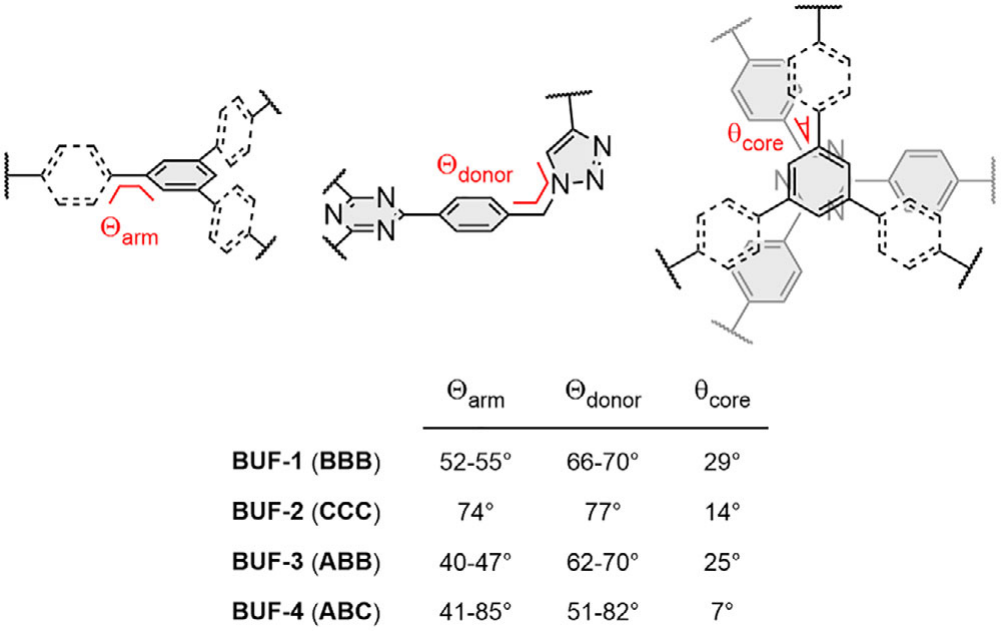
Metrics used for conformational analysis of the cage linkers within the BUF structures.

It is interesting to note the varied conformations adopted by the different cages within the BUFs, despite their structural similarity. Some of these conformational differences are induced by the identities of the arms of the cages, while some are the result of chemically identical arms adopting different conformations. Three parameters (Figure 6) were chosen to exemplify this: (i) the dihedral angles between the cage arms (**A**, **B** or **C**) and the central phenyl spacer (Θ_arm_); (ii) the dihedral angle between the triazole donors and the arms of the triazine face (Θ_donor_); and (iii) the angle of rotation between the 1,3,5‐triarylphenyl and 2,4,6‐triaryl‐1,3,5‐triazine core units of the cage faces (θ_core_).

The potential for significant rotational freedom was demonstrated by the **C** arms in **BUF‐2** and **BUF‐4** having Θ_arm_ values of 74° and 52°–85°, respectively. It is less surprising that the values of Θ_arm_ are lower for **BUF‐1** compared to **BUF‐2** due to the difference in steric clash between the xylyl arms and the central phenyl unit of **BBB** and **CCC**.

The Θ_donor_ values had the greatest range in **BUF‐4** (51°–82°) and more limited variability (62°–77°) between the other three frameworks. Of particular note, the **B** arm in **BUF‐4** had a Θ_donor_ value of 51°, while in **BUF‐1** these were much higher at 66–70°, demonstrating a high degree of conformational flexibility for the triazole units to rotate and change the directionality of the linker coordination vectors.

Finally, the degree of twisting for the cages was shown to vary considerably, with θ_core_ values ranging from 7° for **ABC** in **BUF‐4** up to 29° for **BBB** in **BUF‐1**. All told, this analysis demonstrates both that it is possible to induce conformational changes in the linker scaffold through minor structural changes and that different conformations can be adopted by these semi‐rigid building blocks.

The differences between the MOF structures are significant, given the similarities between the linkers. Indeed, **BUF‐1** and **BUF‐2** are formed from isomeric linkers, as are **BUF‐3** and **BUF‐4**, which are MOFs of different dimensionalities. This behaviour contrasts with established principles of reticular chemistry, where structural isomerism or minor linker modifications typically yield isoreticular frameworks, such as the UiO series of MOFs [104] and their multitude of functionalised analogues. Although at this stage the ability to delineate design principles to purposefully exploit such structure‐directing modifications is limited, the potential ramifications for fine‐tuning such MOF structures are wide‐reaching.

The porosity of the bulk framework materials (obtained after drying under vacuum at 30°C) was subsequently examined to probe the impact of linker structure on functional properties (Table 2). Due to limited quantities of isolated bulk material, CO_2_ adsorption was measured gravimetrically using TGA [105]. It is noted the PXRD patterns of the dried materials did not match those predicted from the SCXRD structures (Figures S122–125); presumably, significant structural changes occurred upon desolvation. The frameworks obtained from cages **CCC** and **ABC** were able to take up more than twice as much CO_2_ as those from **BBB** and **ABC**, demonstrating how small modifications to the linker structure could have significant effects on framework properties. Interestingly, the amorphous framework with **AAA** also exhibited some of the highest CO_2_ uptake values and, post‐sorption analysis, the material had a PXRD pattern (Figure S121) indicative of a degree of structural ordering following gas uptake.

**TABLE 2:** CO_2_ uptake capacity (30°C, 1 atm) of bulk framework materials as determined by TGA.

Cage framework	CO_2_ uptake (cm^3^ g^−1^)
**AAA**	16
**BBB**	6.7
**CCC**	17
**ABB**	16
**ABC**	7.2

## 3 Conclusion

In summary, organic cages of *C*_3_ *_v_* (**BBB**, and **CCC**), *C*_s_ (**ABB**) and *C*_1_ (**ABC**) symmetry were prepared in good isolated yields (up to 74%) and used as linkers in MOF materials through coordination to Ag(I) ions. In this manner, 2D and 3D MOFs were prepared using cages of different structures and symmetries as building blocks to generate customisable porous materials. These represent the first examples of coordination frameworks assembled from reduced symmetry and chiral cage linkers.

Minor modifications to the cage structures, while retaining the core scaffold, resulted in dramatic changes to the resultant MOF architectures, allowing access to frameworks with different topological nets. Compared to prototypical MOF structures, in which porosity is defined by the space between framework components, the use of three‐dimensional building blocks allows close packing and thus interaction between them, enabling control over the framework structures through fine‐tuning of the building blocks. Conformational analysis of the various cages within the MOFs demonstrated their adaptability, which (a) could be adjusted through these minor modifications, and (b) could potentially lead to materials with a degree of flexibility.

This work highlights the potential for the bottom‐up design of sophisticated, customisable framework materials from intrinsically porous building blocks. Precision engineering of the structure, functionality and symmetry of cage building blocks allows fine‐tuning of intrinsic porosity and MOF topology, and thus the functional properties of the materials. Increasing our understanding of both the design of such linkers and how to control their assembly will enable the targeted design of new, bespoke, functional porous materials.

## Author Contributions

**Cameron J. T. Cox**: synthesis, characterisation, data collection and analysis. **Aaron H. Bernardino**: characterisation and data analysis. **Louise Male**: data collection and analysis. **Rebecca L. Greenaway**: supervision and data analysis. **Georgia R. F. Orton**: data analysis, writing–manuscript original draft. **James E. M. Lewis**: project conception and direction, supervision, funding acquisition, writing–manuscript original draft. All authors contributed to editing and approved the final manuscript.

## Conflicts of Interest

The authors declare no conflict of interest.

## Supporting information




**Supporting File 1**: anie72582‐sup‐0001‐SuppMat.pdf.

**Supporting File 2**: anie72582‐sup‐0002‐cif.zip.

## Data Availability

The data that supports the findings of this study are available in the supplementary material this article.
