# Supplementary material for: Reduced Symmetry Metal–Organic Cage‐to‐Framework Materials
Source: Angew Chem Int Ed Engl. 2026 May 14;65(26):e8127392. doi: 10.1002/anie.8127392 (PMC13285475; doi:10.1002/anie.8127392)
Supplement: Supplementary file 1 — Supporting File 1: anie72582‐sup‐0001‐SuppMat.pdf. [file ANIE-65-e8127392-s001.pdf]

Electronic Supporting Information

Reduced Symmetry Metal-Organic Cage-to-Framework Materials

C. J. T. Cox,[a] A. H. Bernardino,[b] L. Male,[a] R. L. Greenaway,[b] G. R. F. Orton[a] and J. E. M. Lewis[a]*

[a]School of Chemistry, University of Birmingham

Edgbaston, Birmingham B15 2TT, United Kingdom

*j.e.m.lewis@bham.ac.uk

[b]Department of Chemistry, Imperial College London

Molecular Sciences Research Hub, White City Campus, London W12 0BZ, UK

Contents

S1. General Experimental 4
S2. Synthetic Procedures 6
Synthesis of **5**^{AAA} 7
Synthesis of **S1**^{AAA} 9
Synthesis of **2**^{AAA} 10
Synthesis of **5**^{BBB} 14
Synthesis of **S1**^{BBB} 18
Synthesis of **2**^{BBB} 19
Synthesis of **5**^{CCC} 23
Synthesis of **S1**^{CCC} 27
Synthesis of **2**^{CCC} 28
Synthesis of **5**^{A} 32
Synthesis of **5**^{ABB} 36
Synthesis of **S1**^{ABB} 40
Synthesis of **2**^{ABB} 41
Synthesis of **5**^{AB} 45
Synthesis of **5**^{ABC} 49
Synthesis of **S1**^{ABC} 53
Synthesis of **2**^{ABC} 54
Synthesis of **1** 58
Synthesis of cage **AAA** 60
Synthesis of cage **BBB** 64
Synthesis of cage **CCC** 68
Synthesis of cage **ABB** 72
Synthesis of cage **ABC** 76
S3. Single crystal X-ray diffraction 80
Sample Preparation 80
Data Collection and Analysis 80
AAA 80
ABB 81
BBB 81
BUF-1 81
BUF-2 82
BUF-3 83
BUF-4 (Dataset #1) 85
BUF-4 (Dataset #2) 86

S4. Powder X-ray diffraction 87
S5. Thermogravimetric Analysis 93
S6. CO_2 Uptake Profiles 96
S7. Optical Microscopy Images.. 100
S8. References 102

S1. General Experimental

Synthesis: Unless otherwise stated, all reagents, including anhydrous solvents, were purchased from commercial sources and used without further purification. $CDCl_3$ was stored over 4 Å molecular sieves prior to use. All reactions were carried out under an atmosphere of N_2 using degassed, anhydrous solvents unless otherwise stated. Analytical TLC was performed on pre-coated silica gel plates (0.25 mm thick, 60F254, Merck, Germany) and observed under UV light.

Analysis: NMR spectra were recorded on Bruker 400 MHz or 600 MHz instrument, at a constant temperature of 298 K. Chemical shifts are reported in parts per million from low to high field and referenced to residual solvent ($CDCl_3$: ^1H δ 7.26 ppm, ^{13}C δ 77.16 ppm; CD_3OD: ^1H δ 3.31 ppm). Standard abbreviations indicating multiplicity were used as follows: m = multiplet, quint = quintet, q = quartet, t = triplet, d = doublet, s = singlet, app. = apparent, br. = broad. Signal assignment was carried out using 2D NMR methods (HSQC, HMBC, COSY, NOESY) where necessary. In the case of some signals absolute assignment was not possible. Here indicative either/or assignments (e.g. H_a/H_b for H_a or H_b) are provided. Mass spectrometry was carried out using a Waters Xevo-G2-XS.

Thermogravimetric Analysis: TGA was carried out using a Waters TGA550-1655 Discovery (TA instruments) with an automated vertical overhead thermobalance. Decomposition measurements were performed with samples heated in platinum pans under N_2 at a rate of 10 ºC/min to 600 °C. CO_2 adsorption measurements used the following program: the samples were heated in platinum pans under N_2 at a rate of 20 °C/min to 90 °C, held at this temperature for 40 minutes, and subsequently cooled to 30 °C at a rate of 5 °C/min, followed by an isothermal hold for 30 minutes. Finally, the samples were purged with CO_2 (1 bar) for 30 minutes at a flow rate of 95 mL/min for the sample gas and 5 mL/min for the balance gas. CO_2 uptake measurements were carried out only on the coordination framework samples, with two replicates performed for each sample. The average values and corresponding standard deviations were calculated (Table S1), while plots corresponding to a single experiment per sample are also presented (Figs S124-128). Prior to TGA and gravimetric CO_2 uptake measurements, samples were activated overnight in a vacuum oven at 90 °C.

Powder X-ray Diffraction: PXRD measurements were conducted in Bragg–Brentano geometry using a Malvern Panalytical Empyrean X-ray diffractometer equipped with a Cu-anode X-ray source operated at 40 kV and 40 mA. The diffractometer was fitted with iCore and dCore optical modules for the incident and diffracted beams, respectively, a rotational sample stage with an anti-scattering knife, and a Pixel3D detector. Data were collected over a 2θ range of 4°–40°, with a step size of 0.02626°. For post-activation measurements, samples were activated overnight in a vacuum oven at 90 °C. Post-sorption measurements were performed on the samples following CO_2 uptake experiments.

Optical Microscopy/Polarised Optical Microscopy: OM/POM images of cages and coordination frameworks were acquired using an Olympus BX53M Upright Microscope with 10×, 20× and 50× magnification, and a polarising lense only for POM images. Images were captured with EPview software using an Olympus EP50 stand-alone network camera and

analysed using ImageJ software to display the scale bar. Each sample was uniformly dispersed in a drop of soybean oil on a microscope slide. Clear glass microscope slides (0.8–1.0 mm thickness) were used. All polarised optical micrographs are taken at the same relative angle to the cross polarisers.

S2. Synthetic Procedures

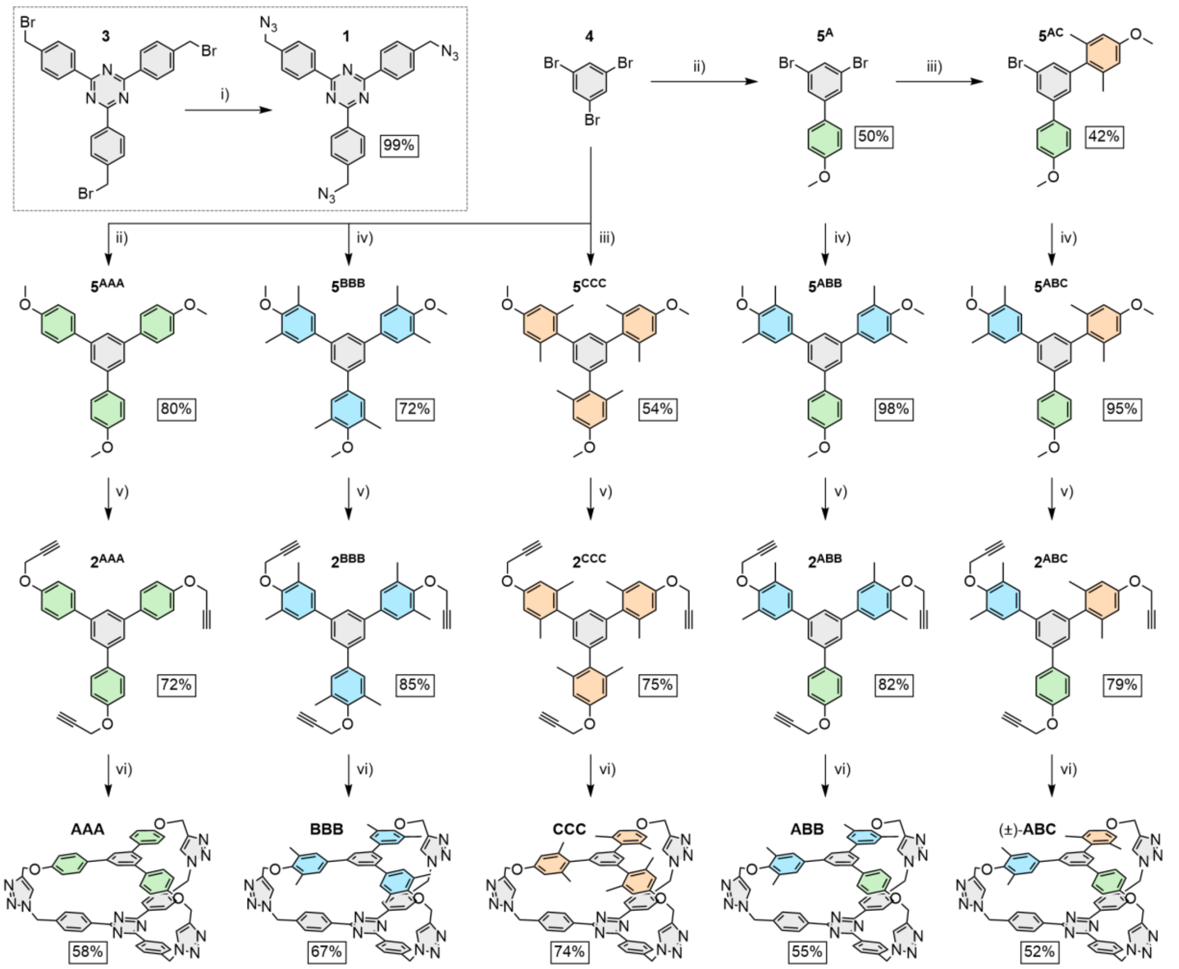

Scheme S1 Synthesis of organic cages **AAA**, **BBB**, **CCC**, **ABB**, and **ABC**. Reagents and conditions: i) NaN_3, DMF, rt; ii) 4-methoxybenzeneboronic acid, $Pd(PPh_3)_2Cl_2$, K_2CO_3, 3:1 v/v dioxane/H_2O, 110 °C; iii) 2,6-dimethyl-4-methoxybenzeneboronic acid, $Pd(PPh_3)_2Cl_2$, K_2CO_3, 3:1 v/v dioxane/H_2O, 110 °C; iv) 3,5-dimethyl-4-methoxybenzeneboronic acid, $Pd(PPh_3)_2Cl_2$, K_2CO_3, 3:1 v/v dioxane/H_2O, 110 °C; v) Step 1: AcOH, 48% $HBr_{(aq)}$, 110 °C; Step 2: 80% propargyl bromide in toluene, K_2CO_3, MeCN, 80 °C; vi) CuI, DBU, THF/toluene, 75-110 °C.

Synthesis of 5^{AAA}

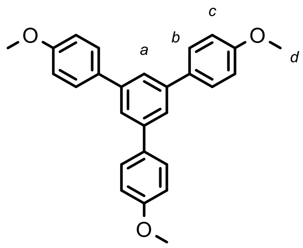

1,3,5-Tribromobenzene (0.315 g, 1.0 mmol, 1 eq.), 4-methoxybenzene boronic acid (0.502 g, 3.3 mmol, 3.3 eq.), $Pd(PPh_3)_2Cl_2$ (0.052 g, 0.075 mmol, 7.5 mol%), and K_2CO_3 (0.104 g, 7.5 mmol, 7.5 eq.) were stirred at 110 °C in 1:3 H_2O/dioxane (degassed, 12 mL) for 30 h. To the cooled reaction mixture was added H_2O (20 mL) and the aqueous phase extracted with CH_2Cl_2 (3 × 20 mL). The combined organic phases were washed with brine (2 × 30 mL), dried ($MgSO_4$) and the solvent removed *in vacuo*. After purification by column chromatography on silica gel (3:2 hexane/CH_2Cl_2) the product was obtained as a white solid (0.314 g, 80%).

^{1}H NMR (400 MHz, $CDCl_3$, 298 K) δ: 7.66(s, 3H, H_a), 7.63 (d, J = 8.7 Hz, 6H, H_b), 7.02 (d, J = 8.7 Hz, 6H, H_c), 3.87 (s, 9H, H_d).

^{13}C NMR (101 MHz, $CDCl_3$, 298 K) δ: 159.4, 142.0, 134.0, 128.5, 124.0, 114.4, 55.5.

HR-ESI-MS m/z = 397.1821 $[M+H]^+$ calc. 397.1804.

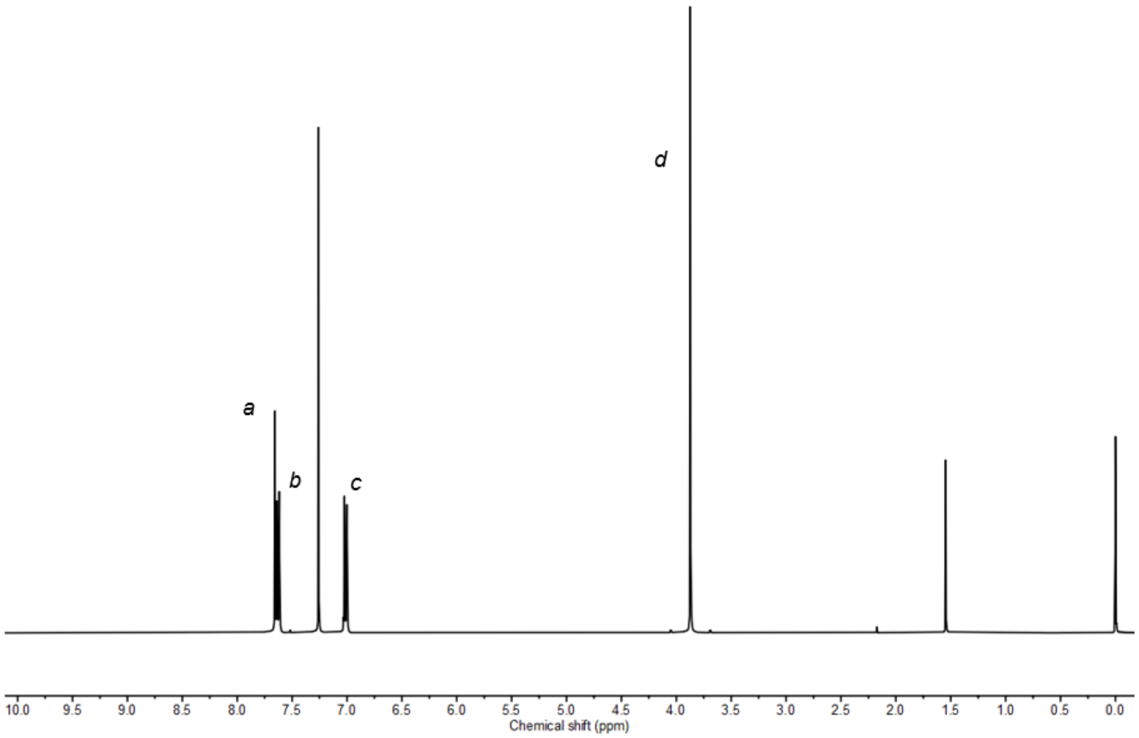

Figure S1 ^{1}H NMR of 5^{AAA} (400 MHz, $CDCl_3$)

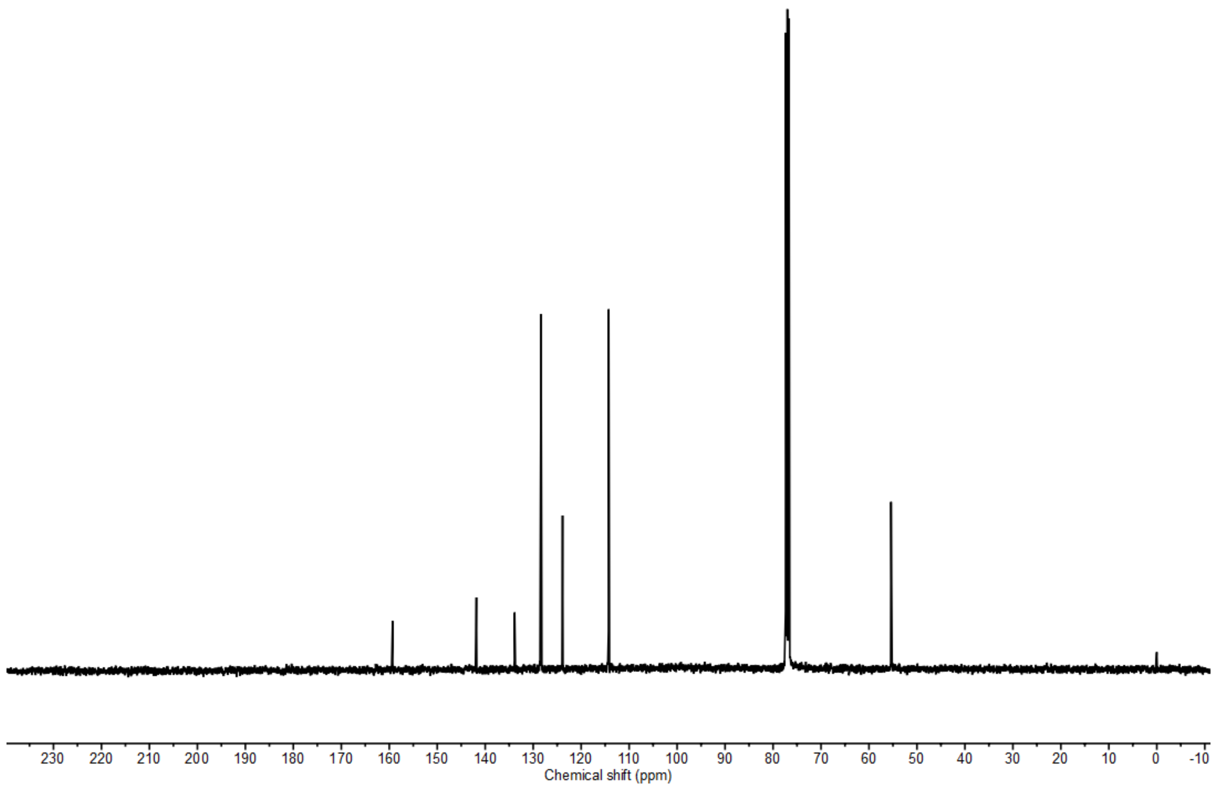

Figure S2 ^{13}C NMR of 5^{AAA} (101 MHz, $CDCl_3$)

Synthesis of S1^{AAA}

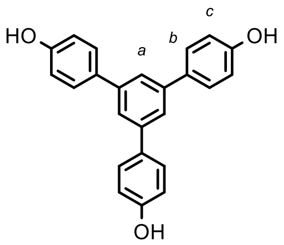

A solution of **5^{AAA}** (0.198 g, 0.5 mmol, 1 eq.) in acetic acid (5 mL) was heated at 110 °C in a reflux apparatus connected to a Dreschel flask containing sat. aq. $NaHCO_3$. 48% $HBr_{(aq)}$ (0.51 mL, 4.5 mmol, 9 eq.) was added dropwise and the reaction stirred for 24 h. To the cooled reaction mixture was added ice water (15 mL). The resultant white precipitate was collected by filtration, washed with H_2O (3 × 10 mL) and dried in air. The off-white solid (0.278 g) was taken forward without further purification.

^{1}H NMR (400 MHz, CD_3OD, 298 K) δ: 7.61 (s, 3H, H$_a$), 7.57 (d, *J* = 8.8 Hz, 6H, H$_b$), 6.91 (d, *J* = 8.8 Hz, 6H, H$_c$).

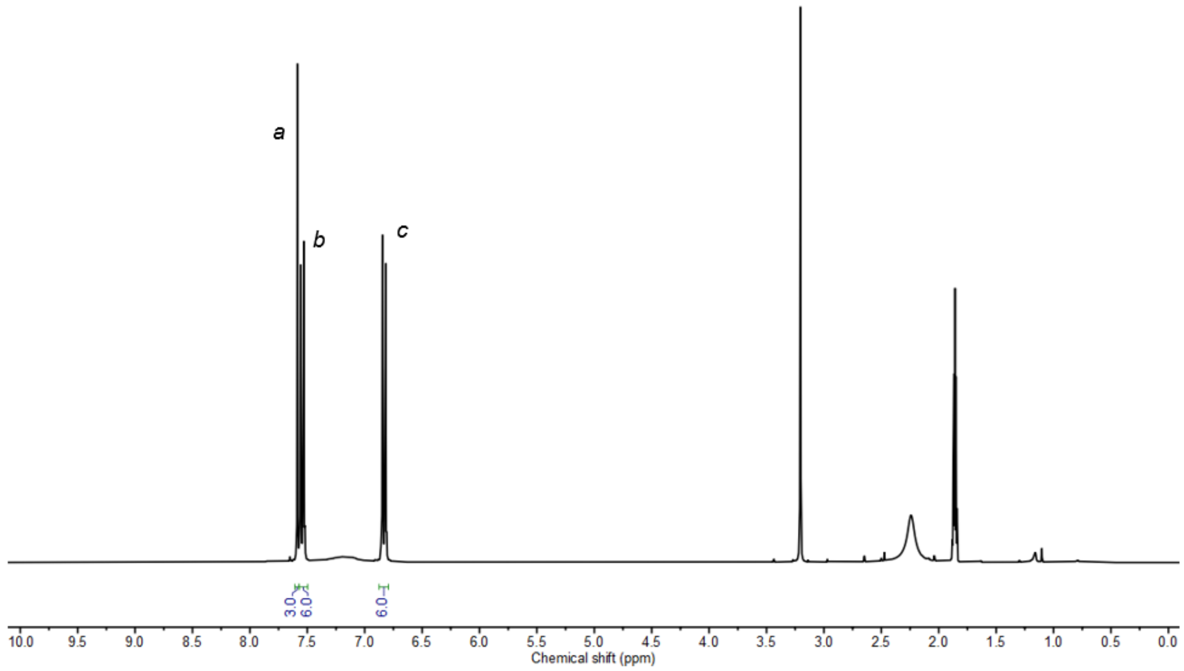

Figure S3 ^{1}H NMR of S1^{AAA} (400 MHz, CD_3OD)

Synthesis of 2^{AAA}

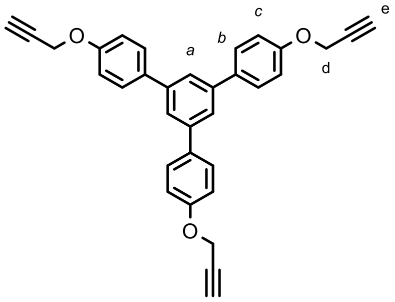

$S1^{AAA}$ (0.142 g) and K_2CO_3 (0.839 g, 6.0 mmol, 15 eq.) were suspended in degassed acetone (15 mL) and stirred at 65 °C. Propargyl bromide solution (80% in toluene, 0.121 mL, 1.6 mmol, 4 eq.) was added to this suspension and allowed to react for 16 h. To the cooled reaction mixture H_2O (30 mL) was added and aqueous phase extracted with CH_2Cl_2 (3 × 30 mL). The combined organic phases were washed with brine (2 × 30 mL), dried ($MgSO_4$) and the solvent removed *in vacuo*. After purification by column chromatography on silica gel (3:2 hexane/CH_2Cl_2) the product was obtained as a white solid (0.120 g, 72% over 2 steps).

^{1}H NMR (400 MHz, $CDCl_3$, 298 K) δ: 7.66 (s, 3H, H_a), 7.63 (d, J = 8.8 Hz, 6H, H_b), 7.09 (d, J = 8.8 Hz, 6H, H_c), 4.76 (d, J = 2.4 Hz, 6H, H_d), 2.56 (t, J = 2.4 Hz, 3H, H_e).

^{13}C NMR (101 MHz, $CDCl_3$, 298 K) δ: 157.3, 141.7, 134.7, 128.4, 124.1, 115.3, 78.5, 75.7, 55.9.

HR-ESI-MS m/z = 469.1816 $[M+H]^+$ calc. 469.1804.

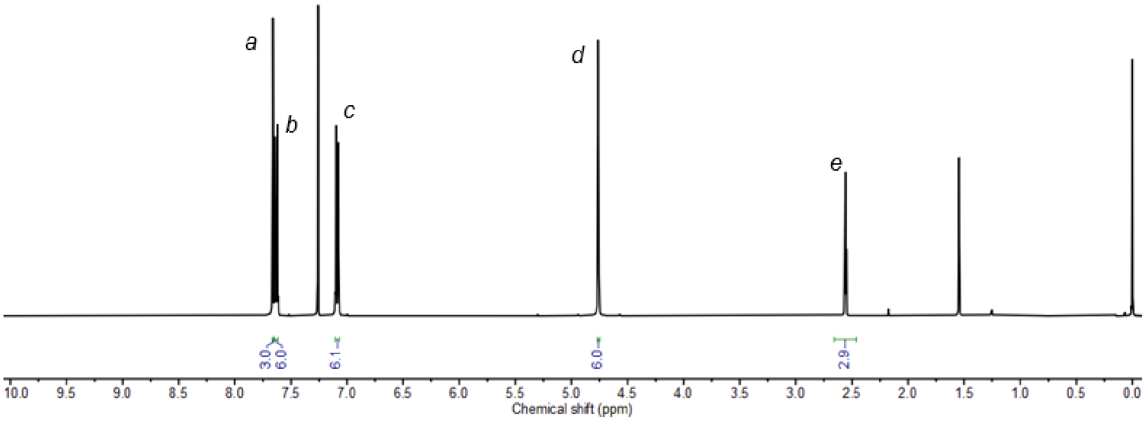

Figure S4 ^{1}H NMR of 2^{AAA} (400 MHz, $CDCl_3$)

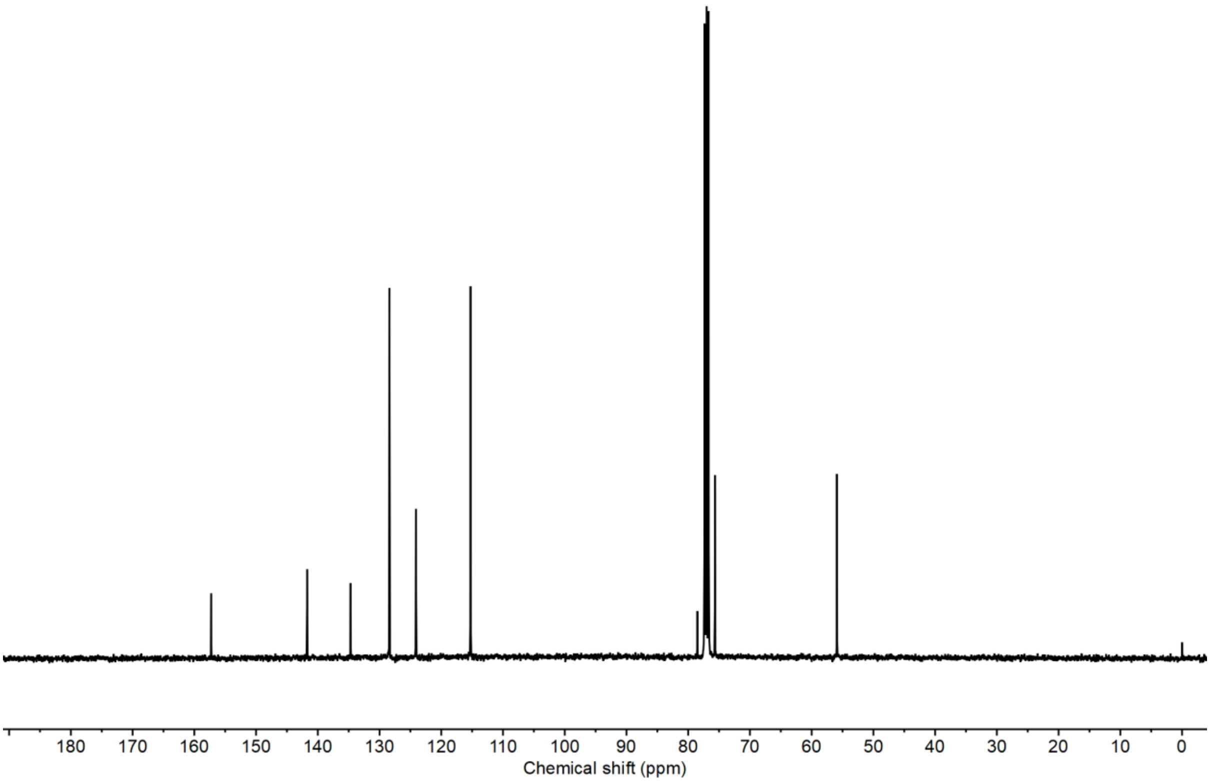

Figure S5 ^{13}C NMR of 2^{AAA} (101 MHz, $CDCl_3$)

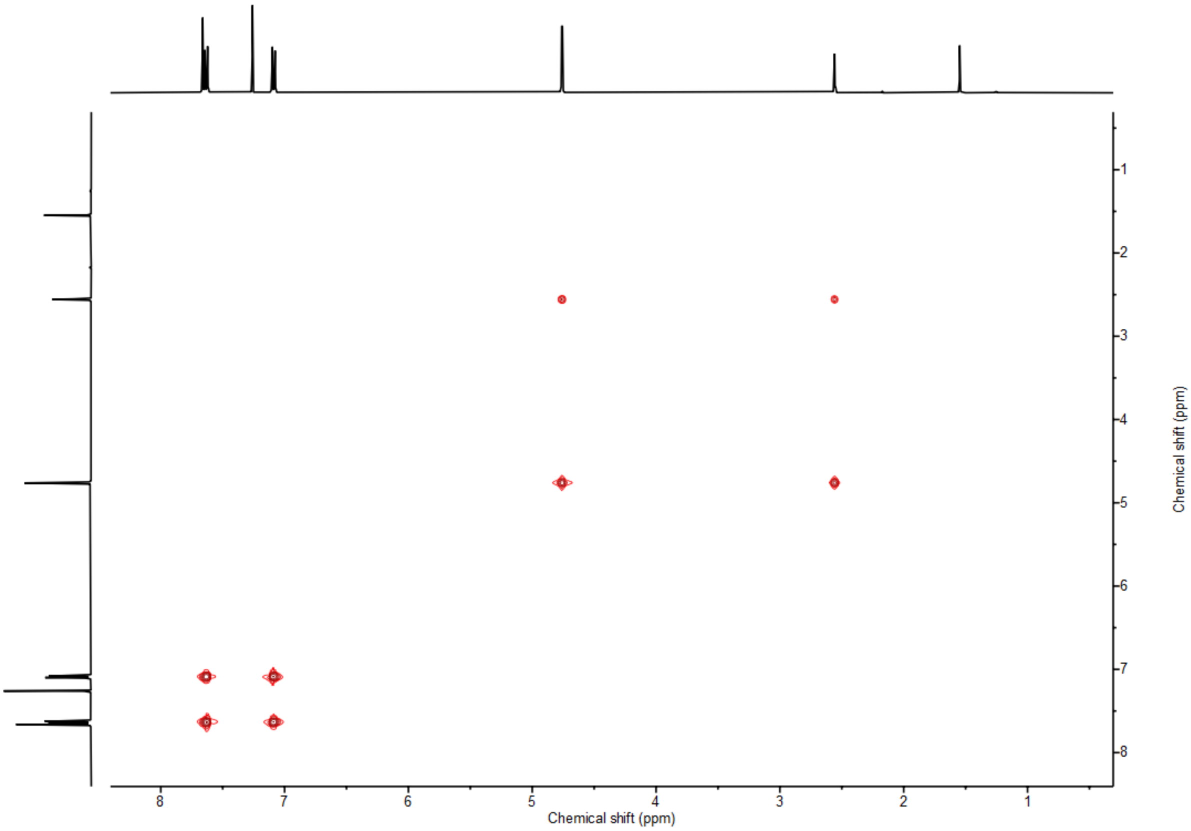

Figure S6 COSY NMR of 2^{AAA} (400 MHz, $CDCl_3$)

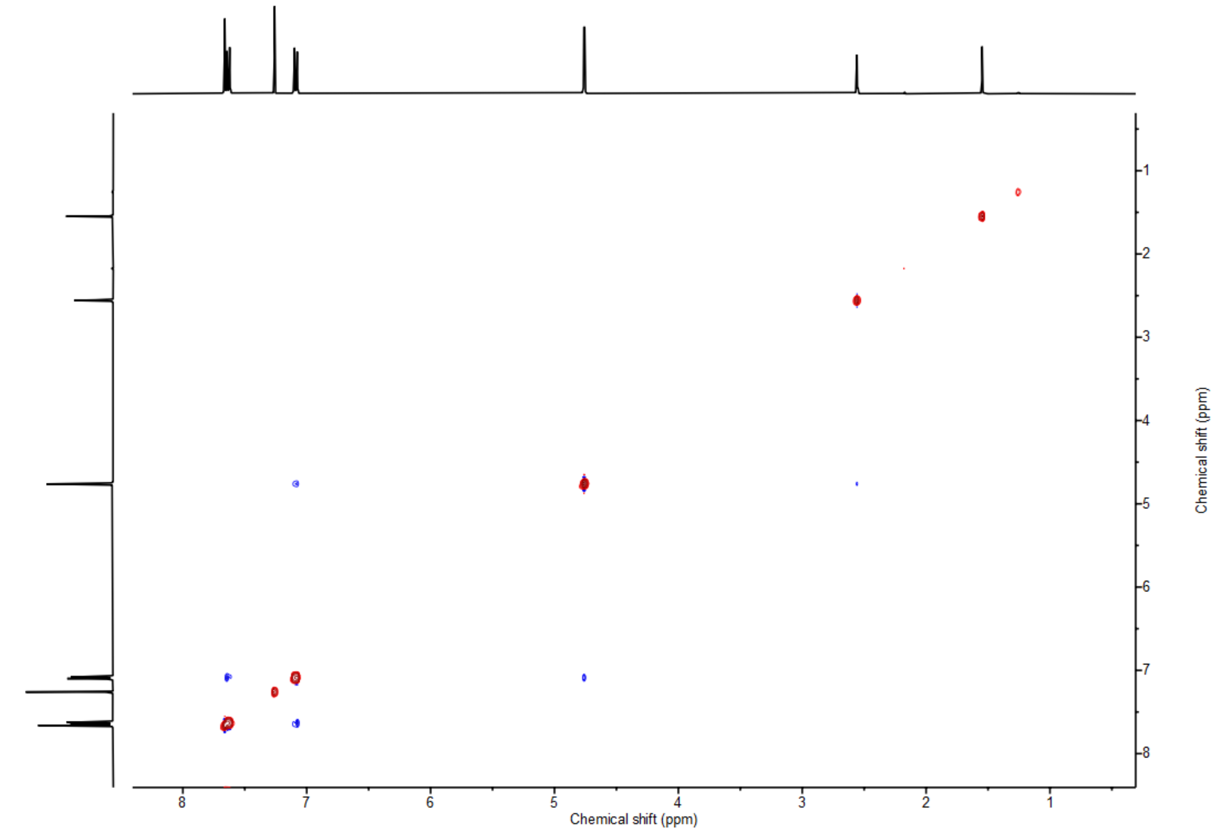

Figure S7 ^{1}H NOESY NMR of 2^{AAA} (400 MHz, CDCl$_3$)

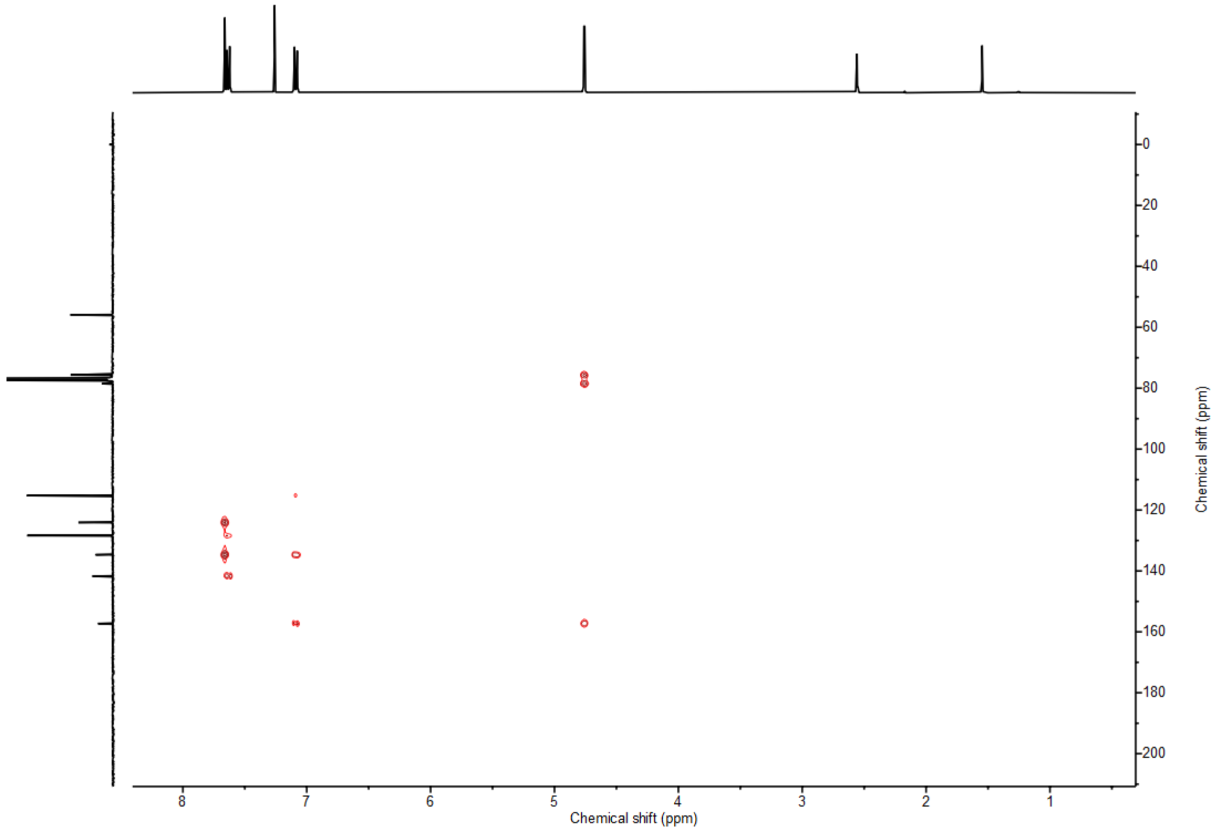

Figure S8 HMBC NMR of 2^{AAA} (CDCl$_3$)

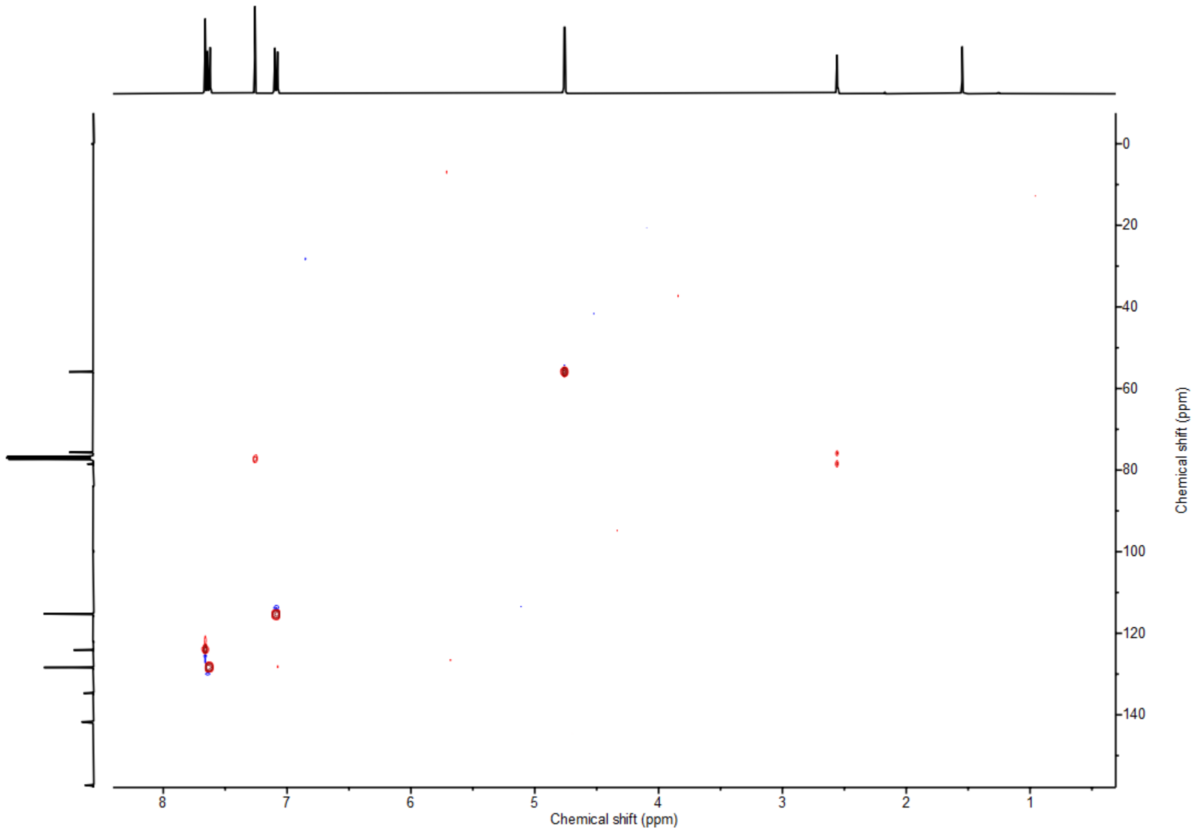

Figure S9 HSQC NMR of 2[AAA] (CDCl$_3$)

Synthesis of 5^{BBB}

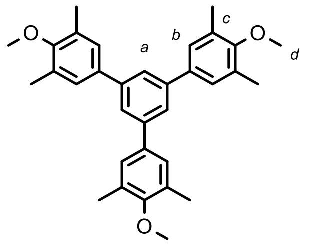

1,3,5-Tribromobenzene (0.315 g, 1.0 mmol, 1 eq.), 4-methoxy-3,5-dimethylphenylboronic acid (0.720 g, 4.0 mmol, 4.0 eq.), $Pd(PPh_3)_2Cl_2$ (0.052 g, 0.075 mmol, 7.5 mol%), and K_2CO_3 (1.05 g, 7.5 mmol, 7.5 eq.) were stirred at 110 °C in 1:3 H_2O/dioxane (degassed, 7.5 mL) for 30 h. To the cooled reaction mixture was added H_2O (20 mL) and the aqueous phase extracted with CH_2Cl_2 (3 × 20 mL). The combined organic phases were washed with brine (2 × 30 mL), dried ($MgSO_4$) and the solvent removed *in vacuo*. After purification by column chromatography on silica gel (9:1 hexane/CH_2Cl_2) the product was obtained as a white solid (0.342 g, 72%).

^{1}H NMR (400 MHz, $CDCl_3$, 298 K) δ: 7.63 (s, 3H, H_a), 7.33 (s, 6H, H_b), 3.78 (s, 9H, H_d), 2.38 (s, 18H, H_c).

^{13}C NMR (101 MHz, $CDCl_3$, 298 K) δ: 156.7, 141.9, 136.9, 131.2, 127.8, 124.5, 59.8, 16.3.

HR-ESI-MS *m/z* = 481.2696 $[M+H]^+$ calc. 481.2743.

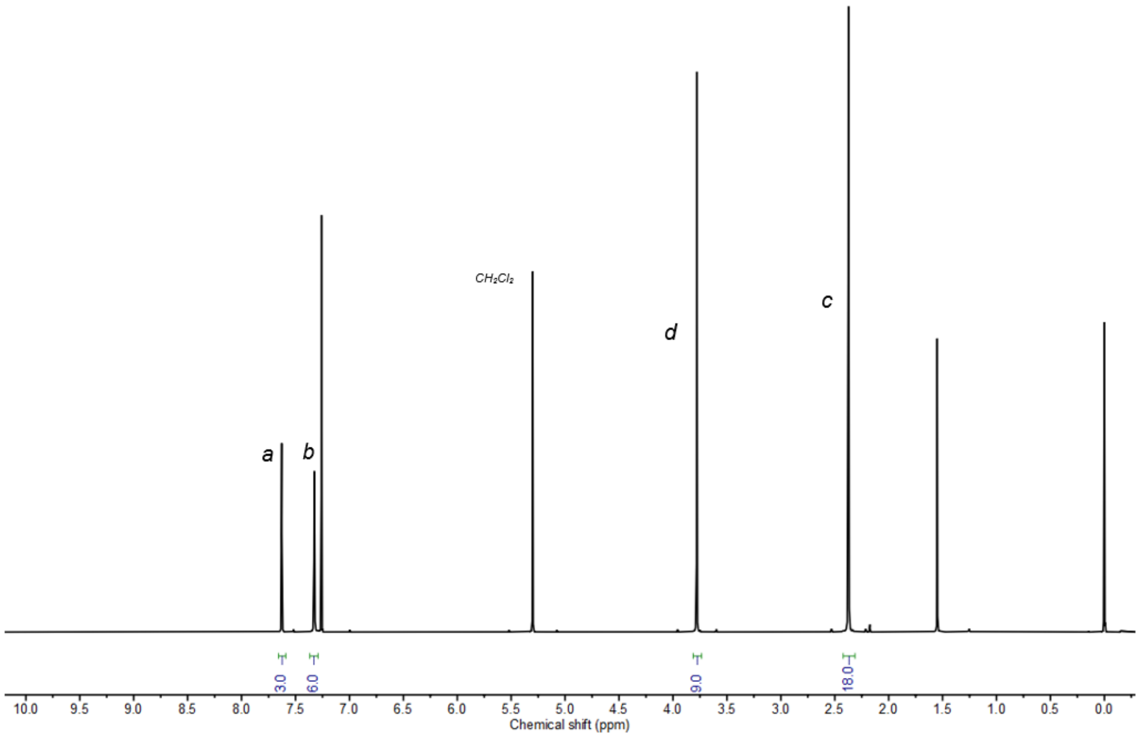

Figure S10 ^{1}H NMR of 5^{BBB} (400 MHz, $CDCl_3$)

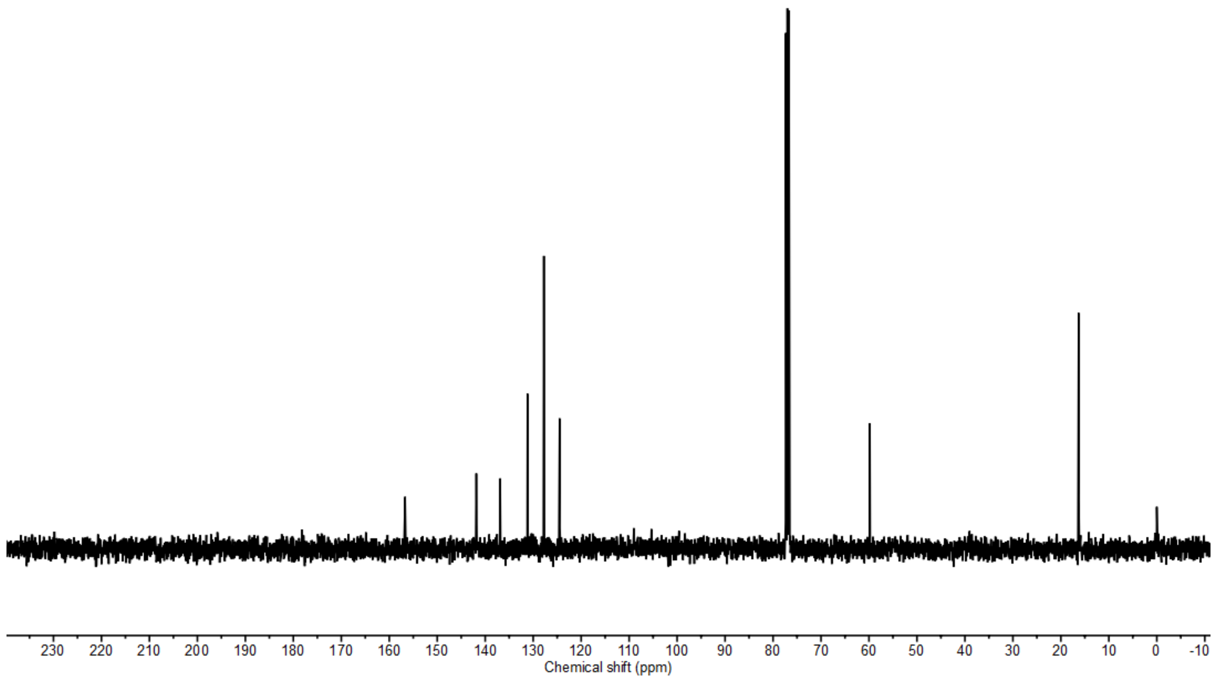

Figure S11 ^{13}C NMR of 5^{BBB} (101 MHz, $CDCl_3$)

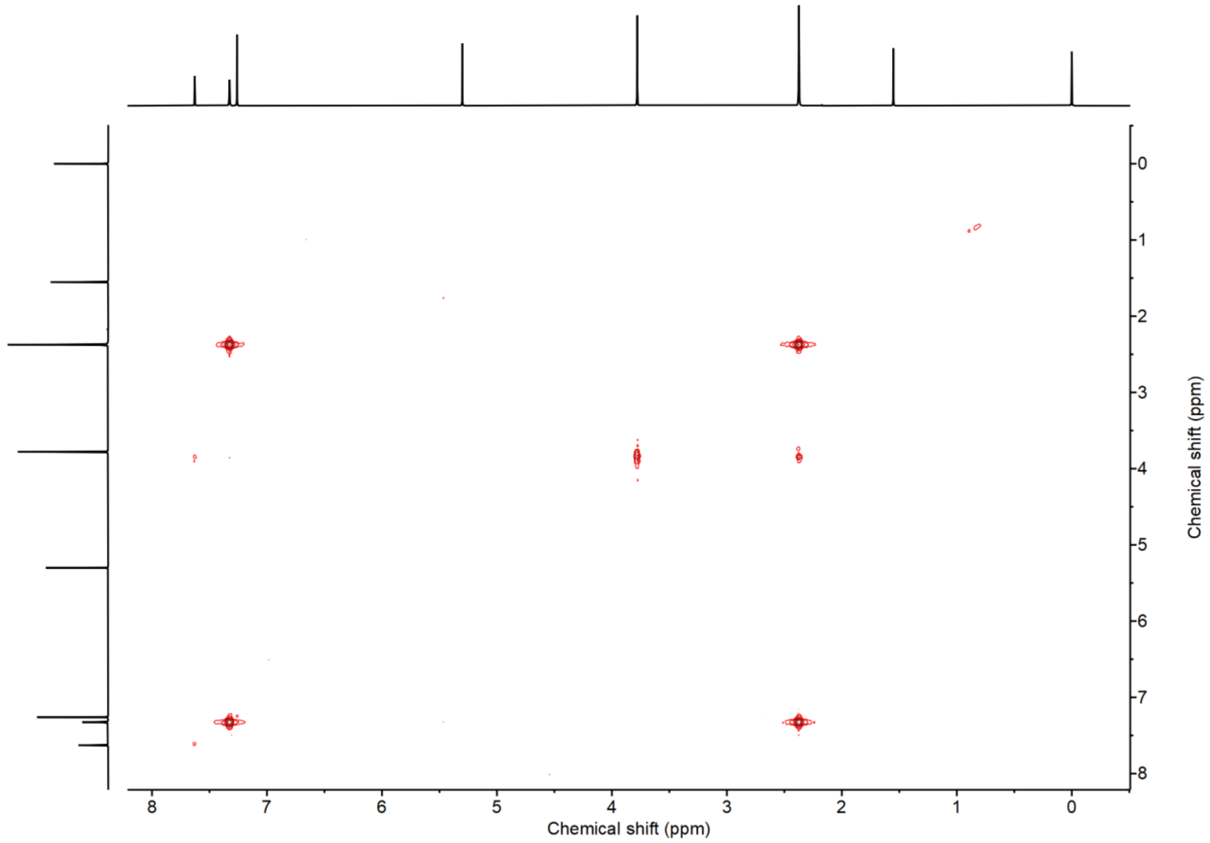

Figure S12 COSY NMR of 5^{BBB} ($CDCl_3$)

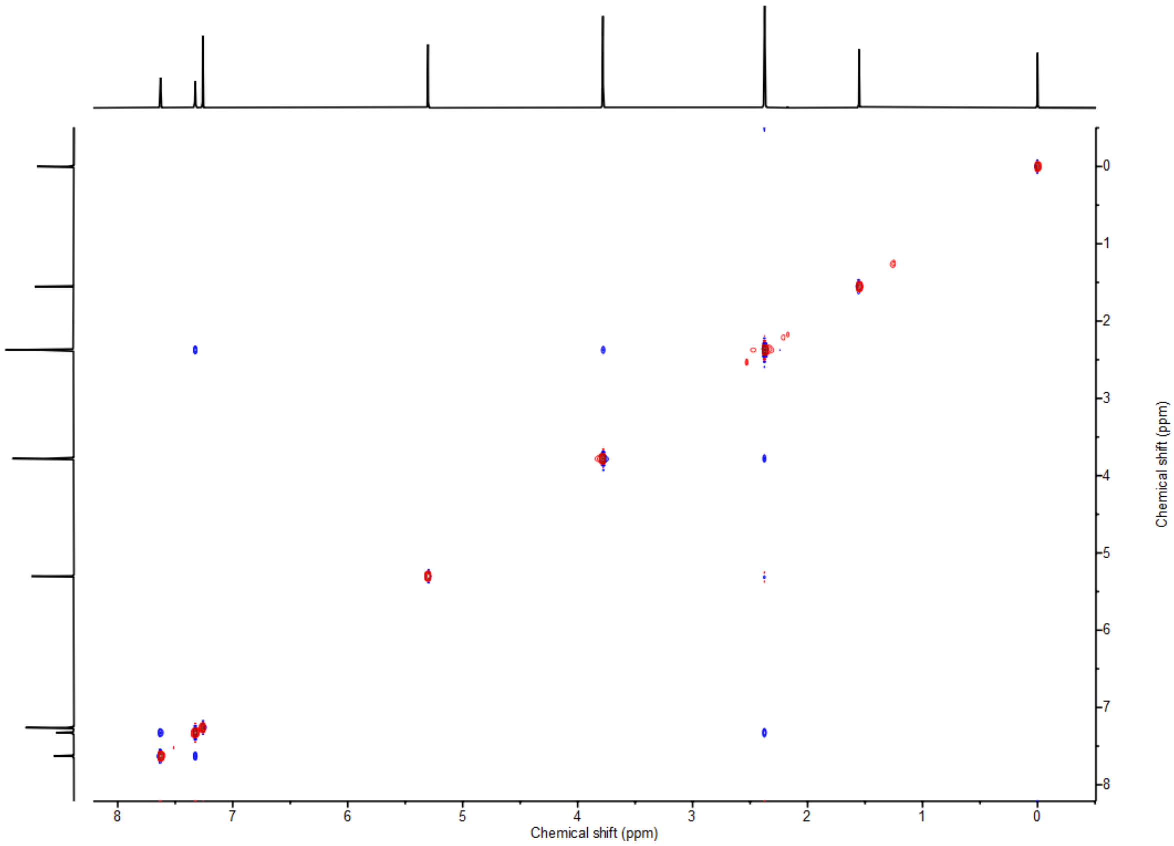

Figure S13 NOESY NMR of 5^{BBB} ($CDCl_3$)

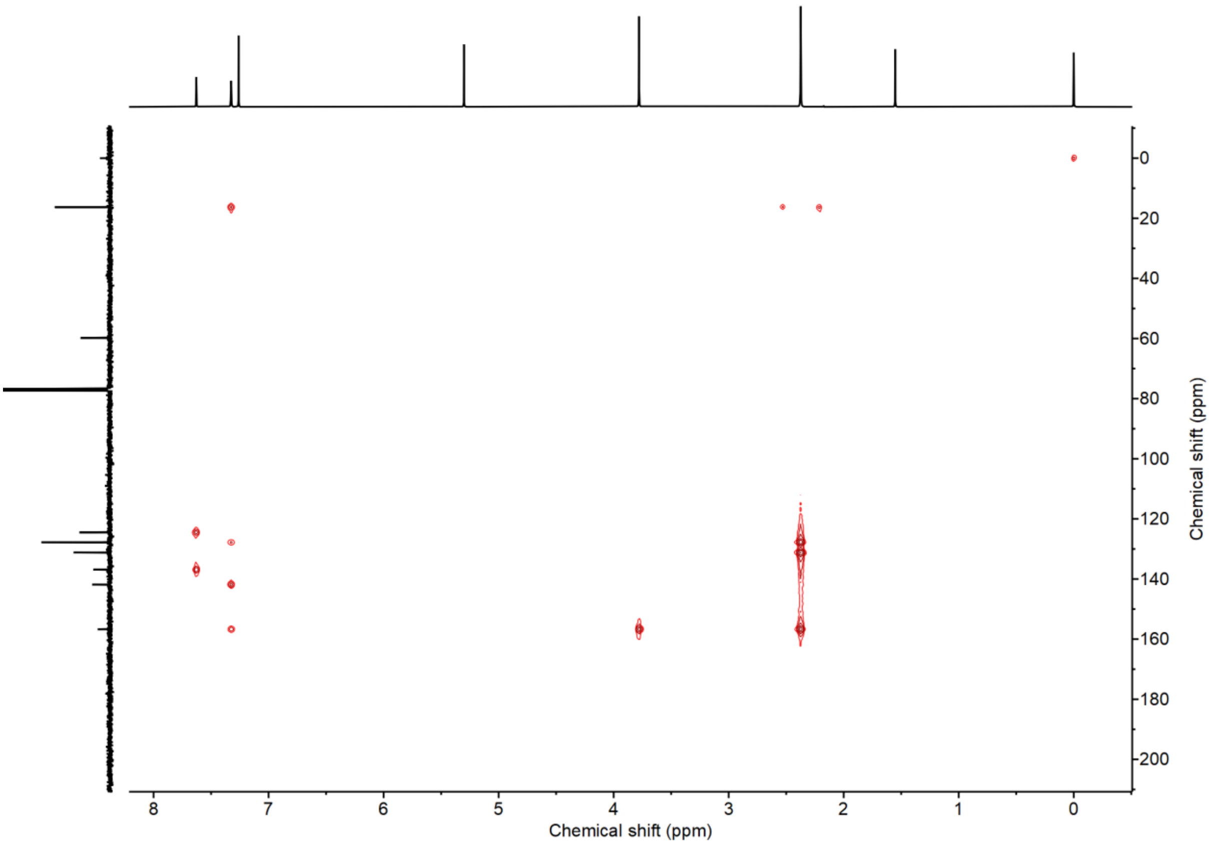

Figure S14 HMBC NMR of 5^{BBB} ($CDCl_3$)

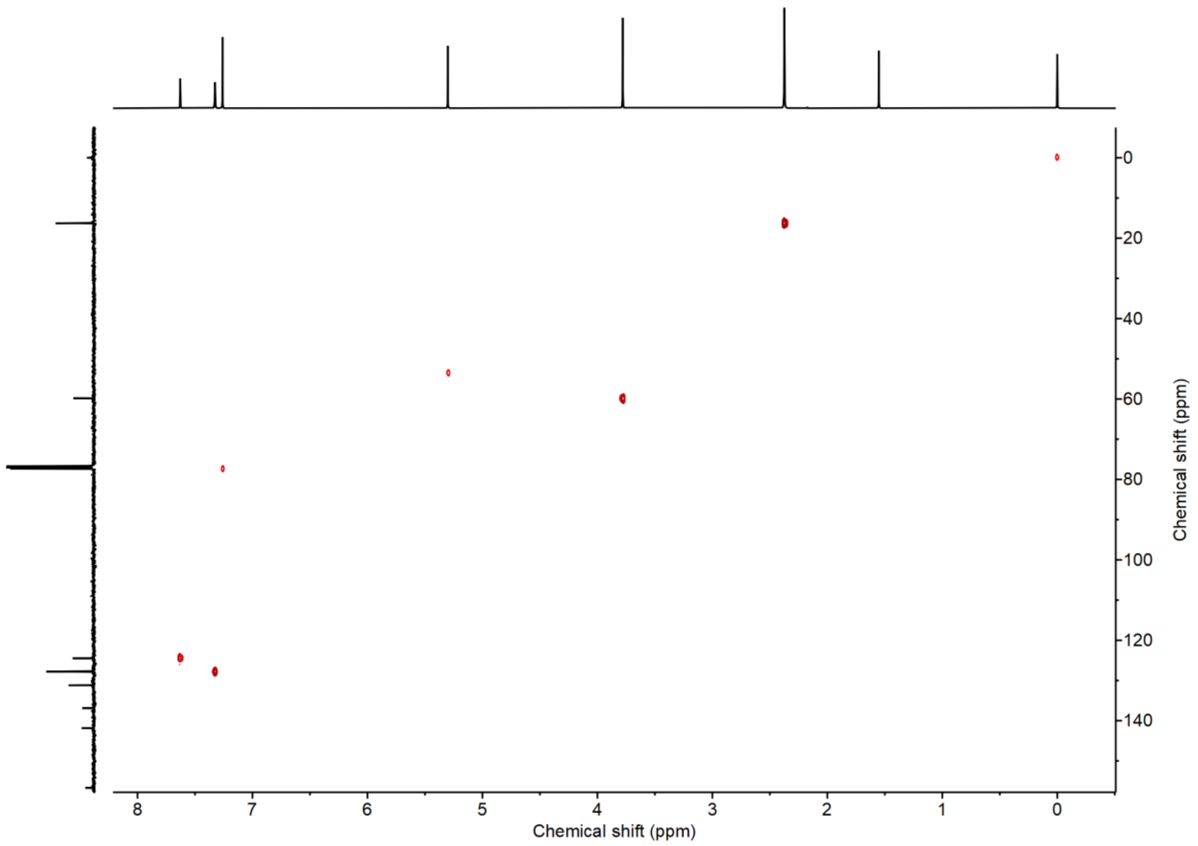

Figure S15 HSQC NMR of 5^{BBB} ($CDCl_3$)

Synthesis of S1^{BBB}

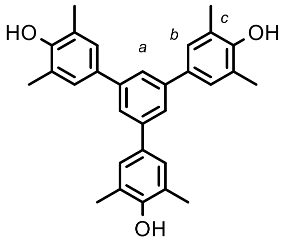

A solution of **5^{BBB}** (0.298 g, 0.62 mmol, 1 eq.) in acetic acid (8 mL) was heated at 110 °C in a reflux apparatus connected to a Dreschel flask containing sat. aq. $NaHCO_3$. 48% $HBr_{(aq)}$ (0.67 mL, 4.5 mmol, 9 eq.) was added dropwise and the reaction stirred for 30 h. To the cooled reaction mixture was added ice water (15 mL). The resultant white precipitate was collected by filtration, washed with H_2O (3 × 10 mL) and dried in air. The off-white solid (0.288 g) was taken forward without further purification.

^{1}H NMR (400 MHz, CD_3OD, 298 K) δ: 7.35 (s, 3H, H_a), 7.09 (s, 6H, H_b), 2.18 (s, 18H, H_c).

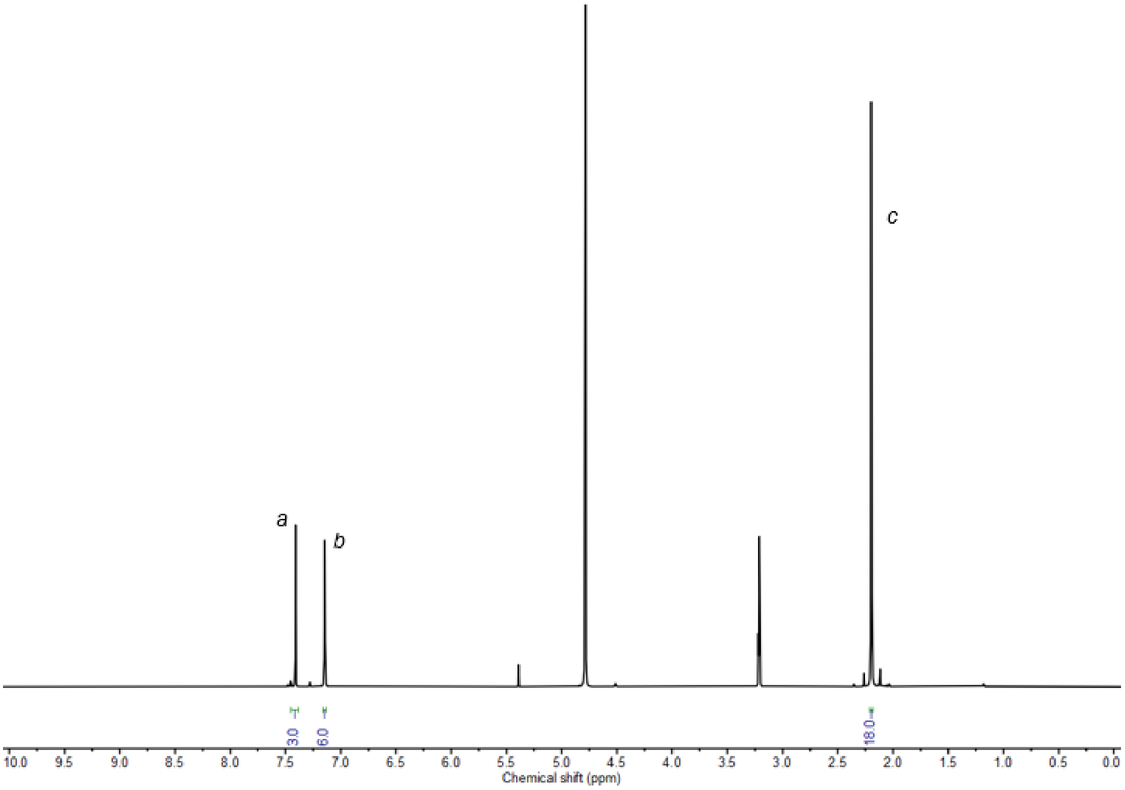

Figure S16 ^{1}H NMR of S1^{BBB} (400 MHz, CD_3OD)

Synthesis of 2^{BBB}

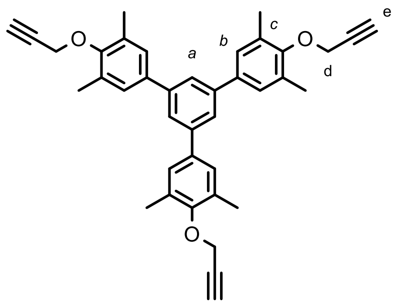

$S1^{BBB}$ (0.205 g) and K_2CO_3 (0.660 g, 4.8 mmol, 10 eq.) were suspended in dry MeCN (5 mL) and stirred at 80 °C. Propargyl bromide solution (80% in toluene, 0.164 mL, 2.16 mmol, 4 eq.) was added to this suspension and allowed to react for 16 h. To the cooled reaction mixture H_2O (20 mL) was added and aqueous phase extracted with CH_2Cl_2 (3 × 20 mL). The combined organic phases were washed with brine (2 × 30 mL), dried ($MgSO_4$) and the solvent removed *in vacuo*. After purification by column chromatography on silica gel (3:2 hexane/CH_2Cl_2) the product was obtained as a white solid (0.1763 g, 85% over 2 steps).

^{1}H NMR (400 MHz, $CDCl_3$, 298 K) δ: 7.63 (s, 3H, H_a), 7.34(s, 6H, H_b), 4.56 (d, J = 2.5 Hz, 6H, H_d), 2.55 (t, J = 2.4 Hz, 3H, H_e), 2.41 (s, 18H, H_c).

^{13}C NMR (101 MHz, $CDCl_3$, 298 K) δ: 155.0, 141.7, 137.4, 131.5, 127.8, 124.6, 79.3, 75.1, 59.9, 16.8.

HR-ESI-MS m/z = 553.2744 $[M+H]^+$ calc. 553.2743.

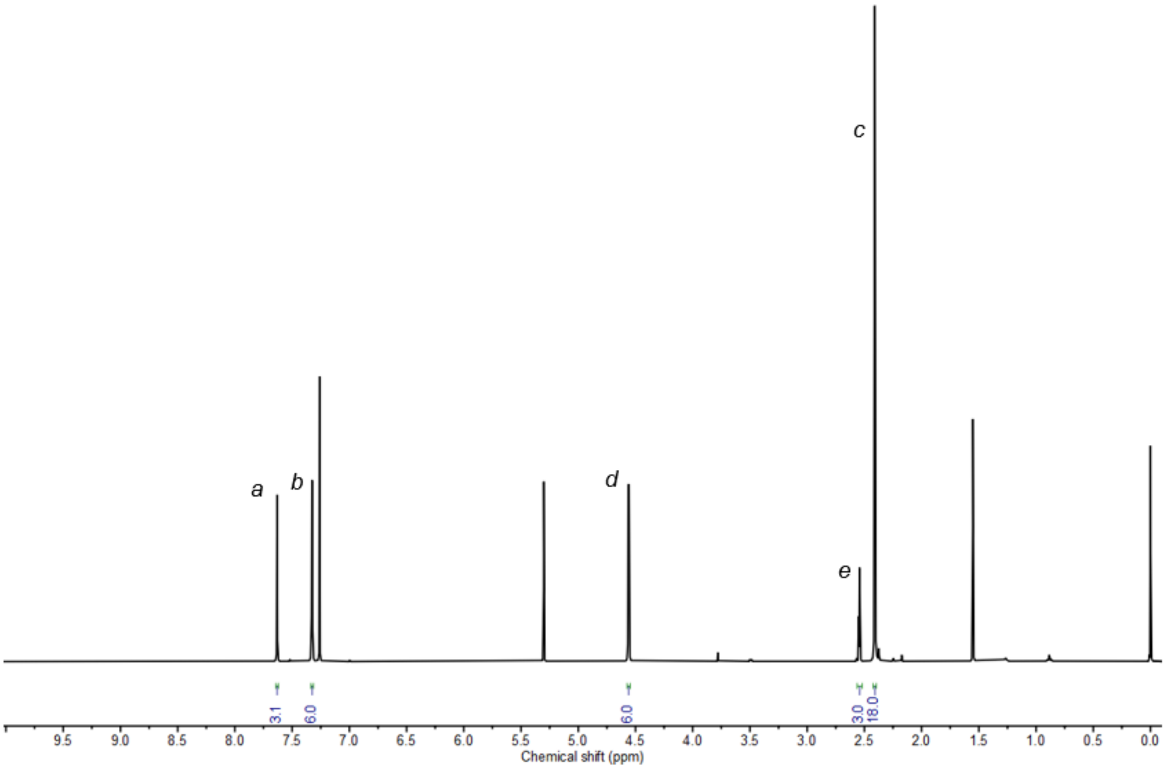

Figure S17 ^{1}H NMR of 2^{BBB} (400 MHz, $CDCl_3$)

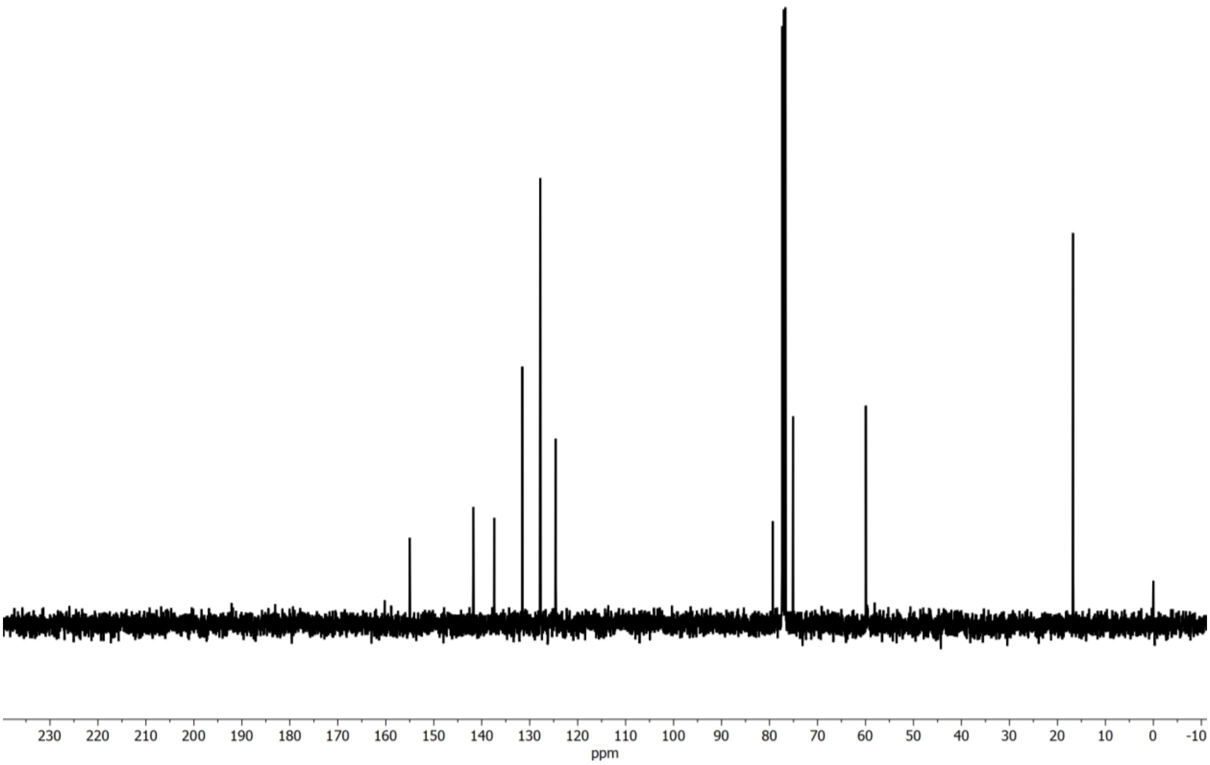

Figure S18 ^{13}C NMR of 2^{BBB} (101 MHz, CDCl₃)

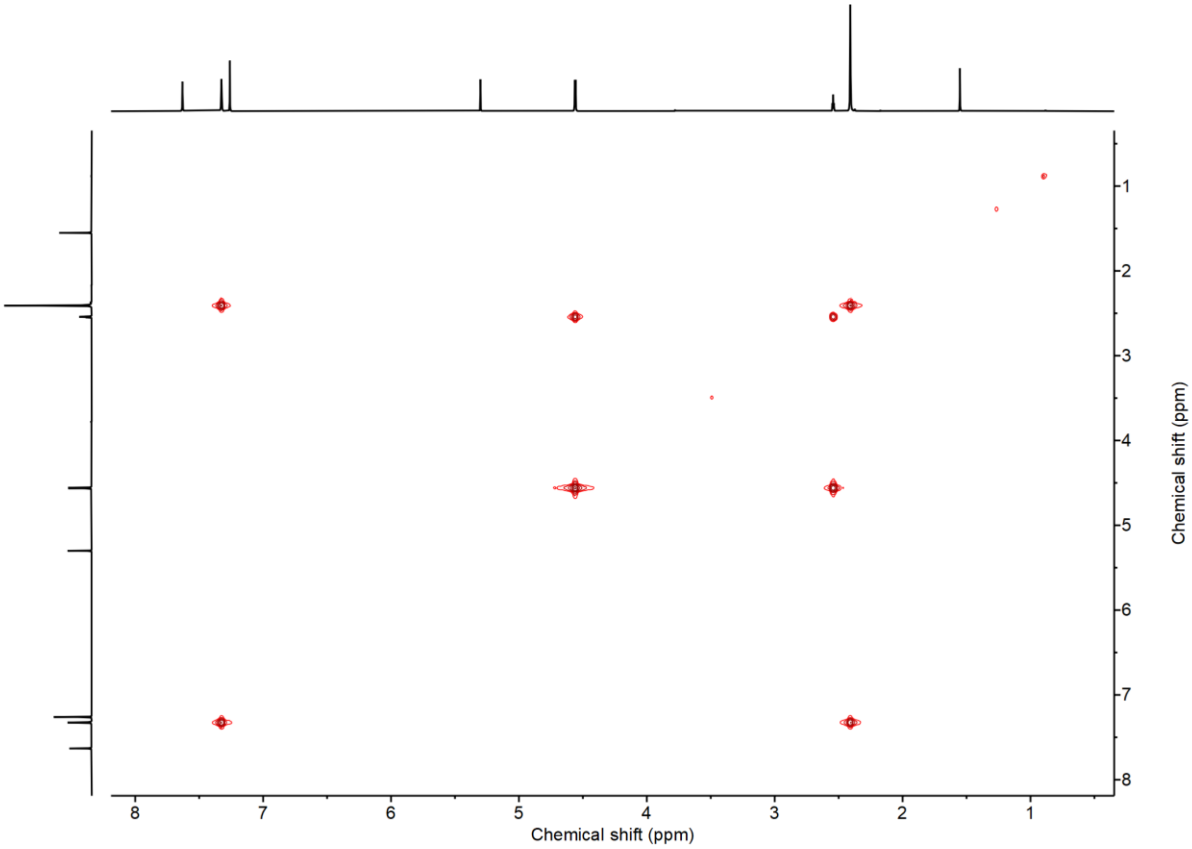

Figure S19 ^{1}H COSY NMR of 2^{BBB} (400 MHz, CDCl₃)

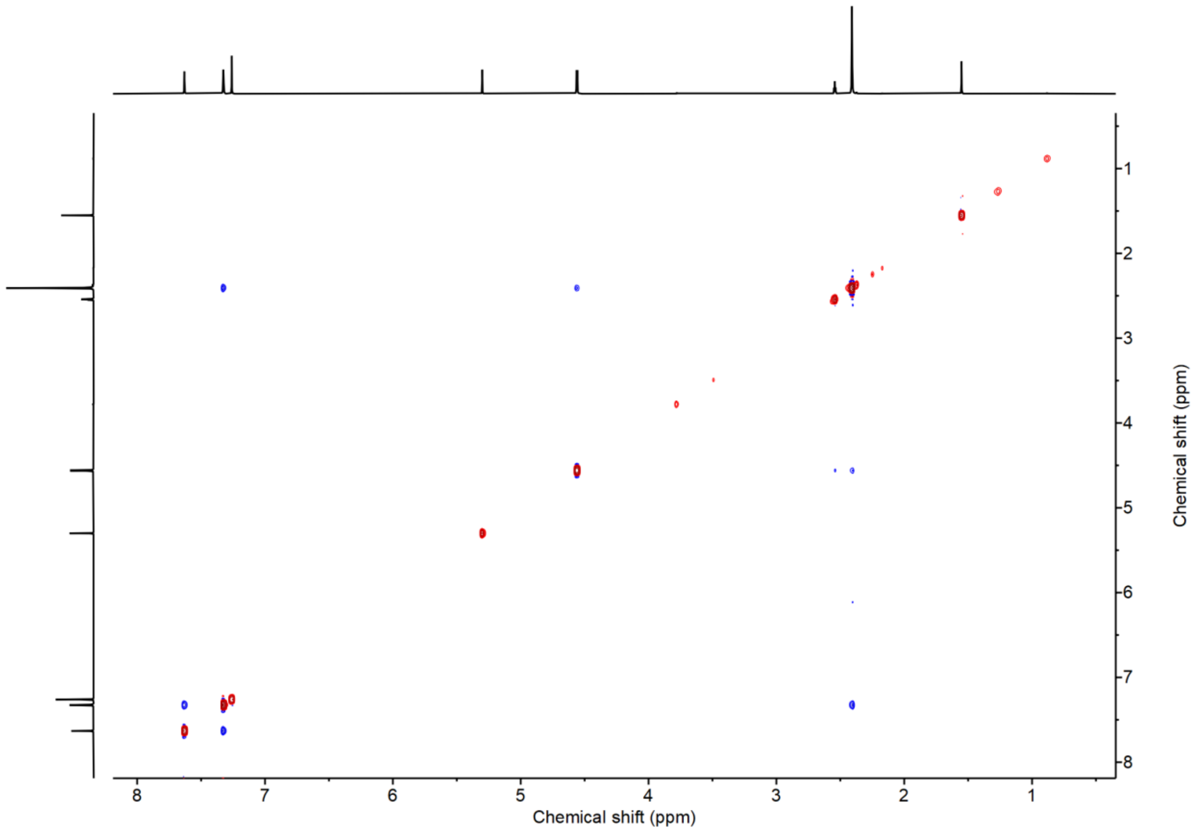

Figure S20 NOESY NMR of 2^{BBB} (400 MHz, $CDCl_3$)

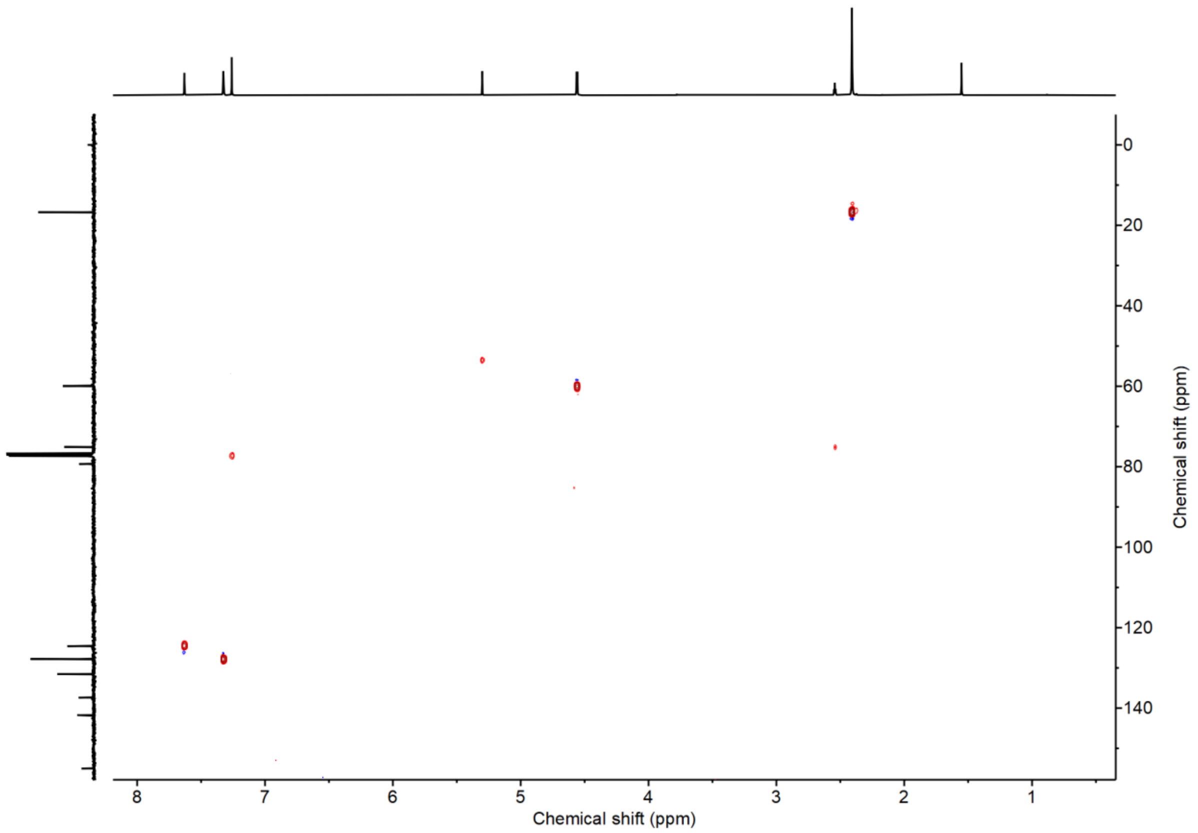

Figure S21 HSQC NMR of 2^{BBB} ($CDCl_3$)

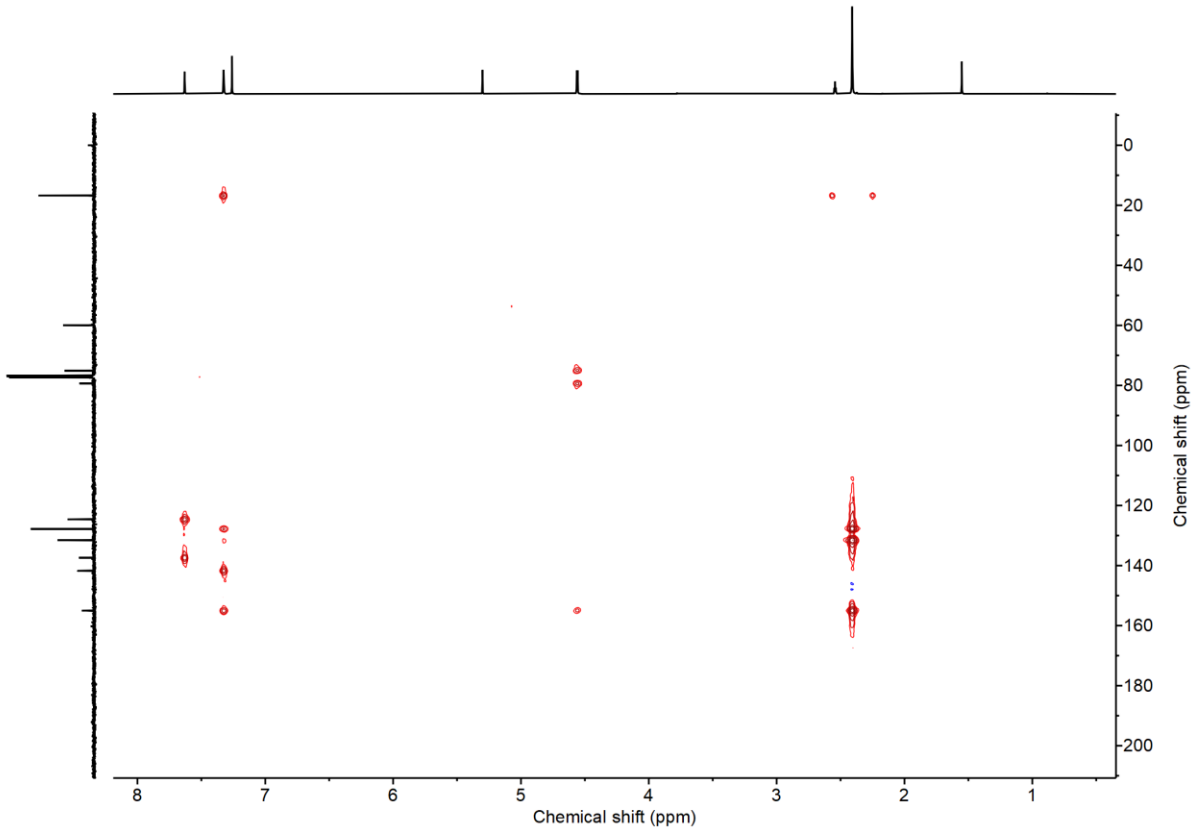

Figure S22 HMBC NMR of 2^{BBB} (CDCl$_3$)

Synthesis of 5^{CCC}

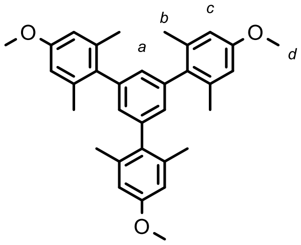

1,3,5-Tribromobenzene (0.157 g, 0.50 mmol, 1.0 eq.) 4-methoxybenzene boronic acid (0.360 g, 2.0 mmol, 4.0 eq.), $Pd(PPh_3)_2Cl_2$ (0.0260 g, 0.0375 mmol, 7.5 mol%), and K_2CO_3 (0.265 g, 3.75 mmol, 7.5 eq.) were stirred at 110 °C in 1:3 H_2O/dioxane (degassed, 12 mL) for 30 h. To the cooled reaction mixture was added H_2O (20 mL) and the aqueous phase extracted with CH_2Cl_2 (3 × 20 mL). The combined organic phases were washed with brine (2 × 30 mL), dried ($MgSO_4$) and the solvent removed *in vacuo*. After purification by column chromatography on silica gel (9:1 hexane/CH_2Cl_2) the product was obtained as a colourless oil (0.1021 g, 54%).

^{1}H NMR (400 MHz, $CDCl_3$, 298 K) δ: 6.87 (s, 3H, H_a), 6.66 (s, 6H, H_c), 3.81 (s, 9H, H_d), 2.10 (s, 18H, H_b).

^{13}C NMR (101 MHz, $CDCl_3$, 298 K) δ: 158.2, 141.0, 137.2, 134.5, 129.2, 112.6, 55.2, 21.2.

HR-ESI-MS *m/z* = 481.2792 $[M+H]^+$ calc. 481.2743.

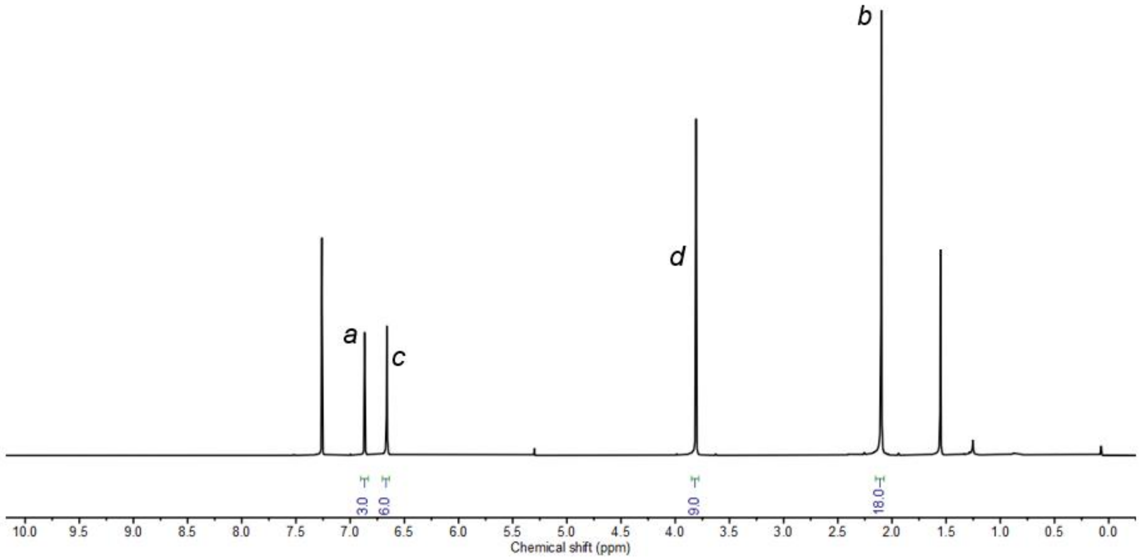

Figure S23 ^{1}H NMR of 5^{CCC} (400 MHz, $CDCl_3$)

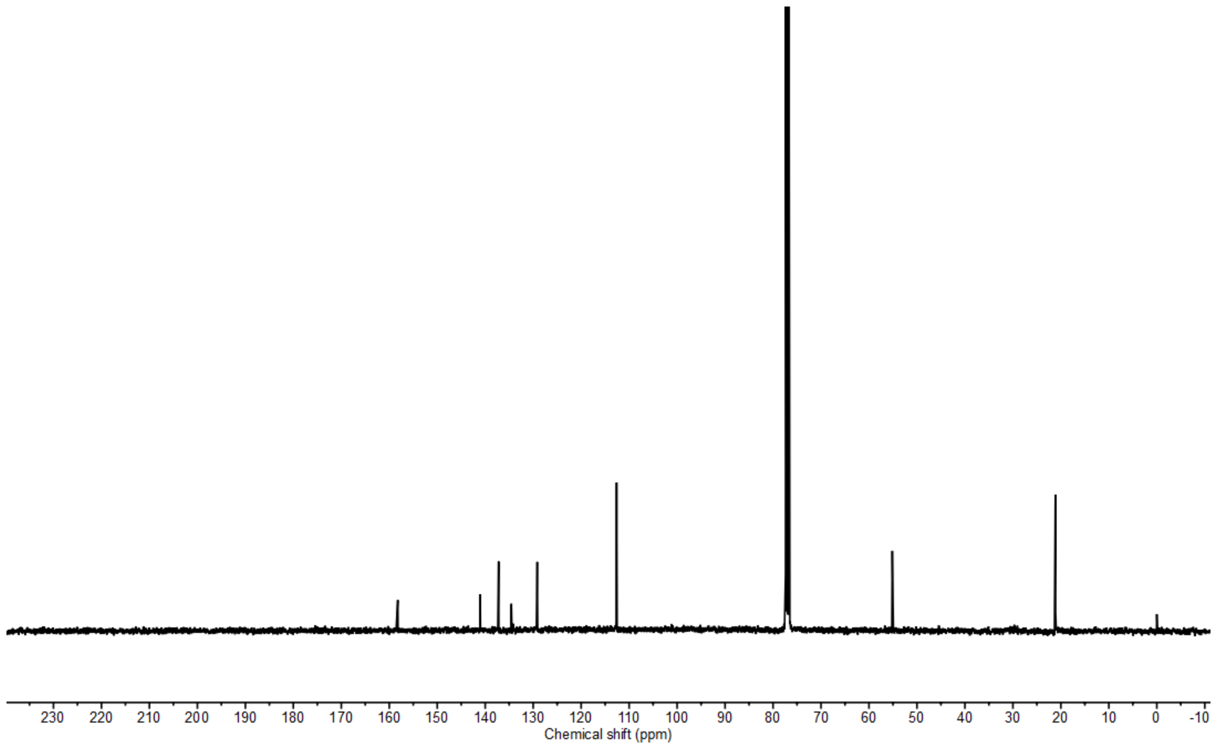

Figure S24 ^{13}C NMR of 5^{CCC} (101 MHz, $CDCl_3$)

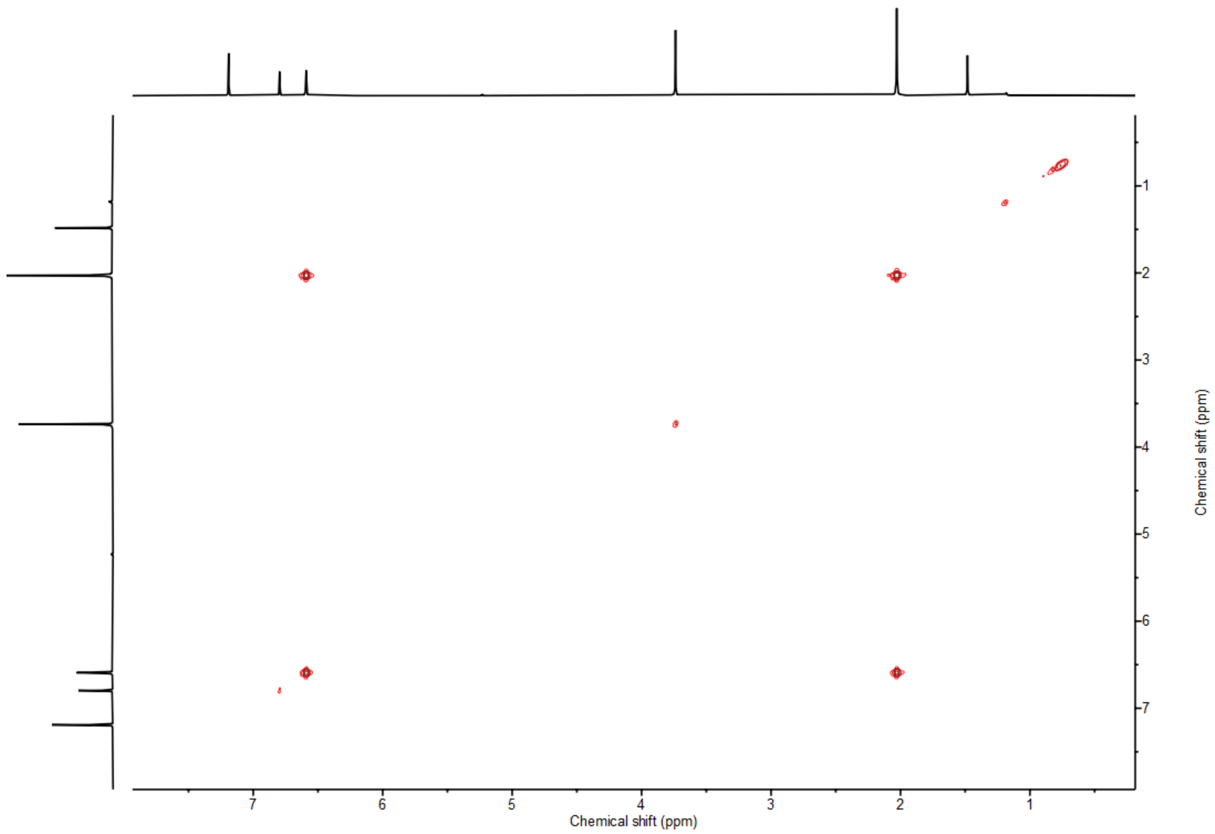

Figure S25 COSY NMR of 5^{CCC} ($CDCl_3$)

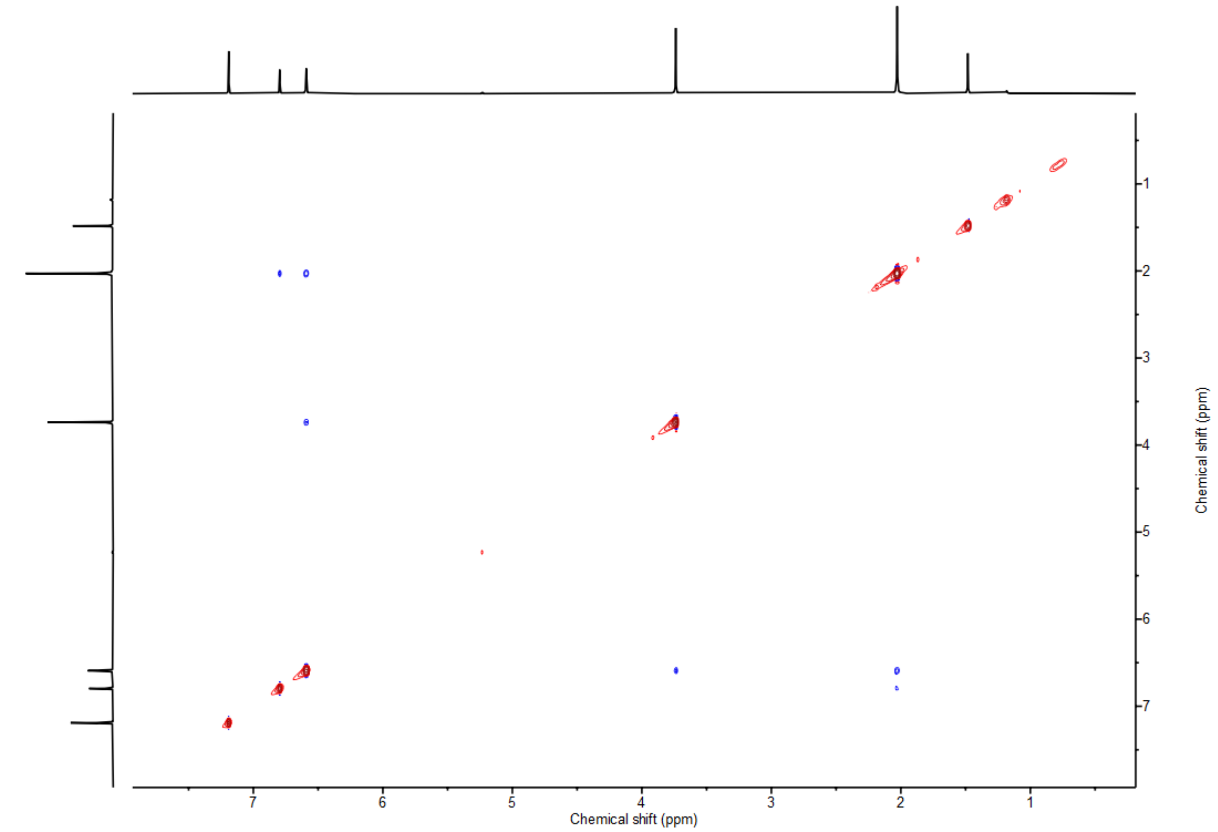

Figure S26 NOESY NMR of 5^{CCC} ($CDCl_3$)

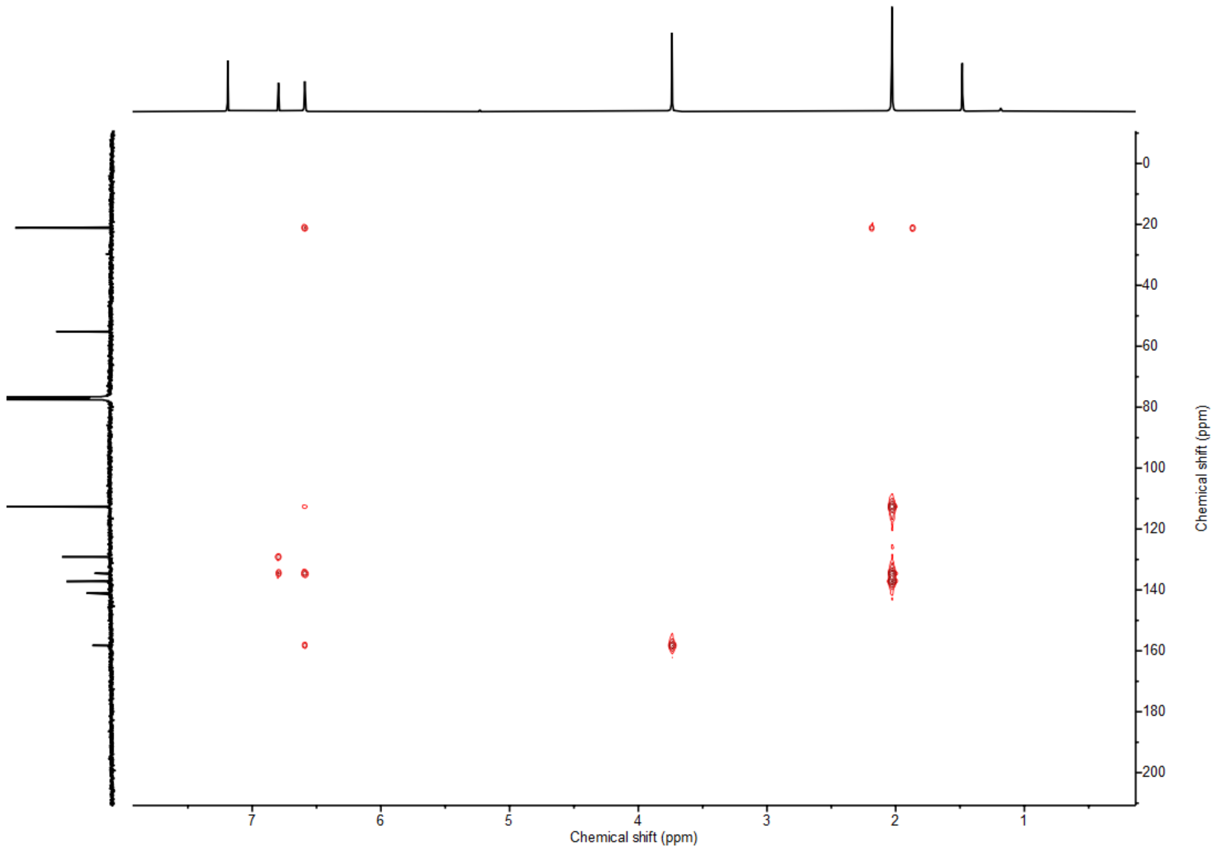

Figure S27 HMBC of 5^{CCC} ($CDCl_3$)

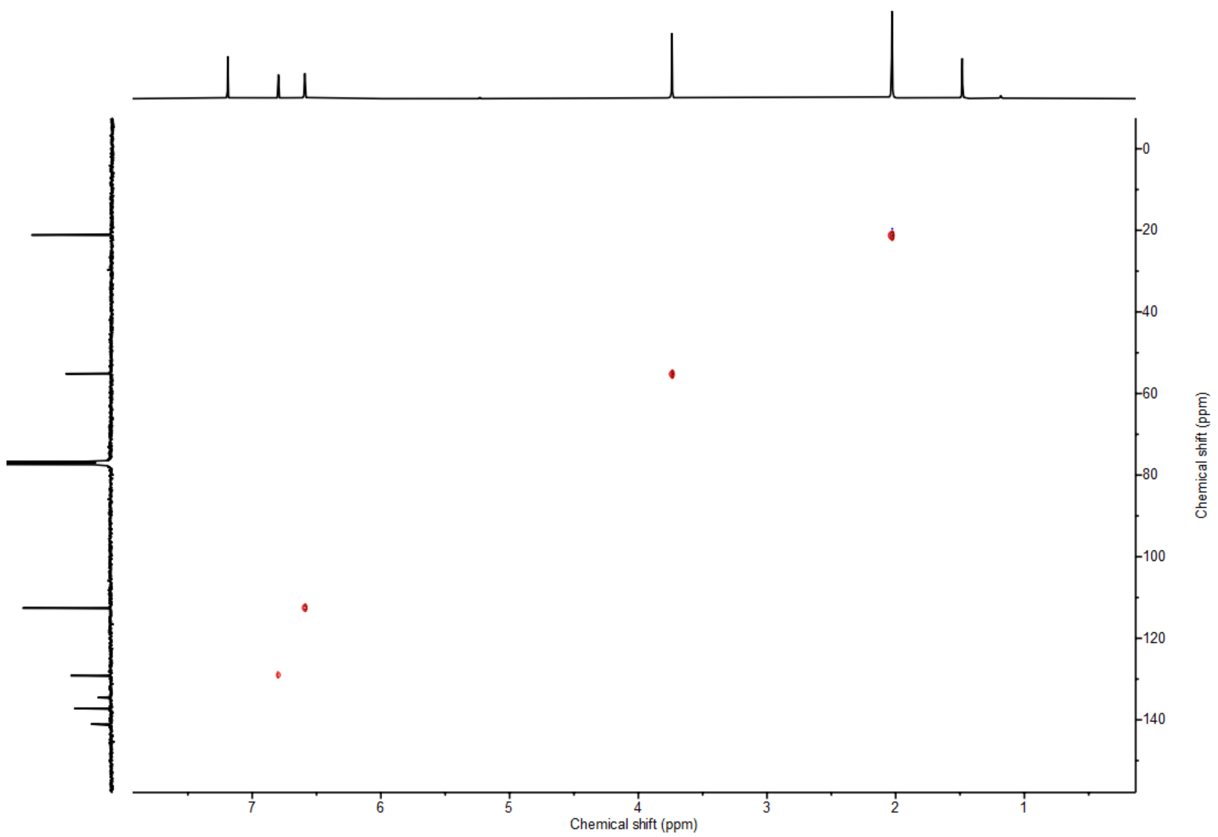

Figure S28 HSQC of 5CCC ($CDCl_3$)

Synthesis of S1^{CCC}

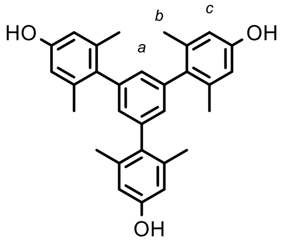

A solution of $\mathbf{5^{CCC}}$ (0.0843 g, 0.175 mmol, 1 eq.) in acetic acid (3 mL) was heated at 110 °C in a reflux apparatus connected to a Dreschel flask containing sat. aq. $NaHCO_3$. 48% $HBr_{(aq)}$ (0.18 mL, 4.5 mmol, 9 eq.) was added dropwise and the reaction stirred for 30 h. To the cooled reaction mixture was added ice water (15 mL). The resultant white precipitate was collected by filtration, washed with H_2O (3 × 10 mL) and dried in air. The off-white solid (0.0917 g) was taken forward without further purification.

^{1}H NMR (400 MHz, CD_3OD, 298 K) δ: 6.76 (s, 3H, H$_a$), 6.57 (s, 6H, H$_c$), 2.05 (s, 18H, H$_b$).

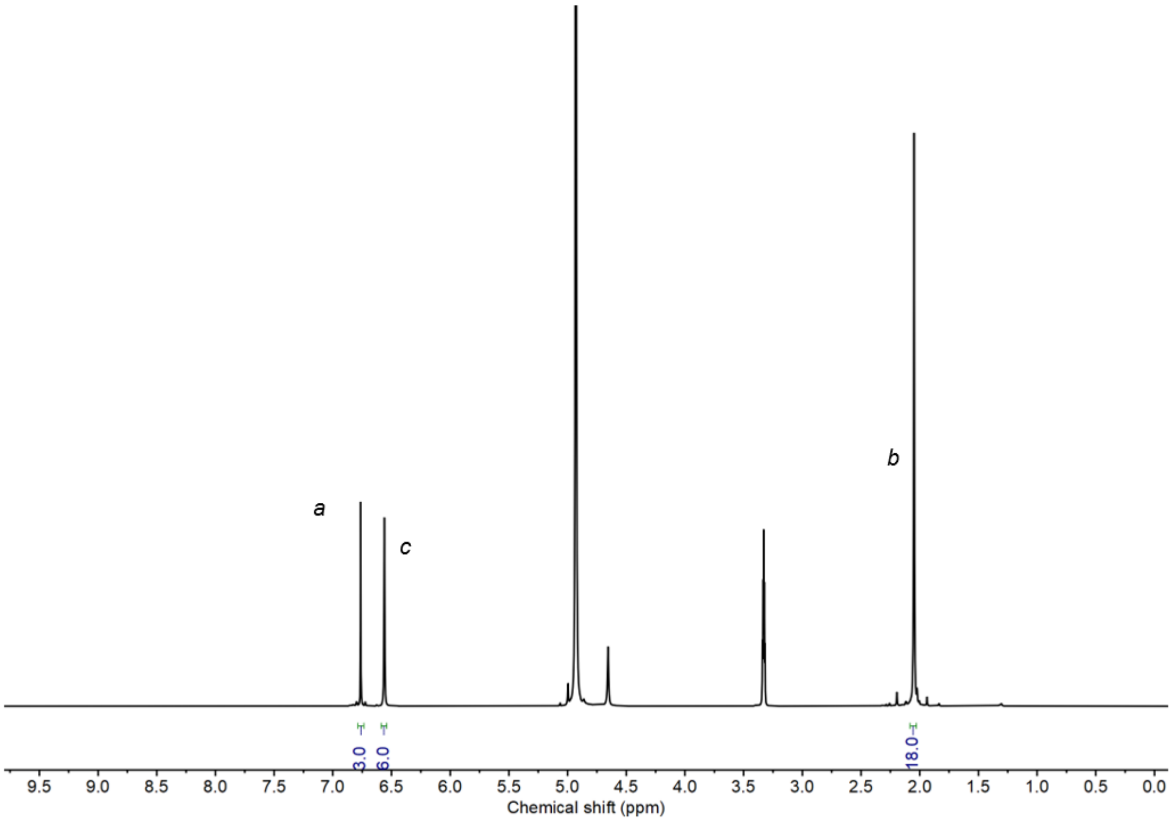

Figure S29 ^{1}H NMR of S1^{CCC} (400 MHz, CD_3OD)

Synthesis of 2^{CCC}

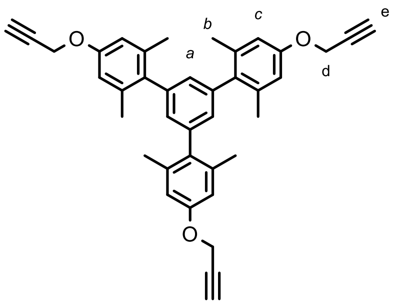

S1^{CCC} (0.0528 g) and K_2CO_3 (0.165 g, 1.2 mmol, 10 eq.) were suspended in dry MeCN (3 mL) and stirred at 80 °C. Propargyl bromide solution (80% in toluene, 0.041 mL, 0.54 mmol, 4 eq.) was added to this suspension and allowed to react for 16 h. To the cooled reaction mixture H_2O (15 mL) was added and aqueous phase extracted with CH_2Cl_2 (3 × 15 mL). The combined organic phases were washed with brine (2 × 20 mL), dried ($MgSO_4$) and the solvent removed *in vacuo*. After purification by column chromatography on silica gel (1:4 hexane/CH_2Cl_2) the product was obtained as a colourless oil. (0.0420 g, 75% over 2 steps).

^{1}H NMR (400 MHz, $CDCl_3$, 298 K) δ: 6.80 (s, 3H, H_a), 6.66 (s, 6H, H_c), 4.62 (d, J = 2.4 Hz, 6H, H_d), 2.46 (t, J = 2.4 Hz, 3H, H_e), 2.03 (s, 18H, H_b).

^{13}C NMR (101 MHz, $CDCl_3$, 298 K) δ: 156.2, 141.0, 137.3, 135.3, 129.1, 113.5, 78.9, 75.3, 55.7, 21.2.

HR-ESI-MS *m/z* = 553.2744 $[M+H]^+$ calc. 553.2743.

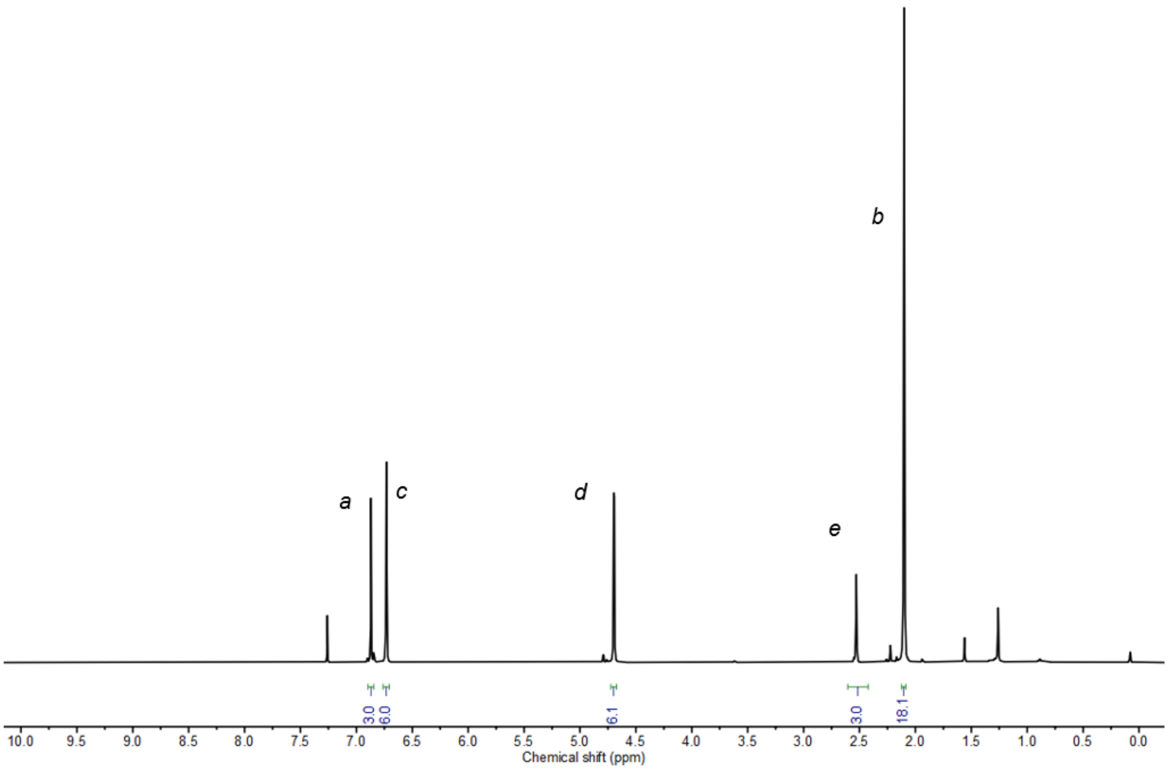

Figure S30 **^{1}H NMR of 2^{CCC} (400 MHz, $CDCl_3$)**

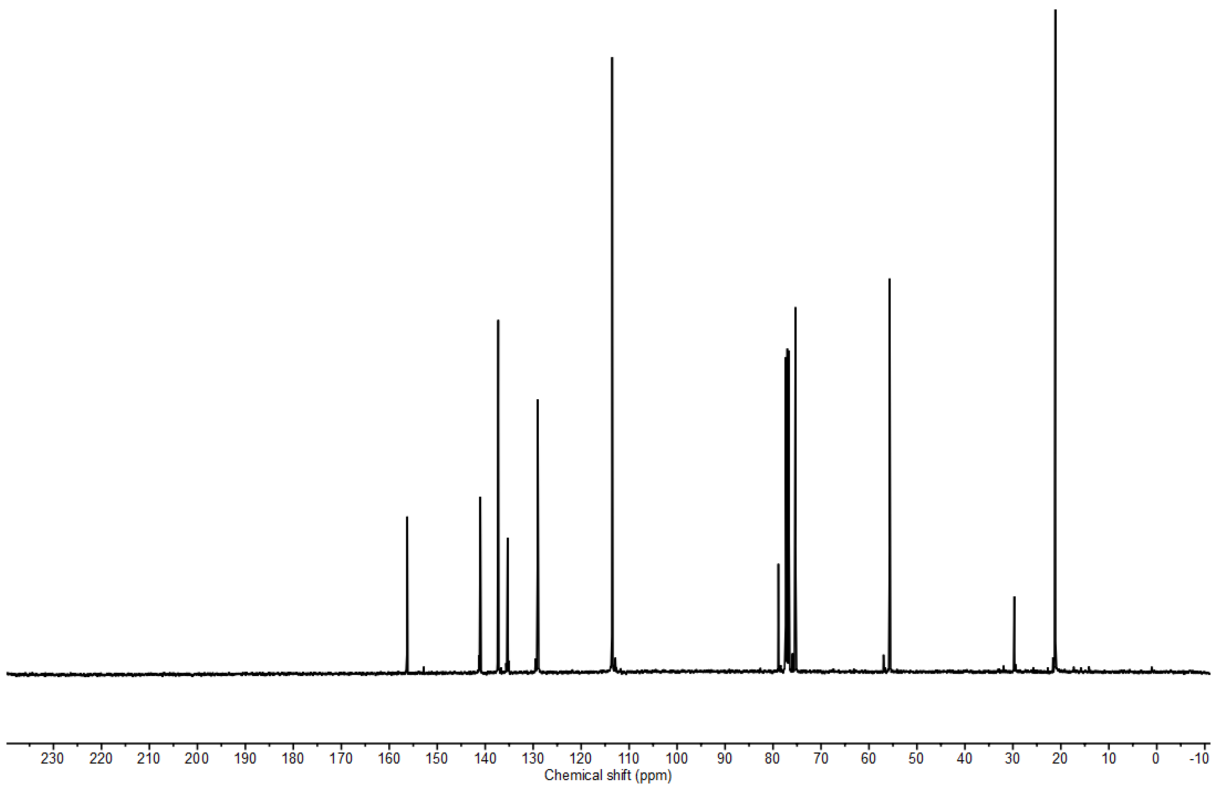

Figure S31 ^{13}C NMR of 2^{CCC} (101 MHz, $CDCl_3$)

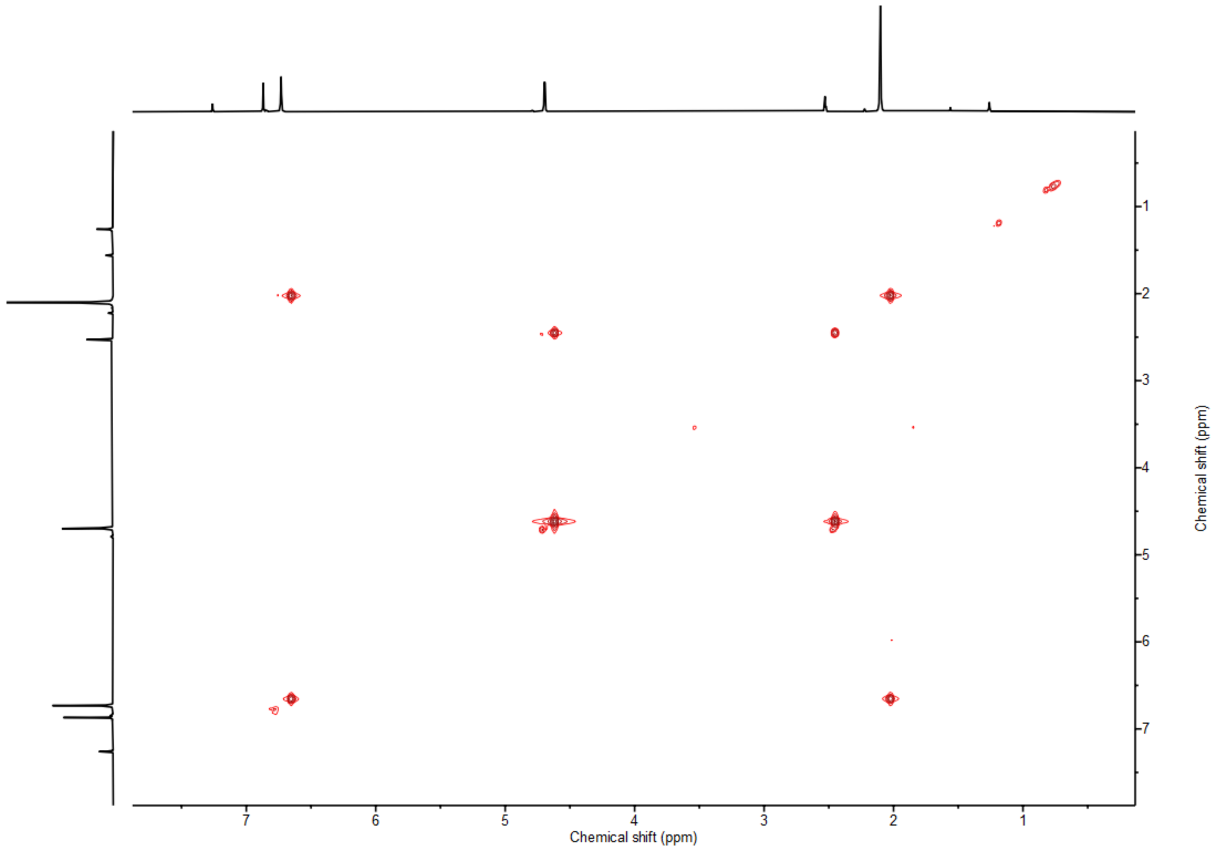

Figure S32 COSY NMR of 2^{CCC} (400 MHz, $CDCl_3$)

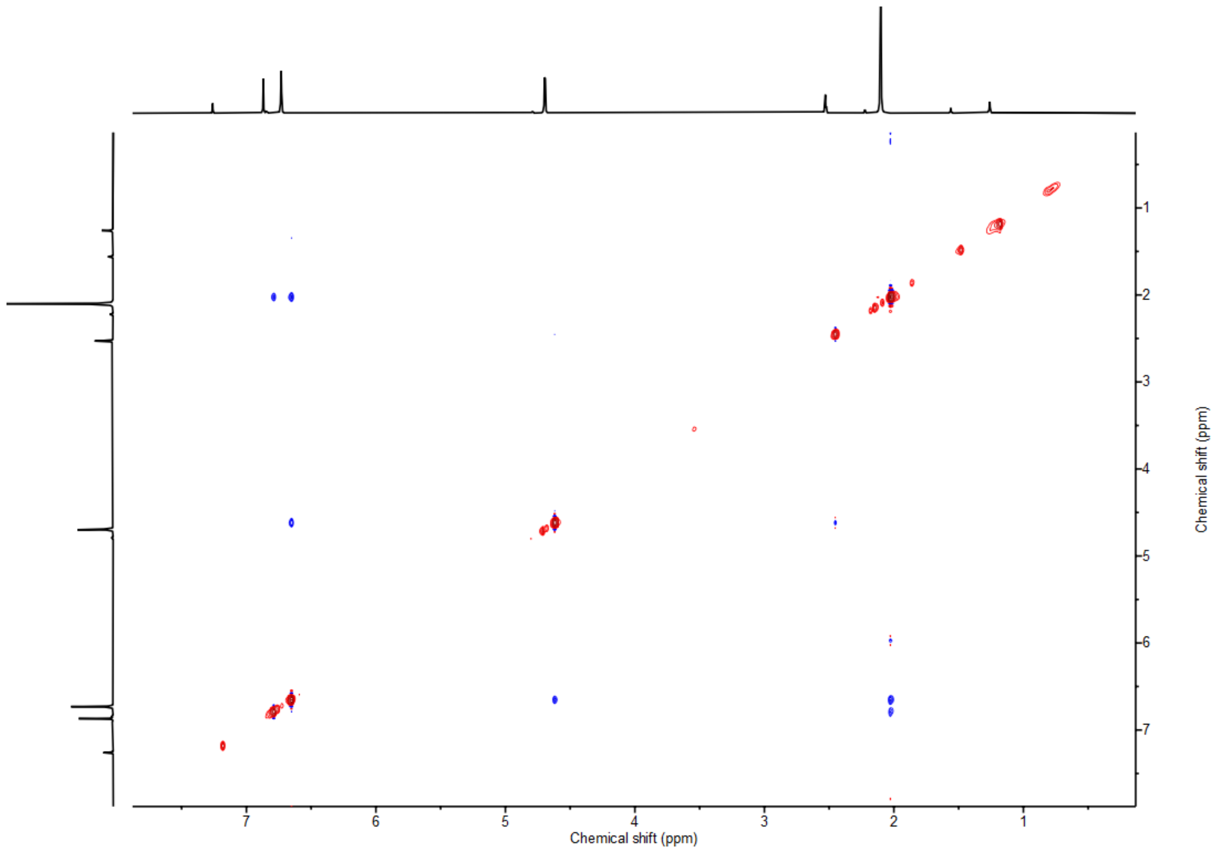

Figure S33 NOESY NMR of 2^{CCC} (400 MHz, $CDCl_3$)

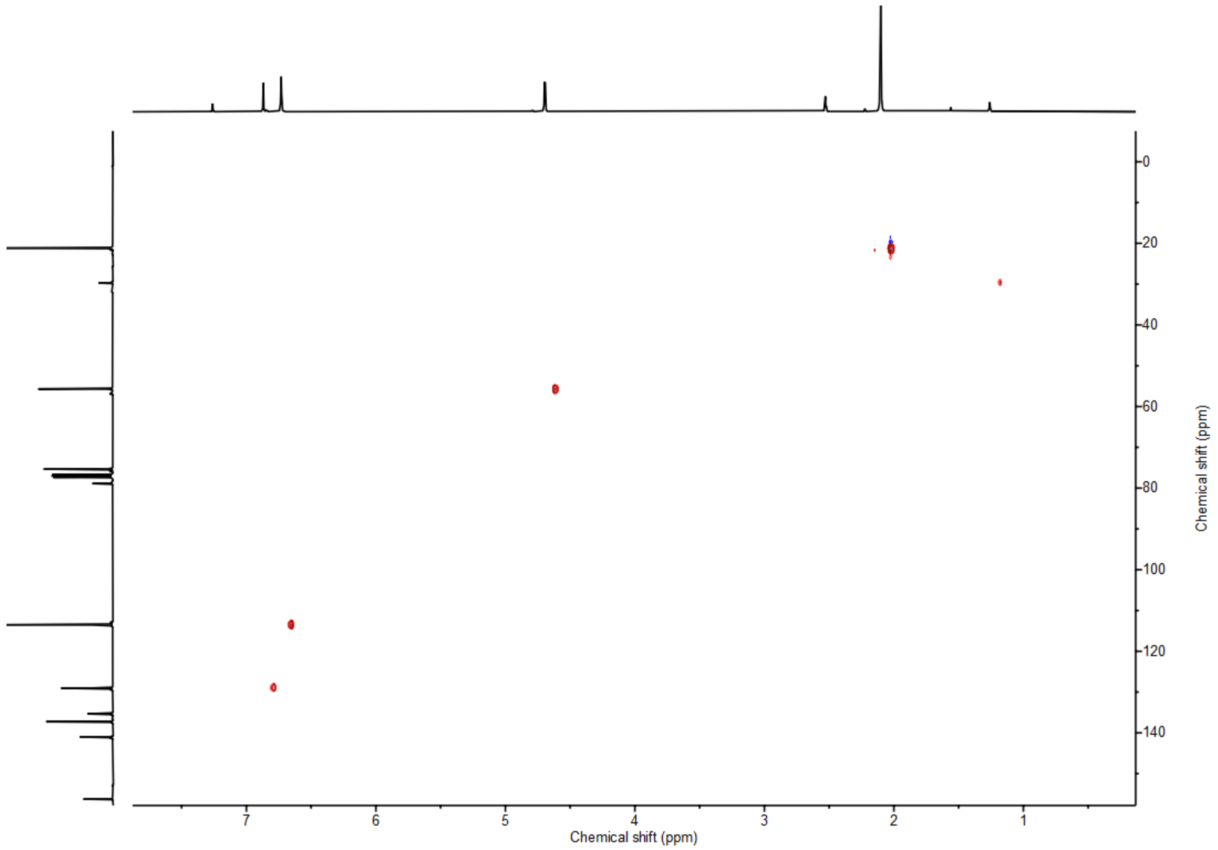

Figure S34 HSQC NMR of 2^{CCC} ($CDCl_3$)

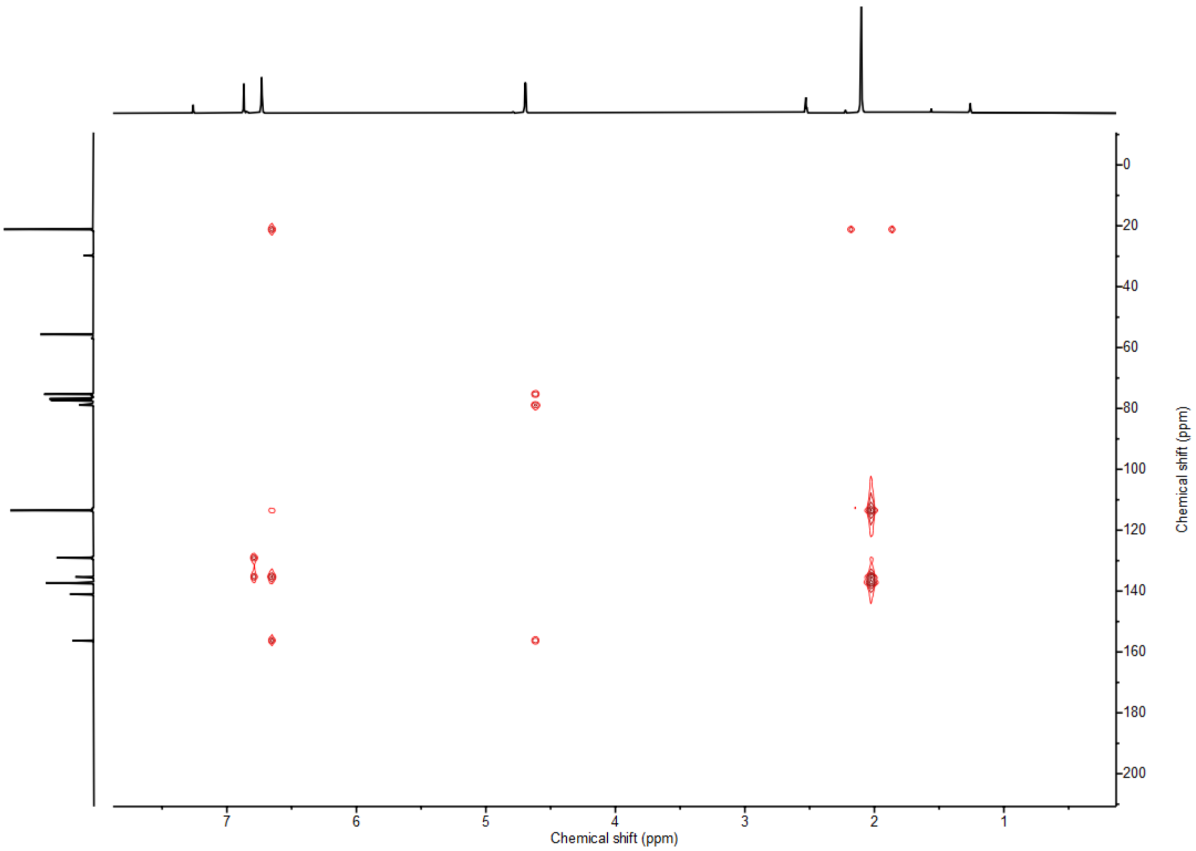

Figure S35 HMBC NMR of 2^{CCC} ($CDCl_3$)

Synthesis of 5[A]

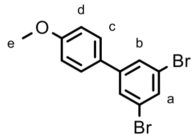

1,3,5-Tribromobenzene (4.00 g, 12.5 mmol, 2.5 eq.), 4-methoxybenzene boronic acid (0.7580 g, 5 mmol, 1 eq.), $Pd(PPh_3)_2Cl_2$ (0.170 g, 0.25 mmol, 5 mol%), and K_2CO_3 (1.75 g, 1.25 mmol, 2.5 eq.) were stirred at 110 °C in 1:3 H_2O/dioxane (degassed, 15 mL) for 24 h. To the cooled reaction mixture was added H_2O (60 mL) and the aqueous phase extracted with CH_2Cl_2 (3 × 50 mL). The combined organic phases were washed with brine (2 × 50 mL), dried ($MgSO_4$) and the solvent removed *in vacuo*. After purification by column chromatography on silica gel (9:1 hexane/CH_2Cl_2) the product was obtained as a white solid (0.7195 g, 42%).

^{1}H NMR (400 MHz, $CDCl_3$, 298 K) δ: 7.61 (d, *J* = 1.8 Hz, 2H, H_b), 7.58 (t, *J* = 1.8 Hz, 1H, H_a), 7.46 (d, *J* = 9.0 Hz, 2H, H_c), 6.97 (d, *J* = 8.9 Hz, 2H, H_d), 3.85 (s, 3H, H_e).

^{13}C NMR (101 MHz, $CDCl_3$, 298 K) δ: 161.3, 144.4, 131.9, 130.8, 128.5, 128.2, 123.2, 114.5, 55.4.

HR-ESI-MS *m/z* = 340.9142 $[M+H]^+$ calc. 340.9177.

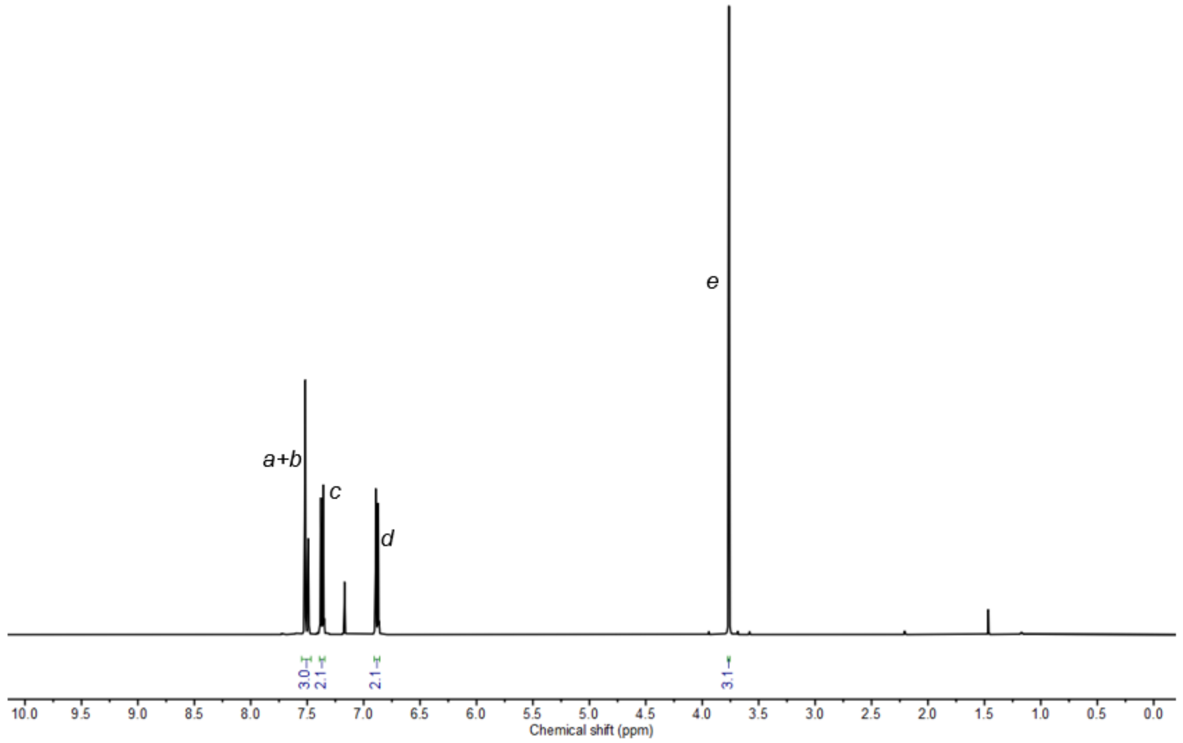

Figure S36 ^{1}H NMR of 5^A (400 MHz, $CDCl_3$)

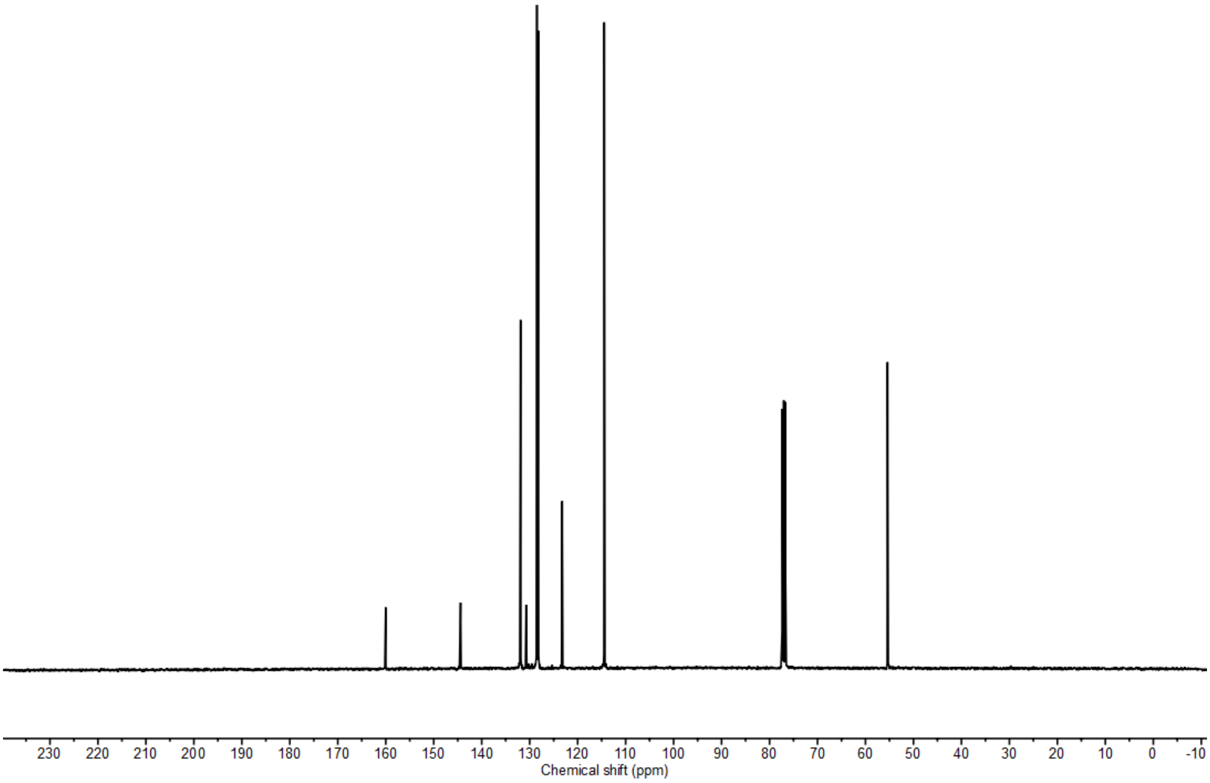

Figure S37 ^{13}C NMR of 5^{A} (101 MHz, $CDCl_3$)

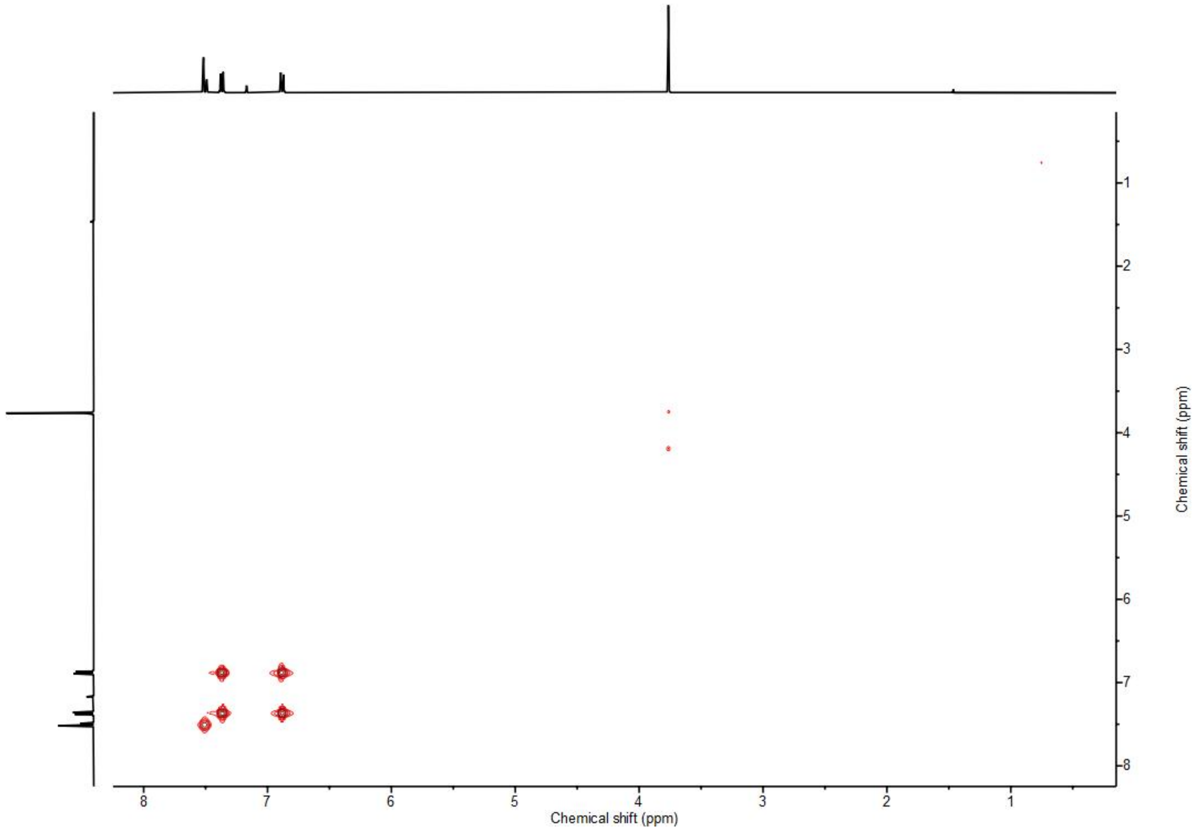

Figure S38 COSY NMR of 5^{A} (400 MHz, $CDCl_3$)

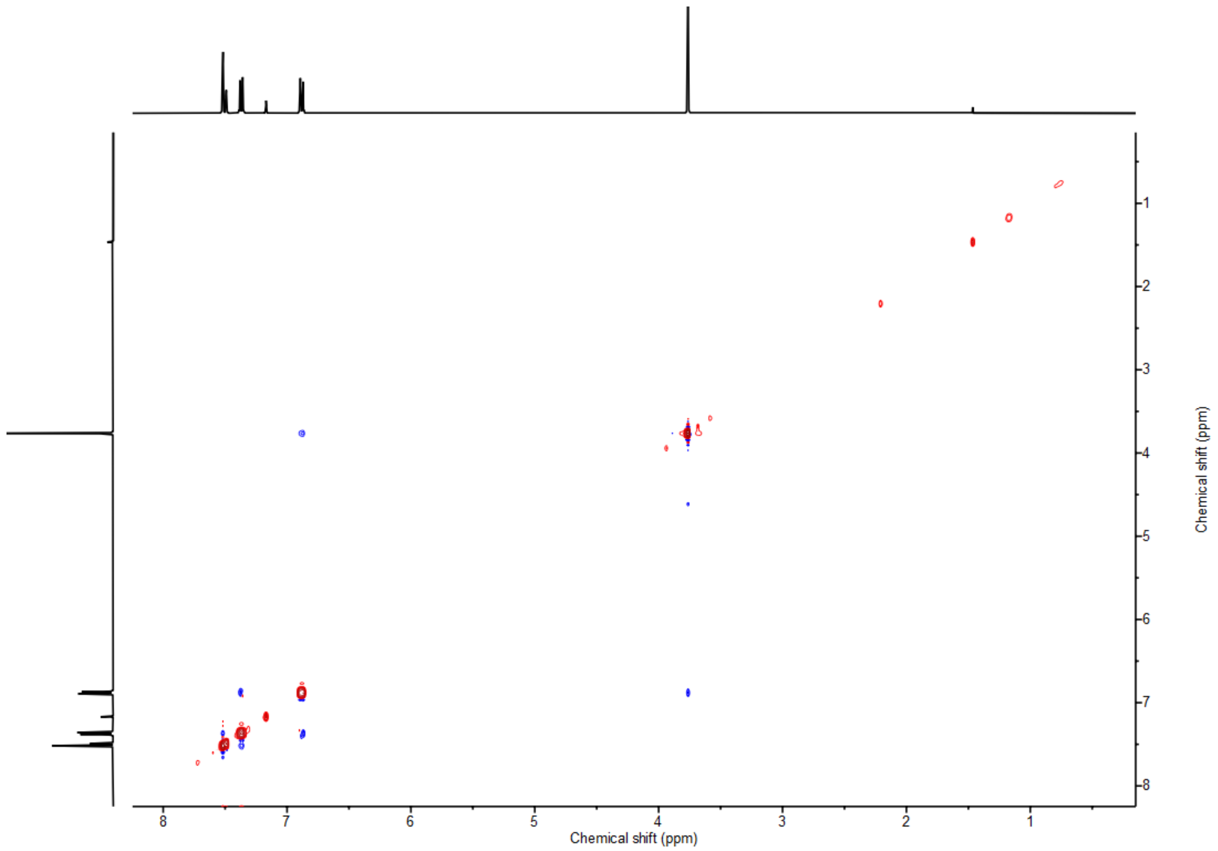

Figure S39 NOESY NMR of 5[A] (400 MHz, $CDCl_3$)

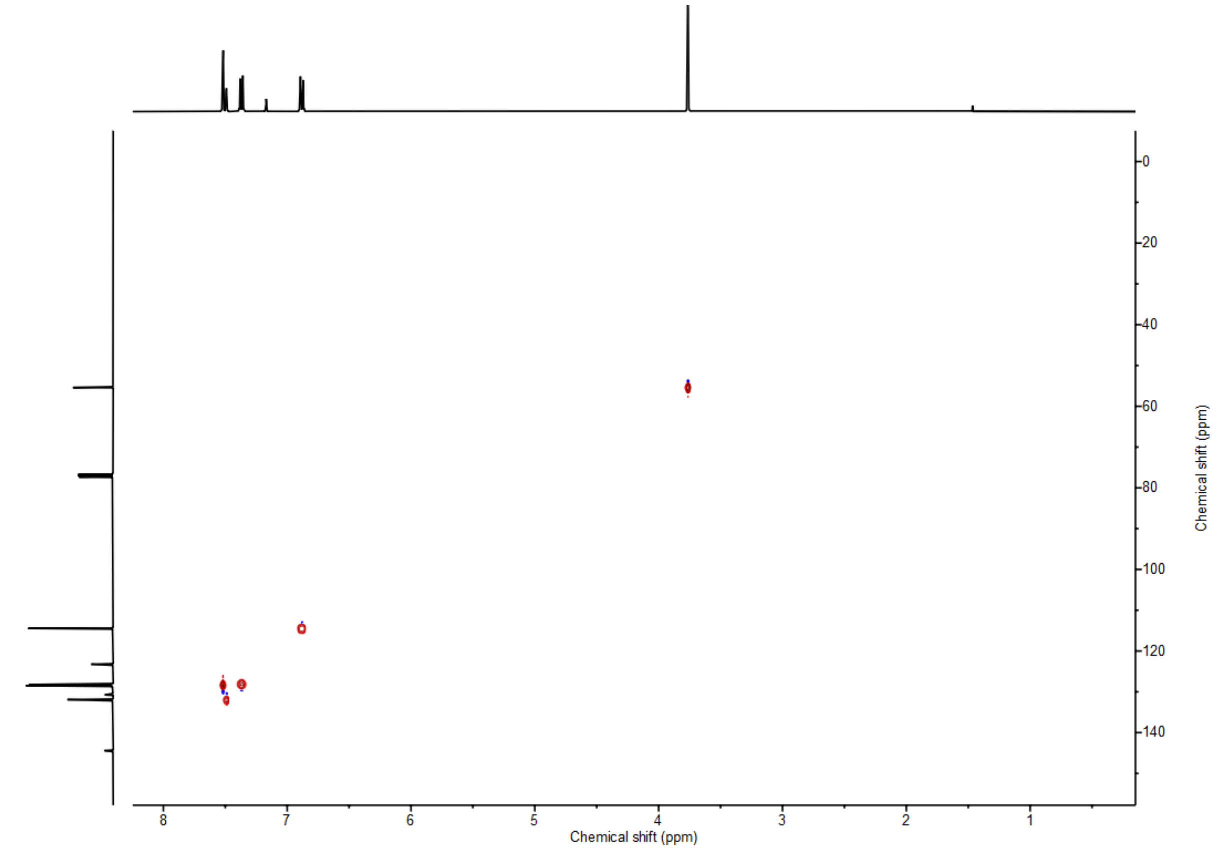

Figure S40 HSQC NMR of 5[A] ($CDCl_3$)

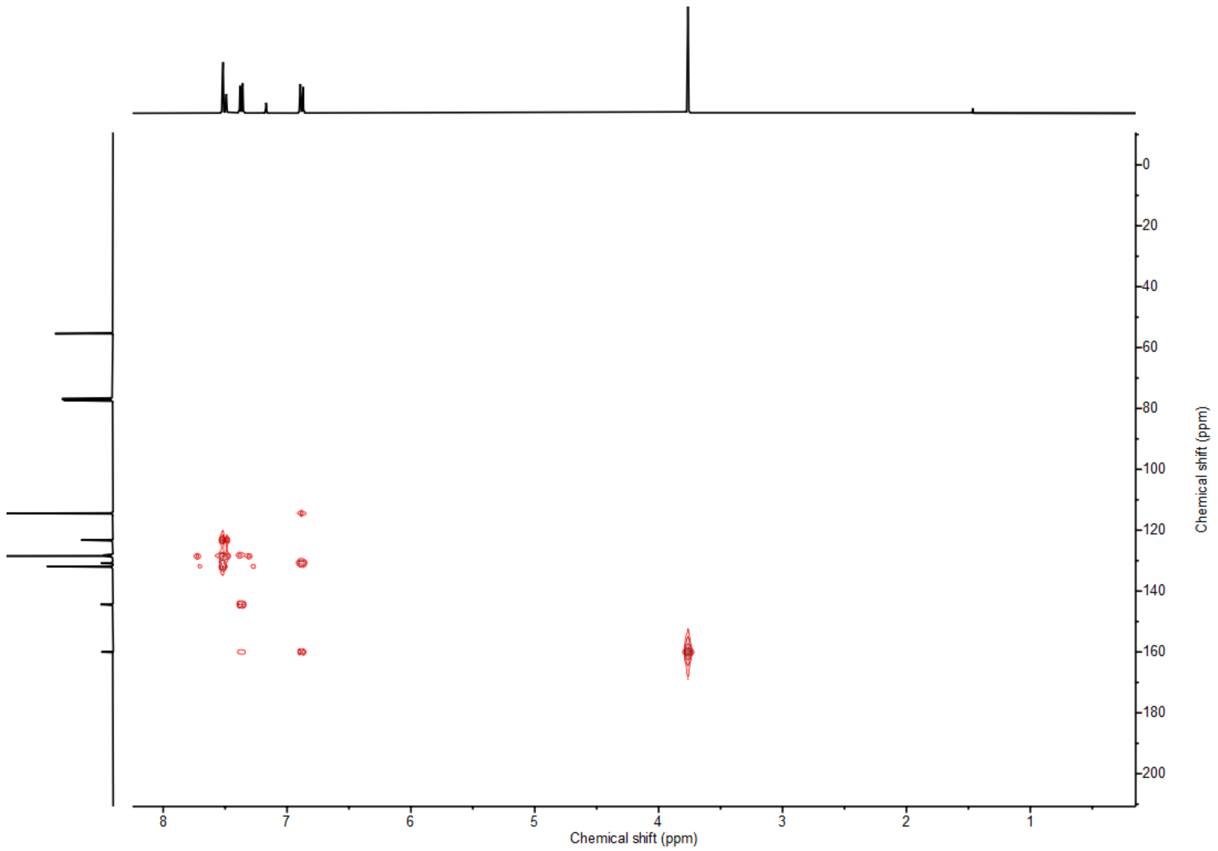

Figure S41 HMBC NMR of 5A (CDCl$_3$)

Synthesis of 5^{ABB}

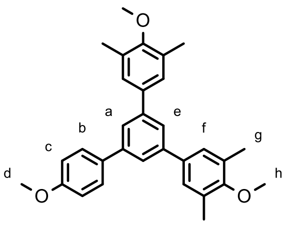

5^{A} (0.145 g, 0.425 mmol, 1 eq.), 4-methoxy-3,5-dimethylphenylboronic acid (0.180 g, 0.977 mmol, 2.3 eq.), $Pd(PPh_3)_2Cl_2$ (0.015 g, 0.021 mmol, 5 mol%), and K_2CO_3 (0.147 g, 1.06 mmol, 2.5 eq.) were stirred at 110 °C in 1:3 H_2O/dioxane (degassed, 15 mL) for 24 h. To the cooled reaction mixture was added H_2O (60 mL) and the aqueous phase extracted with CH_2Cl_2 (3 × 50 mL). The combined organic phases were washed with brine (2 × 50 mL), dried ($MgSO_4$) and the solvent removed *in vacuo*. After purification by column chromatography on silica gel (0-40% gradient CH_2Cl_2 in hexane) the product was obtained as a white solid (0.188 g, 98%).

^{1}H NMR (400 MHz, $CDCl_3$, 298 K) δ: 7.66 – 7.60 (m, 5H, H_a, H_b, H_e), 7.33 (s, 4H, H_f), 7.01 (d, J = 8.8 Hz, 2H, H_c), 3.87 (s, 3H, H_d), 3.78 (s, 6H, H_h), 2.37 (s, 12H, H_g).

^{13}C NMR (101 MHz, $CDCl_3$, 298 K) δ: 159.3, 156.7, 141.9, 141.7, 136.9, 133.9, 131.2, 128.4, 127.8, 124.3, 124.3, 114.2, 59.8, 55.4, 16.3.

HR-ESI-MS *m/z* = 453.2446 $[M+H]^+$ calc. 453.2430.

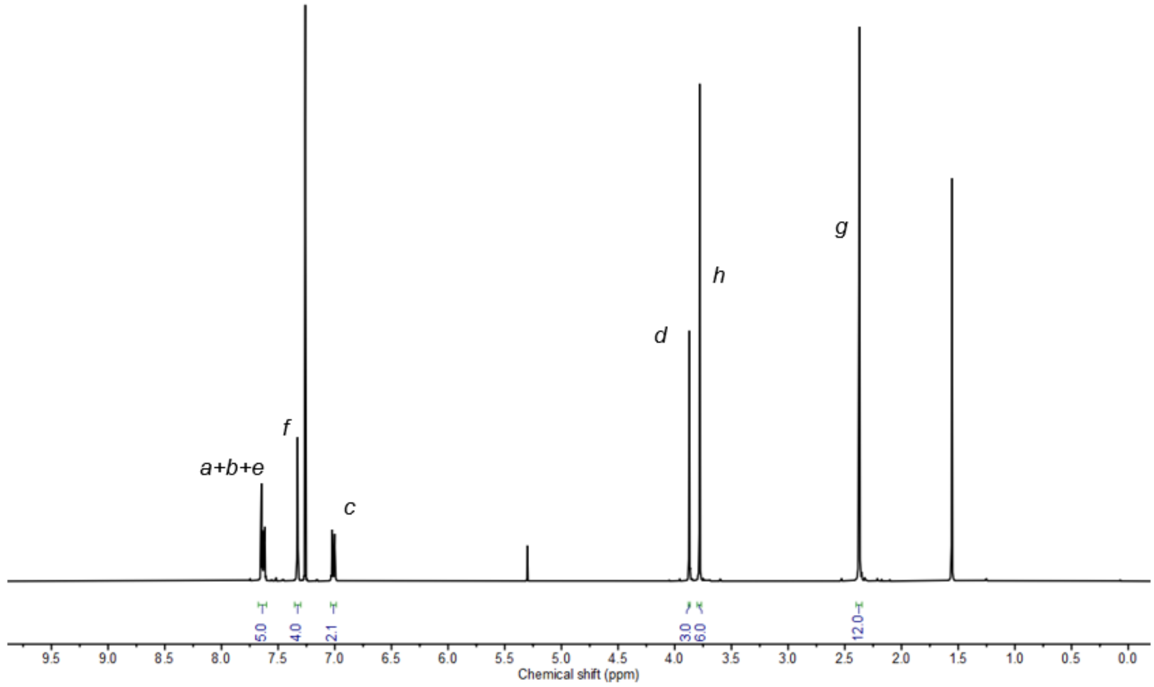

Figure S42 ^{1}H NMR of 5^{ABB} (400 MHz, $CDCl_3$)

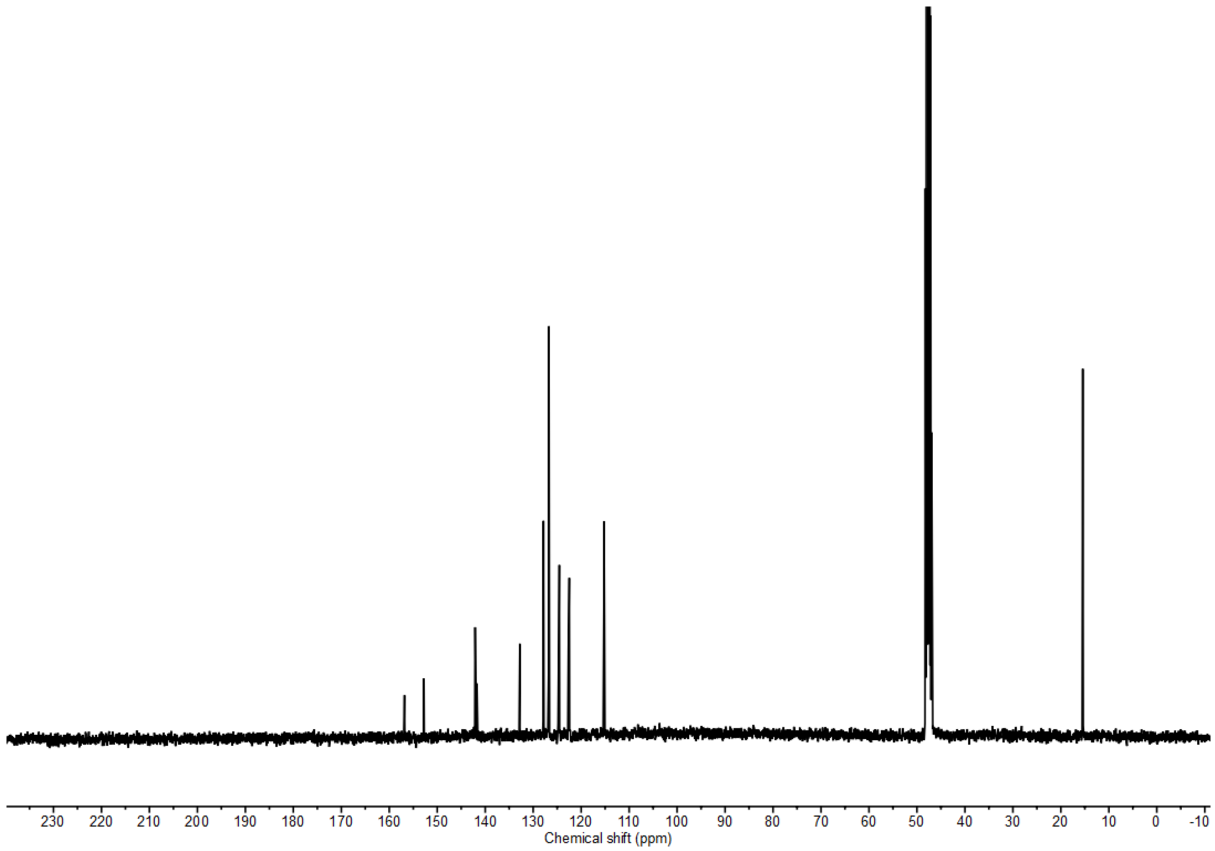

Figure S43 ^{13}C NMR of 5^{ABB} (101 MHz, $CDCl_3$)

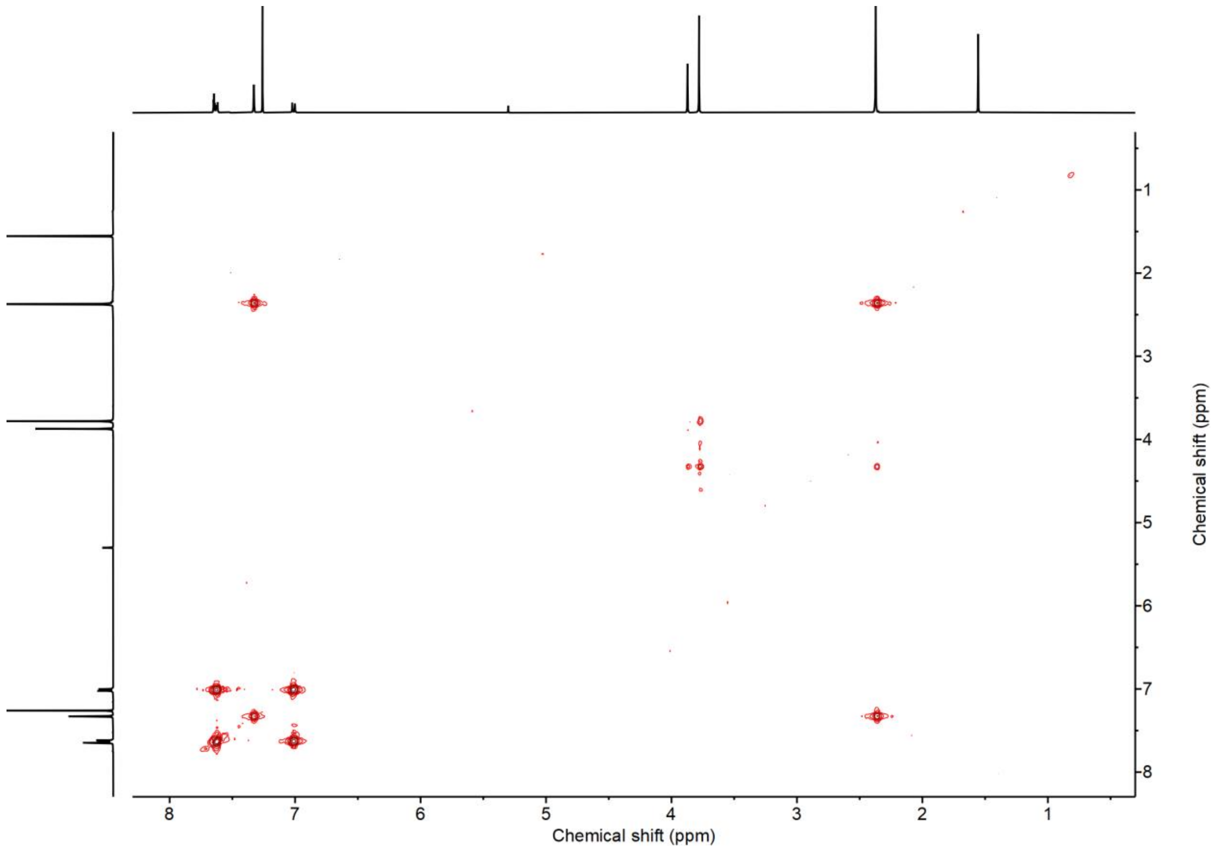

Figure S44 COSY NMR of 5^{ABB} (400 MHz, $CDCl_3$)

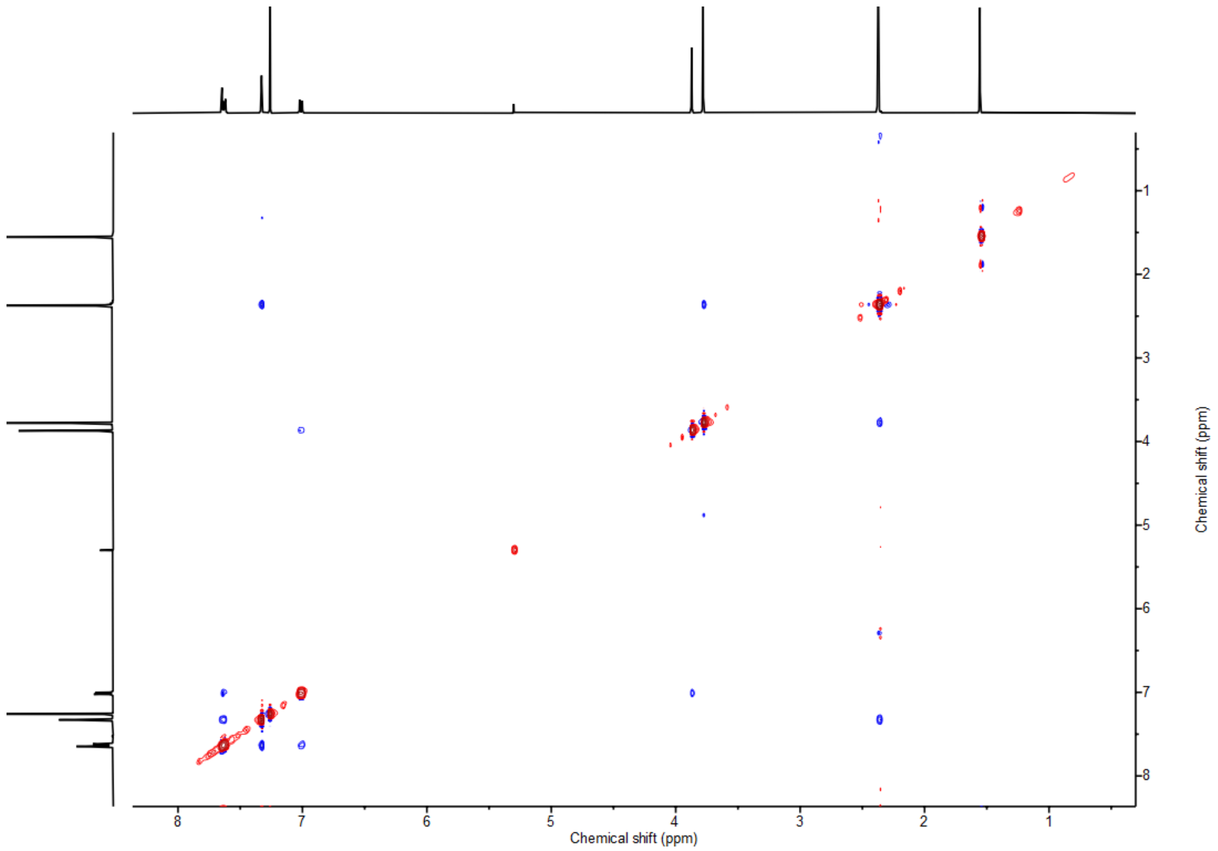

Figure S45 NOESY NMR of 5^{ABB} (400 MHz, $CDCl_3$)

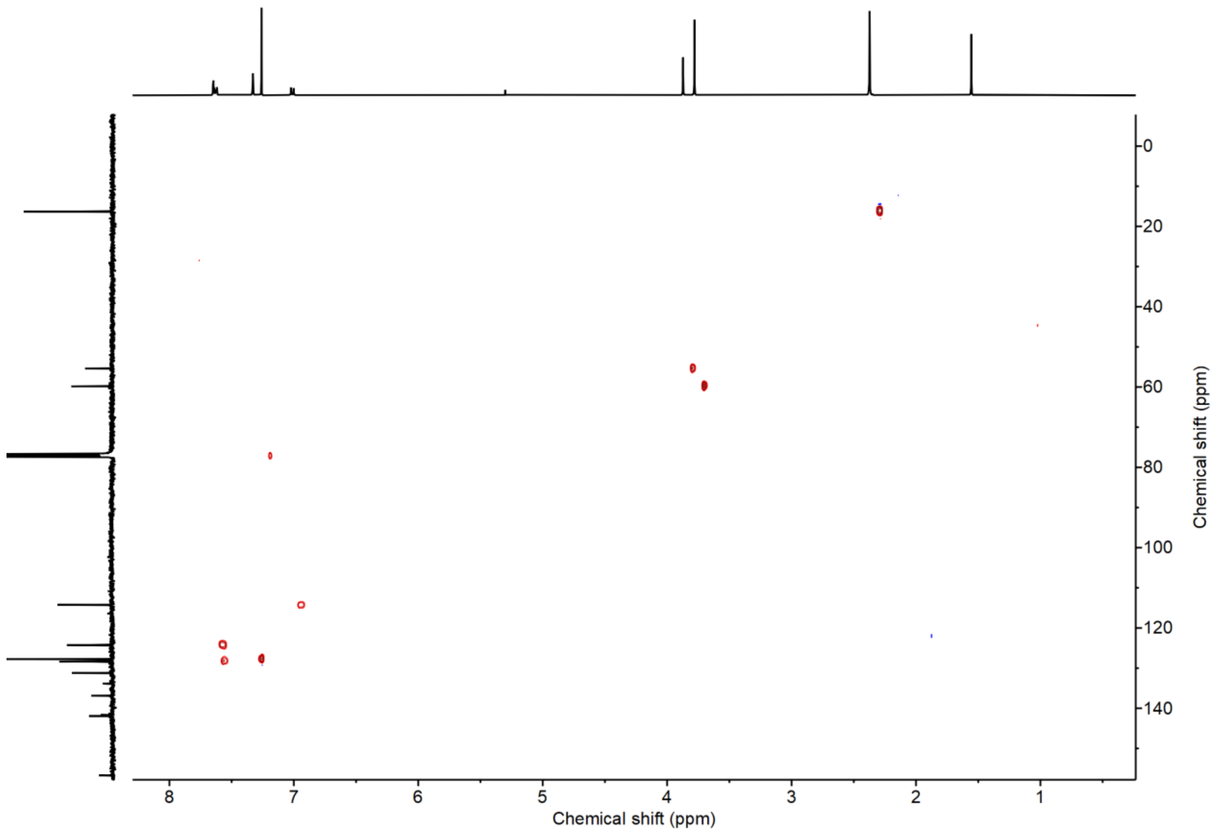

Figure S46 HSQC NMR of 5^{ABB} ($CDCl_3$)

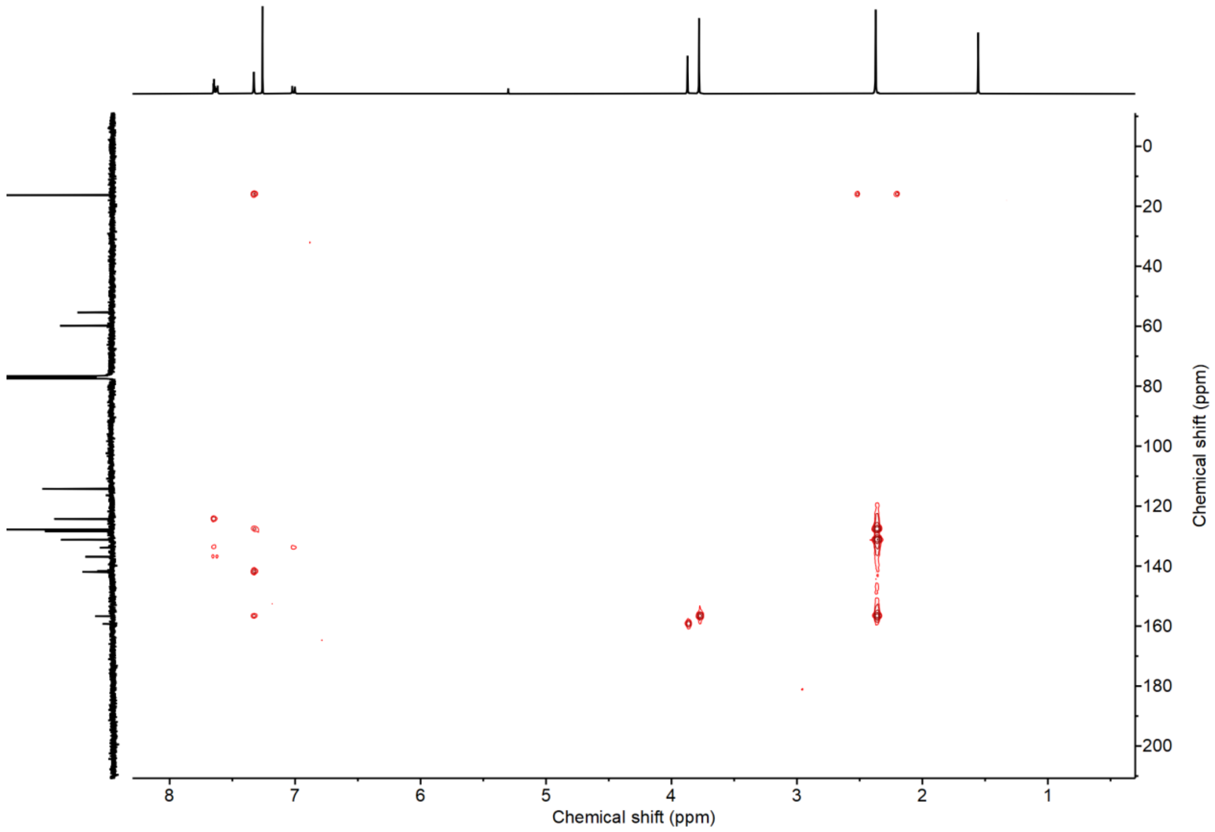

Figure S47 HMBC NMR of 5[ABB] ($CDCl_3$)

Synthesis of S1^{ABB}

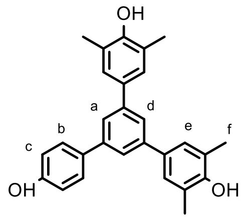

A solution of **5^{ABB}** (0.160 g, 0.175 mmol, 1 eq.) in acetic acid (5 mL) was heated at 110 °C in a reflux apparatus connected to a Dreschel flask containing sat. aq. $NaHCO_3$. 48% $HBr_{(aq)}$ (0.40 mL, 4.5 mmol, 9.0 eq.) was added dropwise and the reaction stirred for 30 h. To the cooled reaction mixture was added ice water (15 mL). The resultant white precipitate was collected by filtration, washed with H_2O (3 × 10 mL) and dried in air. The off-white solid (0.159 g) was taken forward without further purification.

^{1}H NMR (400 MHz, CD_3OD, 298 K) δ: 7.60 – 7.50 (m, 5H, H_a, H_d, H_b), 7.28 (s, 4H, H_e), 6.89 (d, J = 8.6 Hz, 2H, H_c), 2.30 (s, 12H, H_f).

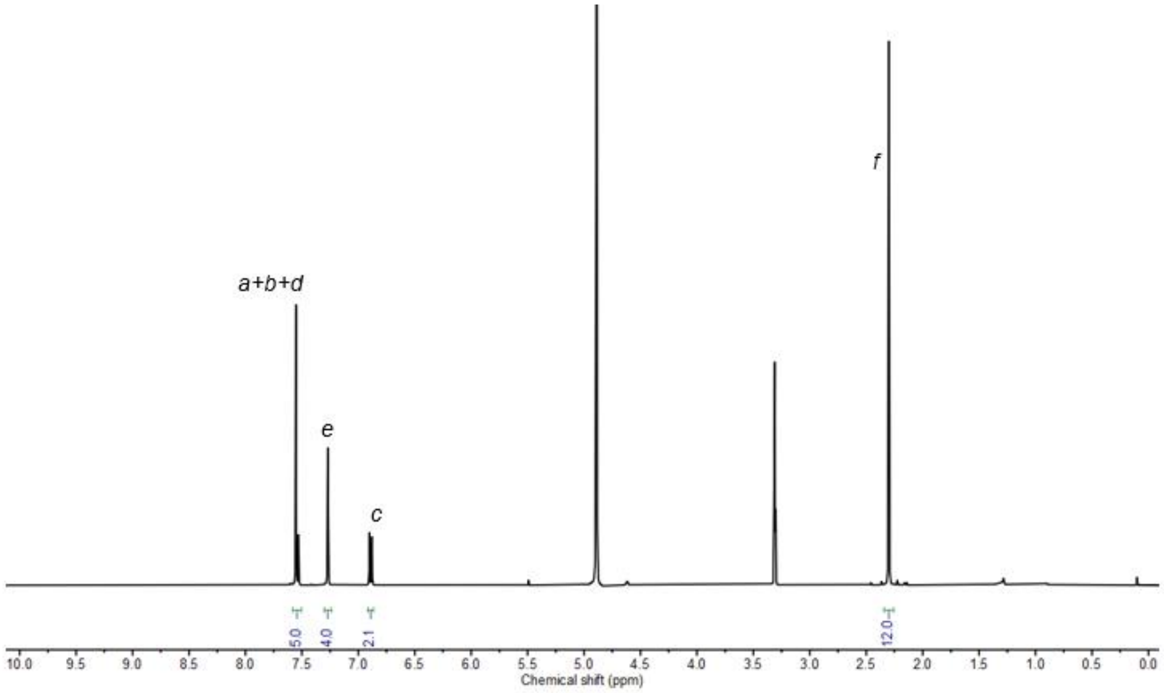

Figure S48 ^{1}H NMR of S1^{ABB} (400 MHz, CD_3OD)

Synthesis of 2^{ABB}

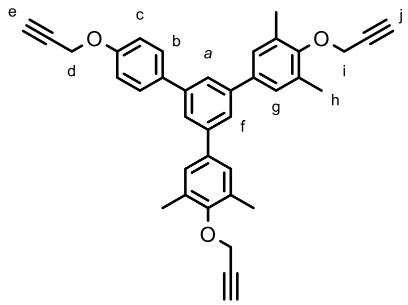

Crude **S1^{ABB}** (0.145 g) and K_2CO_3 (0.600 g, 5.3 mmol, 10 eq.) were suspended in dry MeCN (10 mL) and stirred at 80 °C. Propargyl bromide solution (80% in toluene, 0.15 mL, 1.40 mmol, 4.5 eq.) was added to this suspension and allowed to react for 16 h. To the cooled reaction mixture H_2O (10 mL) was added and aqueous phase extracted with CH_2Cl_2 (3 × 10 mL). The combined organic phases were washed with brine (2 × 10 mL), dried ($MgSO_4$) and the solvent removed *in vacuo*. After purification by column chromatography on silica gel (1:4 hexane/CH_2Cl_2) the product was obtained as a white solid (0.0139 g, 82% over 2 steps).

^{1}H NMR (400 MHz, $CDCl_3$, 298 K) δ: 7.68 – 7.60 (m, 5H, H_a, H_f, H_b), 7.33 (s, 4H, H_g), 7.09 (d, J = 8.8 Hz, 2H, H_c), 4.76 (d, J = 2.4 Hz, 2H, H_d), 4.56 (d, J = 2.4 Hz, 4H, H_i), 2.59 – 2.52 (m, 3H, H_e, H_j), 2.41 (s, 12H, H_h).

^{13}C NMR (101 MHz, $CDCl_3$) δ: 157.25, 155.02, 141.81, 141.55, 137.38, 134.70, 131.57, 128.39, 127.80, 124.45, 124.40, 115.25, 79.33, 78.54, 75.66, 75.11, 59.94, 55.92, 16.76.

HR-ESI-MS *m/z* = 525.2433 $[M+H]^+$ calc. 525.2430.

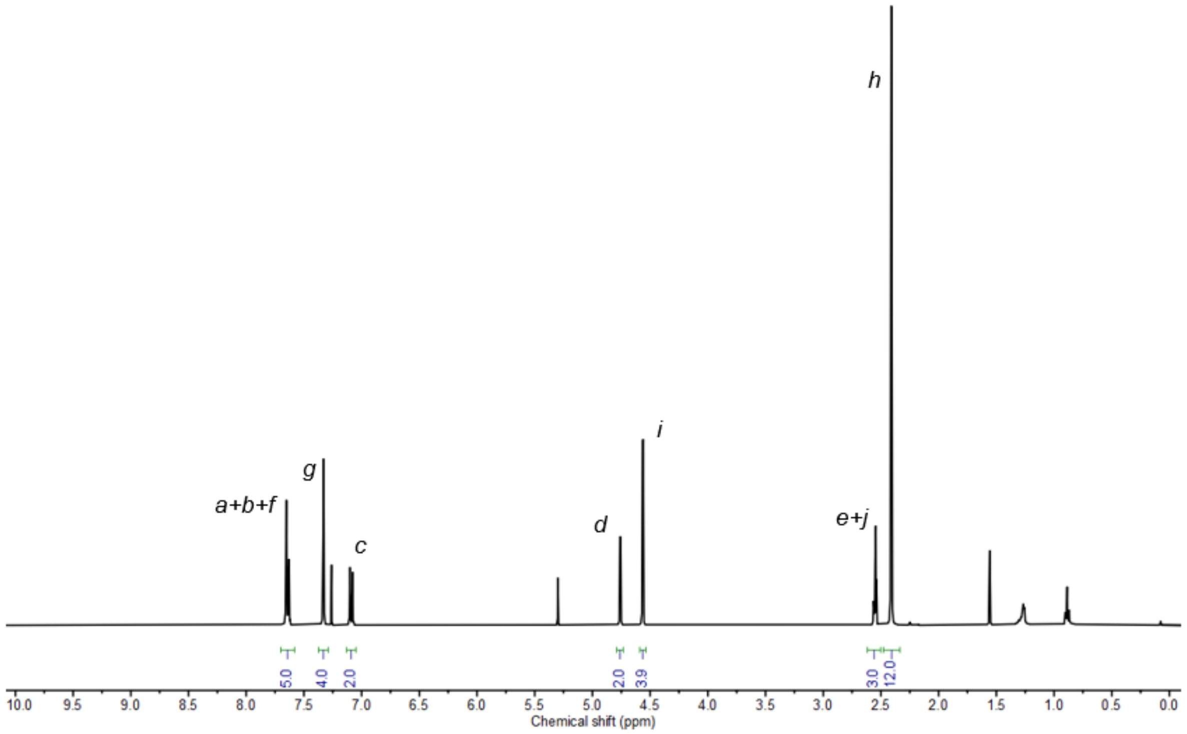

Figure S49 ^{1}H NMR of 2^{ABB} (400 MHz, $CDCl_3$)

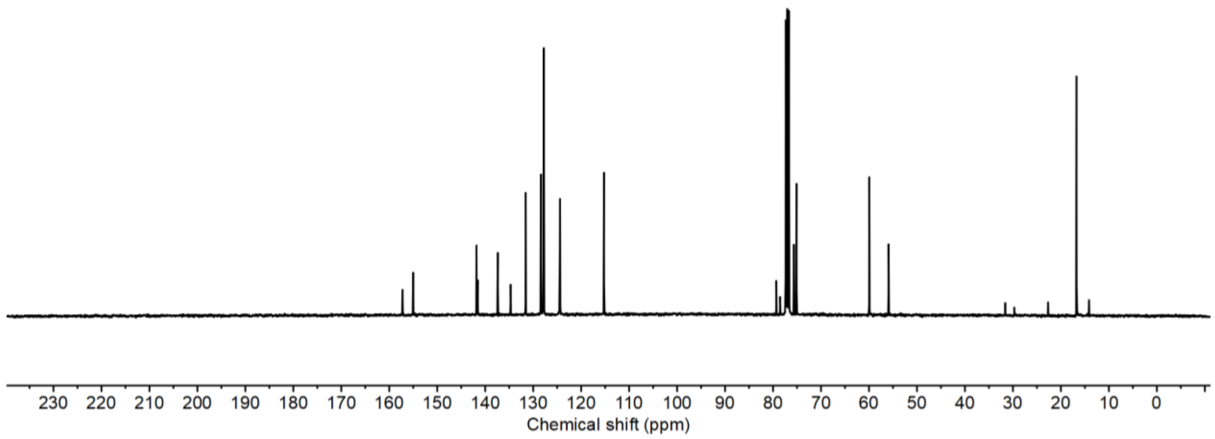

Figure S50 ^{13}C NMR of 2^{ABB} (101 MHz, $CDCl_3$)

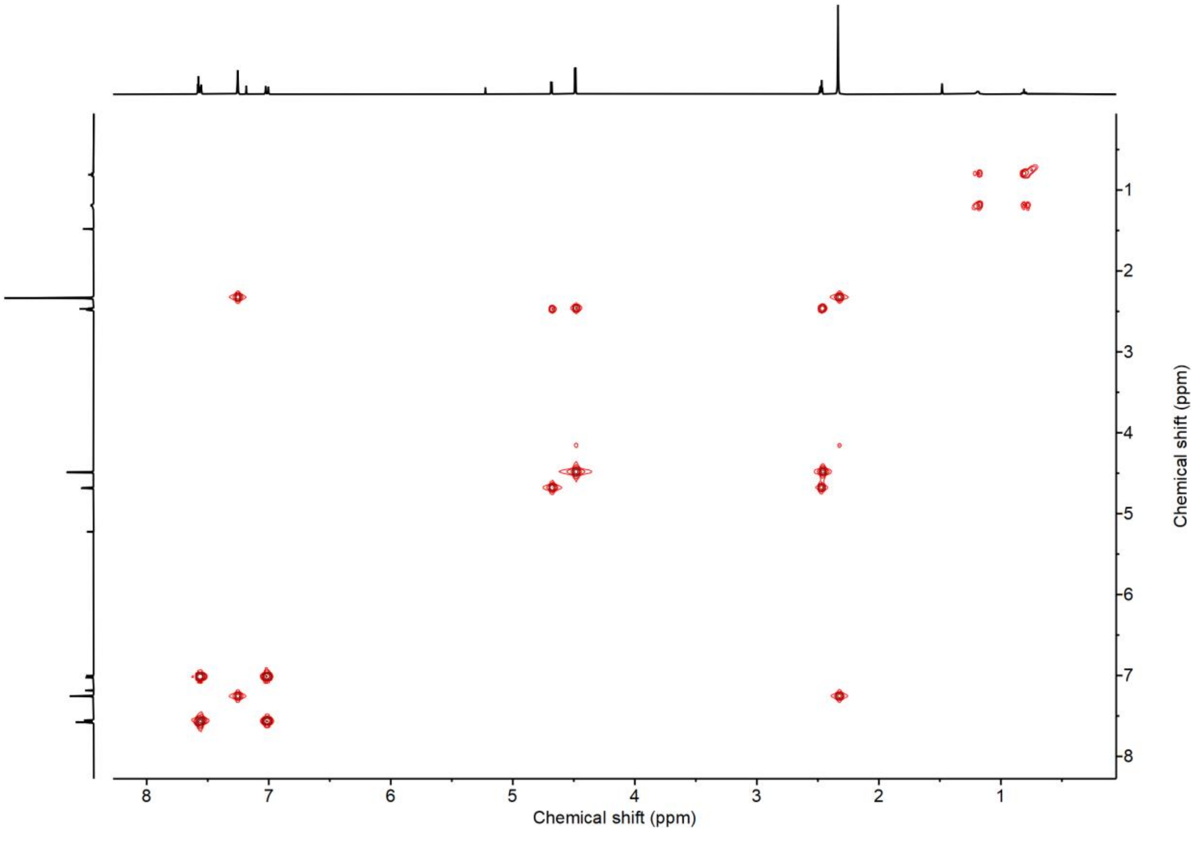

Figure S51 COSY NMR of 2^{ABB} (400 MHz, $CDCl_3$)

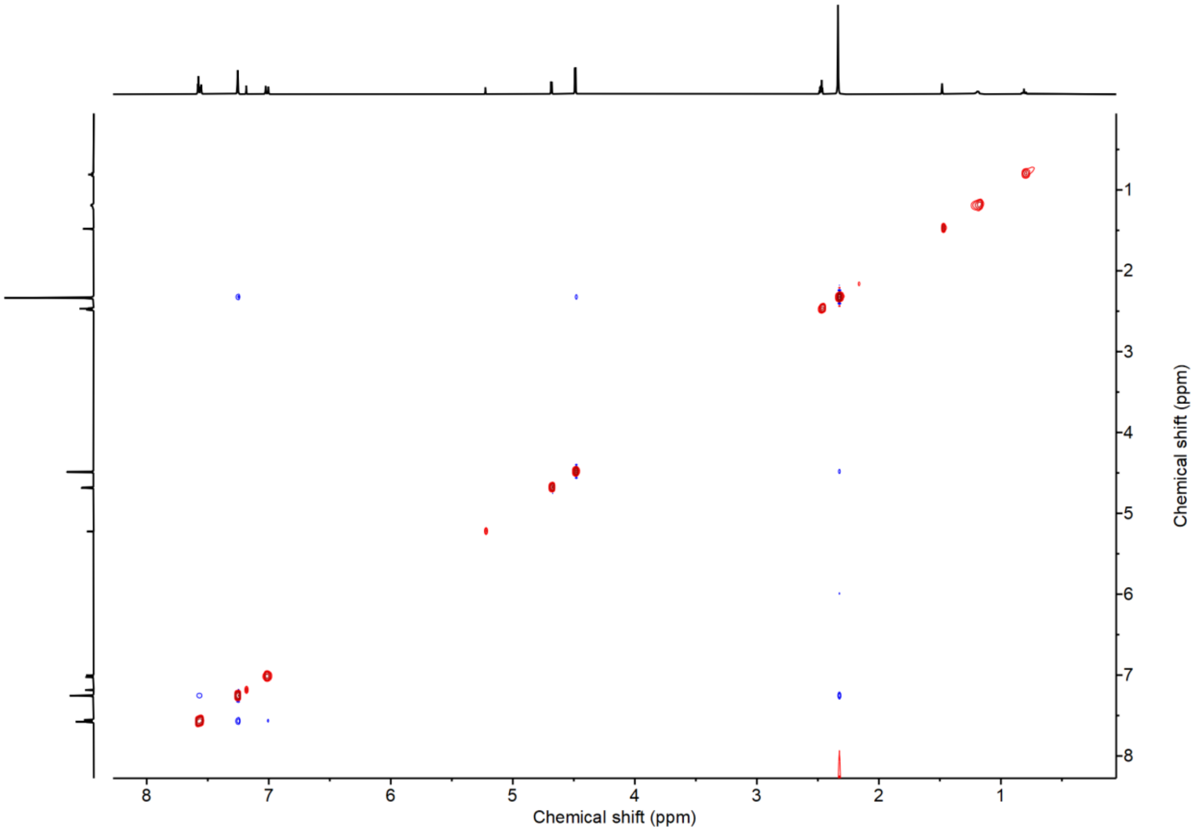

Figure S52 NOESY NMR of 2ABB (400 MHz, $CDCl_3$)

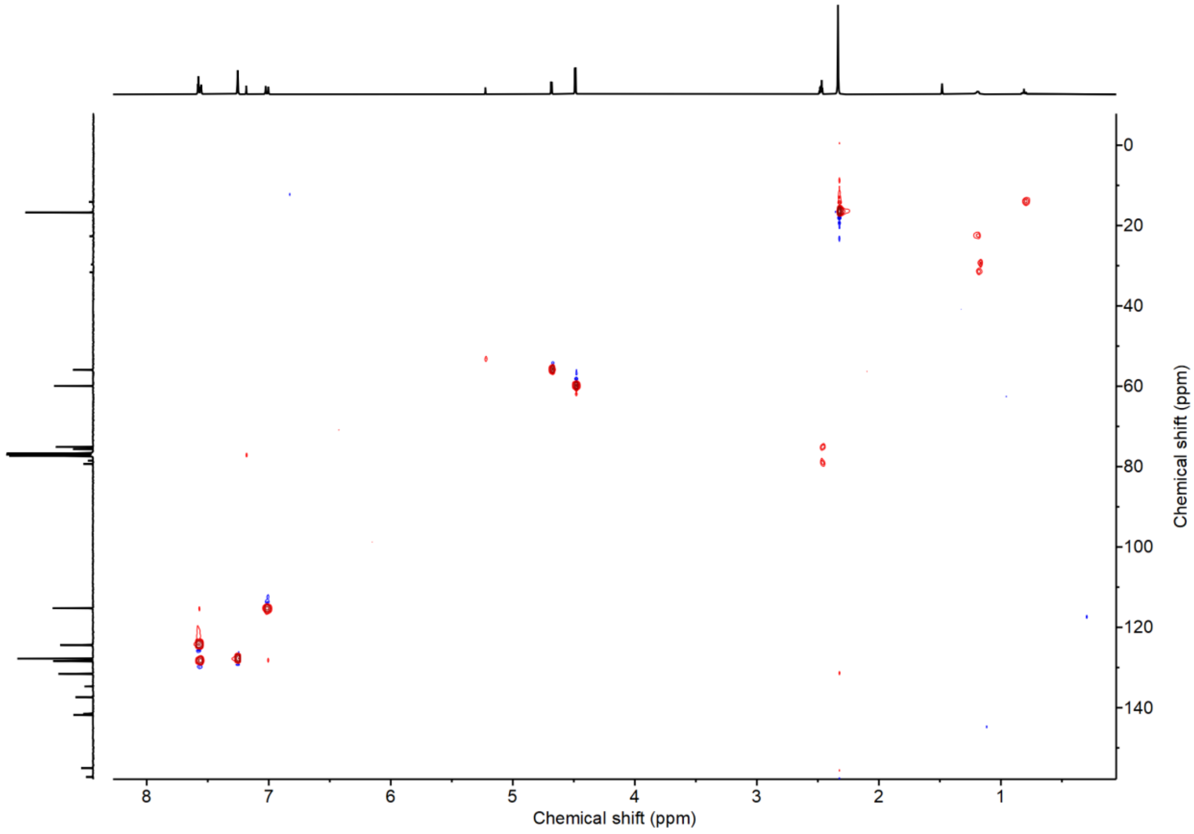

Figure S53 HSQC NMR of 2ABB ($CDCl_3$)

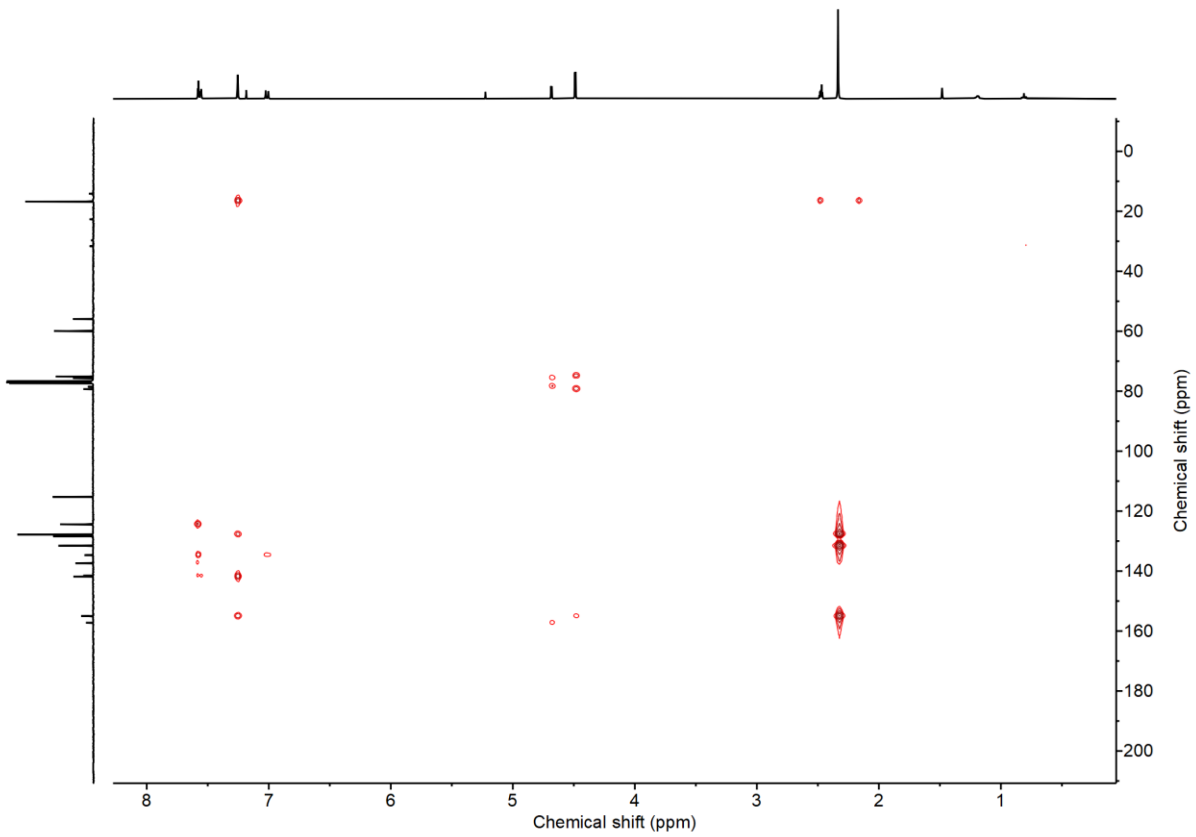

Figure S54 HMBC NMR of 2^{ABB} (CDCl$_3$)

Synthesis of 5^{AB}

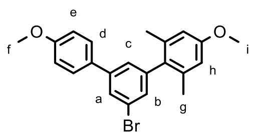

5^{A} (0.349 g, 1.0 mmol, 1 eq.), 2,6-dimethyl-4-methoxybenzene boronic acid (0.184 g, 1 mmol, 1 eq.), $Pd(PPh_3)_2Cl_2$ (0.037 g, 0.05 mmol, 5 mol%), and K_2CO_3 (0.350 g, 2.5 mmol, 2.5 eq.) were stirred at 110 °C in 1:3 H_2O/dioxane (degassed, 4 mL) for 24 h. To the cooled reaction mixture was added H_2O (60 mL) and the aqueous phase extracted with CH_2Cl_2 (3 × 25 mL). The combined organic phases were washed with brine (2 × 25 mL), dried ($MgSO_4$) and the solvent removed *in vacuo*. After purification by column chromatography on silica gel (9:1 hexane/CH_2Cl_2) the product was obtained as a white solid (0.171 g, 42%).

^{1}H NMR (400 MHz, $CDCl_3$, 298 K) δ: 7.67 (t, *J* = 1.8 Hz, 1H, H_a), 7.52 (d, *J* = 8.9 Hz, 2H, H_d), 7.27 (t, *J* = 1.6 Hz, 1H, H_c), 7.23 (t, *J* = 1.7 Hz, 1H, H_b), 6.98 (d, *J* = 8.9 Hz, 2H, H_e), 6.68 (s, 2H, H_h), 3.85 (s, 3H, H_f), 3.83 (s, 3H, H_i), 2.07 (s, 6H, H_g).

^{13}C NMR (101 MHz, $CDCl_3$, 298 K) δ: 161.3, 131.9, 130.8, 128.5, 128.2, 123.2, 114.5, 55.4.

HR-ESI-MS *m/z* = 399.0778 $[M+H]^+$ calc. 399.0781.

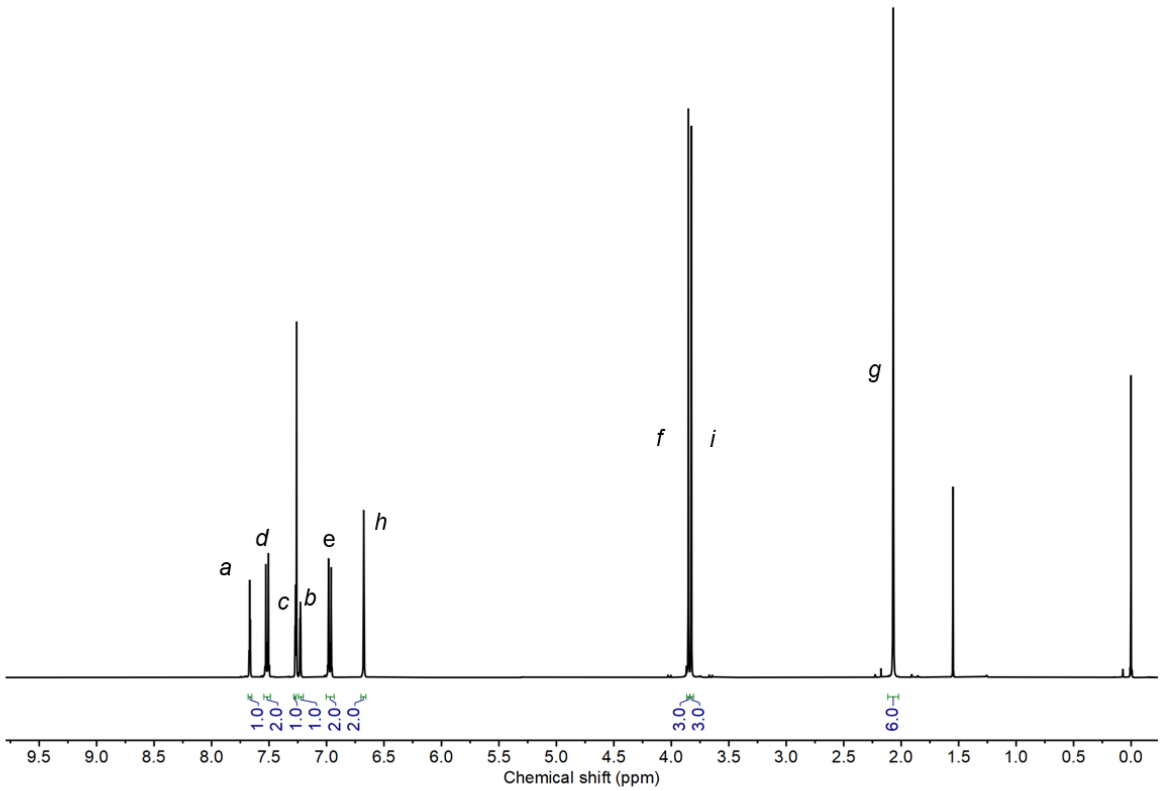

Figure S55 ^{1}H NMR of 5^{AB} (400 MHz, $CDCl_3$)

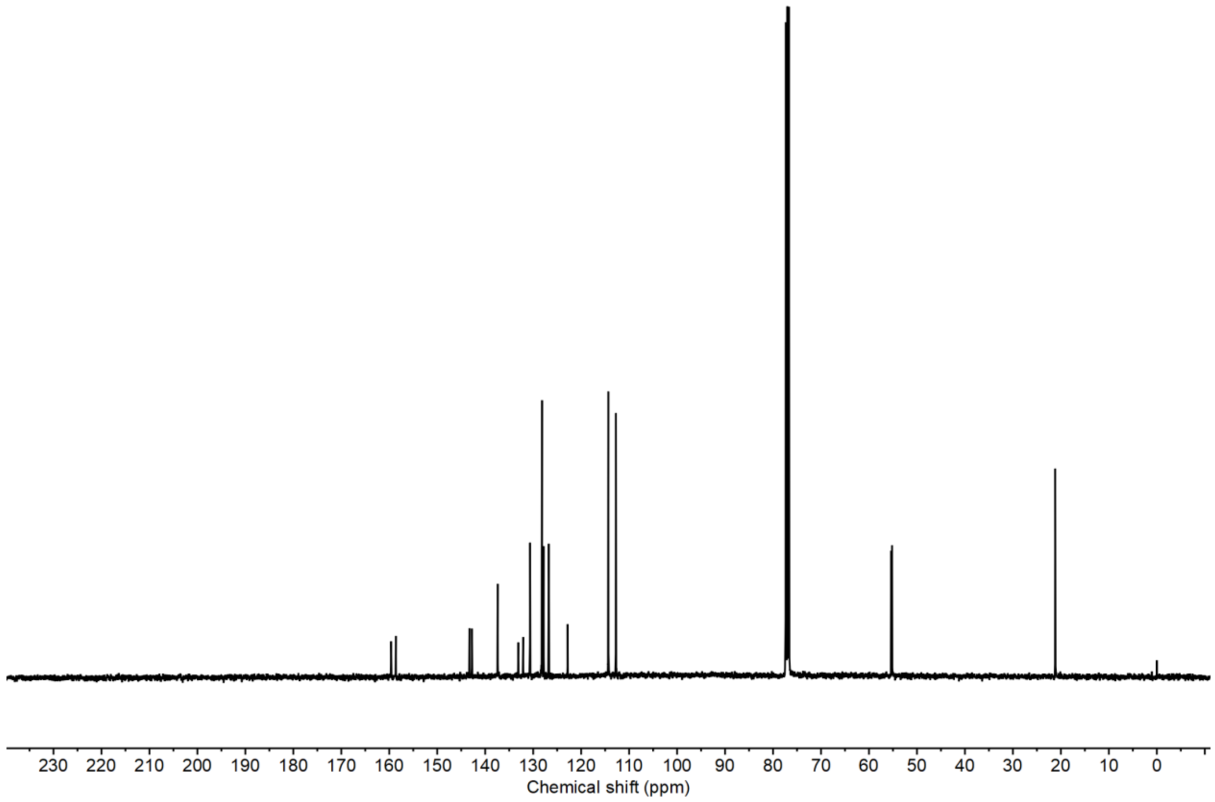

Figure S56 ^{13}C NMR of 5^{AB} (101 MHz, $CDCl_3$)

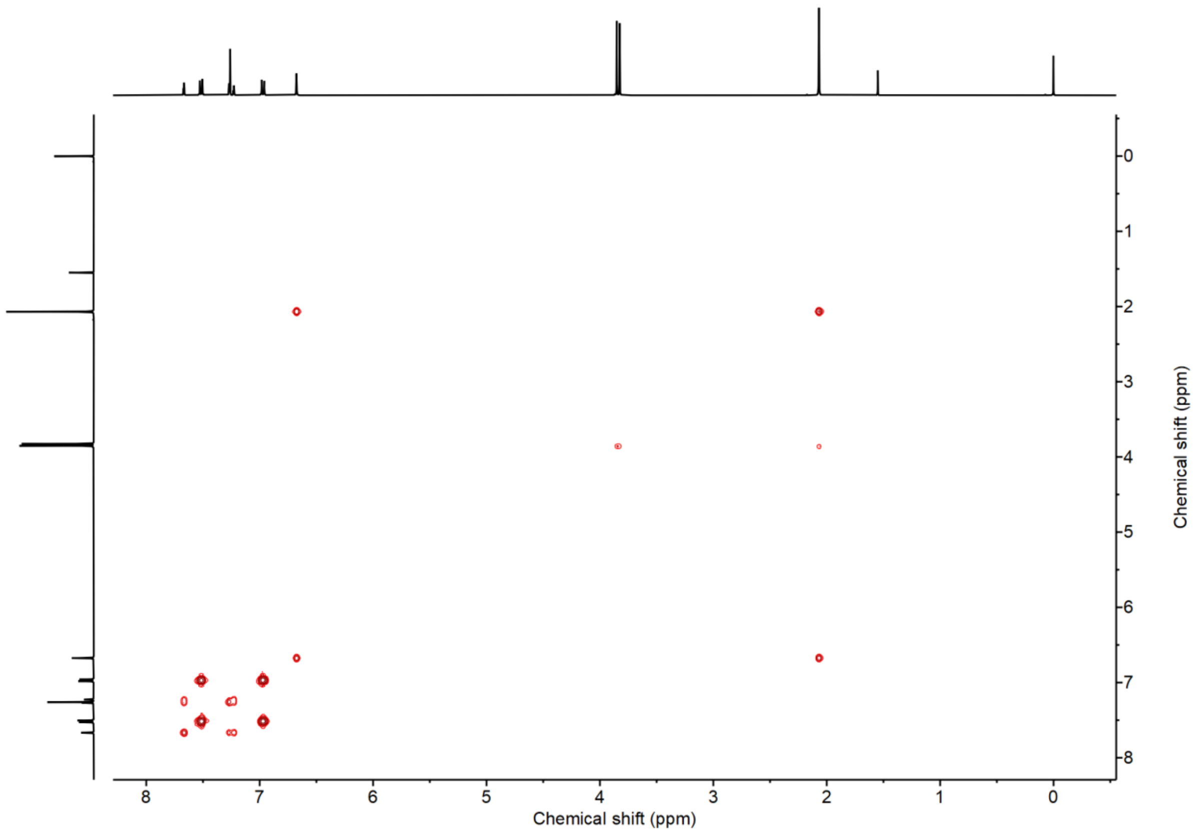

Figure S57 COSY NMR of 5^{AB} (400 MHz, $CDCl_3$)

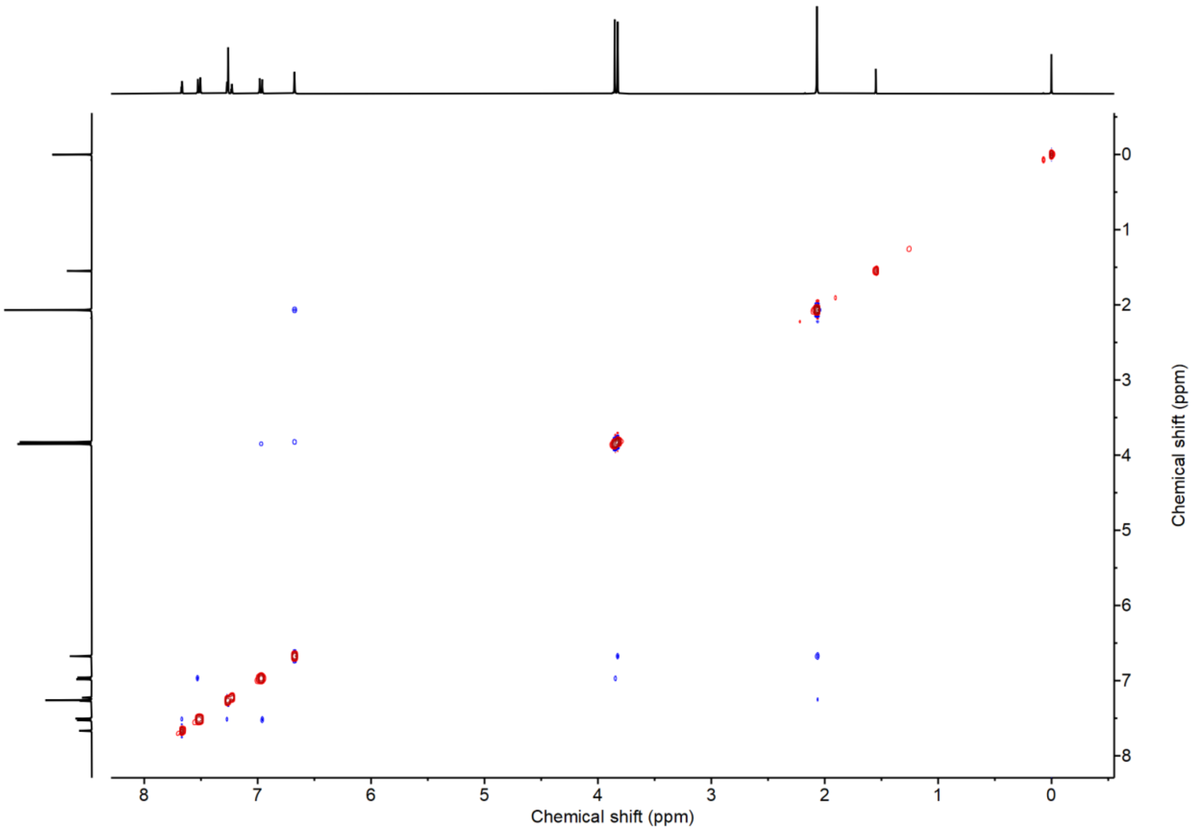

Figure S58 NOESY NMR of 5[AB] (400 MHz, $CDCl_3$)

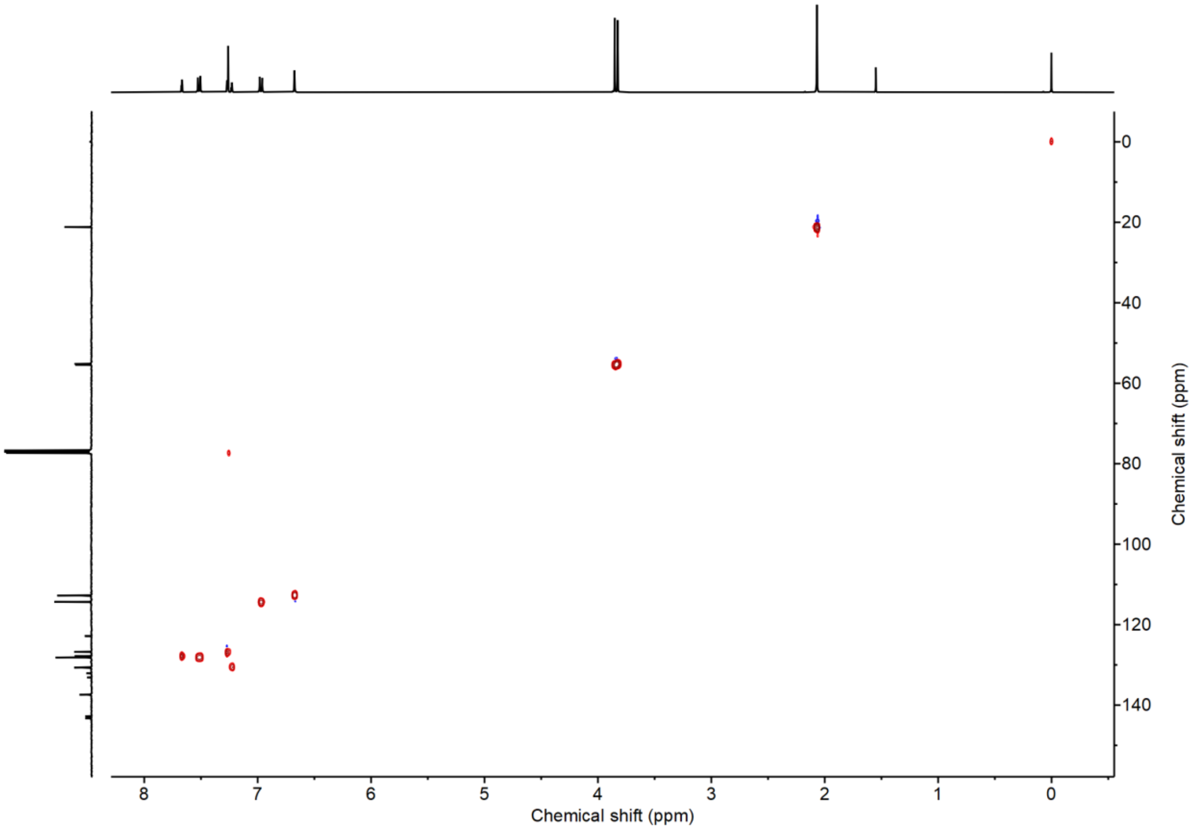

Figure S59 HSQC NMR of 5[AB] ($CDCl_3$)

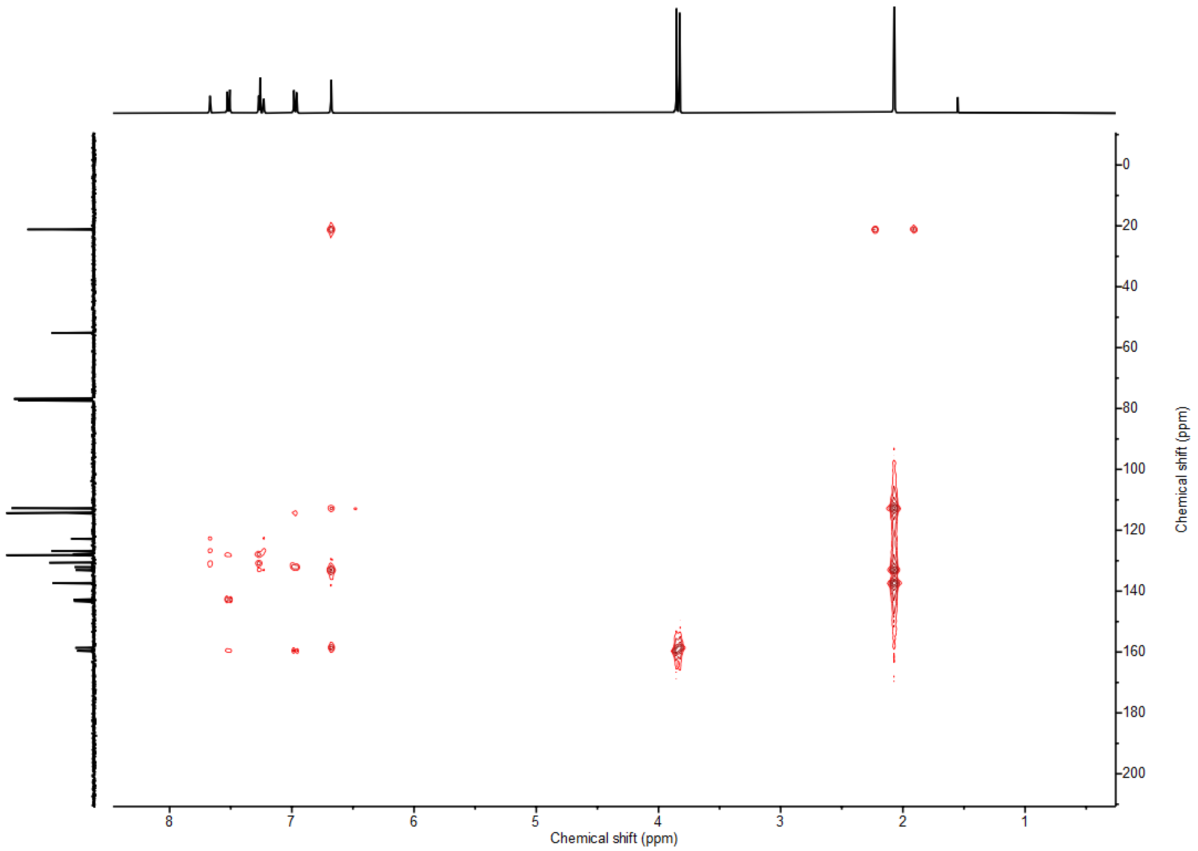

Figure S60 HMBC NMR of 5[AB] ($CDCl_3$)

Synthesis of 5^{ABC}

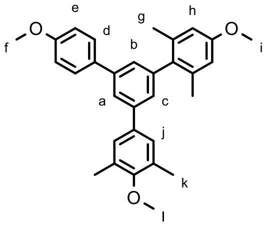

5^{AB} (0.099 g, 0.25 mmol, 1 eq.), 4-methoxy-3,5-dimethylphenyl)boronic acid (0.056 g, 0.33 mmol, 1.3 eq.), $Pd(PPh_3)_2Cl_2$ (0.0090 g, 0.013 mmol), and K_2CO_3 (0.090 g, 0.625 mmol 0.25 eq.) were stirred at 110 °C in 1:3 H_2O/dioxane (degassed, 4 mL) for 24 h. To the cooled reaction mixture was added H_2O (10 mL) and the aqueous phase extracted with CH_2Cl_2 (3 × 10 mL). The combined organic phases were washed with brine (2 × 20 mL), dried ($MgSO_4$) and the solvent removed *in vacuo*. After purification by column chromatography on silica gel (3:2 hexane/CH_2Cl_2) the product was obtained as a white solid (0.1062 g, 95%).

^{1}H NMR (400 MHz, $CDCl_3$, 298 K) δ: 7.69 (t, *J* = 1.8 Hz, 1H, H_a), 7.61 (d, *J* = 8.9 Hz, 2H, H_d), 7.32 (s, 2H, H_j), 7.30 – 7.27 (m, 2H, H_b, H_c), 6.99 (d, 2H, *J* = 8.9 Hz, H_e), 6.70 (s, 2H, H_h), 3.86 (s, 3H, H_f), 3.84 (s, 3H, H_i), 3.77 (s, 3H, H_l), 2.35 (s, 6H, H_k), 2.11 (s, 6H, H_g).

^{13}C NMR (101 MHz, $CDCl_3$, 298 K) δ: 159.2, 158.4, 141.4, 141.2, 137.6, 136.7, 134.5, 131.2, 128.2, 127.6, 126.6, 126.5, 123.5, 114.2, 112.7, 59.8, 55.4, 55.2, 21.3, 16.3.

HR-ESI-MS *m/z* = 453.2430 $[M+H]^+$ calc. 453.2430.

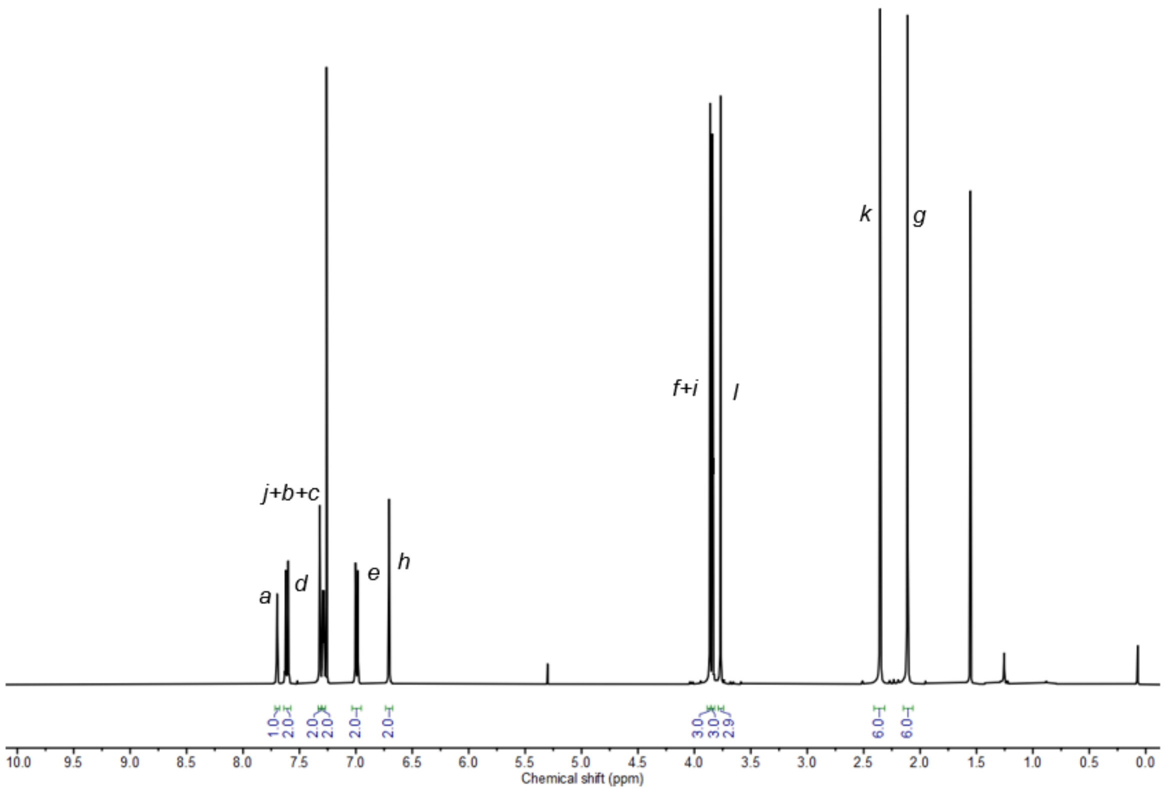

Figure S61 ^{1}H NMR of 5^{ABC} (400 MHz, $CDCl_3$)

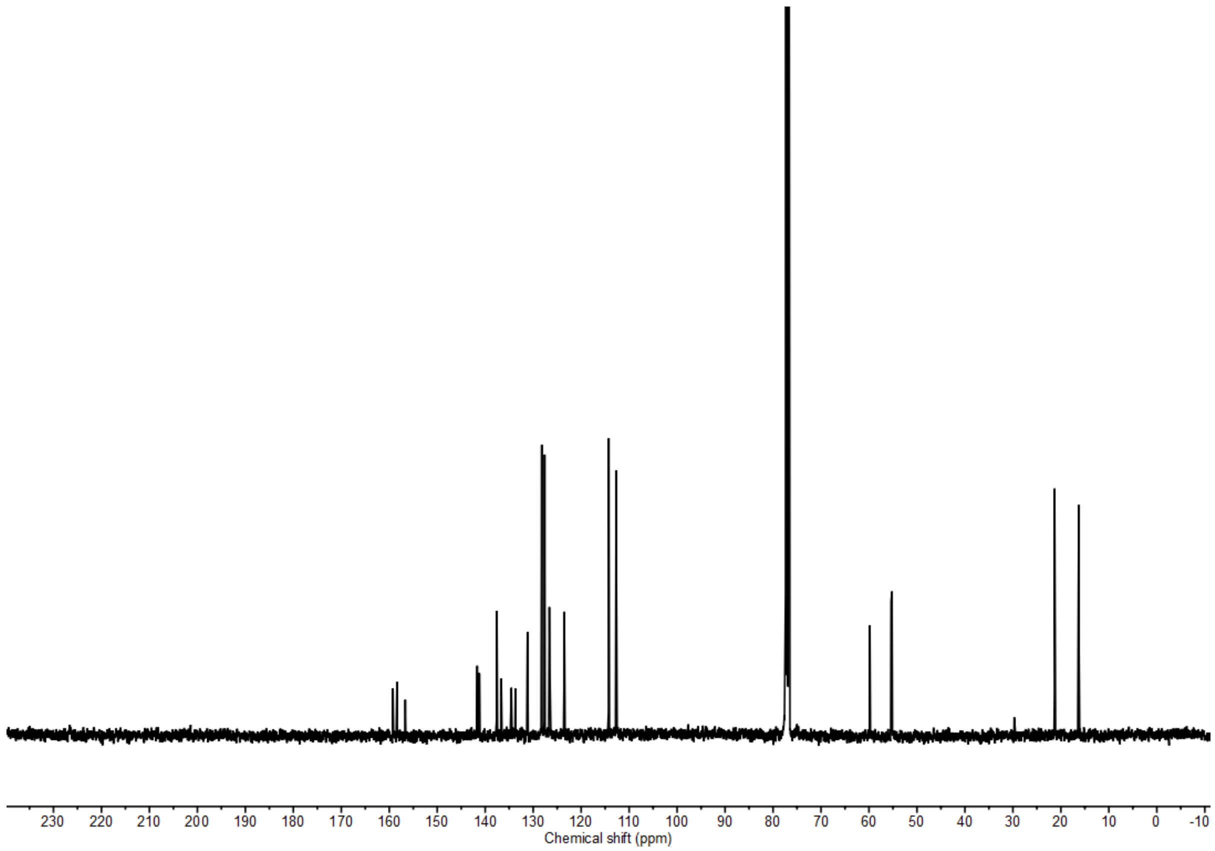

Figure S62 ^{13}C NMR of 5^{ABC} (101 MHz, $CDCl_3$)

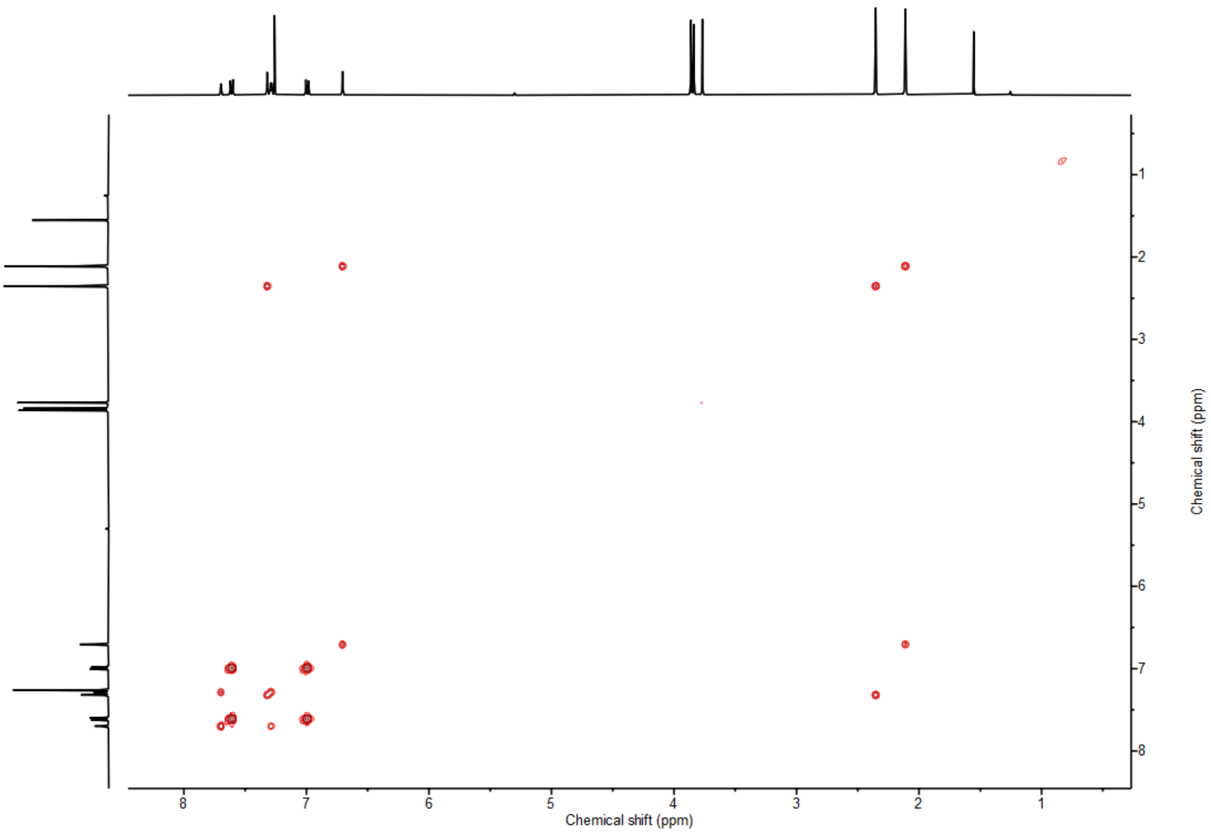

Figure S63 COSY NMR of 5^{ABC} (400 MHz, $CDCl_3$)

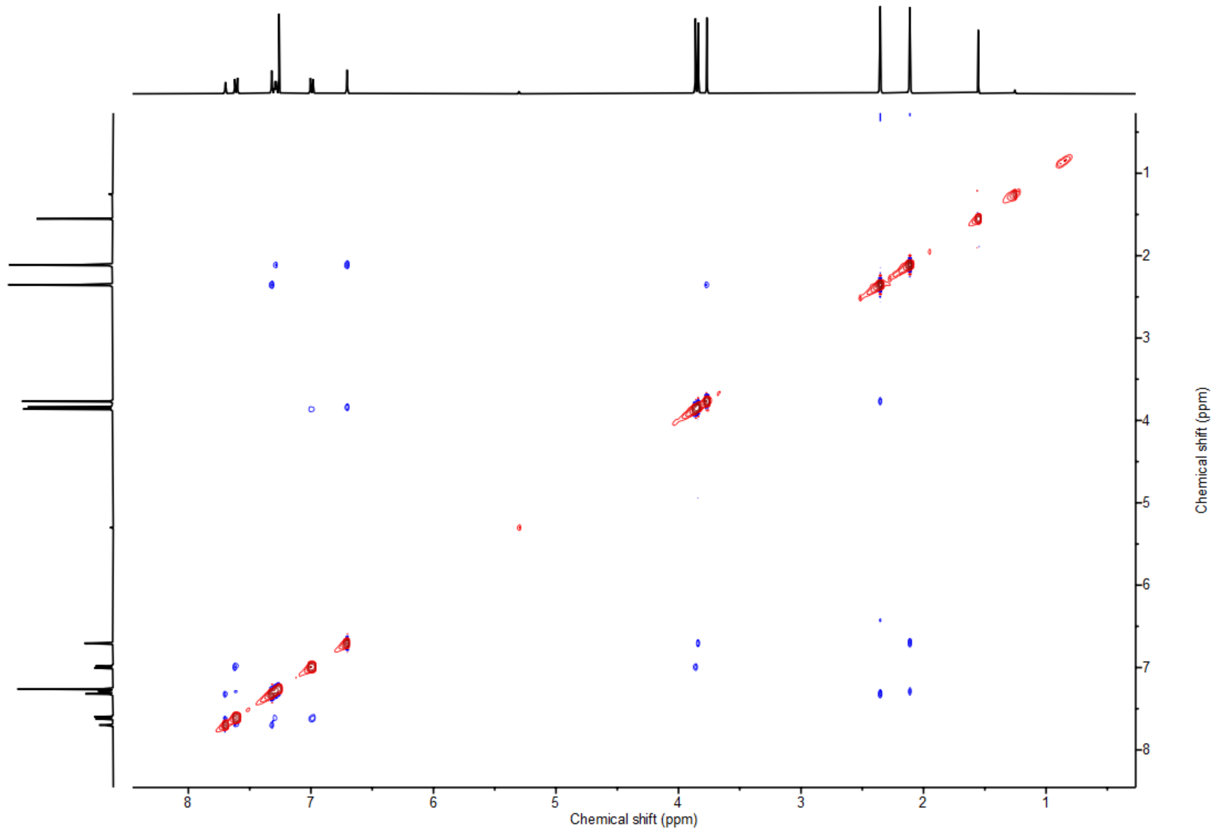

Figure S64 NOESY NMR of 5ABC (400 MHz, $CDCl_3$)

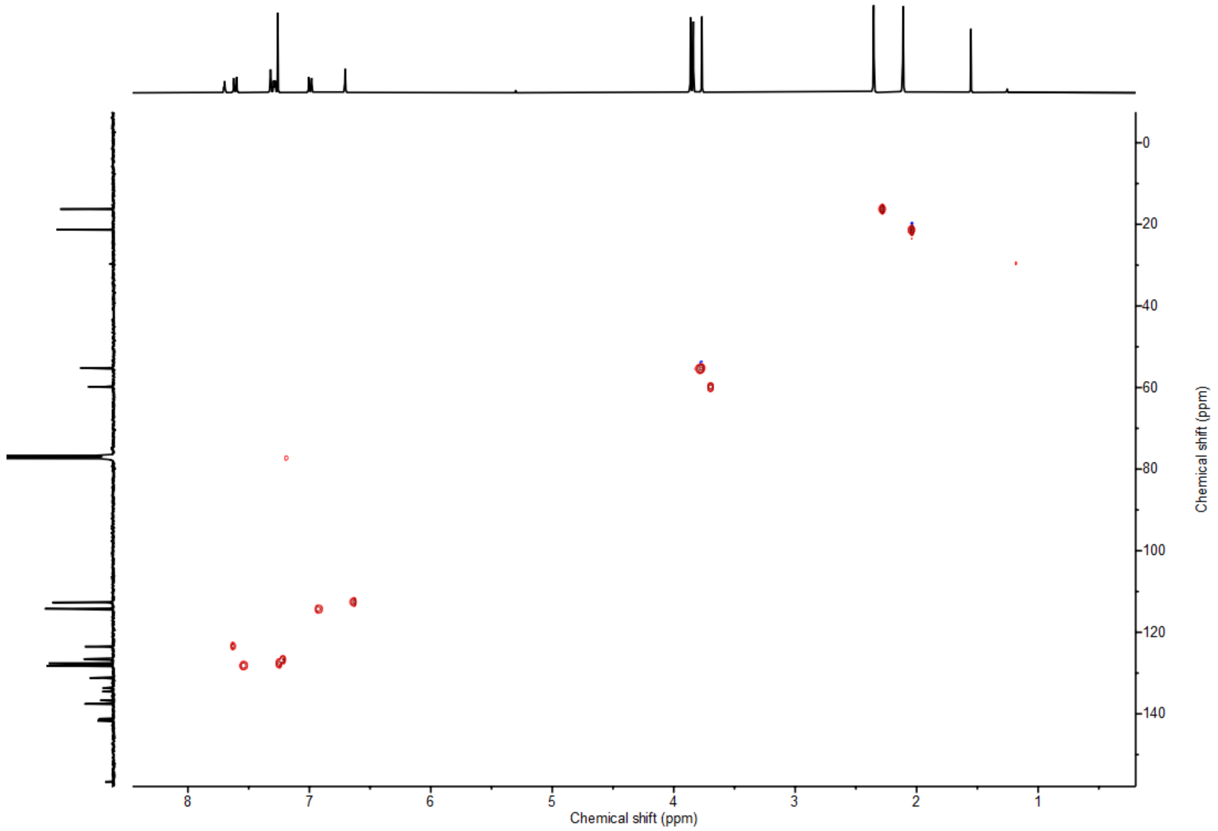

Figure S65 HSQC NMR of 5ABC ($CDCl_3$)

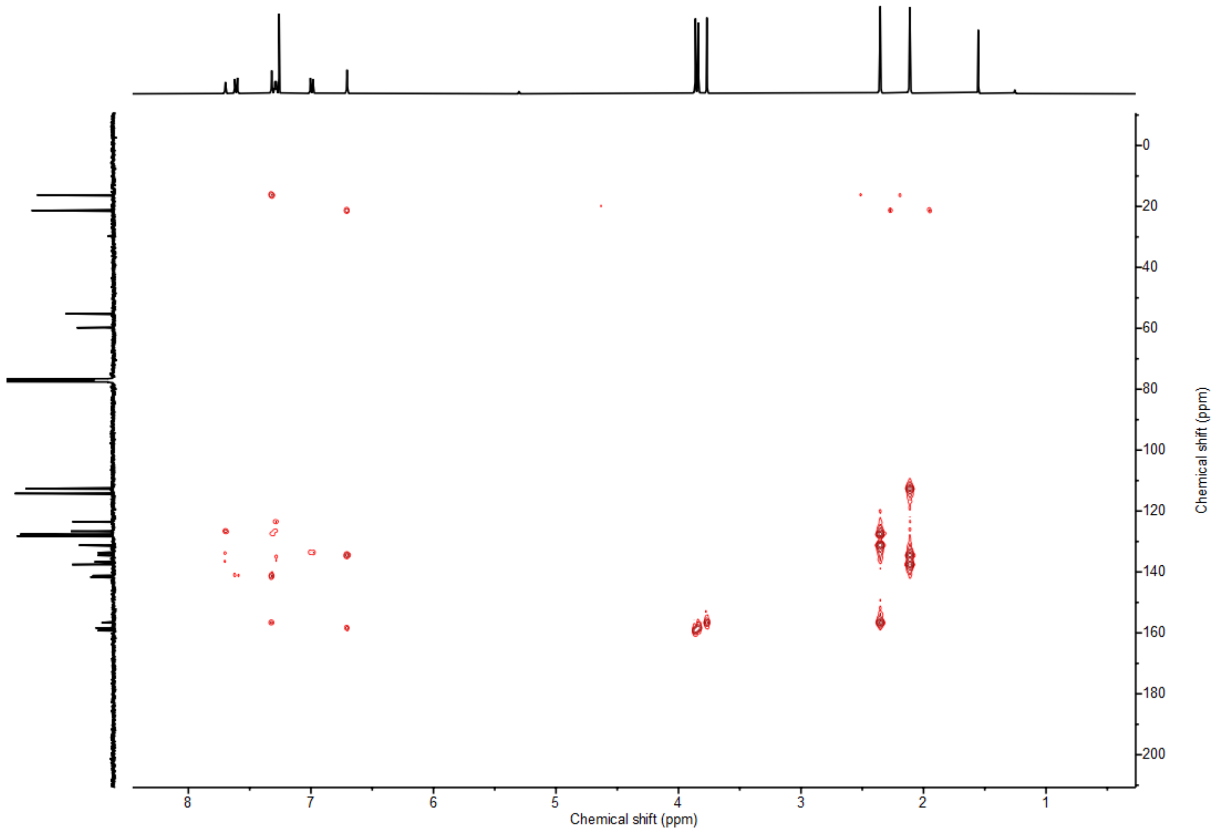

Figure S66 HMBC NMR of 5^{ABC} ($CDCl_3$)

Synthesis of $S1^{ABC}$

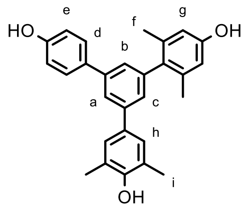

A solution of $\mathbf{5^{ABC}}$ (0.104 g, 0.175 mmol, 1 eq.) in acetic acid (5 mL) was heated at 110 °C in a reflux apparatus connected to a Dreschel flask containing sat. aq. $NaHCO_3$. 48% $HBr_{(aq)}$ (0.27 mL, 4.5 mmol, 9.0 eq.) was added dropwise and the reaction stirred for 30 h. To the cooled reaction mixture was added ice water (15 mL). The resultant white precipitate was collected by filtration, washed with H_2O (3 × 10 mL) and dried in air. The off-white solid (0.0928 g) was taken forward without further purification.

^{1}H NMR (400 MHz, $CDCl_3$, 298 K) δ: 7.67 (t, *J* = 1.8 Hz, 1H, H_a), 7.56 (d, *J* = 8.8 Hz, 2H, H_d), 7.29 (s, 2H, H_h), 7.26 – 7.23 (m, 2H, H_b, H_c), 6.92 (d, *J* = 8.8 Hz, 2H, H_e), 6.63 (s, 2H, H_g), 2.29 (s, 6H, H_f), 2.08 (s, 6H, H_i).

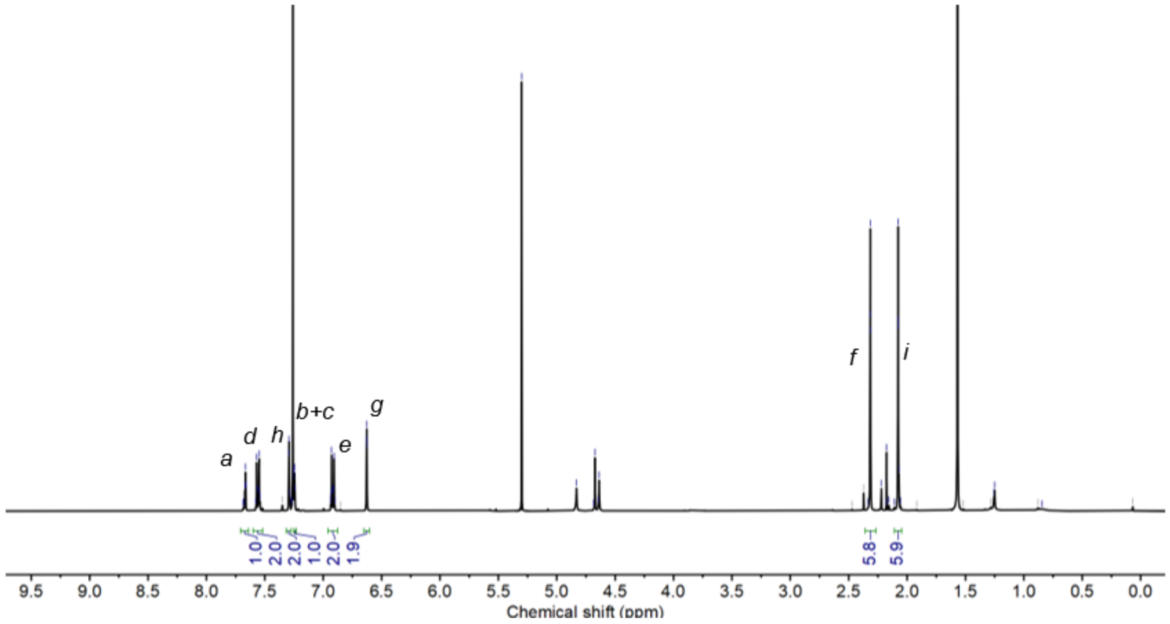

Figure S67 ^{1}H NMR of $S1^{ABC}$ (400 MHz, $CDCl_3$)

Synthesis of 2^{ABC}

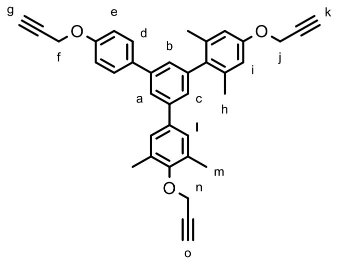

Crude **$S1^{ABC}$** (0.0928 g.) and K_2CO_3 (0.33 g, 2.4 mmol, 10 eq.) was suspended in dry MeCN (4 mL) and stirred at 80 °C. Propargyl bromide solution (80% in toluene, 0.12 mL, 1.0 mmol, 4.0 eq.) was added to this suspension and allowed to react for 16 h. To the cooled reaction mixture H_2O (20 mL) was added and the aqueous phase extracted with CH_2Cl_2 (3 × 20 mL). The combined organic phases were washed with brine (2 × 20 mL), dried ($MgSO_4$) and the solvent removed *in vacuo*. After purification by column chromatography on silica gel (4:1 hexane/CH_2Cl_2) the product was obtained as a white solid (0.094 g, 79% over 2 steps).

^{1}H NMR (400 MHz, $CDCl_3$, 298 K) δ: 7.70 (t, *J* = 1.8 Hz, 1H, H_a), 7.62 (d, *J* = 8.8 Hz, 2H, H_d), 7.32 (s, 2H, H_l), 7.30 – 7.27 (m, 2H, H_b, H_c), 7.07 (d, *J* = 8.9 Hz, 2H, H_e), 6.77 (s, 2H, H_i), 4.75 (d, *J* = 2.4 Hz, 2H, H_f), 4.72 (d, *J* = 2.4 Hz, 2H, H_j), 4.55 (d, *J* = 2.5 Hz, 2H, H_n), 2.58 – 2.50 (m, 3H, H_g, H_h, H_o), 2.39 (s, 6H, H_m), 2.11 (s, 6H, H_h).

^{13}C NMR (101 MHz, $CDCl_3$, 298 K) δ: 156.4, 155.0, 141.3, 141.1, 137.6, 137.1, 135.3, 134.6, 131.6, 128.2, 127.6, 126.7, 123.7, 115.2, 113.6, 77.3, 77.0, 76.7, 75.6, 75.4, 75.1, 59.9, 55.9, 55.7, 21.3, 16.7.

HR-ESI-MS *m/z* = 525.2433 $[M+H]^+$ calc. 525.2430.

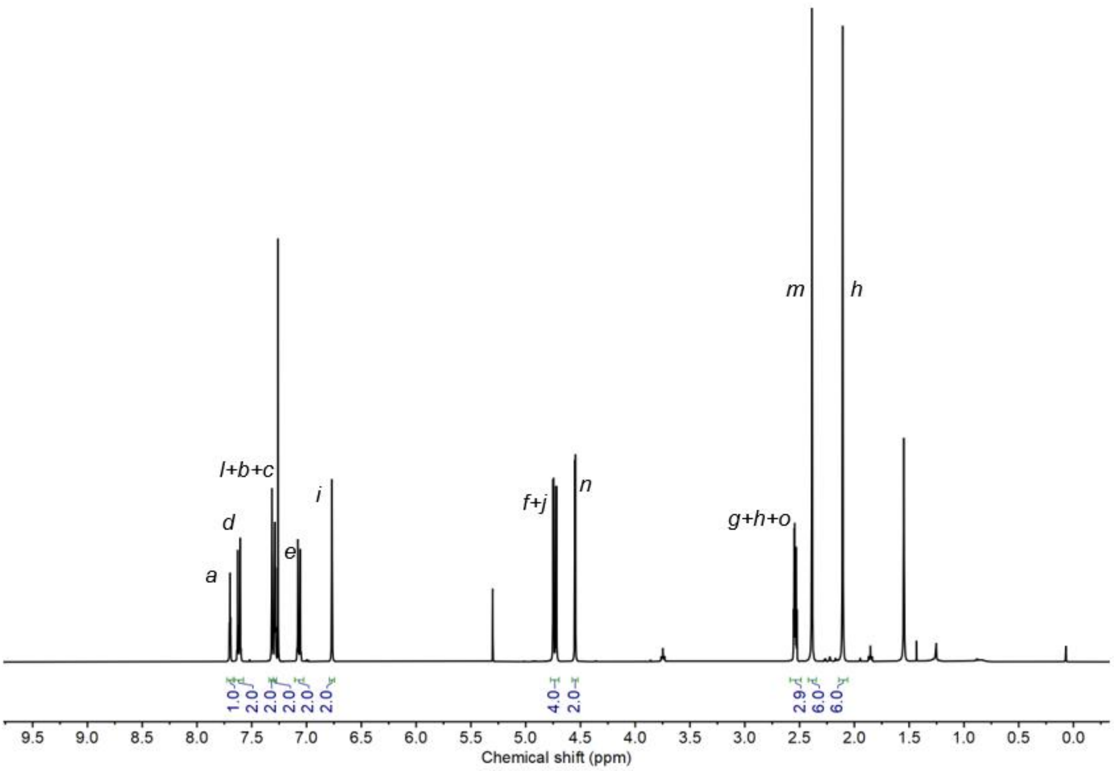

Figure S68 ^{1}H NMR of 2^{ABC} (400 MHz, $CDCl_3$)

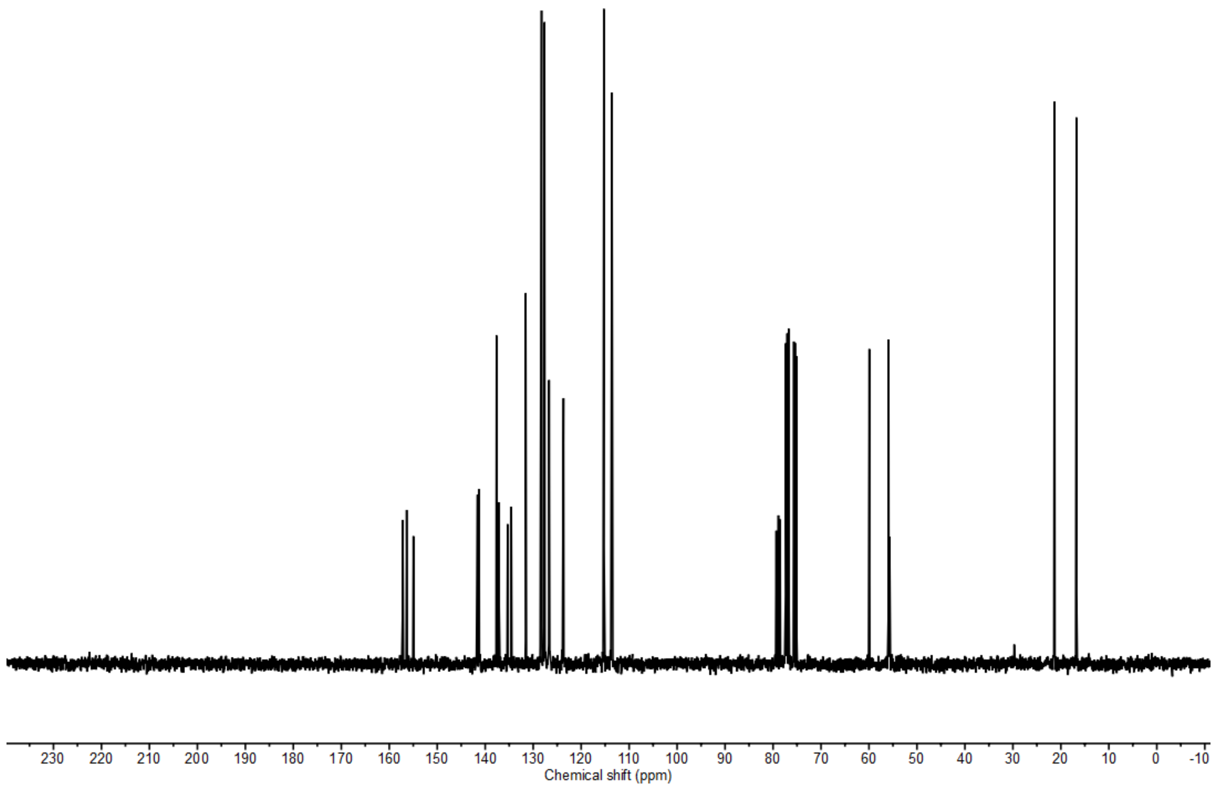

Figure S69 ^{13}C NMR of 2^{ABC} (101 MHz, $CDCl_3$)

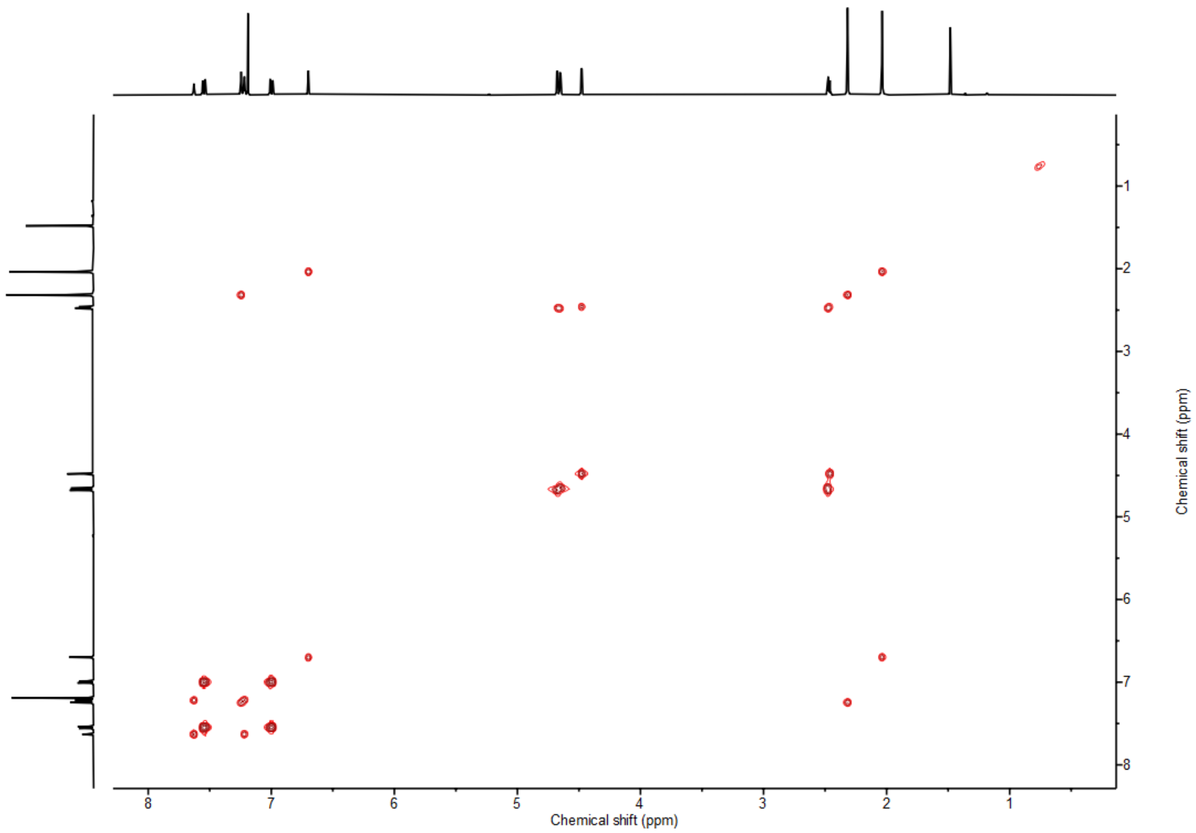

Figure S70 ^1H COSY NMR of 2^{ABC} (400 MHz, $CDCl_3$)

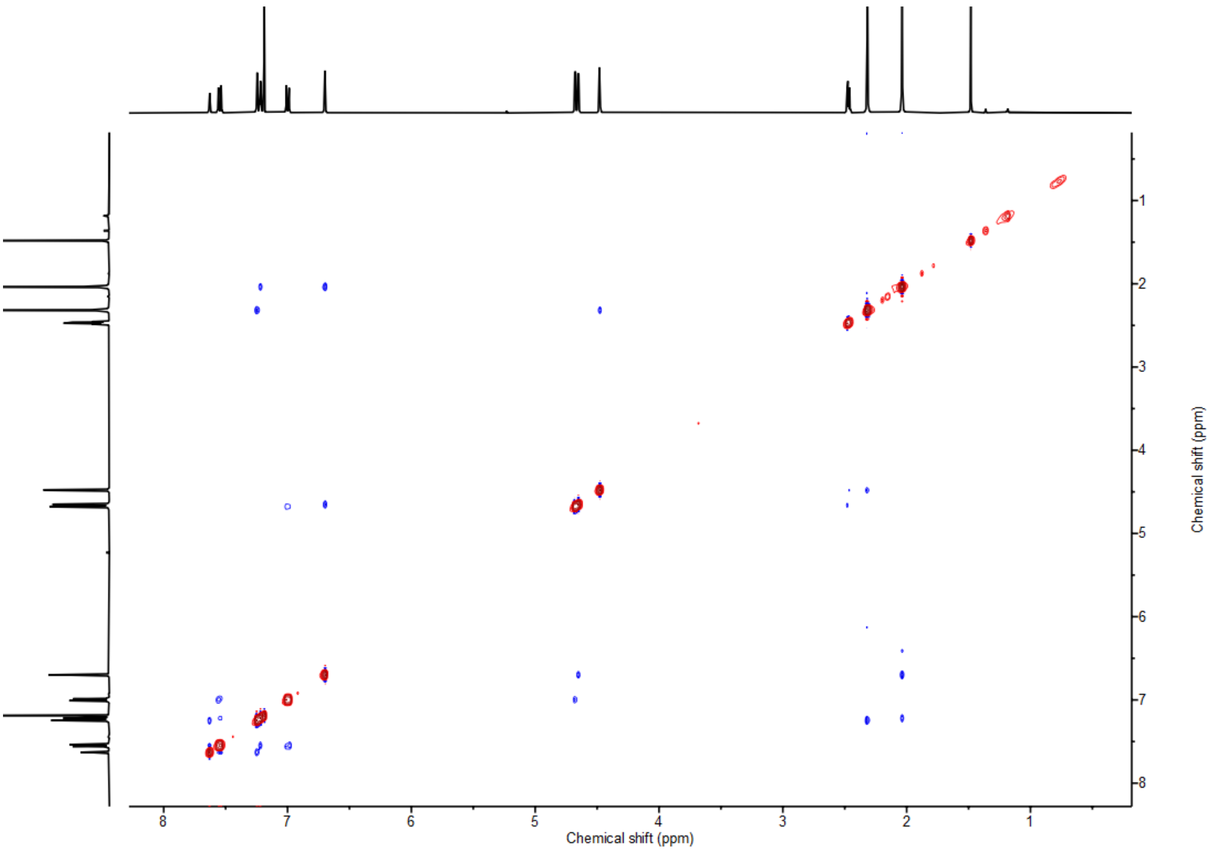

Figure S71 ^{1}H NOESY NMR of 2^{ABC} (400 MHz, $CDCl_3$)

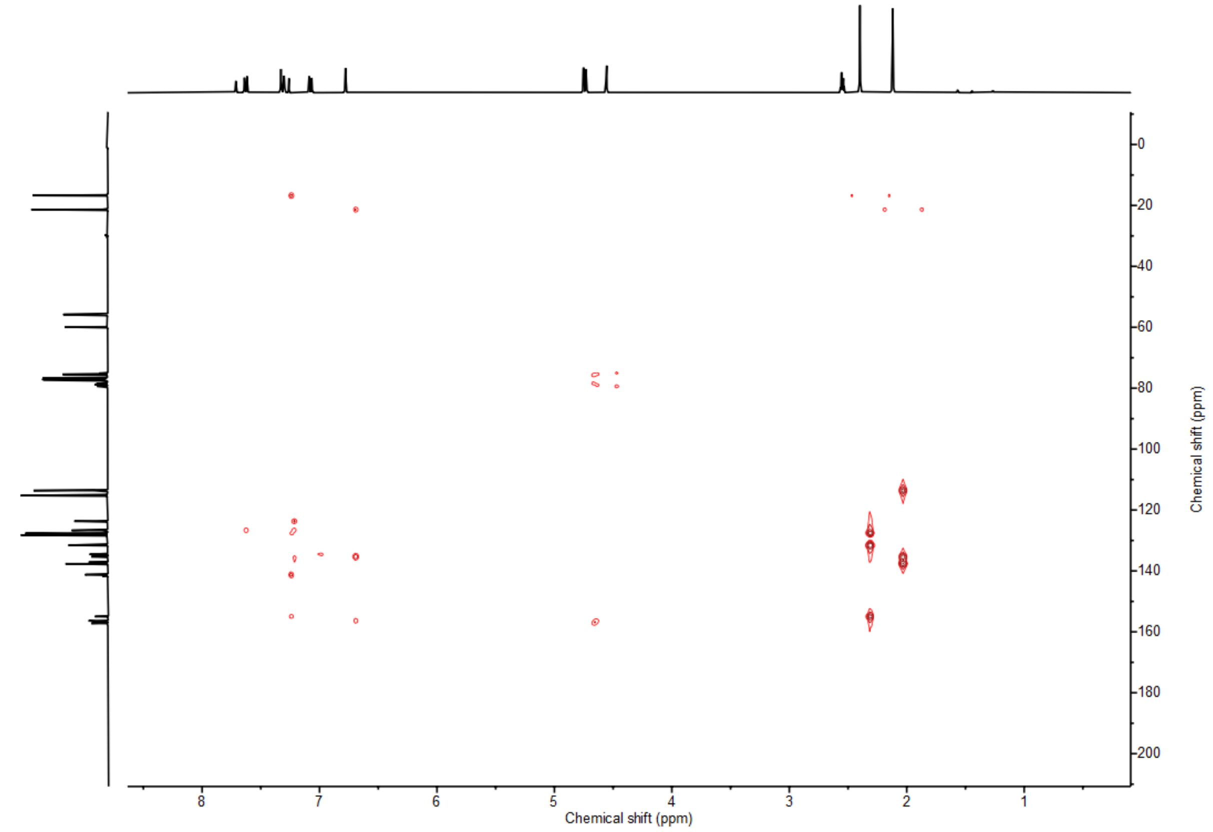

Figure S72 HMBC NMR of 2^{ABC} ($CDCl_3$)

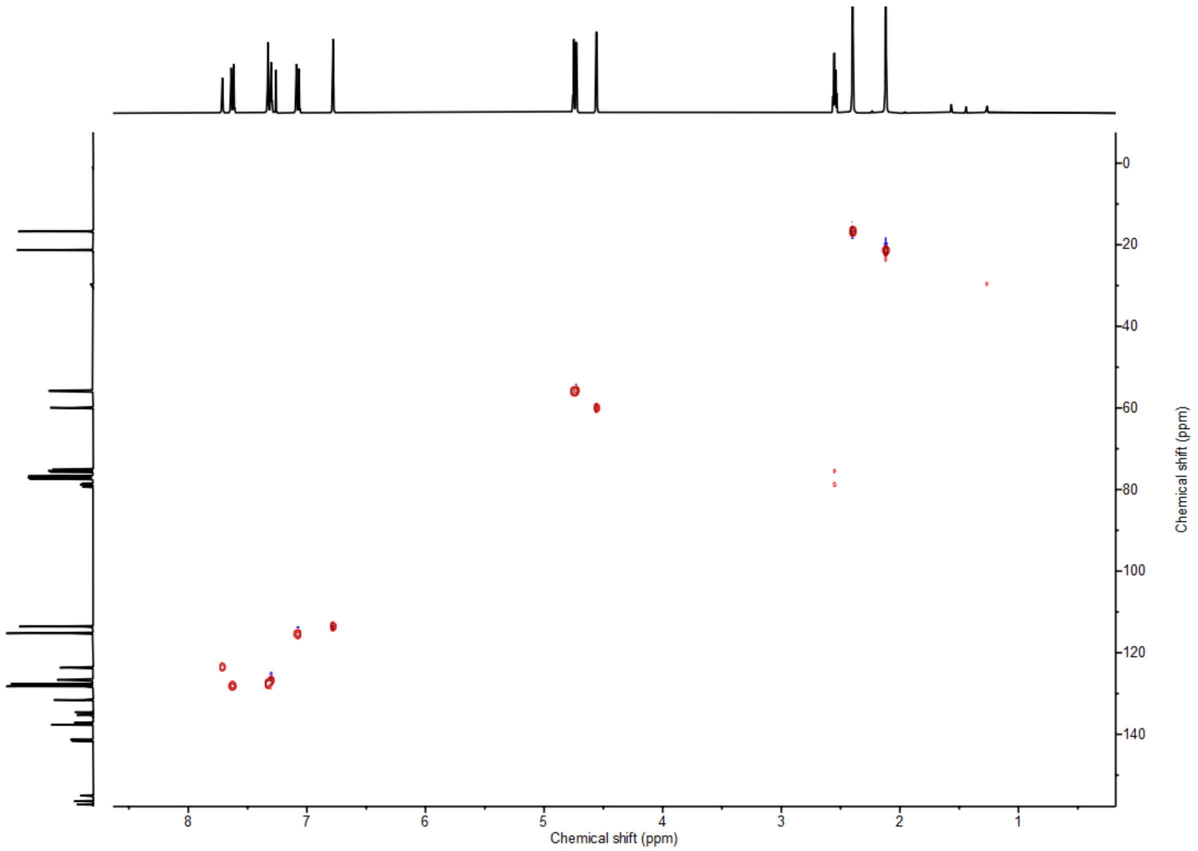

Figure S73 HSQC NMR of 2^{ABC} ($CDCl_3$)

Synthesis of 1

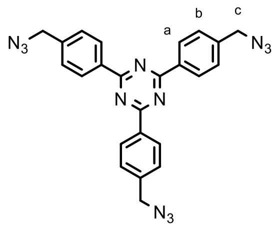

2,4,6-Tris(4-(bromomethyl)phenyl)-1,3,5-triazine (0.412 g, 0.7 mmol, 1 eq.) was dissolved in DMF (15 mL) and NaN_3 (0.1930 g, 2.8 mmol, 4.0 eq.) added portionwise. After stirring at rt for 14 h, water (30 mL) was added and the resultant white precipitate isolated by filtration, washed with water (3 × 20 mL) and dried under vacuum to give the product as a white solid (0.331 g, 99%).

^{1}H NMR (400 MHz, $CDCl_3$, 298 K) δ: 8.79 (d, *J* = 8.4 Hz, 6H, H_a), 7.54 (d, *J* = 8.4 Hz, 6H, H_b), 4.49 (s, 6H, H_c).

^{13}C NMR (101 MHz, $CDCl_3$, 298 K) δ: 171.31, 140.02, 136.08, 129.52, 128.37, 54.51.

HR-ESI-MS *m/z* = 475.1826 $[M+H]^+$ calc. 475.1856.

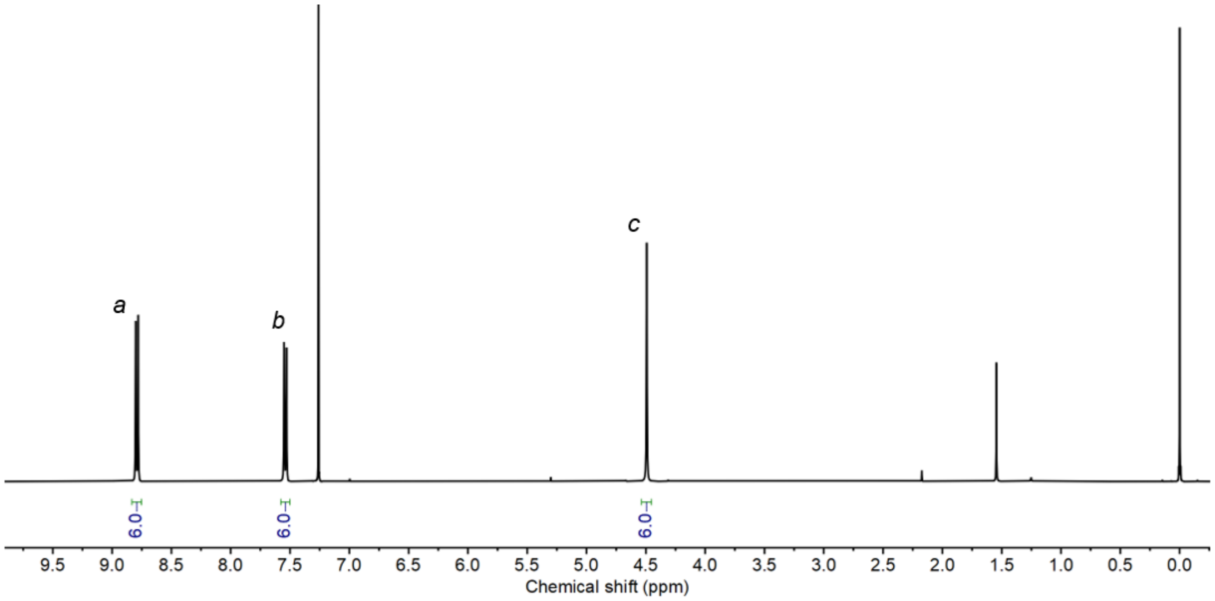

Figure S74 ^{1}H NMR of 1 (400 MHz, $CDCl_3$)

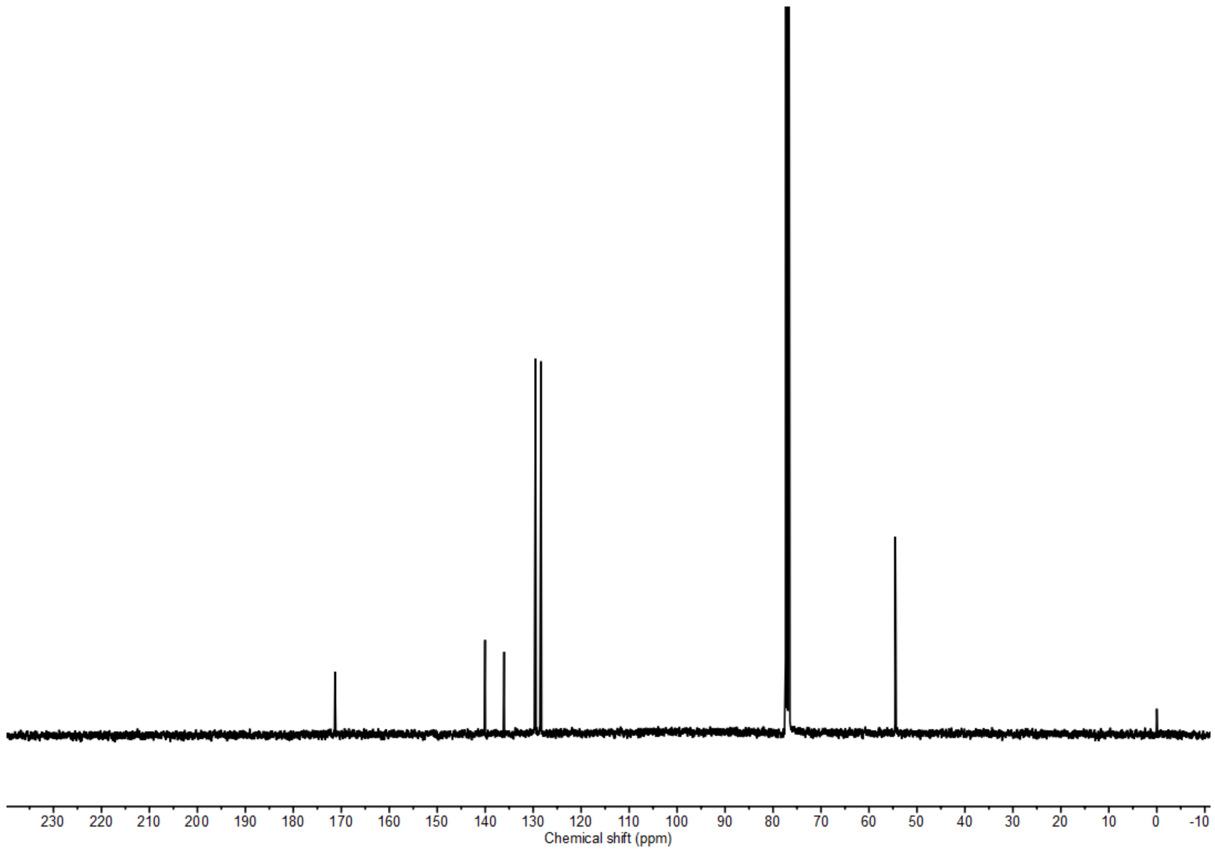

Figure S75 ^{13}C NMR of 1 (101 MHz, $CDCl_3$)

Synthesis of cage AAA

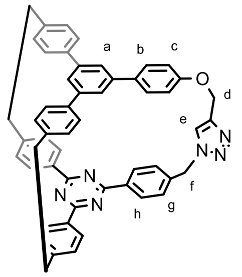

CuI (2.5 mg, 0.13 mmol, 0.5 eq.) was suspended in toluene (38 mL) and heated to 75 °C under N_2 atmosphere. DBU (0.125 mL, 0.43 mmol, 34 eq.) was added to this and stirred for 15 minutes. A solution of **2^{AAA}** (11.9 mg, 0.025 mmol, 1.0 eq.) and **1** (11.8 g, 0.025 mmol, 1.0 eq.) in 1:2 THF/toluene (2.5 mL) was then added to the catalytic solution via syringe pump over 18 h. Once addition was complete, the solution was stirred for an additional 48 h at 110 °C. After the solvent was removed *in vacuo*, the residue was dissolved in CH_2Cl_2 (20 mL). The organic phase was washed with brine (3 × 20 mL), dried ($MgSO_4$) and the solvent removed *in vacuo*. After purification by column chromatography on silica gel (0-3% gradient MeOH in CH_2Cl_2) the product was obtained as an off-white solid (13.8 mg, 58%).

^{1}H NMR (400 MHz, $CDCl_3$, 298 K) δ: 8.47 (d, *J* = 8.2 Hz, 6H, H$_h$), 7.26 – 7.18 (m, 12H, H$_c$, H$_g$), 7.15 (s, 3H, H$_a$), 7.03 (s, 3H, H$_e$), 6.73 (d, *J* = 8.6 Hz, 6H, H$_b$), 5.55 (s, 6H, H$_f$), 5.40 (s, 6H, H$_d$).

^{13}C NMR (101 MHz, 4:1 $CDCl_3$/CD_3OD, 298 K) δ: 170.7, 156.1, 141.0, 139.2, 136.2, 134.3, 129.4, 128.1, 127.8, 123.2, 116.9, 62.4, 54.0, 29.6.

HR-ESI-MS *m/z* = 943.3582 [M+H]$^+$ calc. 943.3581.

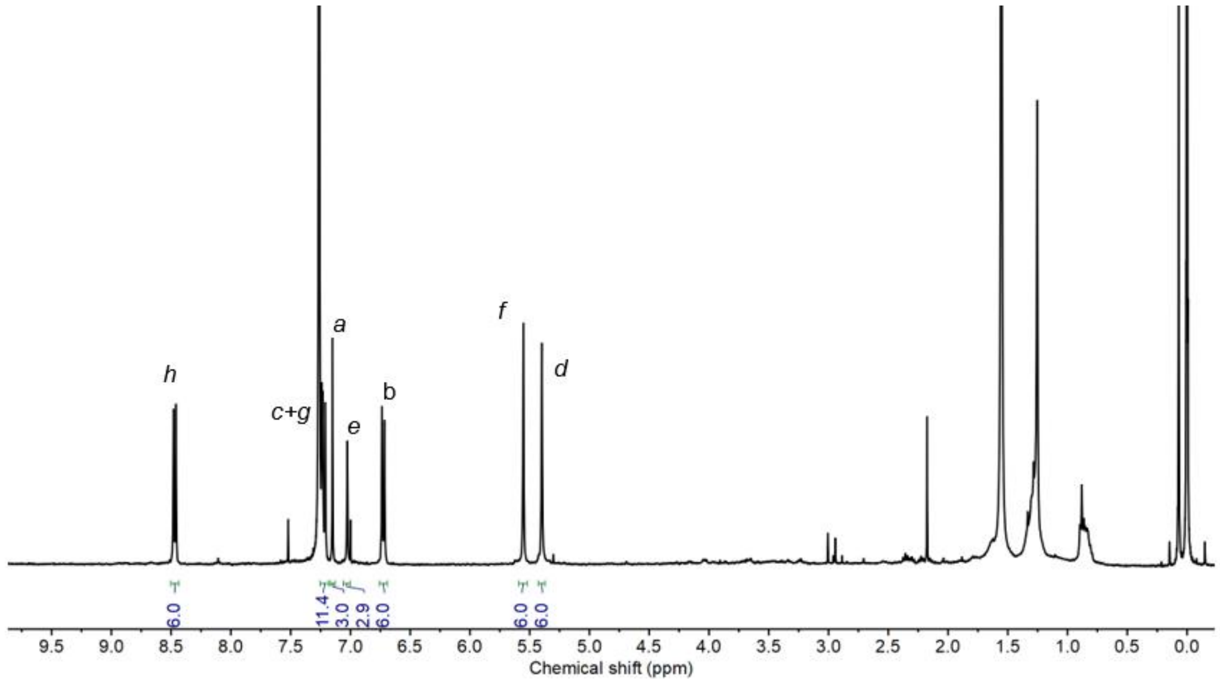

Figure S76 ^{1}H NMR of AAA (400 MHz, $CDCl_3$)

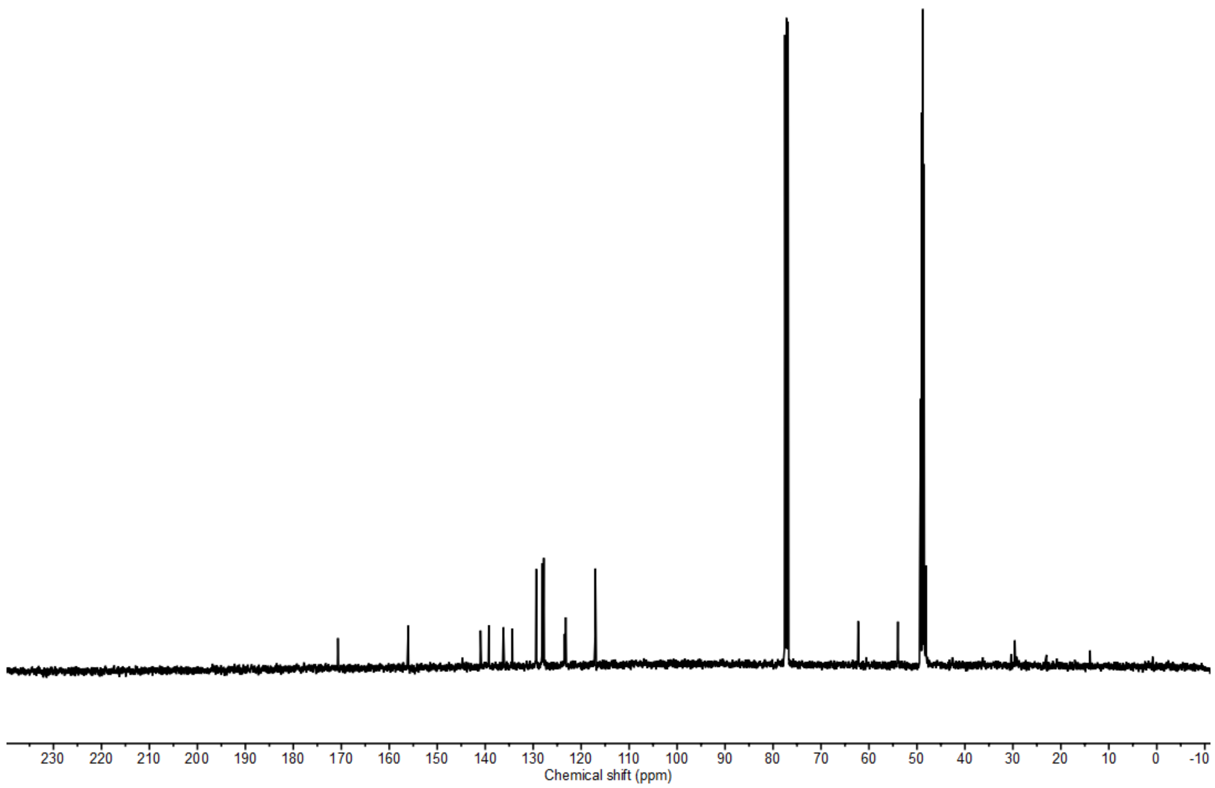

Figure S77 ^{13}C NMR of AAA (101 MHz, 4:1 $CDCl_3/CD_3OD$)

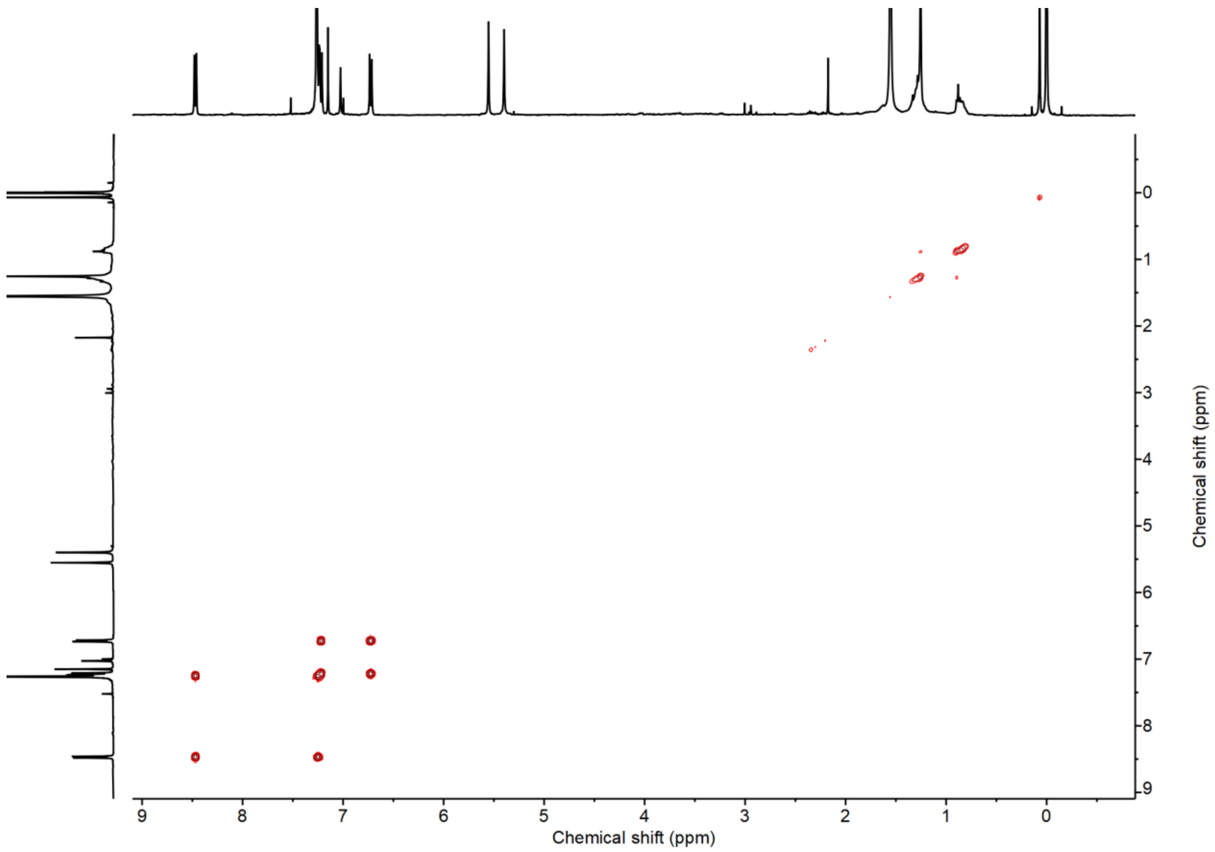

Figure S78 COSY NMR of AAA (400 MHz, $CDCl_3$)

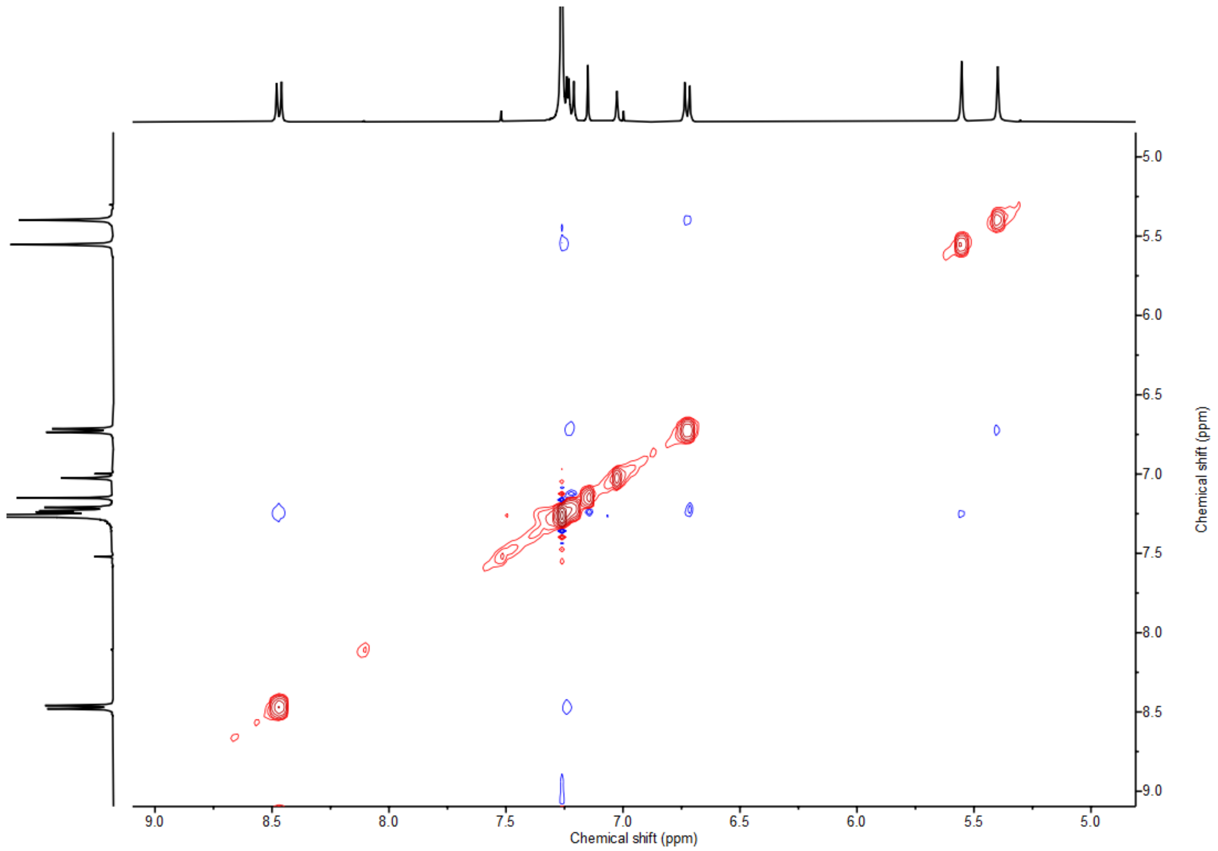

Figure S79 Partial NOESY NMR of AAA (400 MHz, $CDCl_3$)

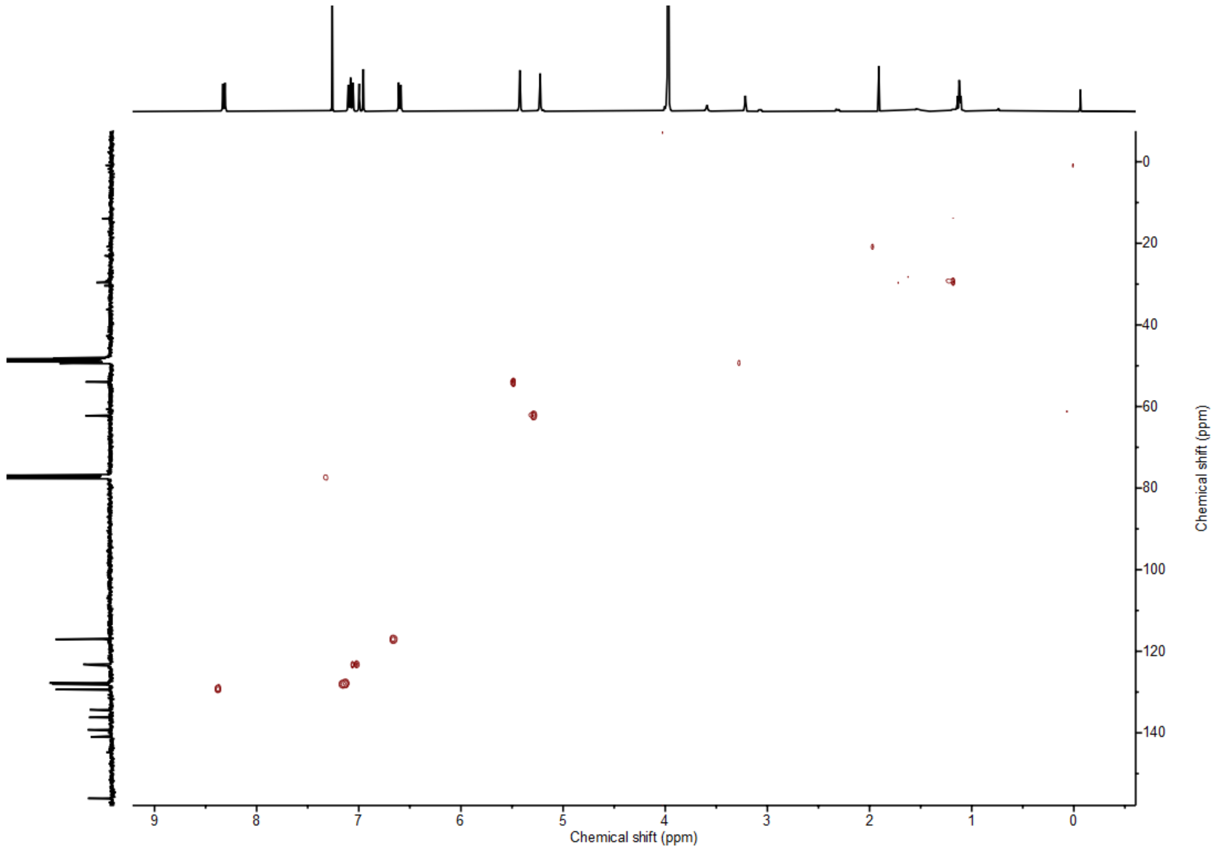

Figure S80 HSQC NMR of AAA (4:1 $CDCl_3$/CD_3OD)

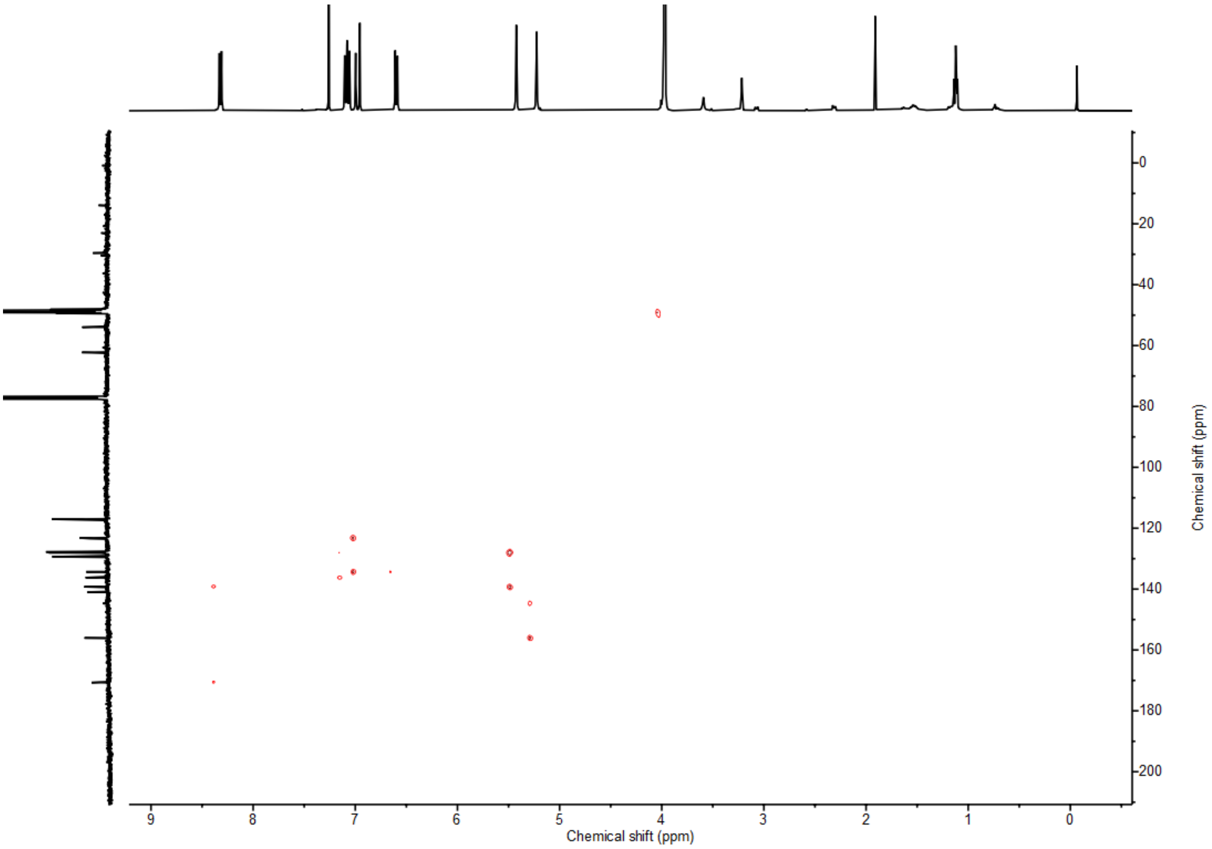

Figure S81 HMBC NMR of AAA (4:1 $CDCl_3/CD_3OD$)

Synthesis of cage BBB

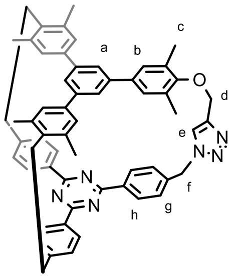

CuI (4.9 mg, 0.25 mmol, 0.5 eq.) was suspended in toluene (75 mL) and heated to 75 °C under N_2 atmosphere. DBU (0.25 mL, 0.85 mmol, 34 eq.) was added to this and stirred for 15 minutes. A solution of **2^BBB^** (27.6 mg, 0.05 mmol, 1.0 eq.) and **1** (23.6 g, 0.05 mmol, 1.0 eq.) in 1:2 THF/toluene (6 mL) was then added to the catalytic solution over 18 h via syringe pump. Once addition was complete, the solution was stirred at 110 °C for an additional 48 h. After the solvent was removed *in vacuo*, the residue was dissolved in CH_2Cl_2 (20 mL). The organic phase was washed with brine (3 × 20 mL), dried ($MgSO_4$) and the solvent removed *in vacuo*. After purification by column chromatography on silica gel (0-3% gradient MeOH in CH_2Cl_2) the product was obtained as a white solid (34.6 mg, 67%).

^{1}H NMR (400 MHz, $CDCl_3$, 298 K) δ: 8.62 (d, *J* = 8.4 Hz, 6H, H_h), 7.32 (d, *J* = 8.2 Hz, 6H, H_e), 6.83 (s, 3H, H_a), 6.71 (s, 6H, H_b), 6.52 (s, 3H, H_e), 5.54 (s, 6H, H_f), 5.22 (s, 6H, H_d), 1.96 (s, 18H, H_c).

^{13}C NMR (101 MHz, $CDCl_3$, 298 K) δ: 157.0, 142.1, 137.4, 131.3, 129.6, 128.5, 128.2, 125.9, 77.2, 54.0, 17.1.

HR-ESI-MS *m/z* = 1027.4486 $[M+H]^+$ calc. 1027.4520. *m/z* = 514.2265 $[M+2H]^{2+}$ calc. 514.2299.

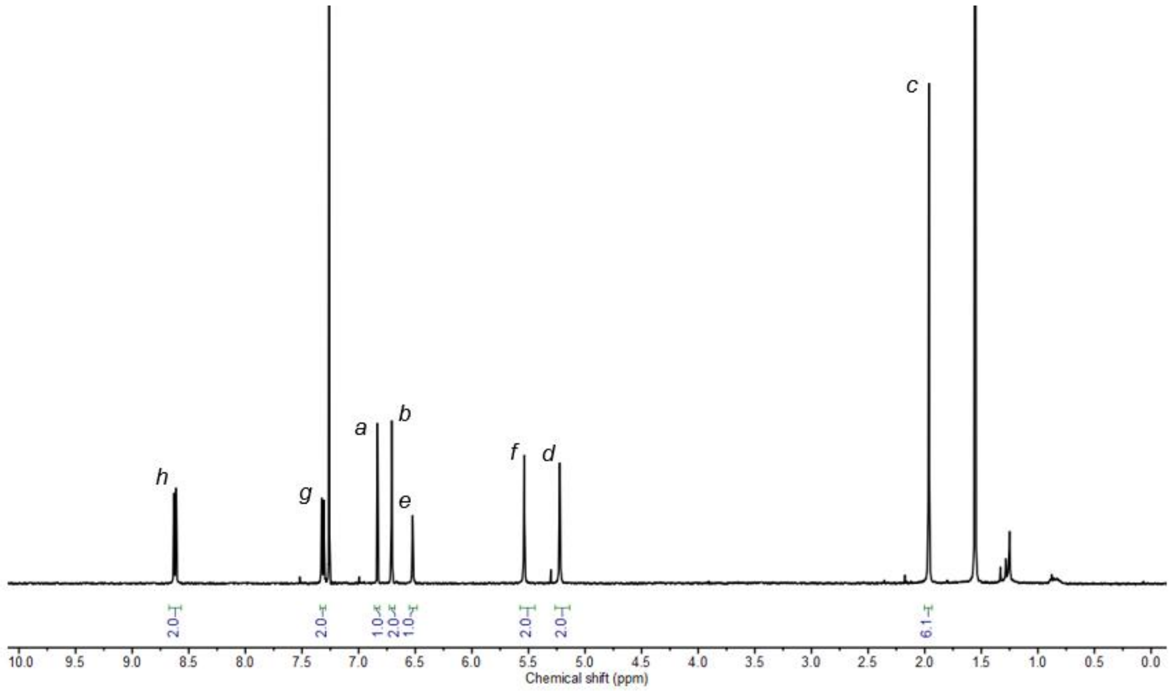

Figure S82 ^{1}H NMR of BBB (400 MHz, $CDCl_3$)

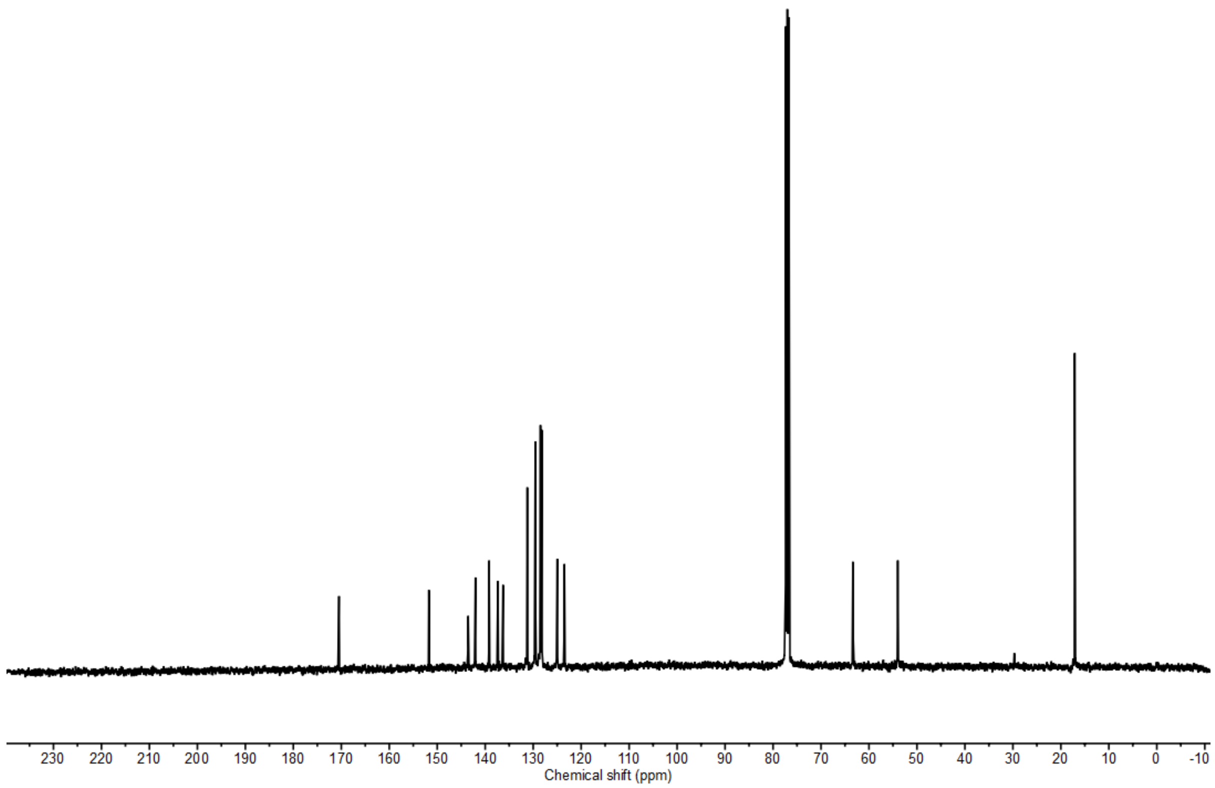

Figure S83 ^{13}C NMR of BBB (101 MHz, 4CDCl$_3$)

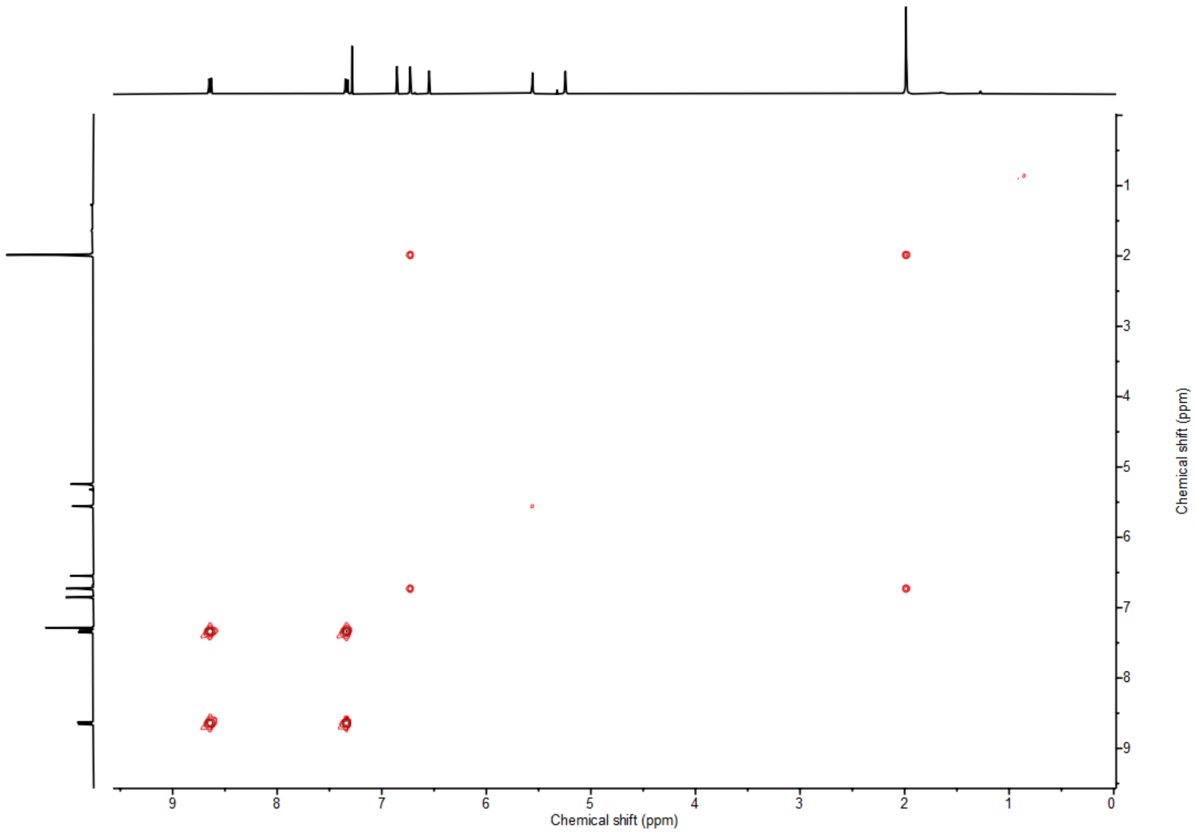

Figure S84 COSY NMR of BBB (400 MHz, CDCl$_3$)

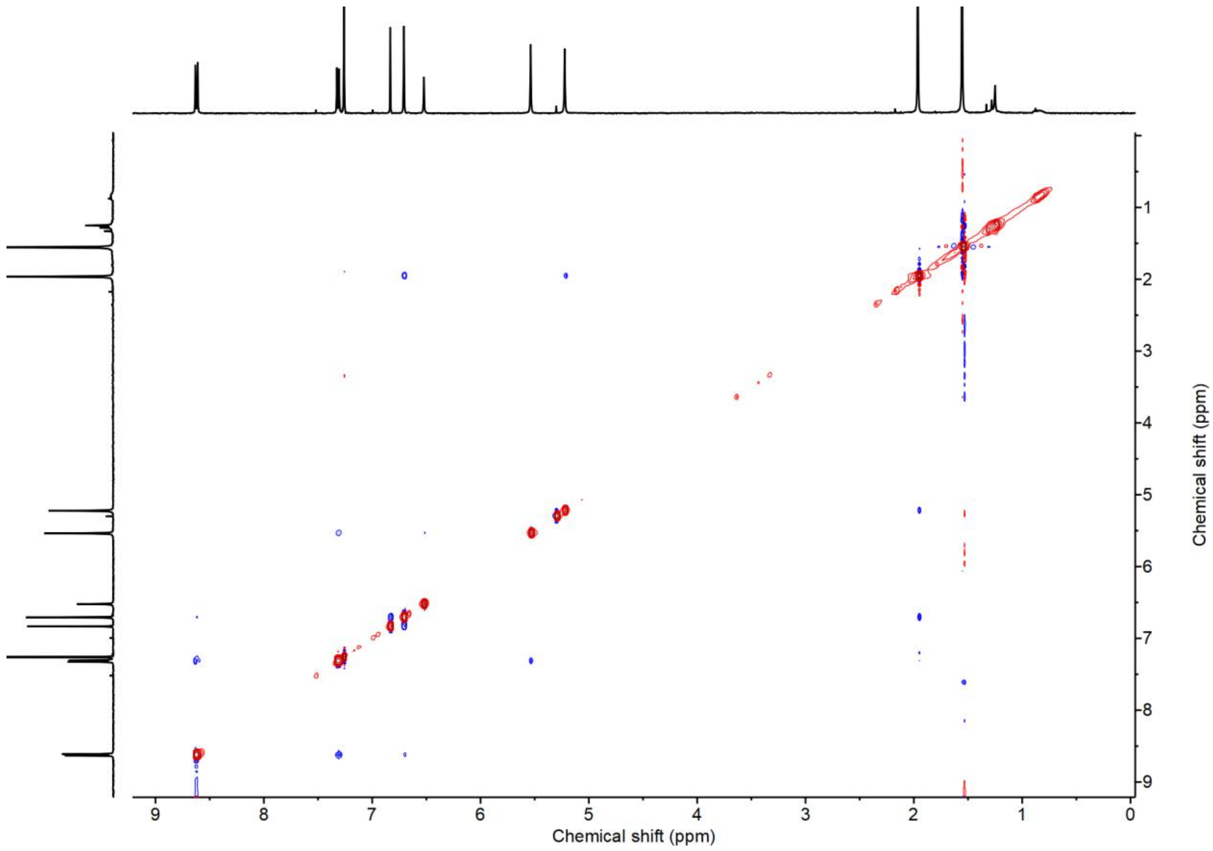

Figure S85 NOESY NMR of BBB (400 MHz, $CDCl_3$)

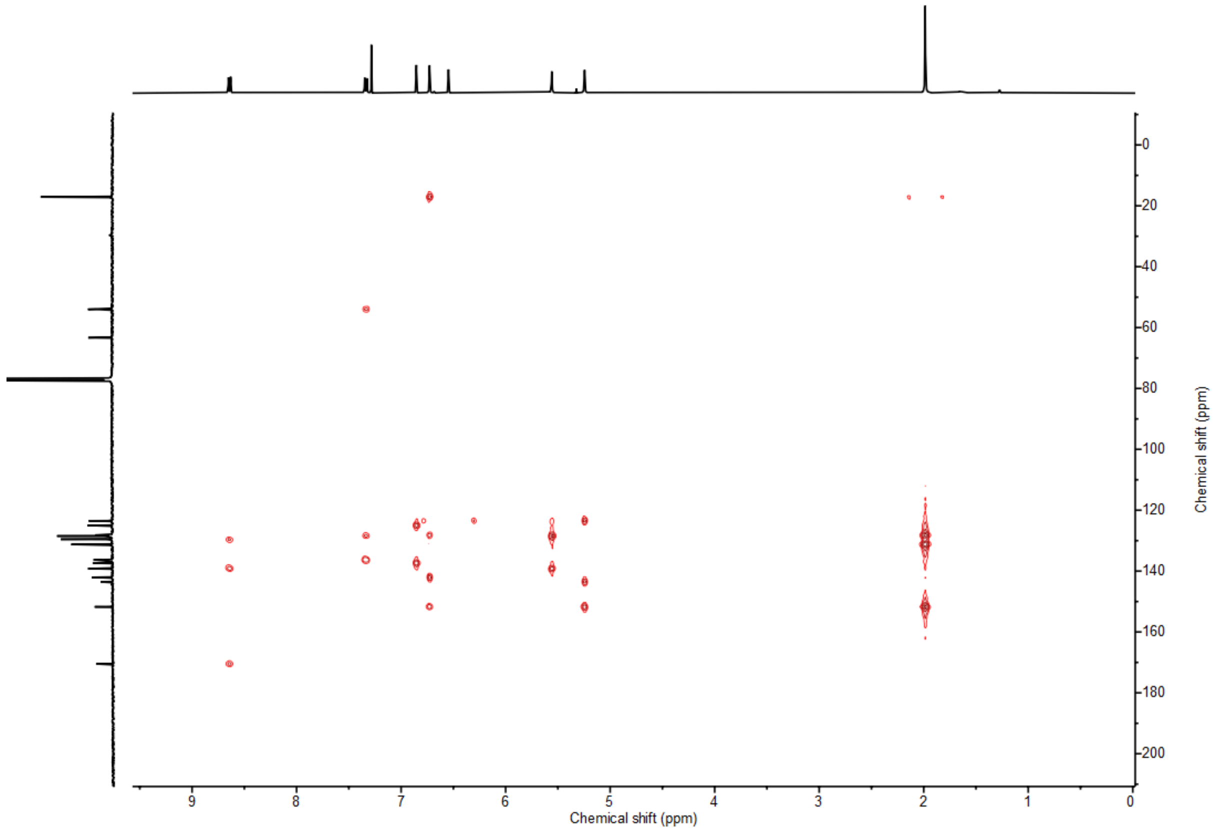

Figure S86 HMBC NMR of BBB ($CDCl_3$)

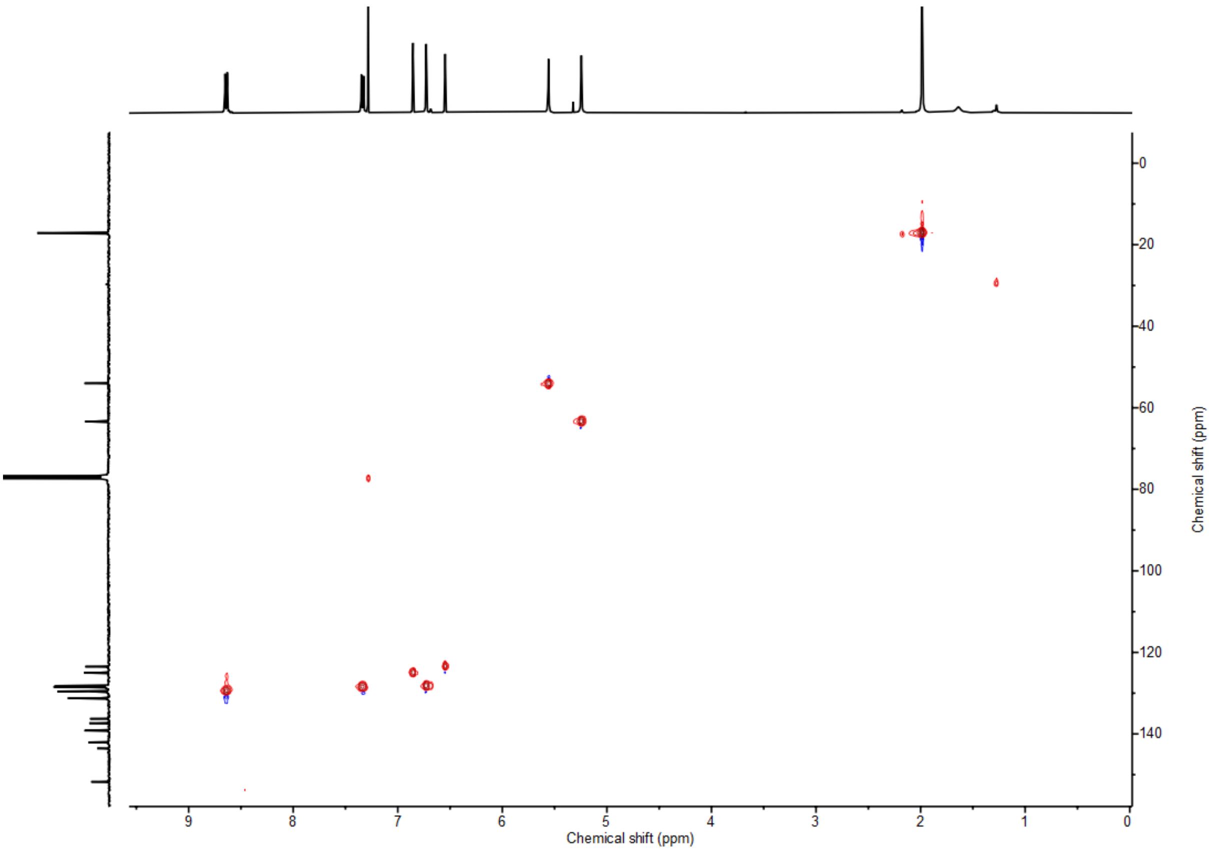

Figure S87 HSQC NMR of BBB ($CDCl_3$)

Synthesis of cage CCC

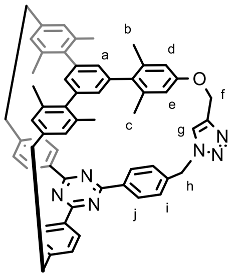

CuI (4.9 mg, 0.25 mmol, 0.5 eq.) was suspended in toluene (75 mL) and heated to 75 °C under N_2 atmosphere. DBU (0.25 mL 0.85 mmol, 34 eq.) was added to this and stirred for 15 minutes. A solution of **2**$^{\mathbf{CCC}}$ (27.6 mg, 0.05 mmol, 1.0 eq.) and **1** (23.6 g, 0.05 mmol, 1.0 eq.) in 2:3 THF/toluene (5 mL) were then added to the catalytic solution over 18 h via syringe pump. Once addition was complete, the solution was stirred at 110 °C for 48 h After the solvent was removed *in vacuo*, the residue was dissolved in CH_2Cl_2 (20 mL). The organic phase was washed with brine (3 × 20 mL), dried ($MgSO_4$) and the solvent removed *in vacuo*. After purification by column chromatography on silica gel (0-3% gradient MeOH in CH_2Cl_2) the product was obtained as an off-white solid (37.7 mg, 74%).

^{1}H NMR (400 MHz, $CDCl_3$, 298 K) δ: 8.61 (d, *J* = 8.3 Hz, 6H, H_j), 7.51 (d, *J* = 8.4 Hz, 6H, H_i), 7.27 (s, 3H, H_a), 6.69 (d, *J* = 2.6 Hz, 3H, H_d), 6.50 (s, 3H, H_g), 5.78 (d, *J* = 2.6 Hz, 3H, H_e), 5.63 (s, 6H, H_h), 5.23 (s, 6H, H_f), 1.98 (s, 18H, H_b), 1.03 (s, 18H, H_c).

^{13}C NMR (101 MHz, $CDCl_3$, 298 K) δ: 171.0, 156.5, 146.0, 140.1, 139.6, 137.1, 136.5, 136.5, 135.3, 129.7, 129.1, 128.8, 122.8, 118.0, 115.6, 65.0, 54.2, 29.7, 20.7, 20.6.

HR-ESI-MS *m/z* = 1027.4565 $[M+H]^+$ calc. 1027.4520.

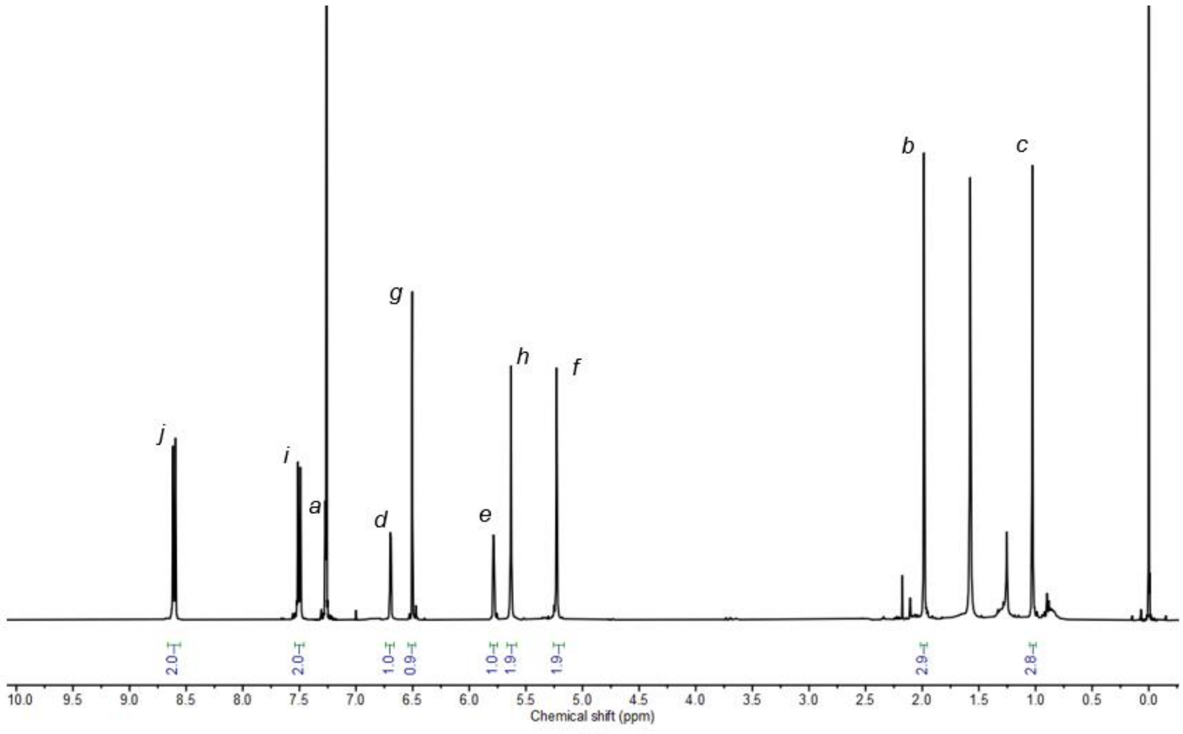

Figure S88 ^{1}H NMR of CCC (400 MHz, $CDCl_3$)

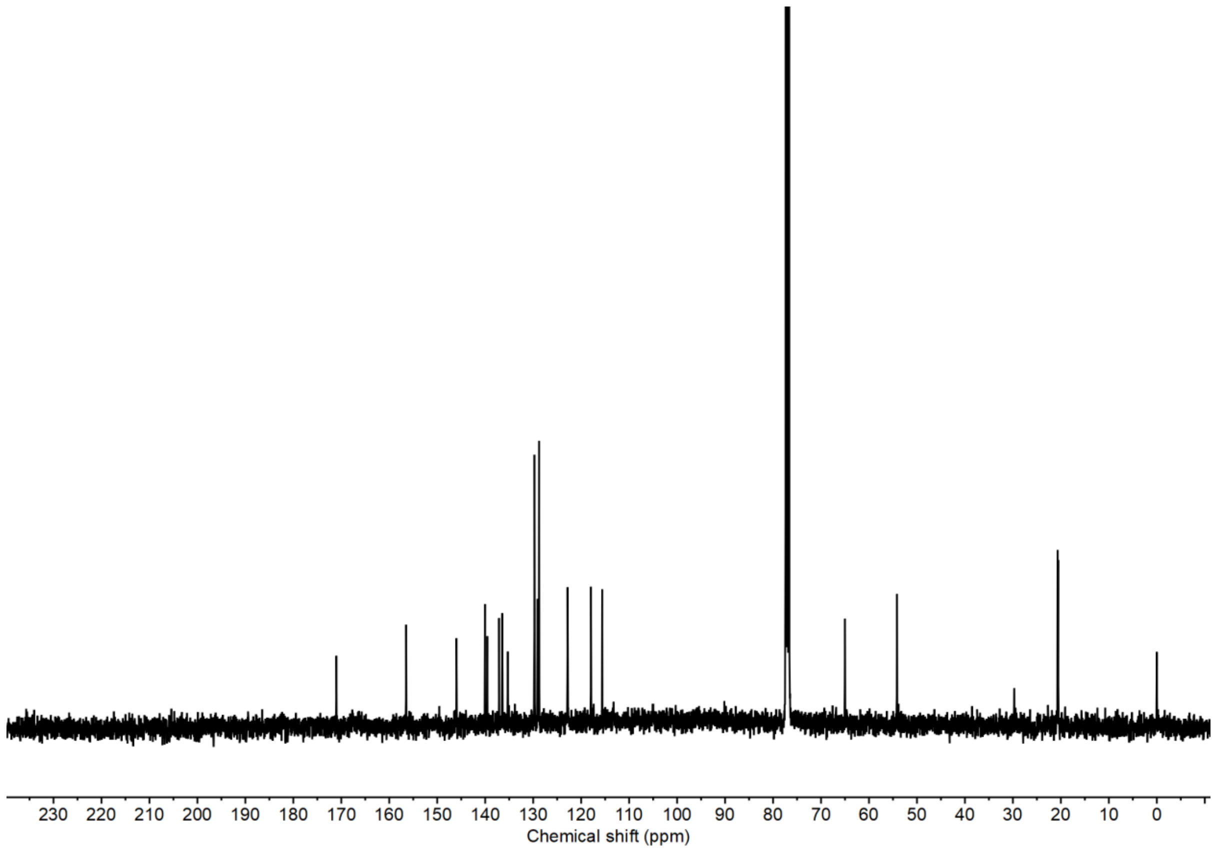

Figure S89 ^{13}C NMR of CCC (101 MHz, $CDCl_3$)

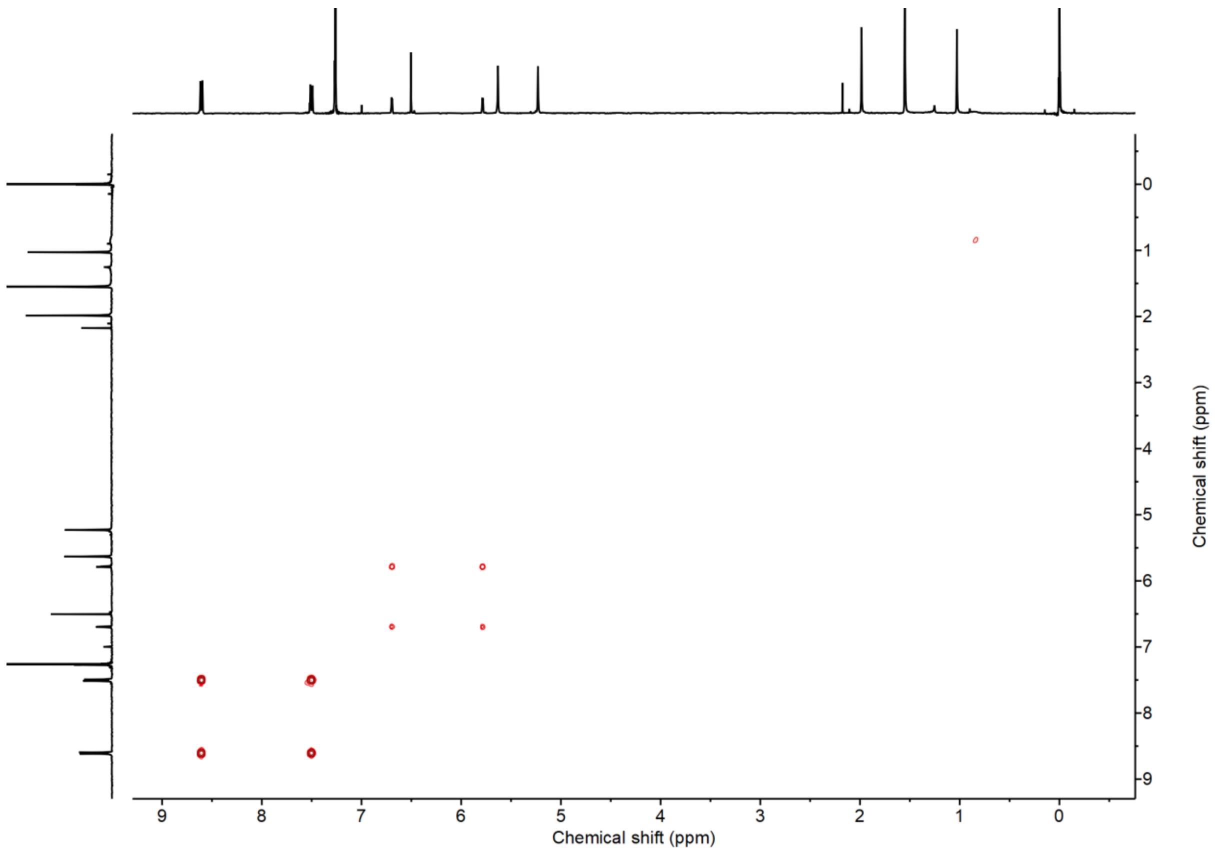

Figure S90 COSY NMR of CCC (400 MHz, $CDCl_3$)

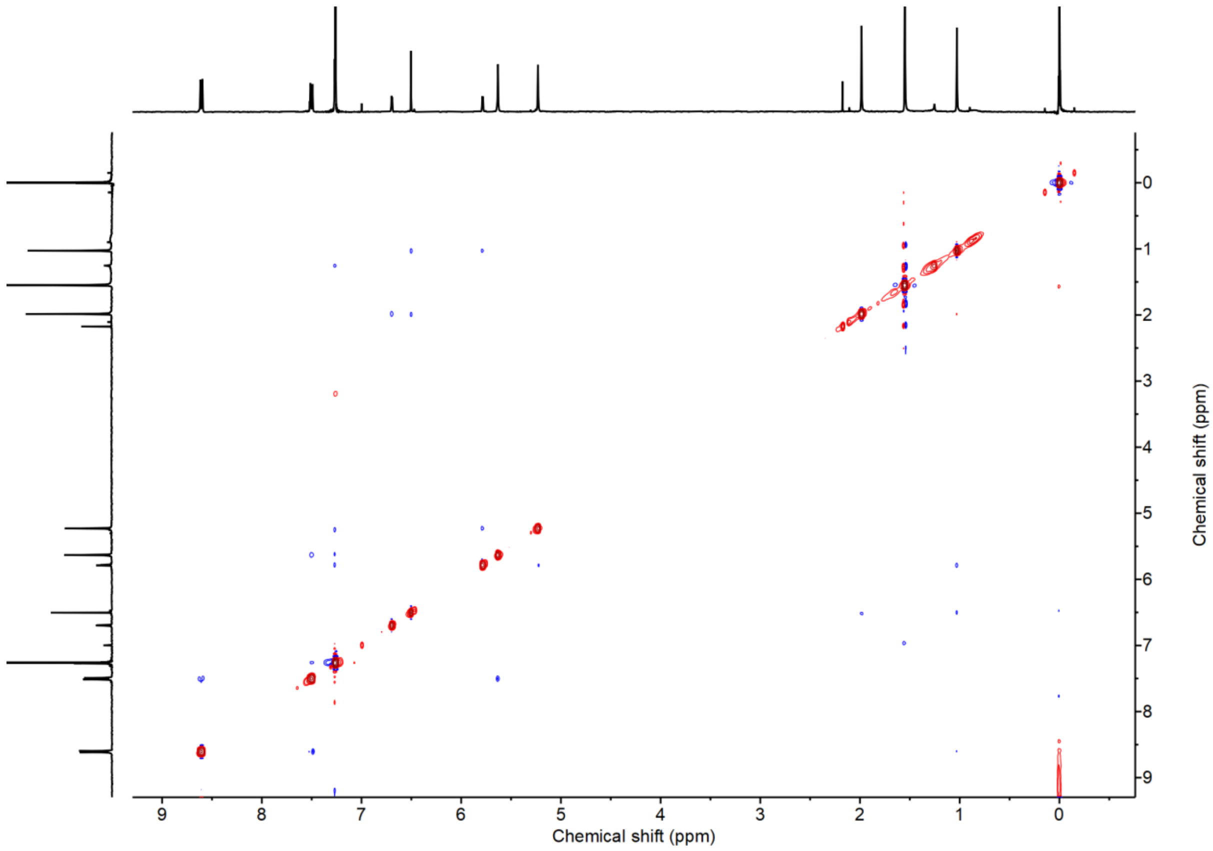

Figure S91 NOESY NMR of CCC (400 MHz, $CDCl_3$)

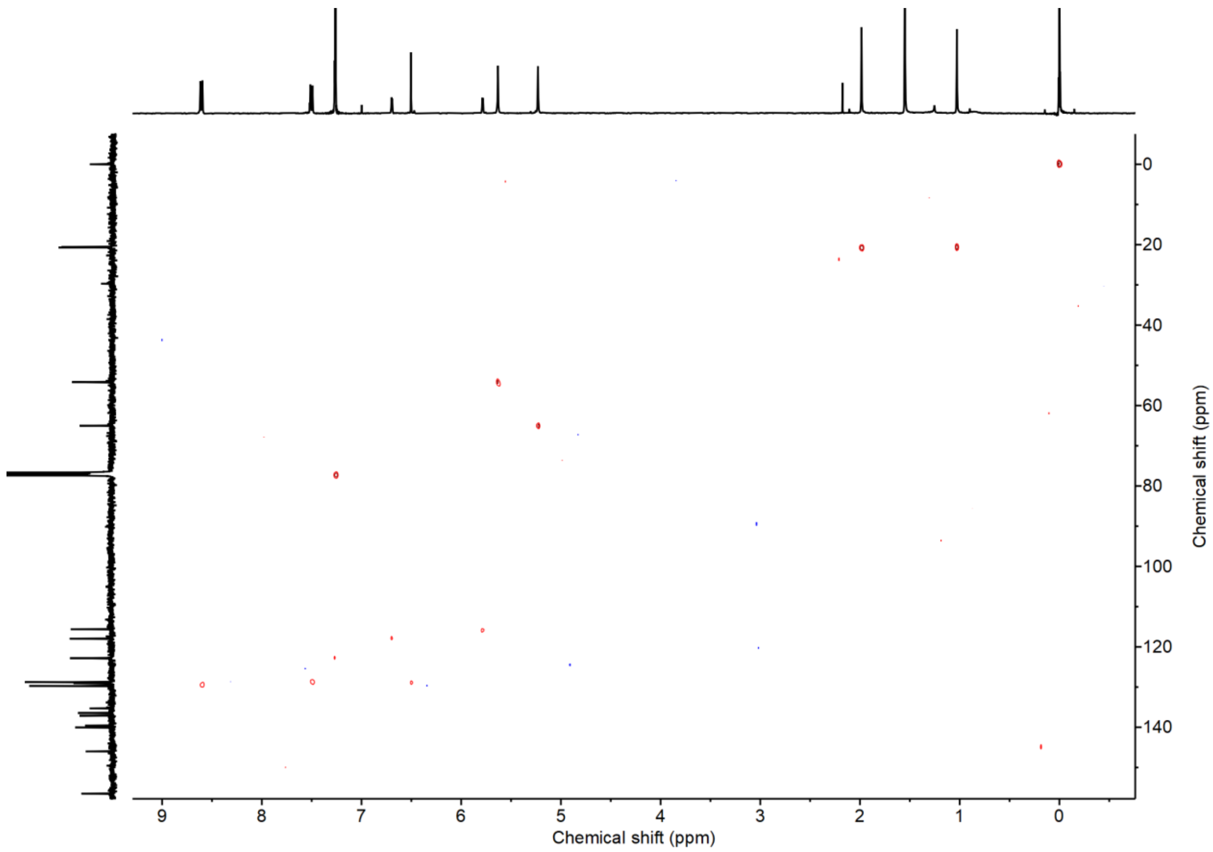

Figure S92 HSQC NMR of CCC ($CDCl_3$)

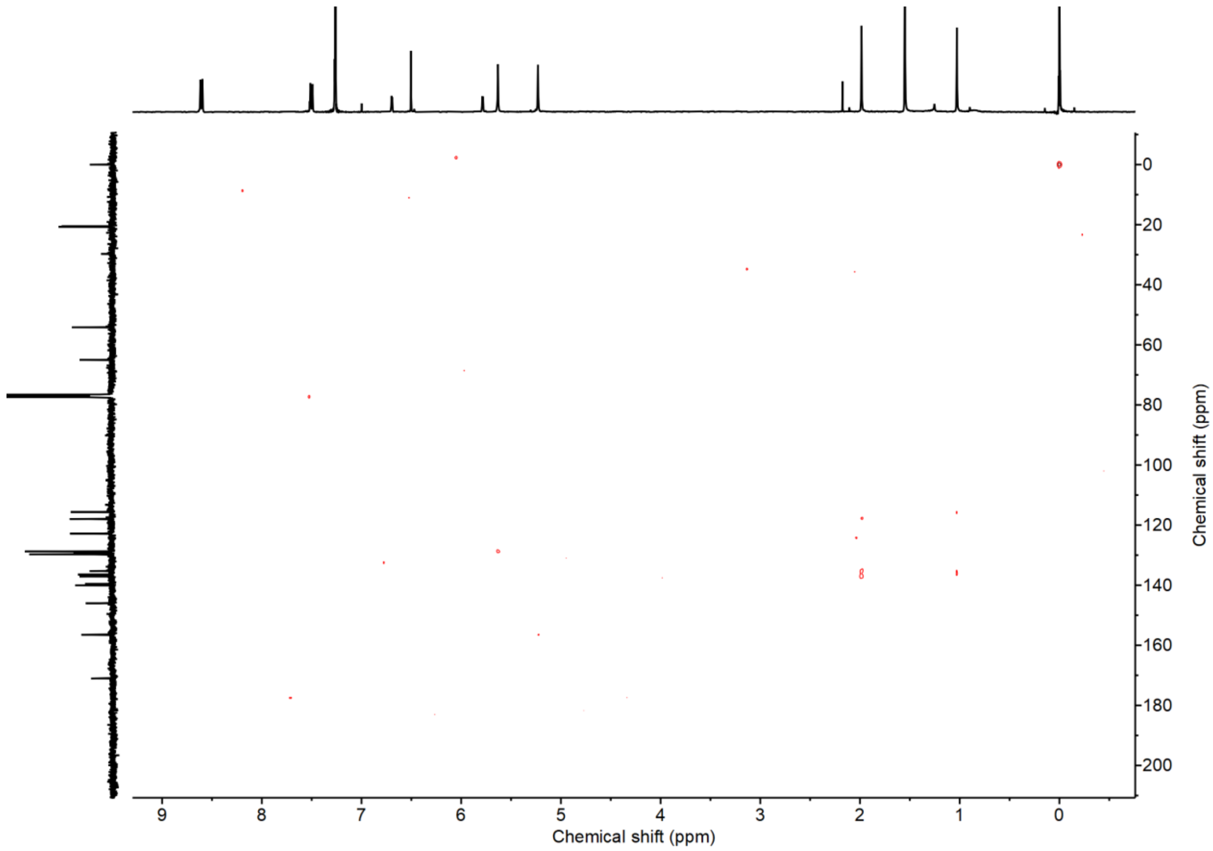

Figure S93 HMBC NMR of CCC ($CDCl_3$)

Synthesis of cage ABB

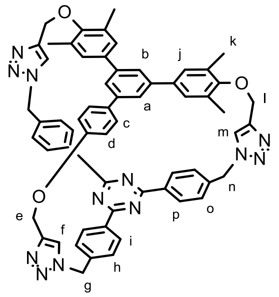

CuI (2.5 mg, 0.13 mmol, 0.5 eq.) was suspended in toluene (38 mL) and heated to 75 °C under N_2 atmosphere. DBU (0.13 mL, 0.43 mmol, 34 eq.) was added to this and stirred for 15 minutes. A solution of **2**$^{\mathbf{ABB}}$ (13.8 mg, 0.025 mmol, 1.0 eq.) and **1** (11.8 g, 0.025 mmol, 1.0 eq.) in 1:2 degassed THF/toluene (3 mL) were then added to the catalytic solution over 18 h via syringe pump. Once addition was complete, the solution was stirred at 110 °C for 48 h. After the solvent was removed *in vacuo*, the residue was dissolved in CH_2Cl_2 (20 mL). The organic phase was washed with brine (3 × 20 mL), dried ($MgSO_4$) and the solvent removed *in vacuo*. After purification by column chromatography on silica gel (0-3% gradient MeOH in CH_2Cl_2) the product was obtained as an off-white solid (14.2 mg, 55%).

^{1}H NMR (400 MHz, $CDCl_3$, 298 K) δ: 8.58 (d, *J* = 8.2 Hz, 4H, H_p), 8.44 (d, *J* = 8.3 Hz, 2H, H_i), 7.29 (d, *J* = 8.2 Hz, 4H, H_o), 7.21 – 7.06 (m, 4H, H_h, H_c), 6.98 – 6.91 (m, 2H, H_f, H_b), 6.87 (d, *J* = 1.6 Hz, 2H, H_a), 6.73 (s, 4H, H_j), 6.68 (d, *J* = 8.7 Hz, 2H, H_d), 6.54 (s, 2H, H_m), 5.56 – 5.51 (m, 6H, H_g, H_n), 5.37 (s, 2H, H_e), 5.24 (s, 4H, H_l), 1.99 (s, 12H, H_k).

^{13}C NMR (101 MHz, $CDCl_3$, 298 K) δ: 170.7, 170.4, 155.8, 151.8, 144.9, 143.5, 142.2, 140.5, 139.7, 139.1, 137.3, 136.3, 136.2, 134.4, 131.3, 131.3, 129.5, 129.3, 128.4, 128.0, 127.8, 124.8, 124.0, 123.6, 117.0, 63.4, 62.2, 54.1, 53.9, 17.1.

HR-ESI-MS *m/z* = 999.4198 $[M+H]^+$ calc. 999.4207.

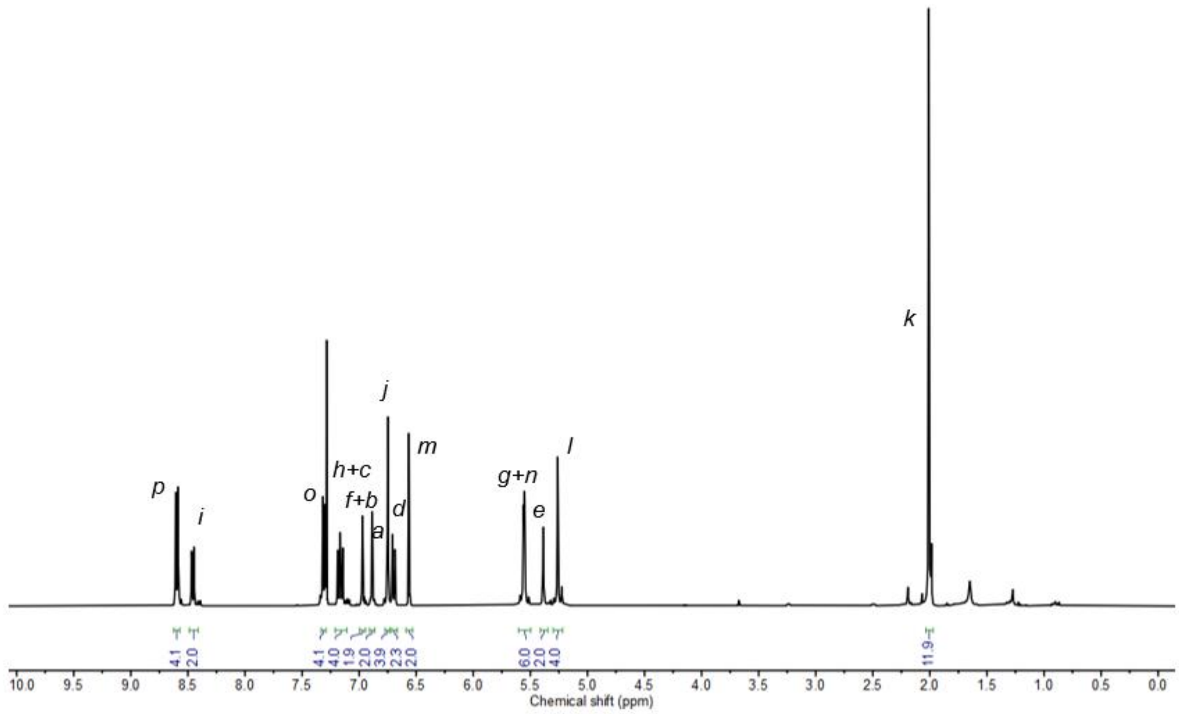

Figure S94 ^{1}H NMR of ABB (400MHz, $CDCl_3$)

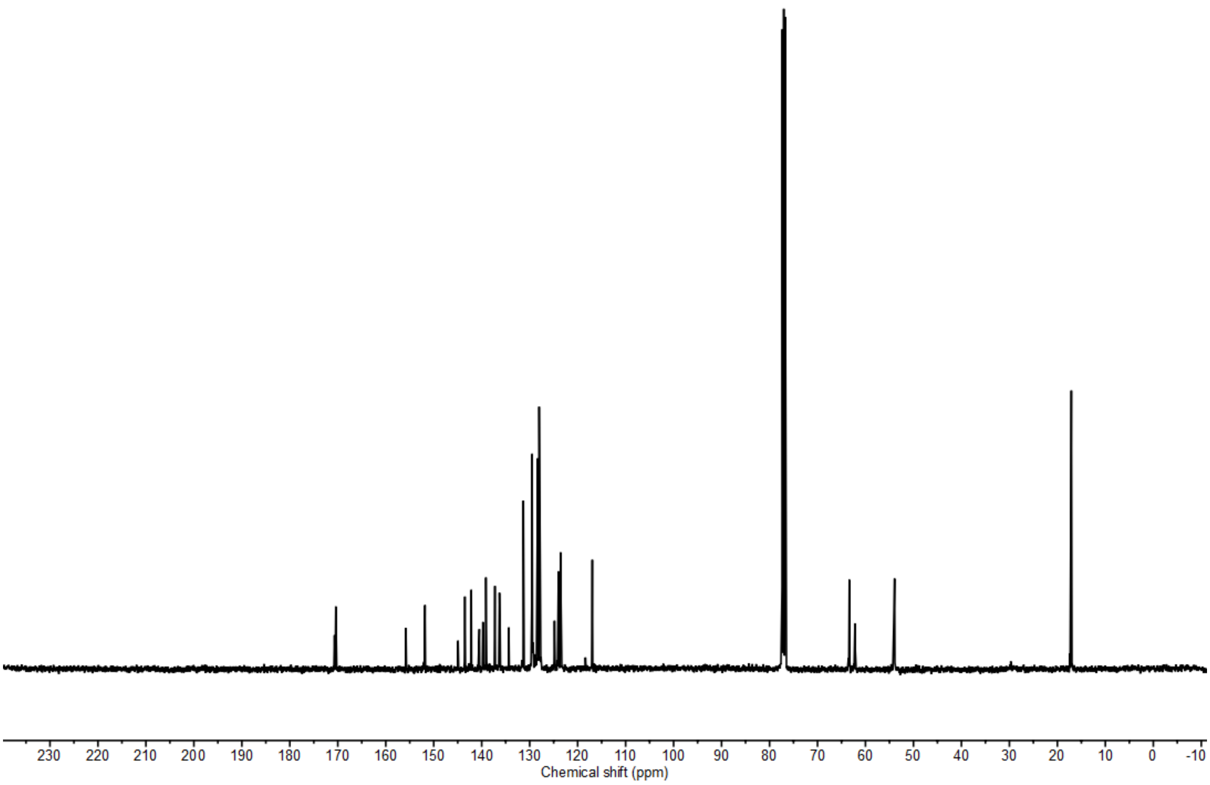

Figure S95 ^{13}C NMR of ABB (101 MHz, $CDCl_3$)

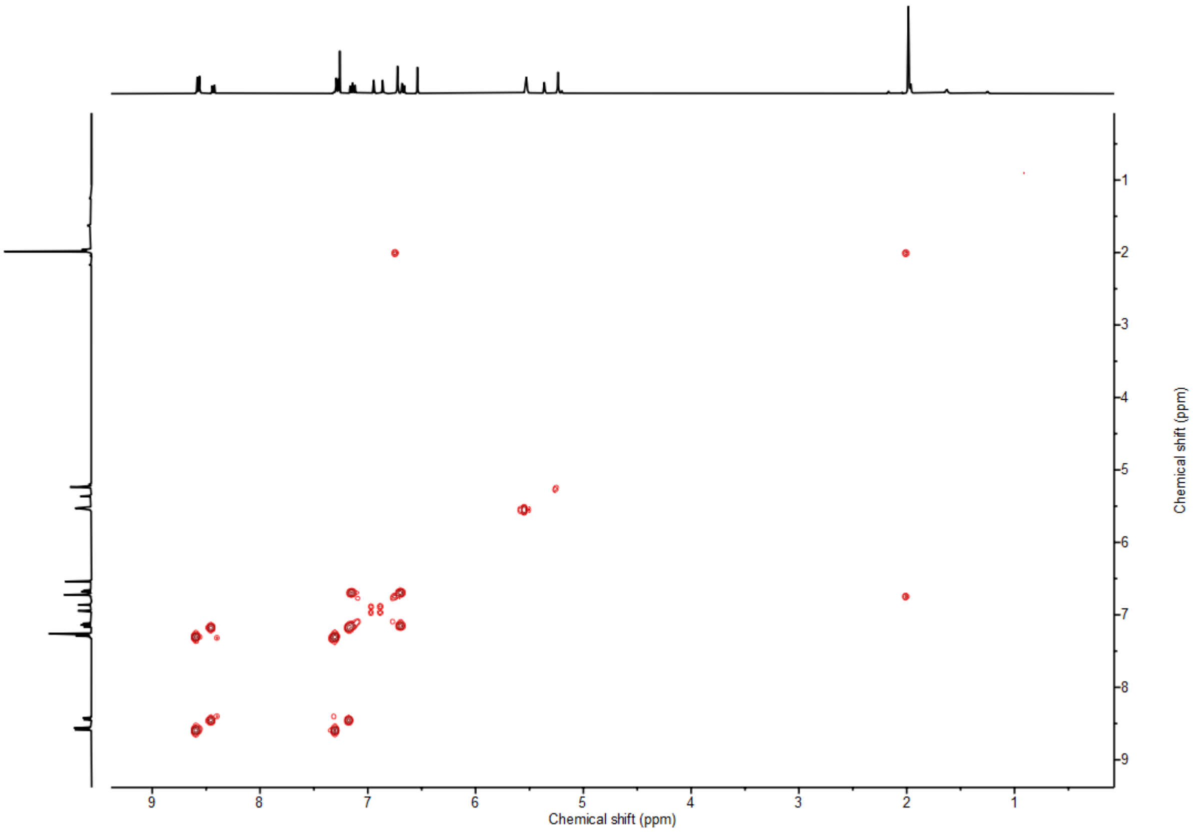

Figure S96 COSY NMR of ABB ($CDCl_3$)

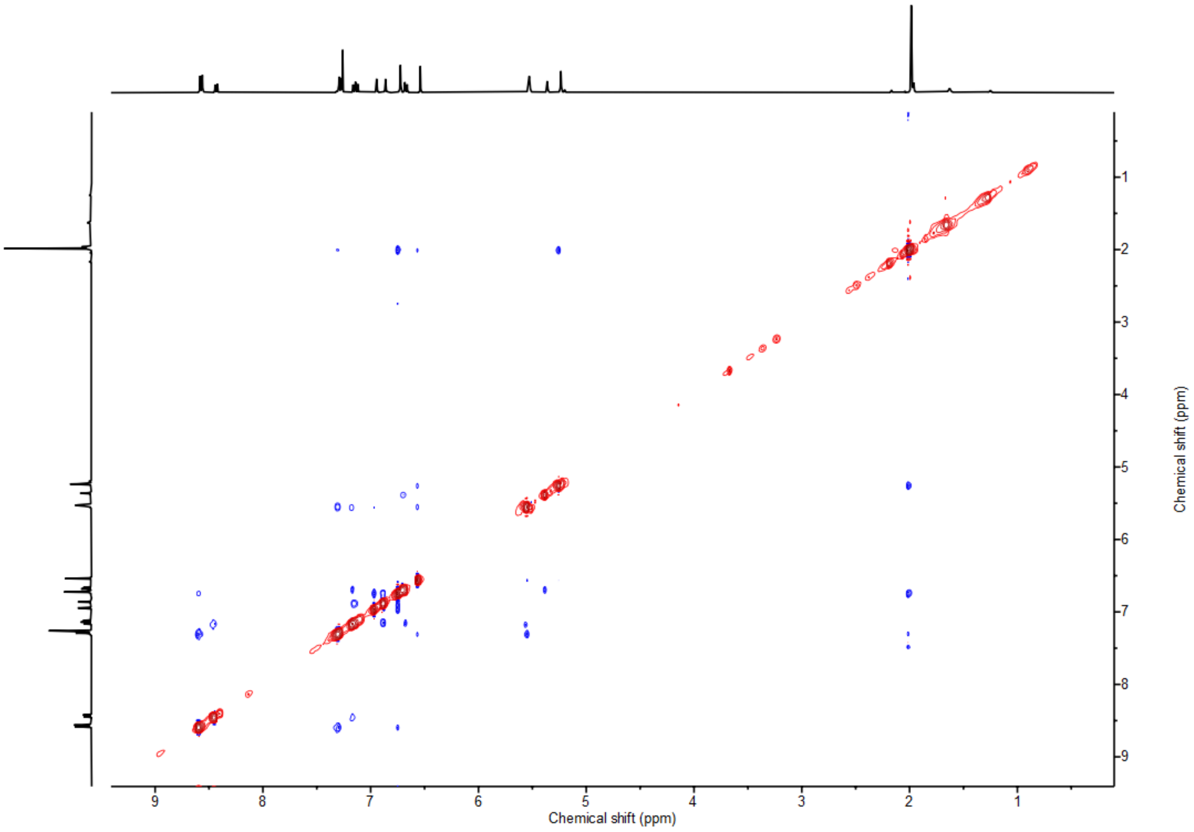

Figure S97 NOESY NMR of ABB ($CDCl_3$)

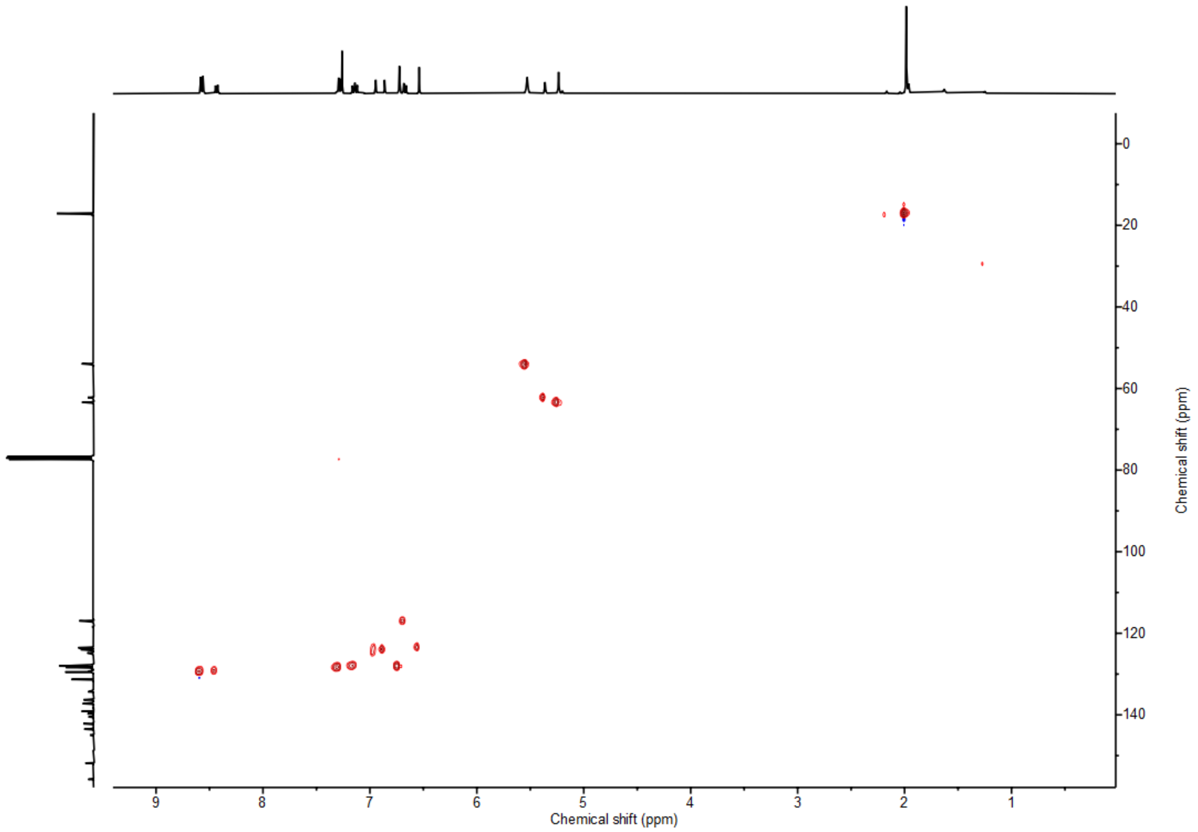

Figure S98 HSQC of ABB ($CDCl_3$)

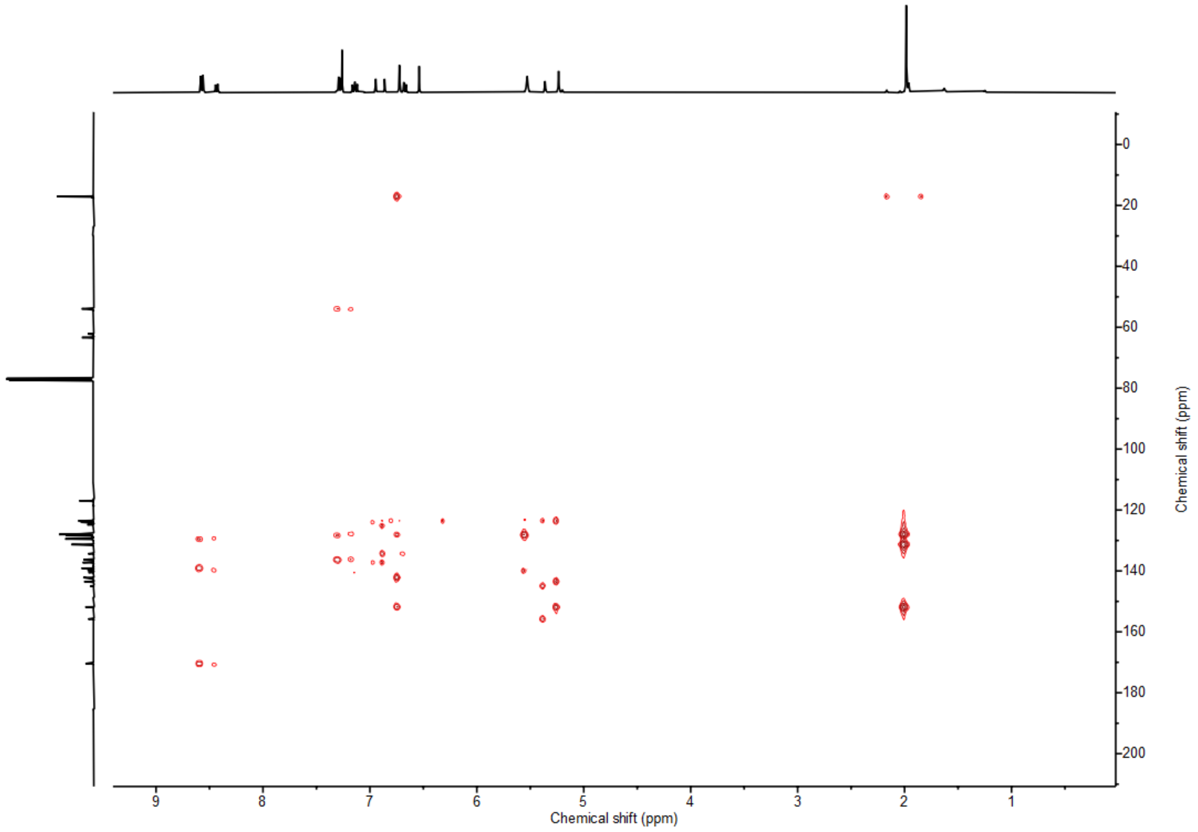

Figure S99 HMBC of ABB ($CDCl_3$)

Synthesis of cage ABC

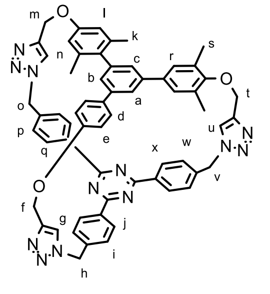

CuI (4.9 mg, 0.25 mmol, 0.5 eq.) was suspended in toluene (75 mL) and heated to 75 °C under N_2 atmosphere. DBU (0.25 mL, 0.85 mmol, 34 eq.) was added to this and stirred for 15 minutes. A solution of **2**$^{\mathbf{ABC}}$ (26.2 mg, 0.05 mmol, 1.0 eq.) and **1** (23.6 g, 0.05 mmol, 1.0 eq.) in 1:3 degassed THF/toluene (4 mL) was then added to the catalytic solution over 18 h via syringe pump. Once addition was complete, the solution was stirred at for 48 h at 110 °C. After the solvent was removed *in vacuo*, the residue was dissolved in CH_2Cl_2 (40 mL). The organic phase was washed with brine (3 × 30 mL), dried ($MgSO_4$) and the solvent removed *in vacuo*. After purification by column chromatography on silica gel (0-3% gradient MeOH in CH_2Cl_2) the product was obtained as an off-white solid (26.5 mg, 52 %).

^{1}H NMR (600 MHz, $CDCl_3$, 298 K) δ: 8.67 (d, *J* = 8.4, 2H, H_j/H_q/H_x), 8.52 (d, *J* = 8.3 Hz, 2H, H_j/H_q/H_x), 8.46 (d, *J* = 8.4 Hz, 2H, H_j/H_q/H_x), 7.48 (d, *J* = 8.3 Hz, 2H, H_i/H_p/H_w), 7.37 (d, *J* = 8.3 Hz, 2H, H_i/H_p/H_w), 7.28 (d, *J* = 8.4 Hz, 2H, H_i/H_p/H_w), 7.26 – 7.24 (m, 1H, H_a), 7.22 – 7.14 (m, 3H, H_e, H_n), 7.07 (s, 1H, H_g), 6.85 (s, 2H, H_r), 6.83 (t, *J* = 1.6 Hz, 1H, H_c), 6.81 (t, *J* = 1.6 Hz, 1H, H_b), 6.73 – 6.63 (m, 4H, H_u, H_d, $H_{l[out]}$), 5.88 (d, *J* = 2.6 Hz, 1H, $H_{l[in]}$), 5.75 – 5.34 (m, 8H, H_m, H_o, H_v), 5.28 – 5.20 (m, 2H, H_m), 5.11 (s, 2H, H_t), 2.00 – 1.91 (m, 9H, H_t, $H_{k[out]}$), 1.28 (s, 3H, $H_{k[in]}$).

^{13}C NMR (101 MHz, $CDCl_3$, 298 K) δ: 171.1, 170.8, 170.8, 156.9, 156.4, 152.9, 146.5, 145.6, 143.9, 141.1, 140.1, 140.0, 139.9, 139.5, 138.4, 137.5, 136.8, 136.8, 136.5, 136.2, 135.1, 133.9, 131.6, 129.9, 129.4, 129.3, 128.9, 128.6, 128.2, 127.5, 127.4, 126.8, 126.3, 123.2, 123.1, 122.5, 122.4, 117.8, 116.1, 114.7, 65.0, 64.4, 62.8, 54.3, 54.0, 29.7, 21.1, 21.0, 16.7.

HR-ESI-MS *m/z* = 999.4198 $[M+H]^+$ calc. 999.4207.

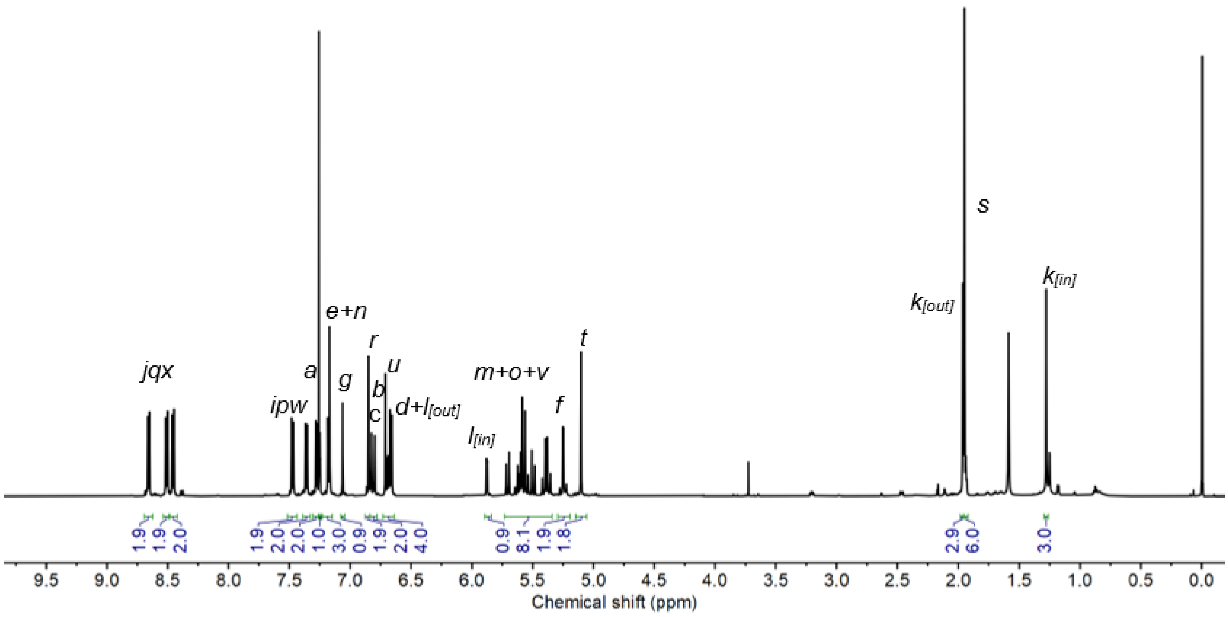

Figure S100 ^{1}H NMR of ABC (600 MHz, $CDCl_3$)

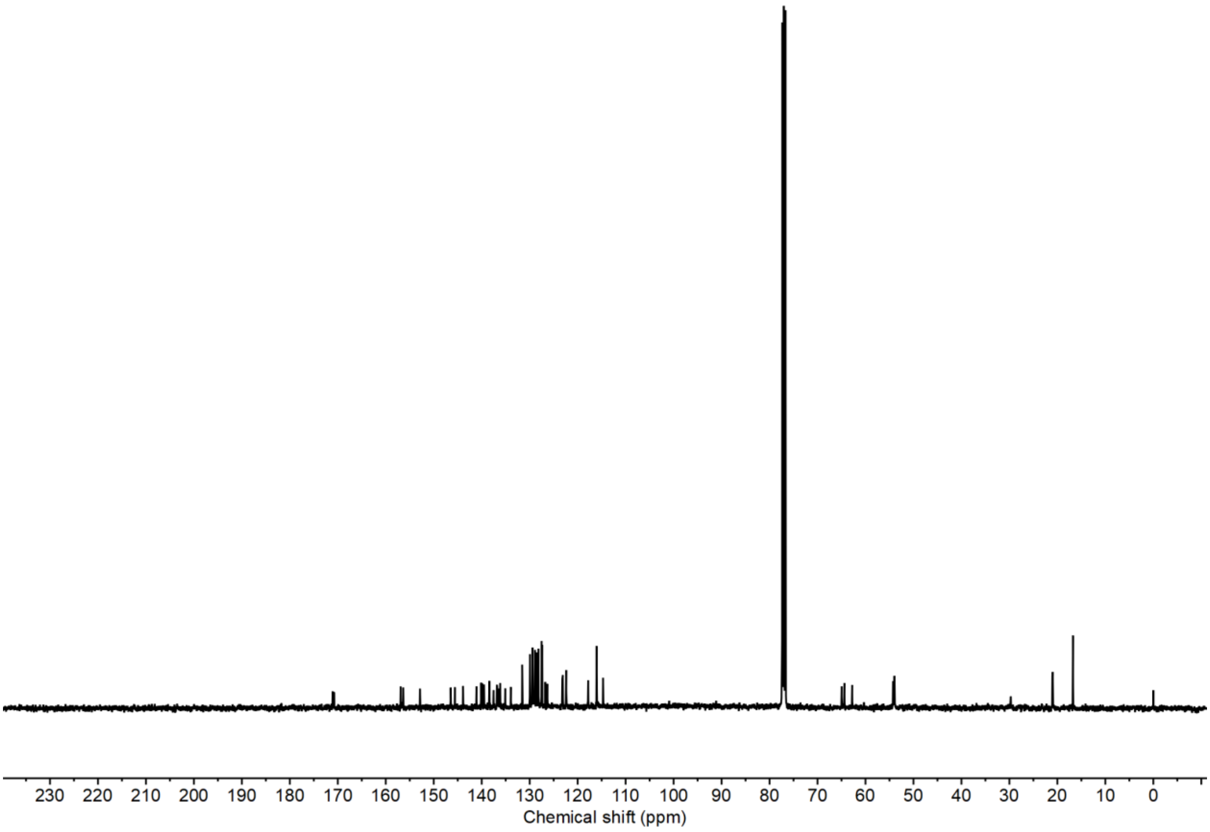

Figure S101 ^{13}C NMR of ABC (101 MHz, $CDCl_3$)

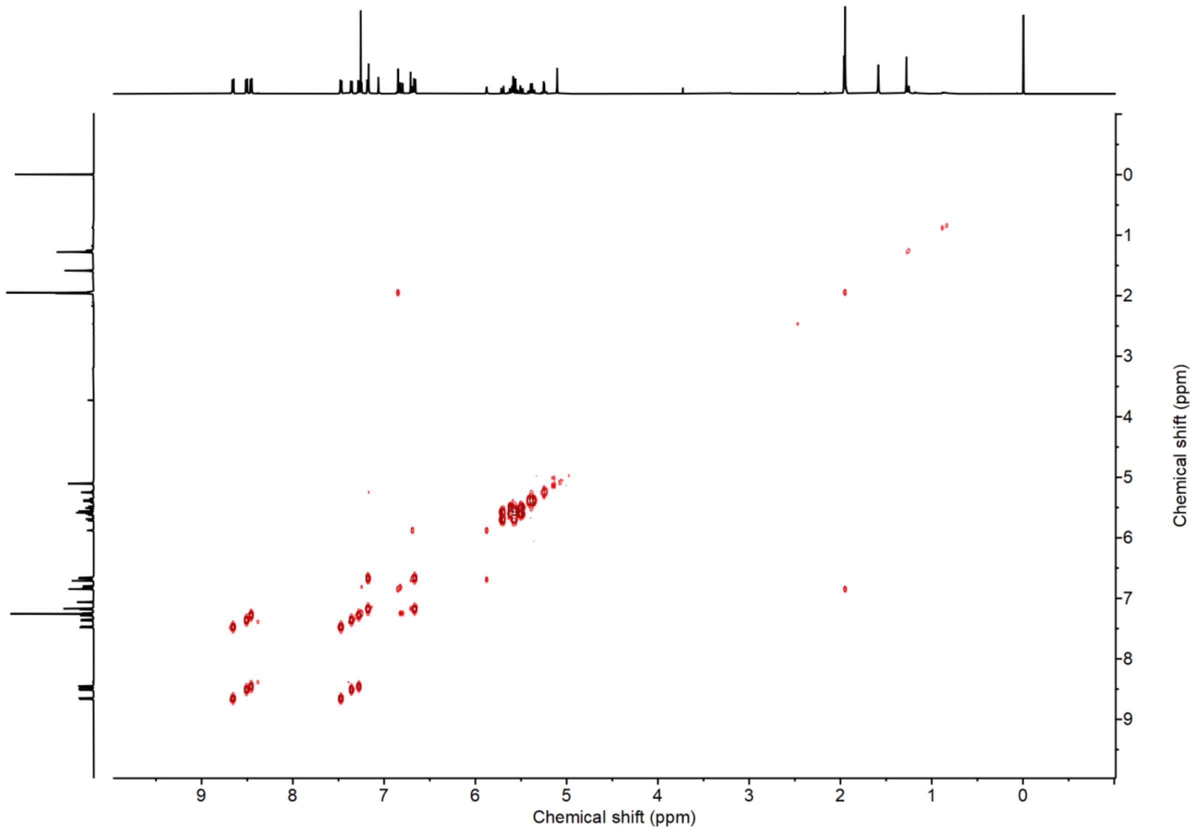

Figure S102 COSY NMR of ABC (600 MHz, $CDCl_3$)

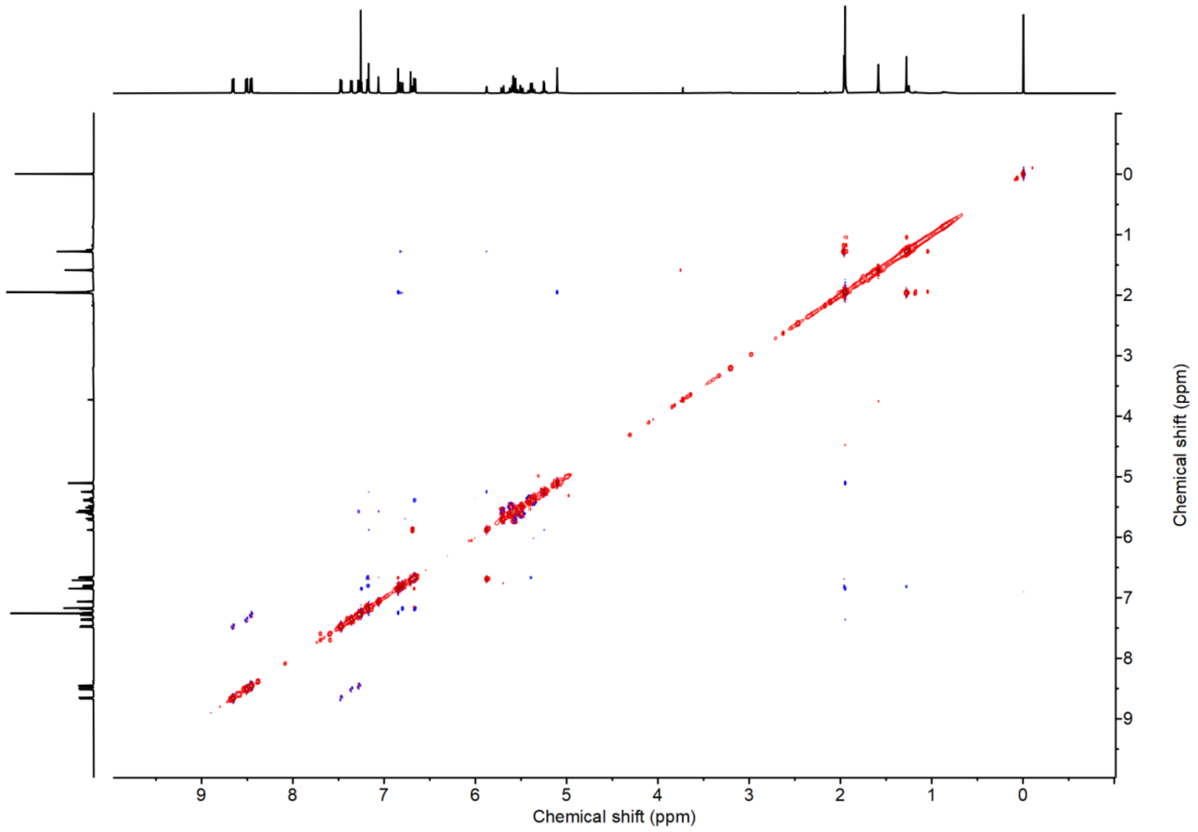

Figure S103 NOESY NMR of ABC (600 MHz, $CDCl_3$)

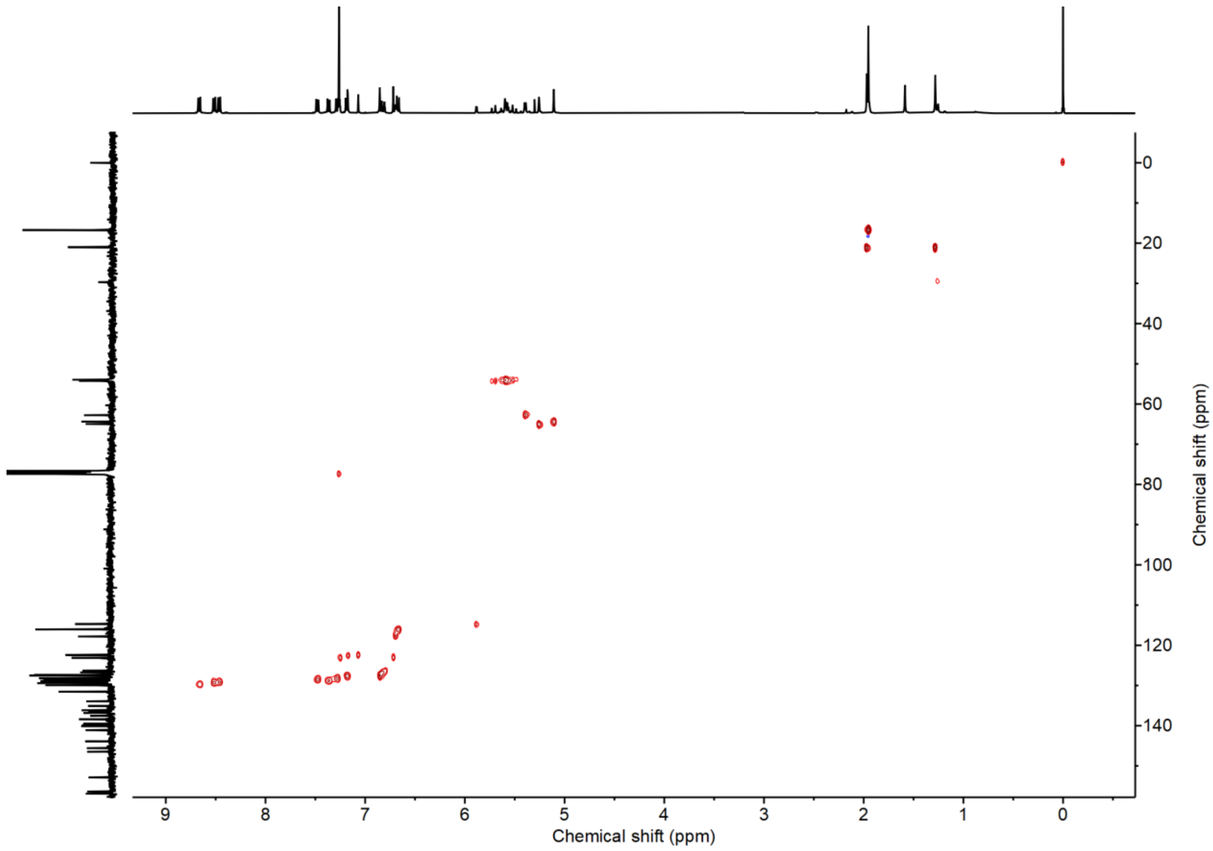

Figure S104 HSQC NMR of ABC ($CDCl_3$)

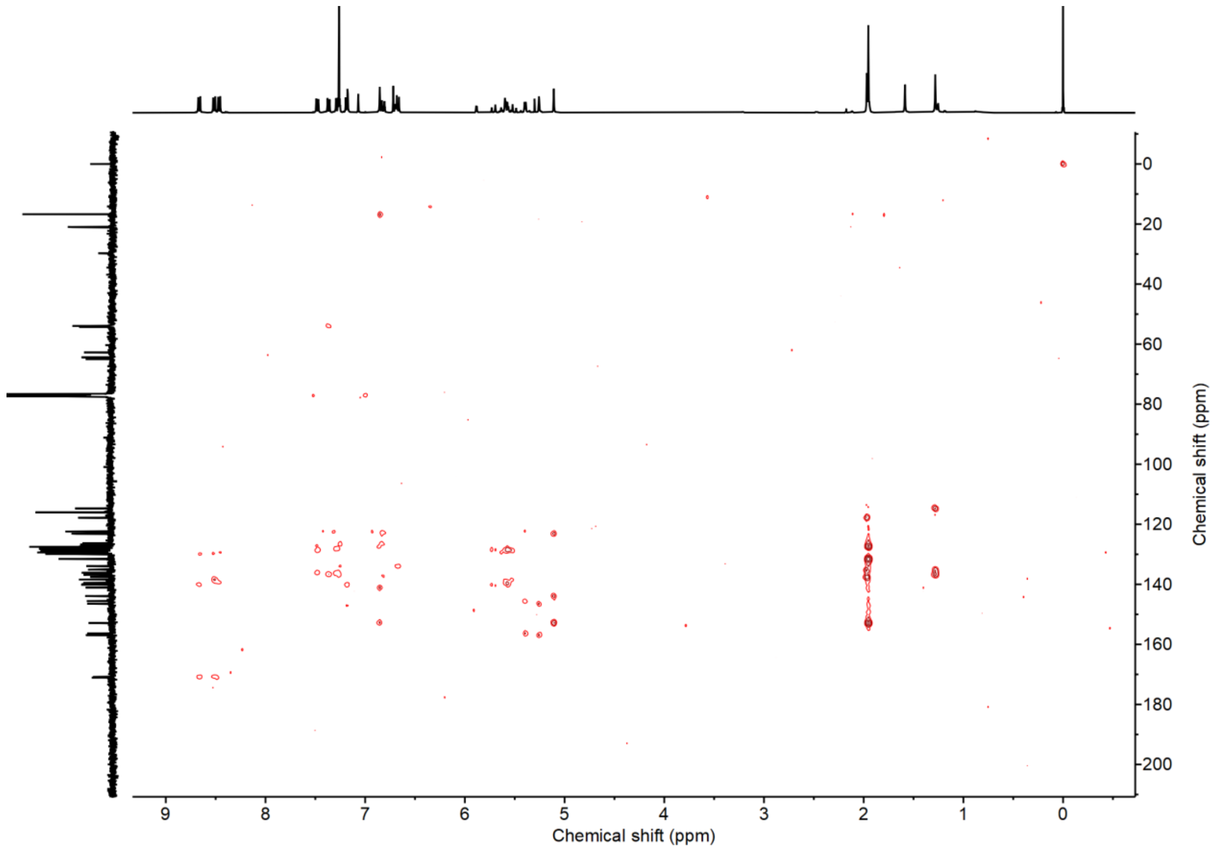

Figure S105 HMBC NMR of ABC ($CDCl_3$)

S3. Single crystal X-ray diffraction

Sample Preparation

Single crystals of **BBB** and **ABB** were grown by vapour diffusion of hexane into a solution of the cage in 1,2-dichloroethane. Single crystals of **AAA**·pyrene were grown by vapour diffusion of hexane in a 1,2-dichloroethane solution of a 1:1 mixture of **AAA** and pyrene.

BBB or **ABC** (2.7 mmol, 1 eq.) was dissolved in CH_2Cl_2 (1 mL) and DMF (0.5 mL) with heat and sonication. A layer of MeOH (2 mL) was carefully placed on top of this solution as a buffer layer. AgBF4 solution in MeOH (15.9 mM, 0.2 mL, 3.2 mmol, 1.2 eq.) was layered on top. This was covered from light and allowed to stand for 1 week, after which the white crystals had formed.

CCC or **ABB** (2.7 mmol, 1 eq.) was dissolved in CH_2Cl_2 (1 mL) and MeOH (0.5 mL) with heat and sonication. A layer of MeOH (2 mL) was carefully placed on top of this solution as a buffer layer. AgBF4 solution in MeOH (15.9 mM, 0.2 mL, 3.2 mmol, 1.2 eq.) was layered on top. This was covered from light and allowed to stand for 1 week, after which the white crystals had formed.

Data Collection and Analysis

The datasets for **AAA**, **ABB** and **BUF-2** were measured on a Rigaku XtaLAB Synergy diffractometer using a HyPix detector. The dataset for **BBB** was measured on an Agilent SuperNova diffractometer using an Atlas detector. These data collections were driven and processed, and absorption corrections were applied using CrysAlisPro.[S1] The datasets for **BUF-1**, **BUF-3**, and **BUF-4** were measured at the Diamond Light Source, Beamline I19-1, using a Dectris PILATUS 2M detector.[S2] These data set were processed and absorption corrections were applied using DIALS 3, XIA2 and AIMLESS.[S3]

Using OLEX2,[S4] the structures were solved using ShelXT,[S5] and were refined by a full-matrix least-squares procedure on F^2 in ShelXL.[S6] All non-hydrogen atoms were refined with anisotropic displacement parameters. In all structures the hydrogen atoms were fixed as riding models and the isotropic thermal parameters (U_{iso}) were based on the U_{eq} of the parent atom.

CCDC 2479243 (**AAA**), 2479244 (**ABB**), 2479245 (**BBB**), 2479246 (**BUF-3**), 2479247 (**BUF-1**), 2479248 (**BUF-4 (Dataset #1)**), 2479249 (**BUF-2**) and 2522046 (**BUF-4 (Dataset #2)**) contain the supplementary crystallographic data for this paper. These data can be obtained free of charge from The Cambridge Crystallographic Data Centre via www.ccdc.cam.ac.uk/data_request/cif.

AAA

$C_{69}H_{55}Cl_4N_{12}O_3$ (M =1242.05 g/mol): triclinic, space group P-1 (no. 2), a = 12.9478(2) Å, b = 14.8175(3) Å, c = 17.2190(3) Å, α = 81.640(2)°, β = 83.058(2)°, γ = 89.239(2)°, V = 3244.44(10) Å^3, Z = 2, T = 99.99(10) K, μ(Cu Kα) = 2.107 mm^{-1}, $Dcalc$ = 1.271 g/cm^3, 60895 reflections measured (5.226° ≤ 2Θ ≤ 156.492°), 12909 unique (R_{int} = 0.0392, R_{sigma} = 0.0317) which were used in all calculations. The final R_1 was 0.1083 (I > 2σ(I)) and wR_2 was 0.3701 (all data).

The structure contains half a molecule of pyrene and two units of 1,1-dichloroethane per cage. The pyrene and one of the dichloroethane molecules are located on inversion centres such that only half are crystallographically-unique. Another dichloroethane is disordered across an inversion centre at 50 % occupancy, by symmetry and another Cl(31)-C(301)-C(302)-Cl(32) / Cl(1A)-C(31A)-C(32A)-Cl(2A) is disordered over two positions at a refined percentage occupancy ratio of 67.0 (6) : 33.0 (6) respectively. Remaining highly disordered solvent molecules could not be refined, and a solvent mask has been used. The disordered solvent has resulted in residual electron density peaks and agreement factors that are higher than would be ideal. This was the best refinement that could be achieved.

ABB

$C_{65}H_{58}Cl_4N_{12}O_3$ (M =1197.03 g/mol): monoclinic, space group P2$_1$/c (no. 14), a = 10.8771(8) Å, b = 20.9934(8) Å, c = 28.5676(10) Å, β = 92.737(5)°, V = 6515.9(6) Å^3, Z = 4, T = 99.98(10) K, μ(Cu Kα) = 2.077 mm^{-1}, $Dcalc$ = 1.220 g/cm^3, 33613 reflections measured (7.492° ≤ 2Θ ≤ 136.49°), 11508 unique (R_{int} = 0.0579, R_{sigma} = 0.0651) which were used in all calculations. The final R_1 was 0.1736 (I > 2σ(I)) and wR_2 was 0.4803 (all data).

The methyl groups C(26) and C(27) / C(26A) and C(27A) are disordered over two arms of the cage at a refined percentage occupancy ratio of 66.9 (12) : 33.1 (12) respectively, such that in the major part C(26) is bonded to C(21) and C(27) to C(25) and in the minor part C(26A) is bonded to C(57) and C(27A) to C(61).

The structure contains two molecules of 1,1-dichloroethane per cage, one of which, Cl(21)-C(201)-C(202)-Cl(22) / Cl(1A)-C(21A)-C(22A)-Cl(2A) is disordered over two positions at a refined percentage occupancy ratio of 65.5 (6) : 34.5 (6) respectively. Remaining highly disordered solvent molecules could not be refined, and a solvent mask has been used. The disordered solvent has resulted in agreement factors that are higher than would be ideal. This was the best refinement that could be achieved.

BBB

$C_{67}H_{62}Cl_4N_{12}O_3$ (M =1225.08 g/mol): monoclinic, space group P2$_1$/c (no. 14), a = 10.94160(10) Å, b = 21.0745(3) Å, c = 28.5597(4) Å, β = 91.3600(10)°, V = 6583.69(14) Å^3, Z = 4, T = 119.99(10) K, μ(Cu Kα) = 2.066 mm^{-1}, $Dcalc$ = 1.236 g/cm^3, 67270 reflections measured (7.48° ≤ 2Θ ≤ 154.386°), 13720 unique (R_{int} = 0.0486, R_{sigma} = 0.0377) which were used in all calculations. The final R_1 was 0.0469 (I > 2σ(I)) and wR_2 was 0.1321 (all data).

The structure contains two molecules of 1,1-dichloroethane per cage, one of which, Cl(21)-C(201)-C(202)-Cl(22) / Cl(1A)-C(21A)-C(22A)-Cl(2A) is disordered over two positions at a refined percentage occupancy ratio of 89.4 (2) : 10.6 (2) respectively. Remaining highly disordered solvent molecules could not be refined, and a solvent mask has been used.

BUF-1

$C_{63}H_{54}AgBF_4N_{12}O_3$ (M =1221.86 g/mol): triclinic, space group P-1 (no. 2), a = 17.1564(6) Å, b = 17.1490(6) Å, c = 31.0993(15) Å, α = 93.570(4)°, β = 100.044(4)°, γ =

119.961(3)°, V = 7685.1(6) Å^3, Z = 4, T = 100.15 K, μ(Synchrotron) = 0.292 mm^{-1}, $Dcalc$ = 1.056 g/cm^3, 85951 reflections measured (2.664° ≤ 2Θ ≤ 45.002°), 21940 unique (R_{int} = 0.1820, R_{sigma} = 0.3143) which were used in all calculations. The final R_1 was 0.1153 (I > 2σ(I)) and wR_2 was 0.3138 (all data).

The structure contains two crystallographically-independent silver-cage units. This sample was very weakly diffracting and although the data were collected at the Diamond synchrotron the ratio of observed/unique reflections and resolution are still low and the R_{int} is high. The crystal demonstrated some signs of twinning, but it was not possible to successfully reduce the data as a twin. The structure includes a large solvent-accessible void containing BF_4 counter ions and solvent molecules that were so badly disordered that a solvent mask was employed. This was the best refinement that could be achieved.

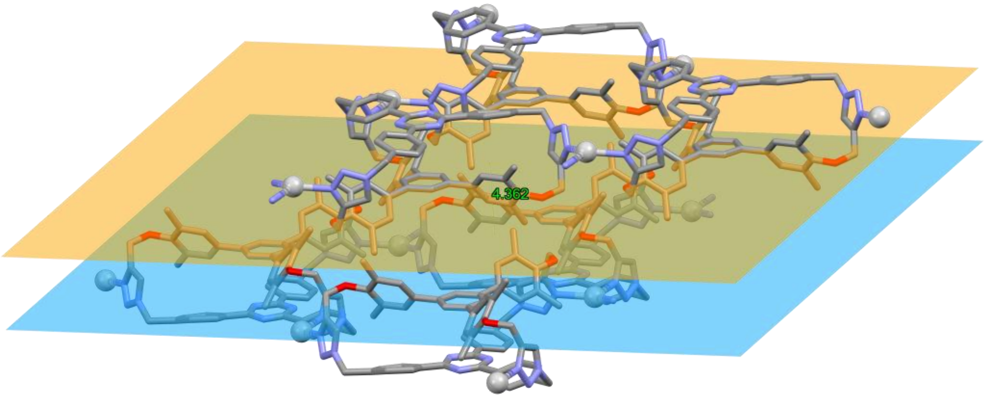

Figure S106 Distance in BUF-1 between planes defined by the central phenyl core of the cage faces.

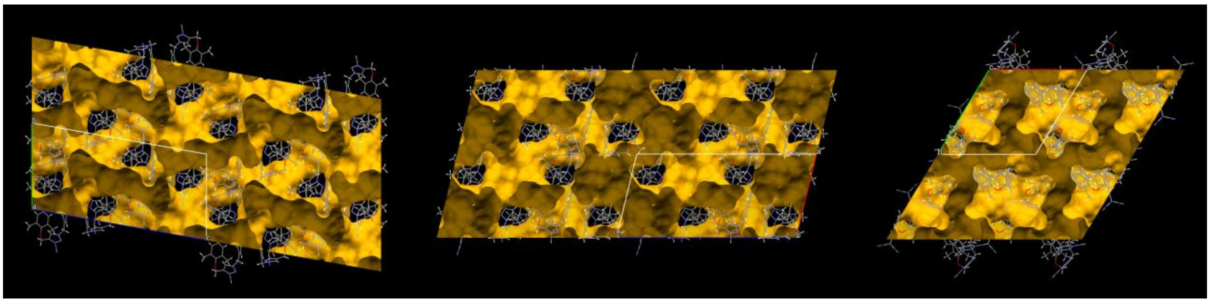

Figure S107 Visualisation of the void space in the cationic framework of BUF-1 viewed along the *a*, *b* and *c* axes (left to right). Prepared in Mercury; contact surface calculated using probe radius of 1.20 Å and approx. grid spacing of 0.20 Å.

BUF-2

$C_{63}H_{51}AgBF_4N_{12}O_3$ (M =1218.84 g/mol): trigonal, space group R-3 (no. 148), a = 14.1129(6) Å, c = 59.962(4) Å, V = 10342.9(11) Å^3, Z = 6, T = 99.99(10) K, μ(Cu Kα) = 2.838 mm^{-1}, $Dcalc$ = 1.174 g/cm^3, 23396 reflections measured (7.382° ≤ 2Θ ≤ 155.068°), 4615 unique (R_{int} = 0.1399, R_{sigma} = 0.0516) which were used in all calculations. The final R_1 was 0.1440 (I > 2σ(I)) and wR_2 was 0.4162 (all data).

There are 3-fold rotation axes running through both the silver atom and the centre of the cage

such that only one third of each is crystallographically-unique. The BF_4 anion is located on a 3-fold rotation axis running through B(1), F(1) and F(1A). The fluorine atoms F(1), F(2) / F(1A) and F(2A) are disordered over two positions each at 50 : 50 percentage occupancy ratio, by symmetry. The structure also contains highly disordered solvent molecules which could not be refined, and a solvent mask has been used. The disordered solvent has resulted in agreement factors that are higher than would be ideal. This was the best refinement that could be achieved.

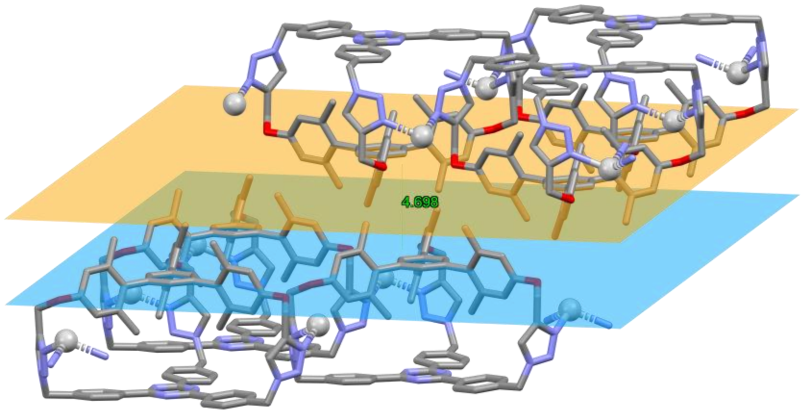

Figure S108 Distance in BUF-2 between planes defined by the central phenyl core of the cage faces.

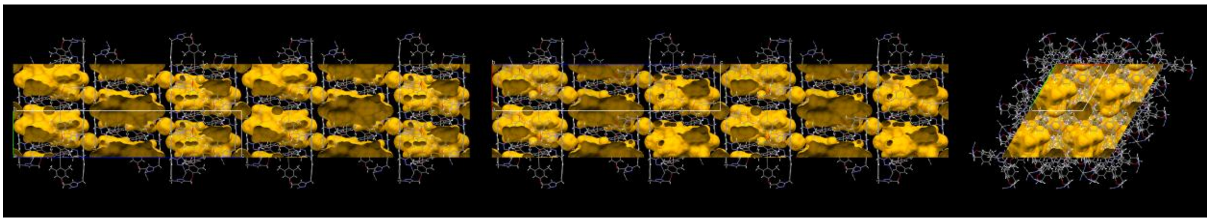

Figure S109 Visualisation of the void space in the cationic framework of BUF-2 viewed along the *a*, *b* and *c* axes (left to right). Prepared in Mercury; contact surface calculated using probe radius of 1.20 Å and approx. grid spacing of 0.20 Å.

BUF-3

$C_{61}H_{50}AgBF_4N_{12}O_3$ (M =1193.81 g/mol): monoclinic, space group C2/c (no. 15), a = 27.7998(6) Å, b = 18.1840(3) Å, c = 27.9594(6) Å, β = 102.579(2)°, V = 13794.5(5) Å^3, Z = 8, T = 100.00 K, μ(Synchrotron) = 0.324 mm^{-1}, $Dcalc$ = 1.150 g/cm^3, 92625 reflections measured (2.614° ≤ 2Θ ≤ 51.006°), 14110 unique (R_{int} = 0.0626, R_{sigma} = 0.0432) which were used in all calculations. The final R_1 was 0.1002 (I > 2σ(I)) and wR_2 was 0.3453 (all data).

The methyl groups C(26) and C(27) / C(26A) and C(27A) are disordered over two arms of the cage at a refined percentage occupancy ratio of 51.4 (11) : 48.6 (11) respectively, such that in the major part C(26) is bonded to C(21) and C(27) to C(25) and in the minor part C(26A) is bonded to C(57) and C(27A) to C(61). The BF_4 anion is disordered over two positions at a percentage occupancy ratio of 50 : 50. Remaining highly disordered solvent molecules could not be refined, and a solvent mask has been used.

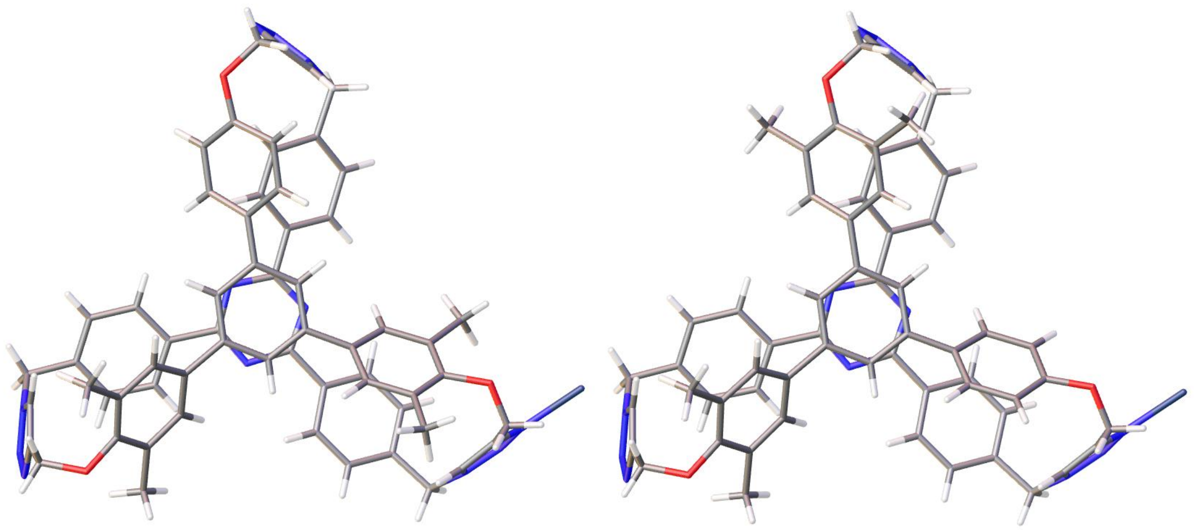

Figure S110 The two rotationally disordered ABB components in BUF-3.

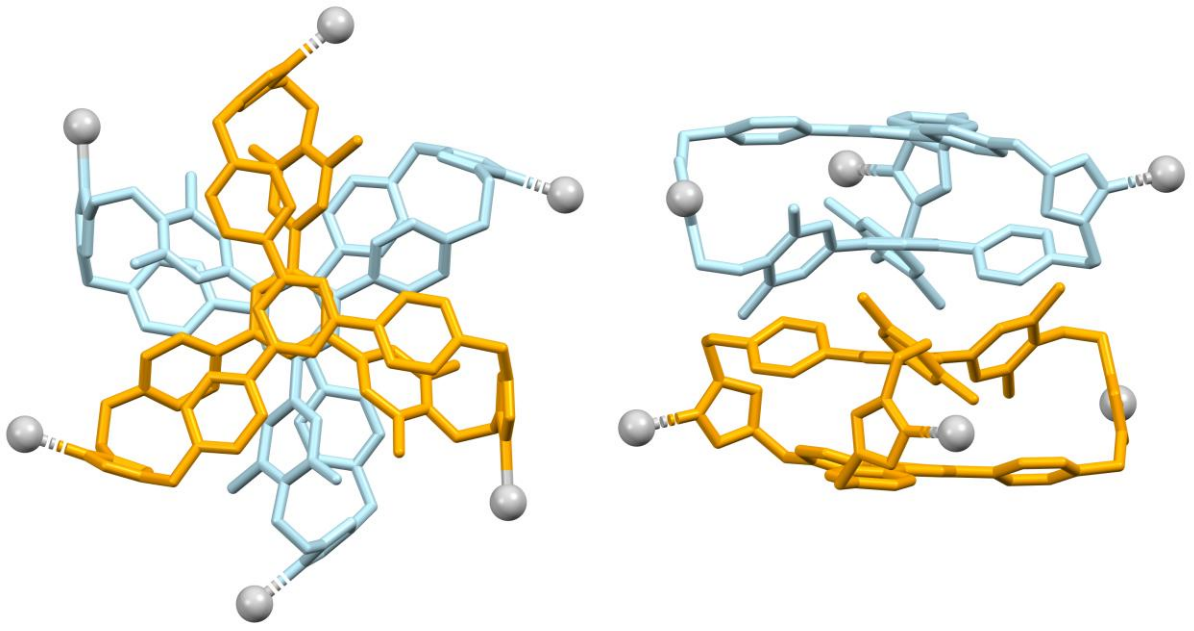

Figure S111 Interdigitation between ABB cages in BUF-3.

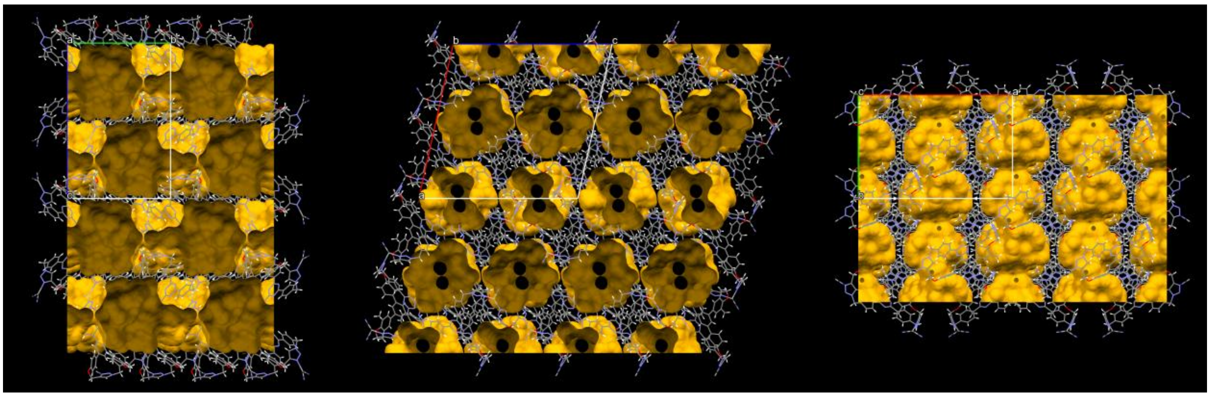

Figure S112 Visualisation of the void space in the cationic framework of BUF-3 viewed along the *a*, *b* and *c* axes (left to right). Prepared in Mercury; contact surface calculated using probe radius of 1.20 Å and approx. grid spacing of 0.20 Å.

BUF-4 (Dataset #1)

$C_{61}H_{50}AgBF_4N_{12}O_3$ (*M* =1193.81 g/mol): monoclinic, space group C2/c (no. 15), *a* = 45.4256(3) Å, *b* = 15.0574(2) Å, *c* = 31.4274(2) Å, *β* = 133.0460(10)°, *V* = 15709.4(3) Å^3, *Z* = 8, *T* = 100.15 K, μ(Synchrotron) = 0.285 mm^{-1}, *Dcalc* = 1.010 g/cm^3, 30007 reflections measured (2.878° ≤ 2Θ ≤ 45.004°), 10350 unique (R_{int} = 0.0377, R_{sigma} = 0.0568) which were used in all calculations. The final R_1 was 0.0790 (I > 2σ(I)) and wR_2 was 0.2757 (all data).

This sample was very weakly diffracting and although the data were collected at the Diamond synchrotron the resolution and completeness are still low. In the cage, the ring C(56)-C(61) / C(56A)-C(61A) is disordered over two positions and the methyl groups C(26) and C(27) / C(26A) and C(27A) are disordered over two arms of the cage, both at a refined percentage occupancy ratio of 60.9 (6) : 39.1 (6). The minor part of the disordered methyl groups are bonded to the minor part of the disordered ring. In the major part C(26) is bonded to C(22) and C(27) to C(24) and in the minor part C(26A) is bonded to C(58A) and C(27A) to C(60A). The BF_4 anion is disordered over two positions at a refined percentage occupancy ratio of 71.8 (8) : 28.2 (8). Highly disordered solvent molecules could not be refined, and a solvent mask has been used. This was the best refinement that could be achieved.

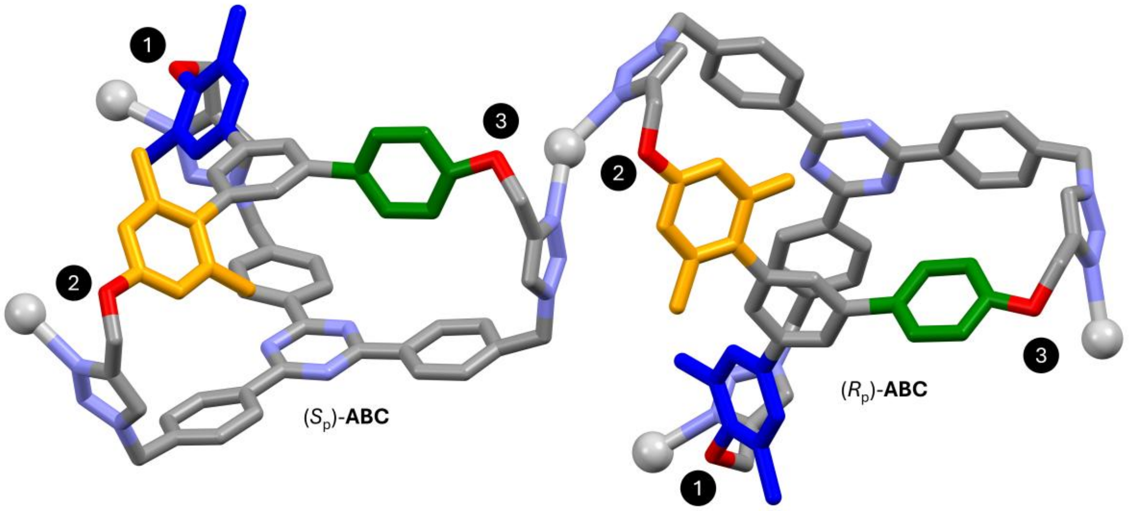

Figure S113 Assignment of chirality for ABC cage. Oxygen atom of the B arm assigned as the highest priority atom in the cage, followed by the C arm and A arm, respectively.

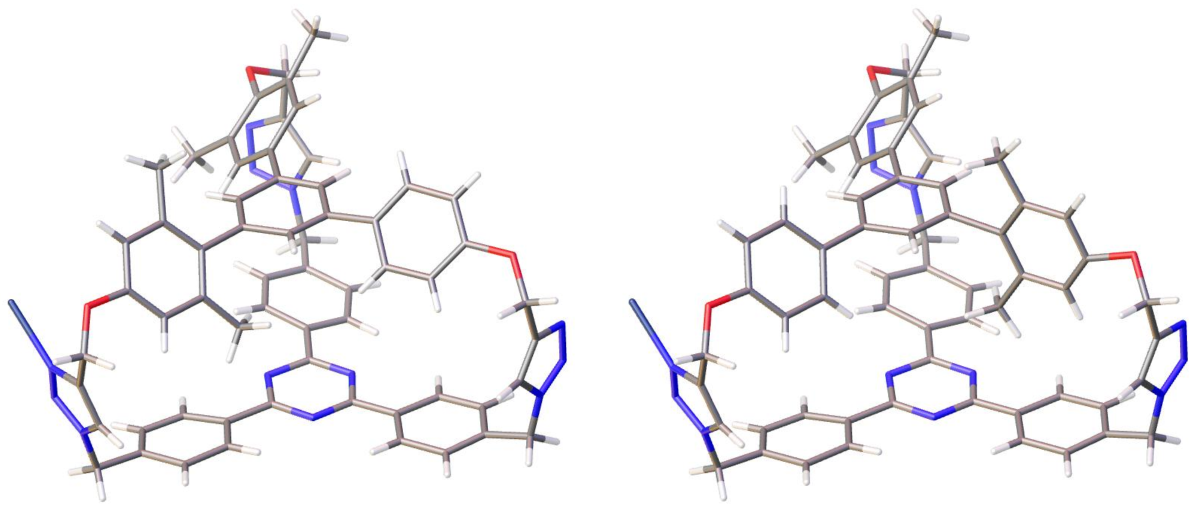

Figure S114 The two disordered ABC components in BUF-4: the (S_p)-ABC enantiomer (left) and the (R_p)-ABC enantiomer (right).

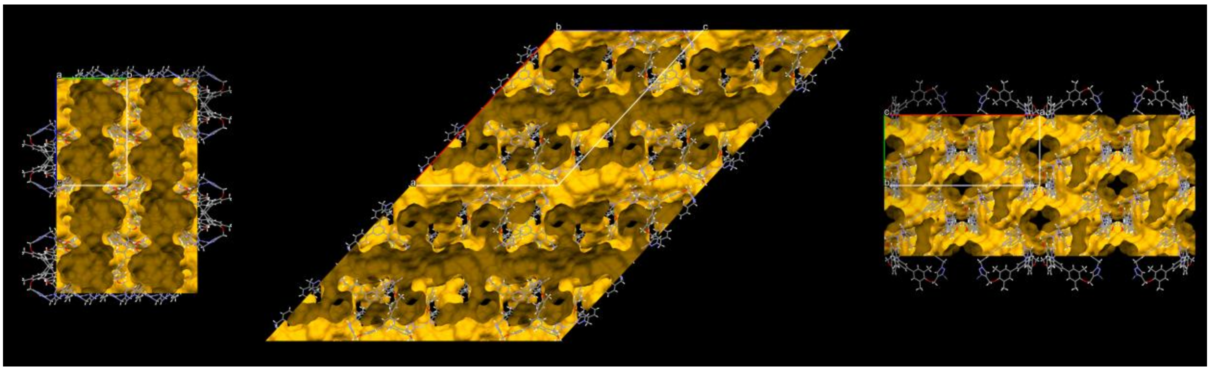

Figure S115 Visualisation of the void space in the cationic framework of BUF-4 viewed along the *a*, *b* and *c* axes (left to right). Prepared in Mercury; contact surface calculated using probe radius of 1.20 Å and approx. grid spacing of 0.20 Å.

BUF-4 (Dataset #2)

$C_{61}H_{50}AgBF_4N_{12}O_3$ (*M* =1193.81 g/mol): monoclinic, space group C2/c (no. 15), *a* = 45.5628(6) Å, *b* = 15.1910(3) Å, *c* = 31.6581(5) Å, *β* = 133.0200(10)°, *V* = 16020.2(5) Å^3, *Z* = 8, *T* = 100.15 K, μ(Synchrotron) = 0.279 mm^{-1}, *Dcalc* = 0.990 g/cm^3, 92199 reflections measured (2.856° ≤ 2Θ ≤ 46.084°), 12300 unique (R_{int} = 0.0460, R_{sigma} = 0.0451) which were used in all calculations. The final R_1 was 0.0991 (I > 2σ(I)) and wR_2 was 0.3255 (all data).

This sample was weakly diffracting and although the data were collected at the Diamond synchrotron the resolution is still low. In the cage, the ring C(56)-C(61) / C(56A)-C(61A) is disordered over two positions and the methyl groups C(26) and C(27) / C(26A) and C(27A) are disordered over two arms of the cage, both at a refined percentage occupancy ratio of 58.8 (7) : 41.2 (7). The minor part of the disordered methyl groups are bonded to the minor part of the disordered ring. In the major part C(26) is bonded to C(22) and C(27) to C(24) and in the minor part C(26A) is bonded to C(58A) and C(27A) to C(60A). The BF_4 anion is disordered over two positions at a refined percentage occupancy ratio of 58.7 (12) : 41.3 (12). Highly disordered solvent molecules could not be refined, and a solvent mask has been used. This was the best refinement that could be achieved.

S4. Powder X-ray diffraction

Coordination framework sample preparation:

Cage (10.8 mmol, 1 eq.) was dissolved in CH_2Cl_2 (1 mL) and MeOH (0.5 mL) with heat and sonication. A layer of MeOH (2 mL) was carefully placed on top of this solution as a buffer layer. $AgBF_4$ solution in MeOH (63.6 mM, 0.2 mL, 13.0 mmol, 1.2 eq.) was layered on top. This was covered from light and allowed to stand for 1 week, after which the white crystals had formed. The mother liquor was carefully decanted and the solids washed three times with diethyl ether and dried at 30 °C *in vacuo* in the dark.

N.B. For PXRD and TGA measurements, the coordination frameworks (CFs) are referred to as **CF^{AAA}**, **CF^{BBB}**, **CF^{CCC}**, **CF^{ABB}** and **CF^{ABC}** rather than as BUFs.

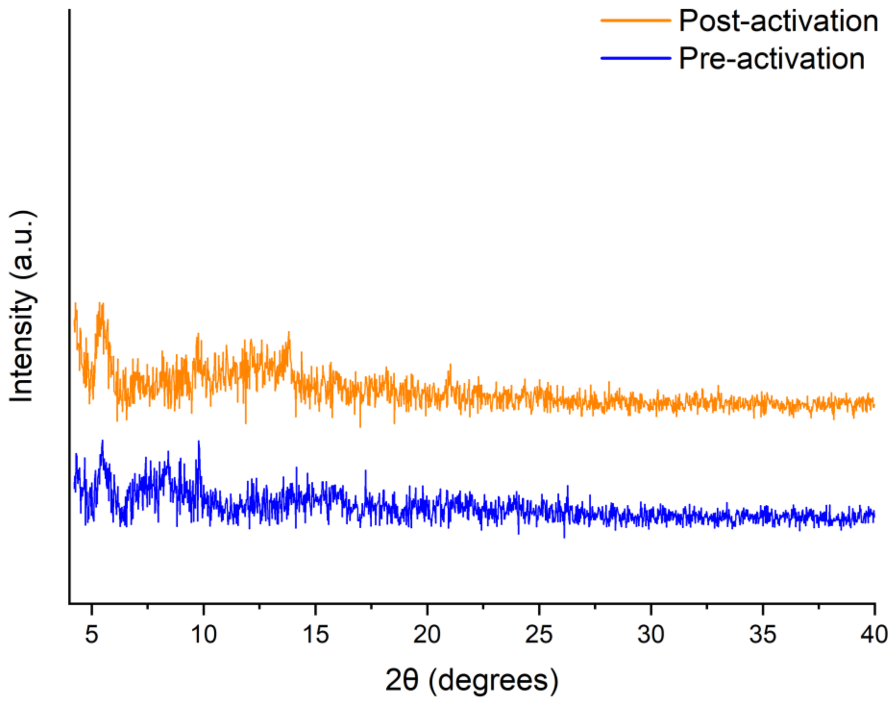

Figure S116 Stacked PXRD patterns for cage AAA pre-activation (*blue*) and post-activation (*orange*).

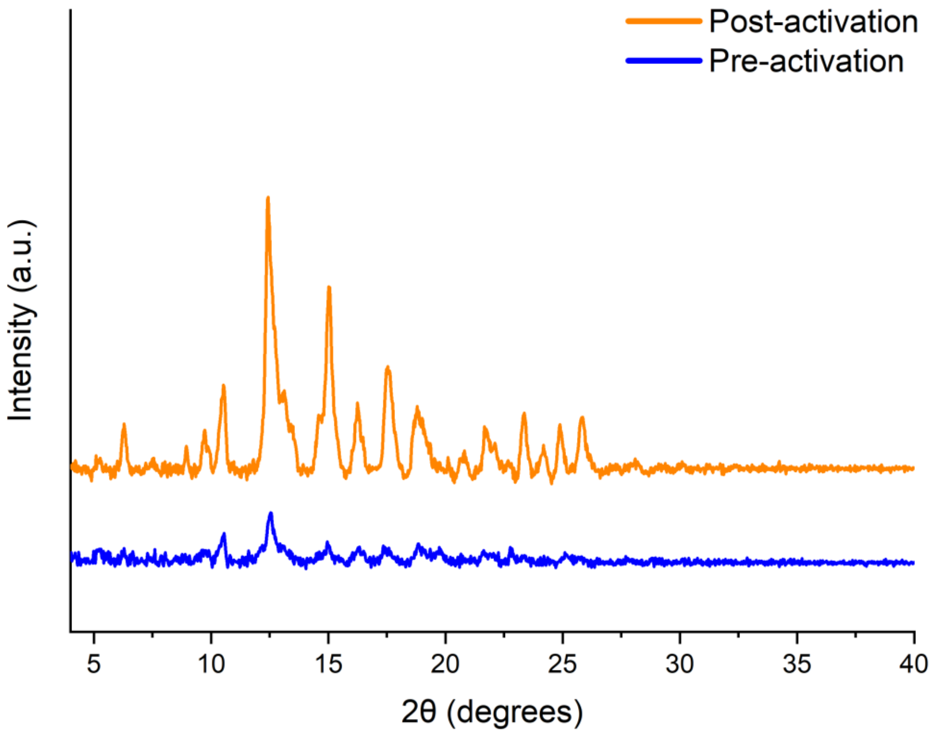

Figure S117 Stacked PXRD patterns for cage BBB pre-activation (*blue*) and post-activation (*orange*).

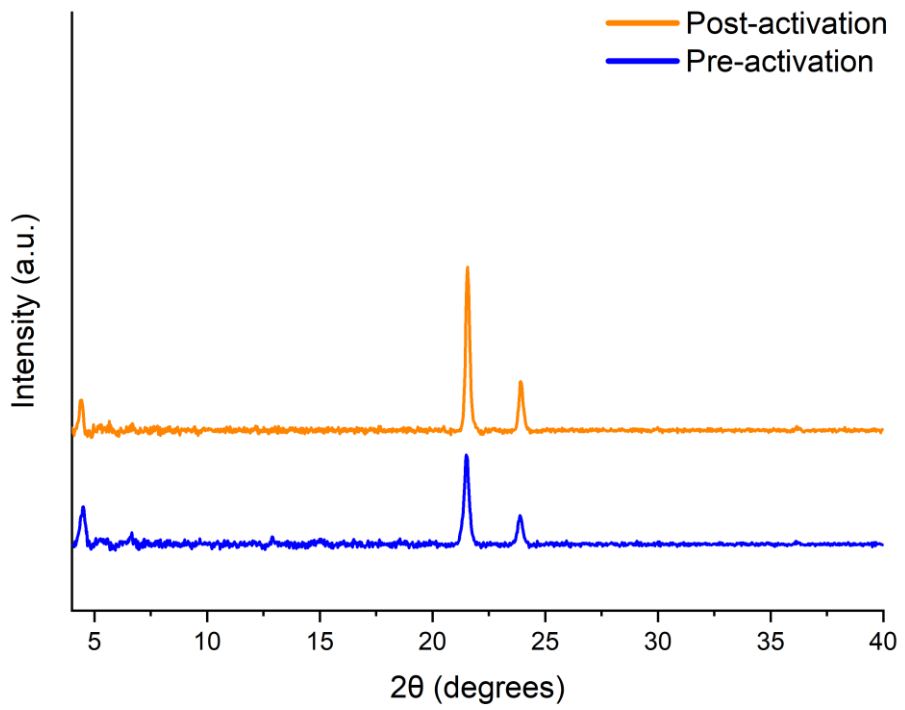

Figure S118 Stacked PXRD patterns for cage CCC pre-activation (*blue*) and post-activation (*orange*).

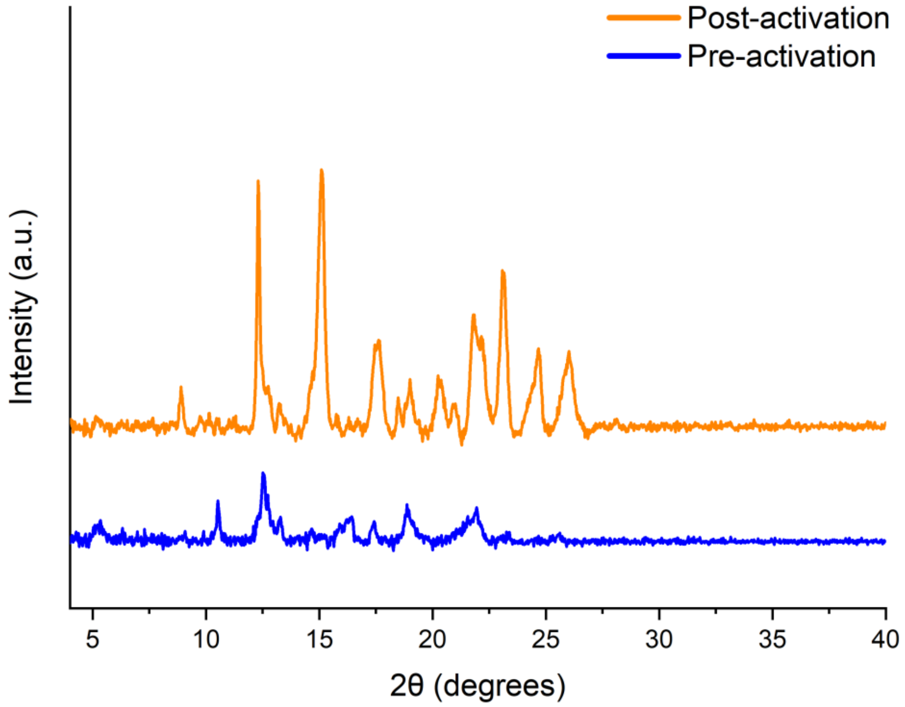

Figure S119 Stacked PXRD patterns for cage ABB pre-activation (*blue*) and post-activation (*orange*).

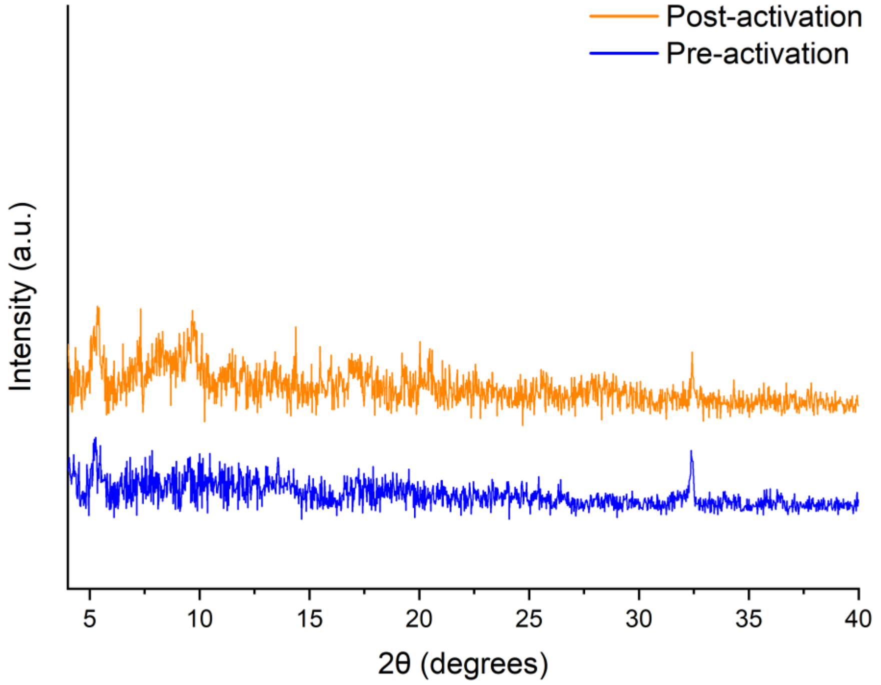

Figure S120 Stacked PXRD patterns for cage ABC pre-activation (*blue*) and post-activation (*orange*).

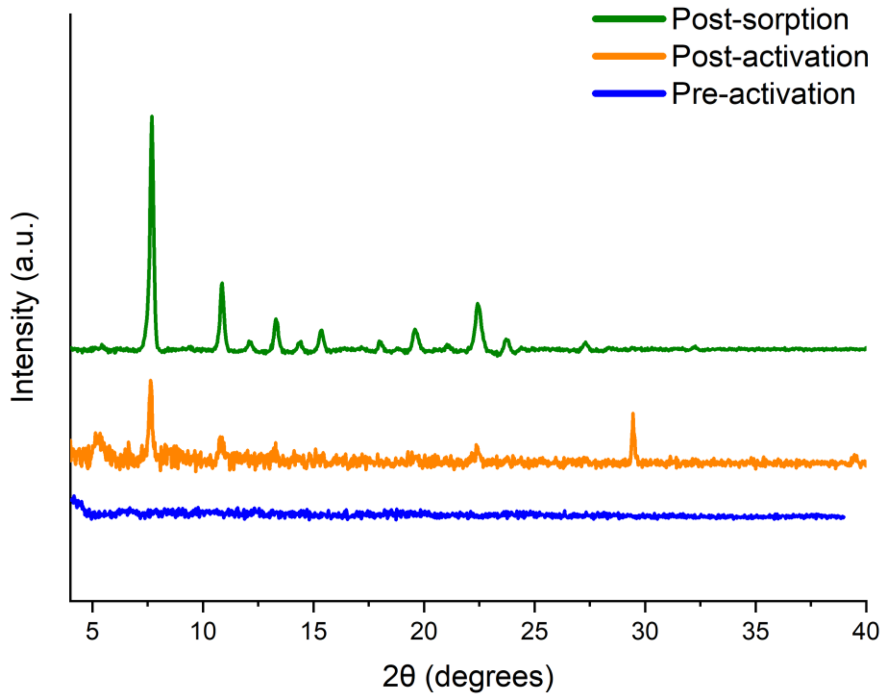

Figure S121 Stacked PXRD patterns for CF^{AAA} pre-activation (*blue*), post-activation (*orange*) and post-sorption (*green*).

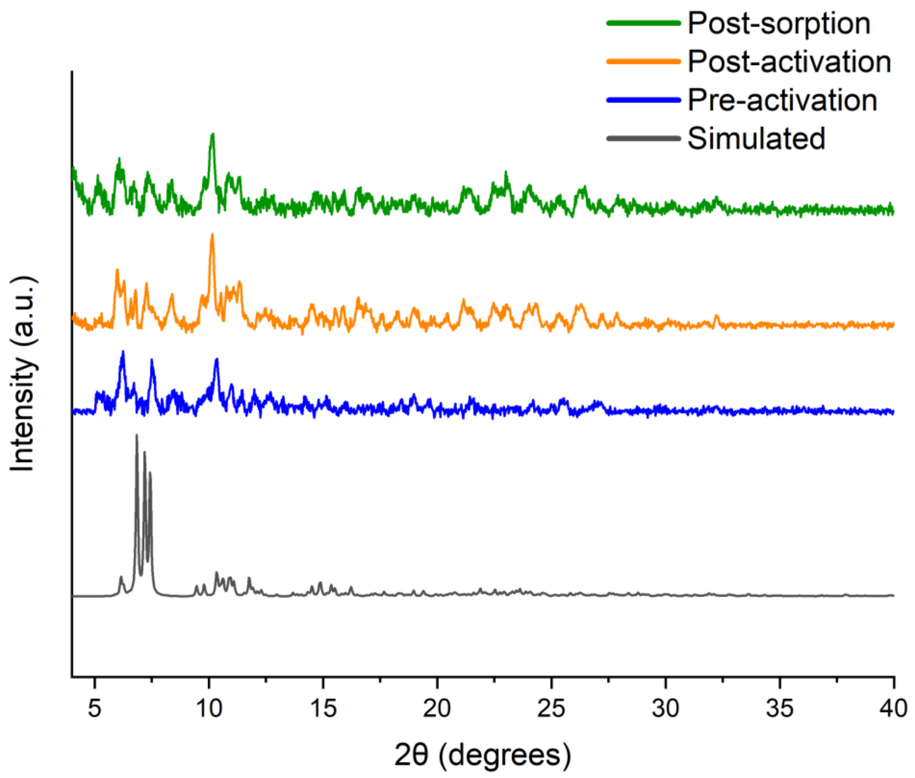

Figure S122 Stacked PXRD patterns for CF^{BBB} pre-activation (*blue*), post-activation (*orange*) and post-sorption (*green*). The simulated PXRD pattern (*black*) derived from the BUF-1 SCXRD data was also plotted for comparison.

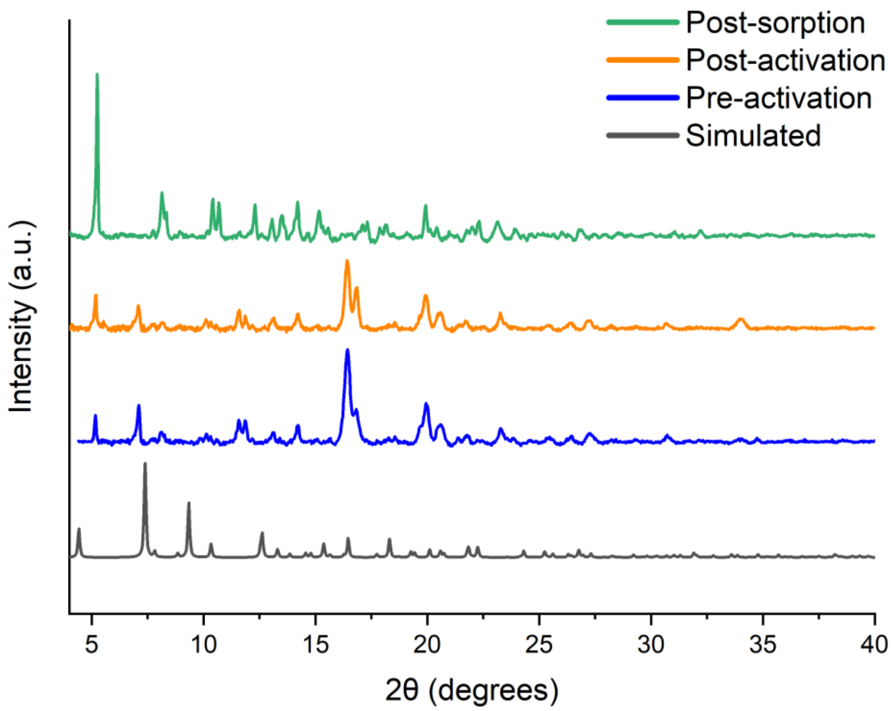

Figure S123 Stacked PXRD patterns for CF^{CCC} pre-activation (*blue*), post-activation (*orange*) and post-sorption (*green*). The simulated PXRD pattern (*black*) derived from the BUF-2 SCXRD data was also plotted for comparison.

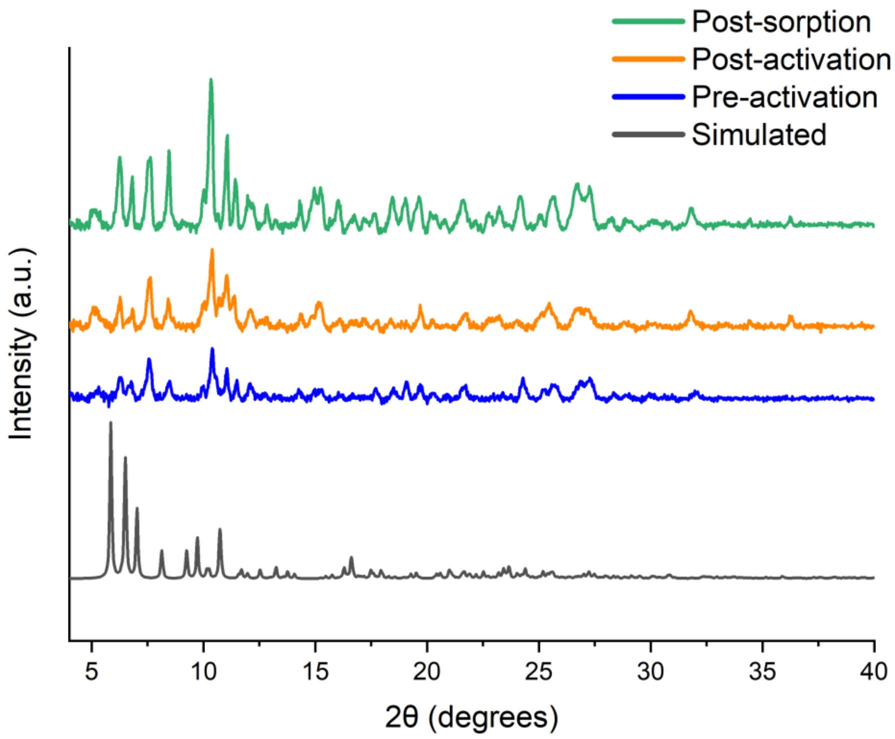

Figure S124 Stacked PXRD patterns for CF^{ABB} pre-activation (*blue*), post-activation (*orange*) and post-sorption (*green*). The simulated PXRD pattern (*black*) derived from the BUF-3 SCXRD data was also plotted for comparison.

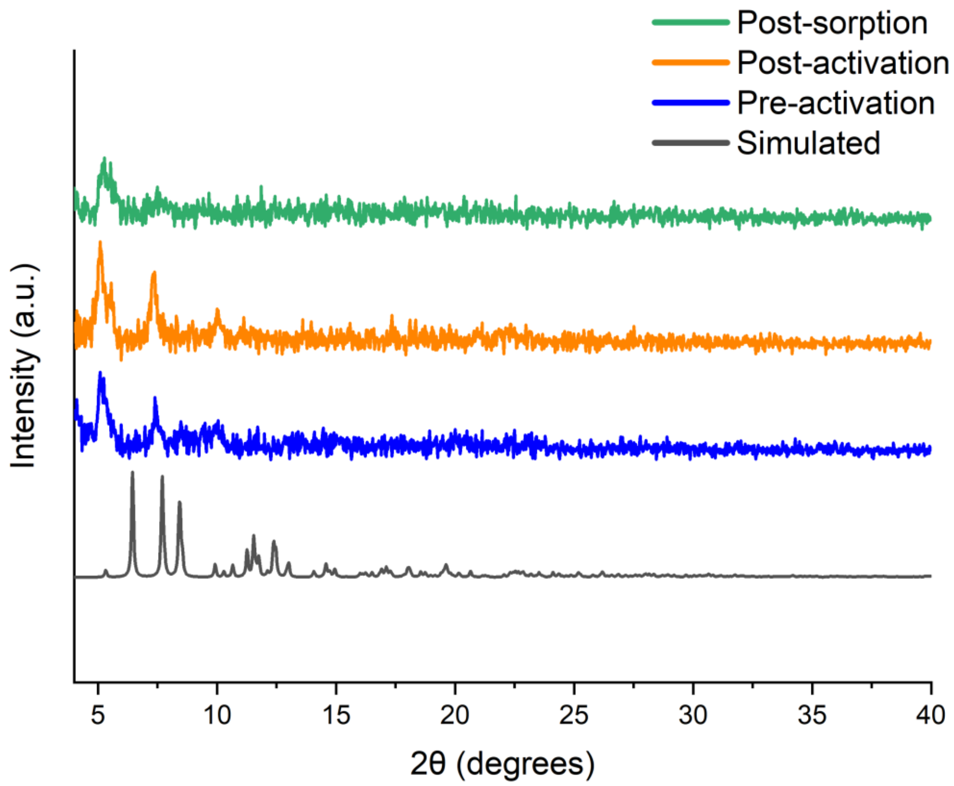

Figure S125 Stacked PXRD patterns for CF^{ABC} pre-activation (*blue*), post-activation (*orange*) and post-sorption (*green*). The simulated PXRD pattern (*black*) derived from the BUF-4 SCXRD data was also plotted for comparison.

S5. Thermogravimetric Analysis

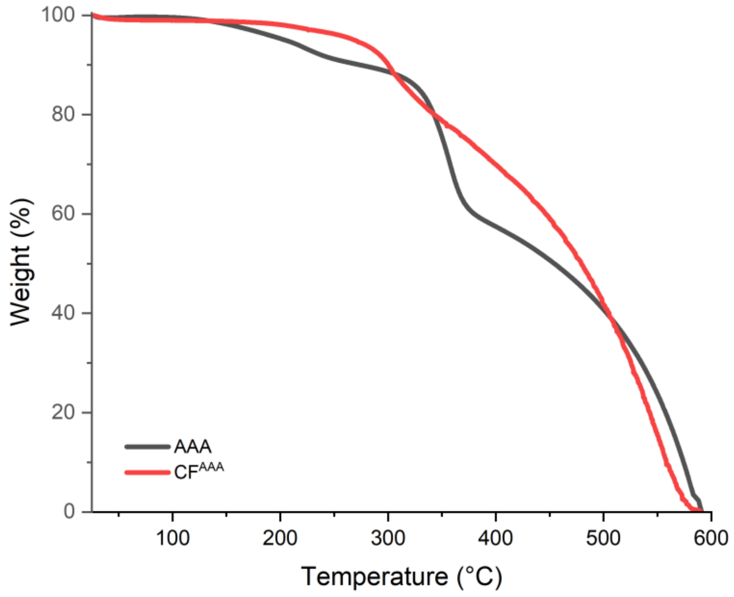

Figure S126 TGA curves of cage AAA (*black*) and its corresponding coordination framework CF^{AAA} (*red*). For AAA, an initial mass loss is observed (T_{onset} = 140 °C), followed by the onset of material decomposition (T_{onset} = 300 °C). In contrast, CF^{AAA} shows decomposition at temperatures above 300 °C (T_{onset} = 280 °C).

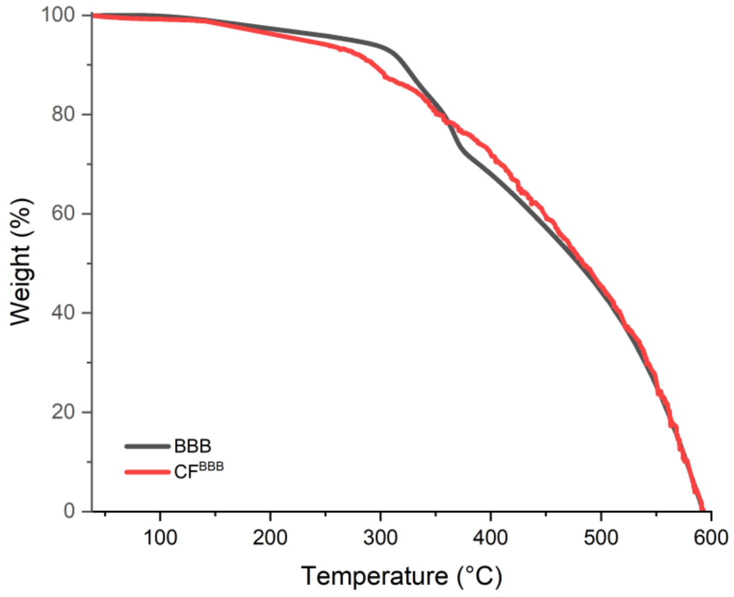

Figure S127 TGA curves of cage BBB (*black*) and its corresponding coordination framework CF^{BBB} (*red*). Both BBB and CF^{BBB} exhibit decomposition at similar temperatures (respectively T_{onset} = 306 °C and T_{onset} = 280 °C), with BBB showing a more pronounced mass-loss step as the temperature increases.

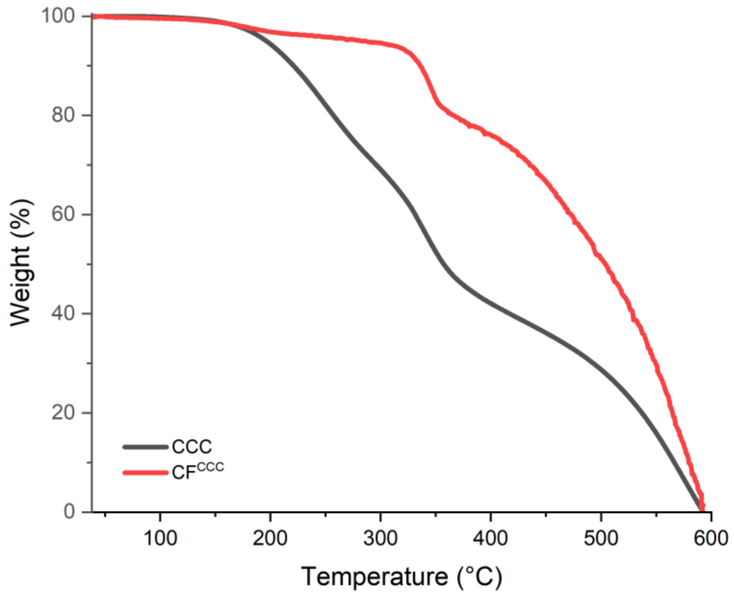

Figure S128 TGA curves of cage CCC (*black*) and its corresponding coordination framework CF^{CCC} (*red*). CCC decomposes above 200 °C (T_{onset} = 180 °C), while CF^{CCC} shows considerably greater thermal stability, with decomposition only occurring above 300 °C (T_{onset} = 320 °C).

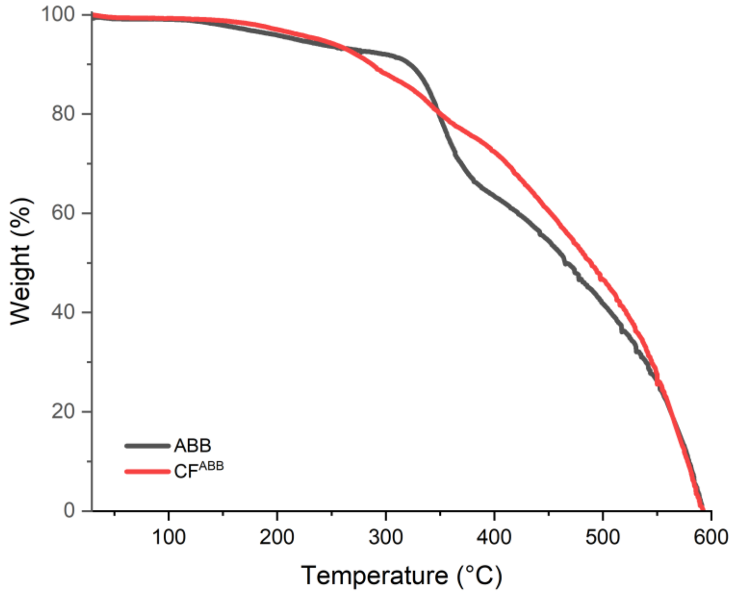

Figure S129 TGA curves of cage ABB (*black*) and its corresponding coordination framework CF^{ABB} (*red*). ABB undergoes significant decomposition above 300 °C (T_{onset} = 320 °C), while CF^{ABB} shows an initial mass loss (T_{onset} = 180 °C) that becomes more pronounced above 300 °C (T_{onset} = 260 °C).

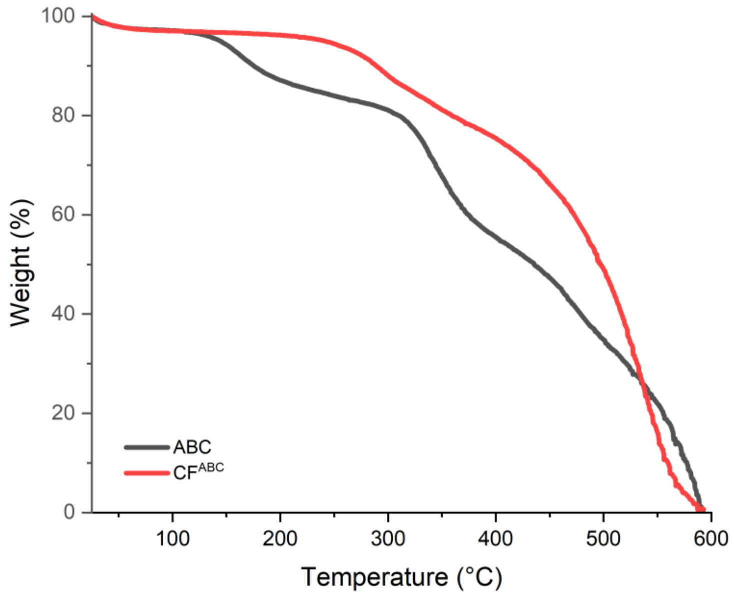

Figure S130 TGA curves of cage ABC (*black*) and its corresponding coordination framework CF^{ABC} (*red*). For ABC, a stepwise mass loss occurs between 130 and 400 °C before complete decomposition, whereas CF^{ABC} generally exhibits a more gradual mass loss as the temperature increases (T_{onset} = 260 °C).

S6. CO_2 Uptake Profiles

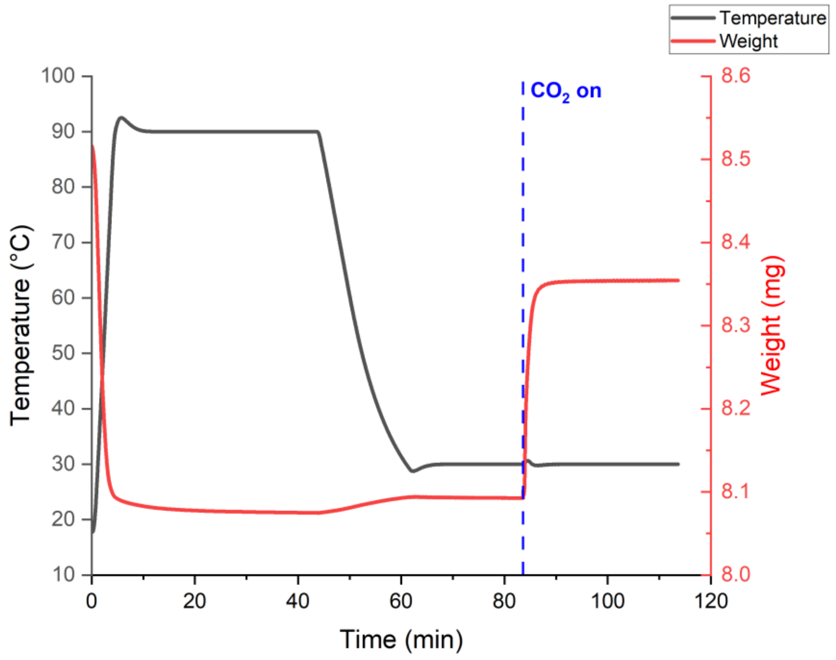

Figure S131 Gravimetric CO_2 uptake profile for CF[AAA] involving initial activation of the sample at 90 °C under N_2, followed by cooling and equilibration at 30 °C, and subsequent CO_2 purging (1 bar) until a plateau is reached.

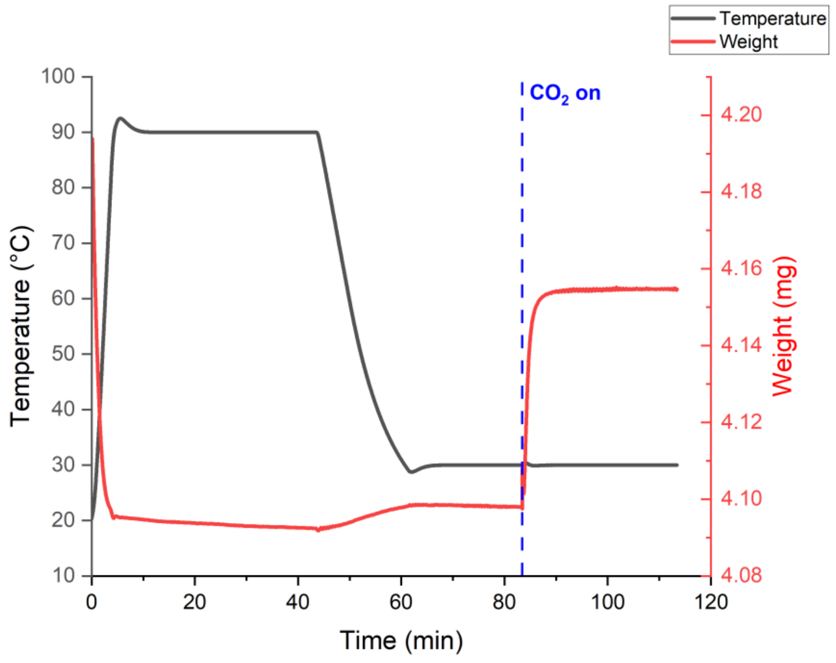

Figure S132 Gravimetric CO_2 uptake profile for CF[BBB] involving initial activation of the sample at 90 °C under N_2, followed by cooling and equilibration at 30 °C, and subsequent CO_2 purging (1 bar) until a plateau is reached.

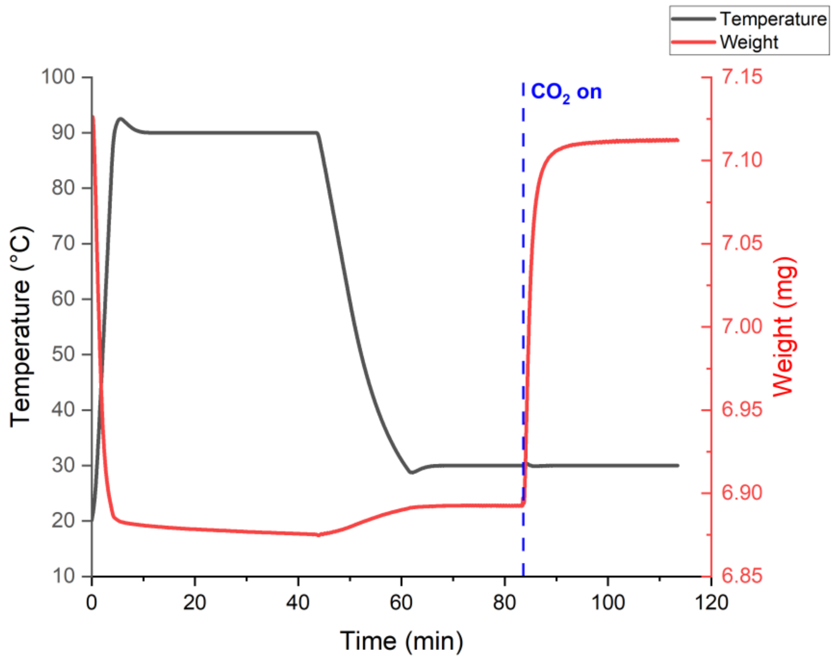

Figure S133 Gravimetric CO_2 uptake profile for CF^{CCC} involving initial activation of the sample at 90 °C under N_2, followed by cooling and equilibration at 30 °C, and subsequent CO_2 purging (1 bar) until a plateau is reached.

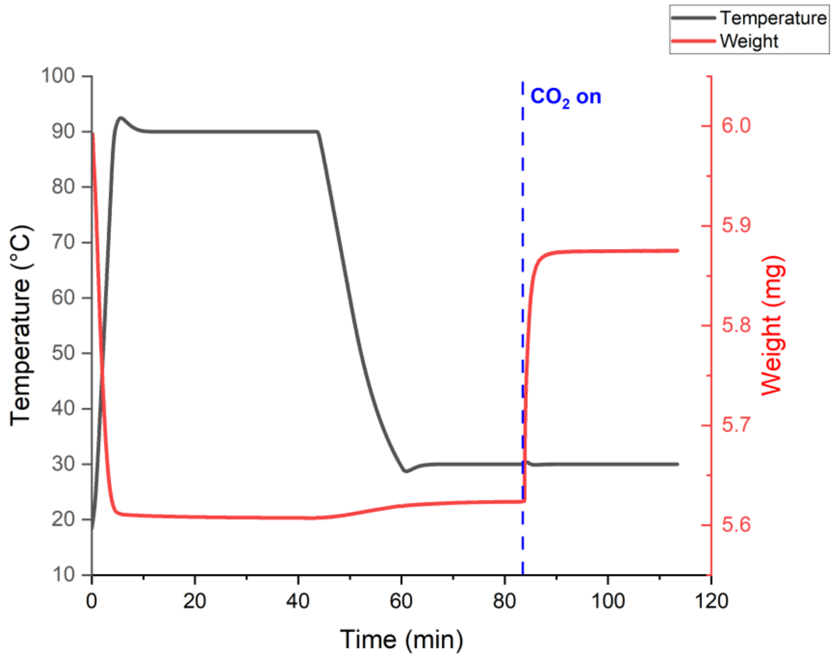

Figure S134 Gravimetric CO_2 uptake profile for CF^{ABB} involving initial activation of the sample at 90 °C under N_2, followed by cooling and equilibration at 30 °C, and subsequent CO_2 purging (1 bar) until a plateau is reached.

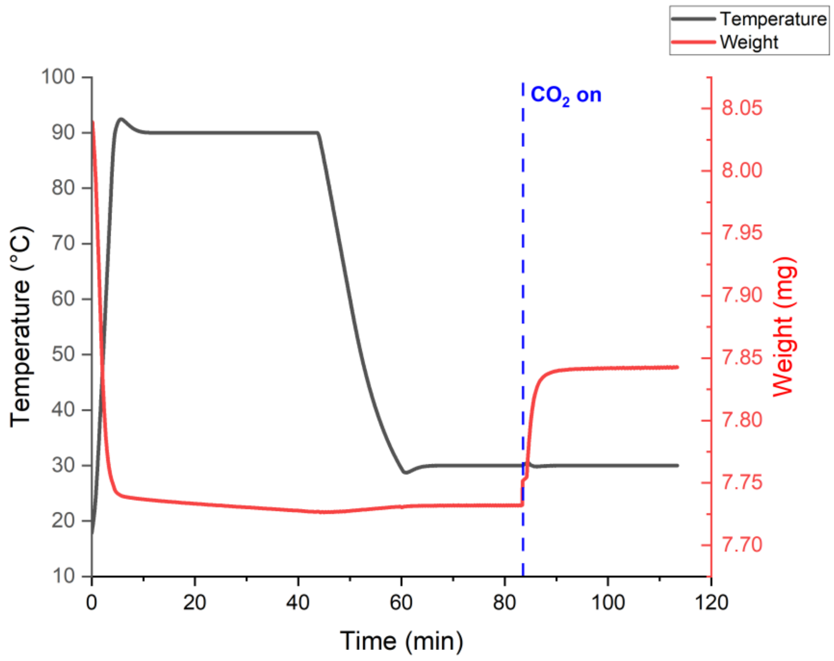

Figure S135 Gravimetric CO_2 uptake profile for CF^{ABC} involving initial activation of the sample at 90 °C under N_2, followed by cooling and equilibration at 30 °C, and subsequent CO_2 purging (1 bar) until a plateau is reached.

Table S1 Summary of CO_2 uptake calculations per gram of coordination framework sample (MW of CO_2 = 44.01 g/mol). Two measurements per sample were performed; mean values and standard deviations reported.

Sample	Mass (mg)	Mass (g)	CO_2 uptake (mg)	CO_2 uptake (mmol)	CO_2 uptake (mmol/g)	Average	Std Dev
**CF^{AAA}**	7.031	0.0070	0.18	0.0041	**0.58**	**0.64**	**0.08**
**CF^{AAA}**	8.512	0.0085	0.26	0.0059	**0.69**		
**CF^{BBB}**	3.924	0.0039	0.04	0.0009	**0.23**	**0.27**	**0.05**
**CF^{BBB}**	4.188	0.0042	0.06	0.0013	**0.30**		
**CF^{CCC}**	4.957	0.0050	0.15	0.0034	**0.69**	**0.69**	**0.01**
**CF^{CCC}**	7.117	0.0071	0.22	0.0050	**0.70**		
**CF^{ABB}**	3.296	0.0033	0.10	0.0022	**0.65**	**0.65**	**0.01**
**CF^{ABB}**	6.060	0.0061	0.17	0.0039	**0.64**		
**CF^{ABC}**	8.046	0.0080	0.11	0.0025	**0.31**	**0.29**	**0.02**
**CF^{ABC}**	6.924	0.0069	0.09	0.0019	**0.28**		

S7. Optical Microscopy Images

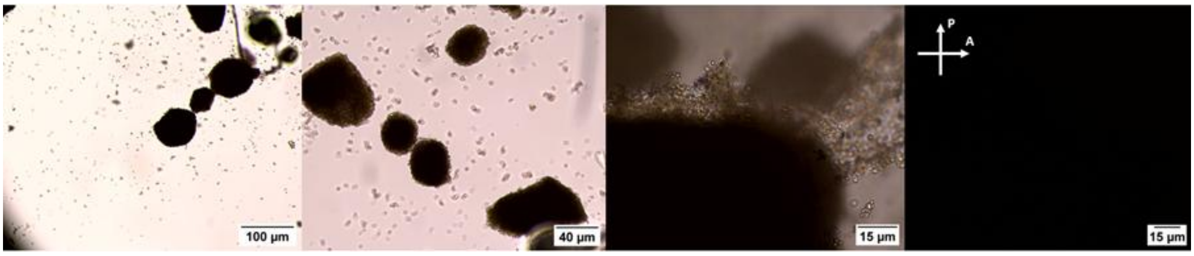

Figure S136 Optical micrographs of CF^{AAA} at 10×, 20×, and 50× magnification (*first, second and third figure*). The polarised optical micrograph at 50× (*last figure*) shows no birefringence, indicating an isotropic structure consistent with amorphous behaviour. This observation suggests the absence of long-range crystalline order at the microscale. "A" denotes the analyser direction; "P" denotes the polariser direction.

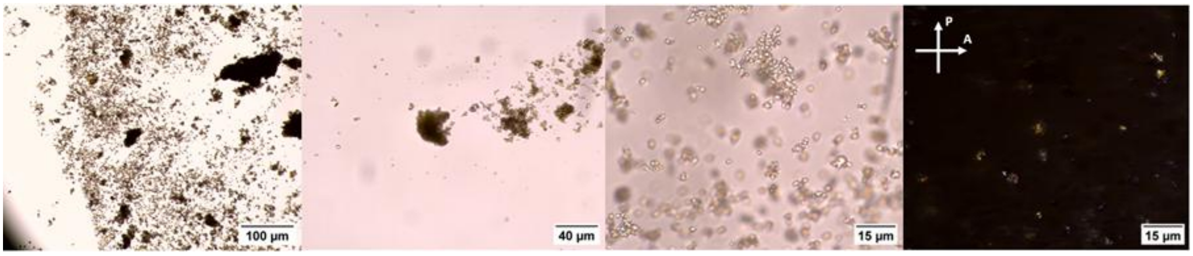

Figure S137 Optical micrographs of CF^{BBB} at 10×, 20×, and 50× magnification (*first, second and third figure*). The polarised optical micrograph at 50× (*last figure*) shows birefringence, indicating the presence of anisotropic domains consistent with crystalline behaviour. "A" denotes the analyser direction; "P" denotes the polariser direction.

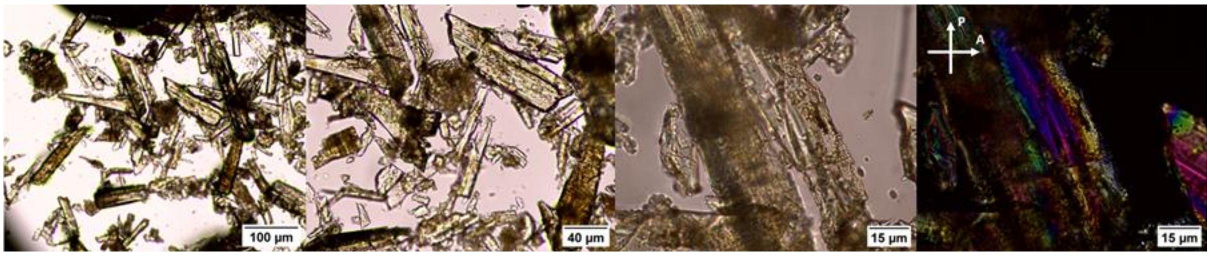

Figure S138 Optical micrographs of CF^{CCC} at 10×, 20×, and 50× magnification (*first, second and third figure*). The polarised optical micrograph at 50× (*last figure*) shows birefringence, indicating the presence of anisotropic domains consistent with crystalline behaviour. "A" denotes the analyser direction; "P" denotes the polariser direction.

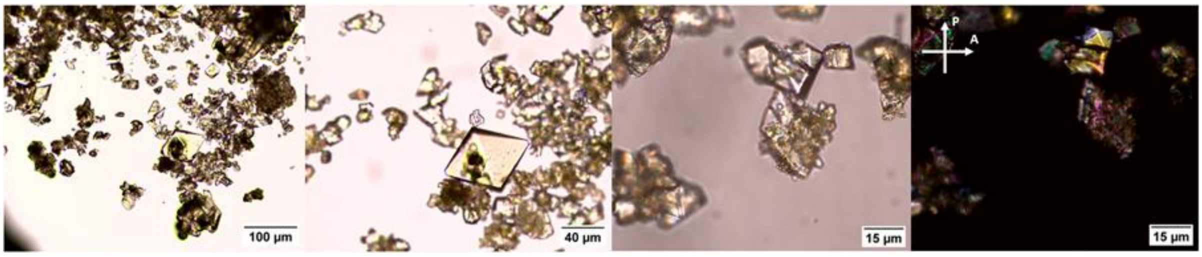

Figure S139 Optical micrographs of CF^{ABB} at 10×, 20×, and 50× magnification (*first, second and third figure*). The polarised optical micrograph at 50× (*last figure*) shows birefringence, indicating the presence

of anisotropic domains consistent with crystalline behaviour. “A” denotes the analyser direction; “P” denotes the polariser direction.

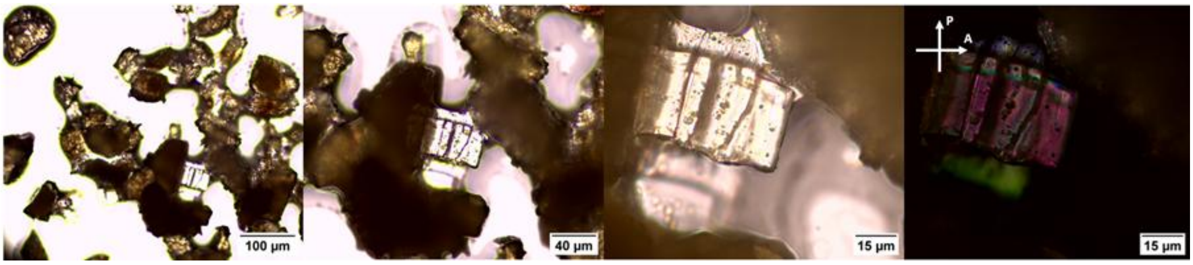

Figure S140 Optical micrographs of CF^{ABC} at 10×, 20×, and 50× magnification (*first, second and third figure*). The polarised optical micrograph at 50× (*last figure*) shows birefringence, indicating the presence of anisotropic domains consistent with crystalline behaviour. “A” denotes the analyser direction; “P” denotes the polariser direction.

S8. References

[S1] CrysAlisPro, Rigaku Oxford Diffraction, 2024 & 2025.

[S2] D. R. Allan et al., *Crystals* **2017**, *7*(11), 336.

[S3] J. Beilsten-Edmands et al., *Acta Cryst.* **2020**, *D76*, 385-399; P. Evans, *Acta Cryst.* **2006**, *D62*, 72-82; P. R. Evans and G. N. Murshudov, *Acta Cryst.* **2013**, *D69*, 1204-1214; M. D. Winn et al., *Acta Cryst.* **2011**, *D67*, 235-242, G. Winter, *J. Appl. Cryst.* **2010**, *43*, 186-190; G. Winter et al., *Acta Cryst.* **2018**, *D74*, 85-97.

[S4] O. V. Dolomanov, L. J. Bourhis, R. J. Gildea, J. A. K. Howard, H. Puschmann, *J. Appl. Crystallogr.* **2009**, *42*, 339-341.

[S5] G. M. Sheldrick, *Acta Cryst.* **2015**, *A71*, 3-8.

[S6] G. M. Sheldrick, *Acta Cryst.* **2015**, *C71*, 3-8.
